# Sequencing and *De Novo* Assembly of the Asian Clam (*Corbicula fluminea*) Transcriptome Using the Illumina GAIIx Method 

**DOI:** 10.1371/journal.pone.0079516

**Published:** 2013-11-07

**Authors:** Huihui Chen, Jinmiao Zha, Xuefang Liang, Jihong Bu, Miao Wang, Zijian Wang

**Affiliations:** State Key Laboratory of Environmental Aquatic Chemistry, Research Center for Eco-Environmental Sciences, Chinese Academy of Sciences, Beijing, China; National University of Singapore, Singapore

## Abstract

**Background:**

The Asian clam (*Corbicula fluminea*) is currently one of the most economically important aquatic species in China and has been used as a test organism in many environmental studies. However, the lack of genomic resources, such as sequenced genome, expressed sequence tags (ESTs) and transcriptome sequences has hindered the research on *C. fluminea*. Recent advances in large-scale RNA-Seq enable generation of genomic resources in a short time, and provide large expression datasets for functional genomic analysis.

**Methodology/Principal Findings:**

We used a next-generation high-throughput DNA sequencing technique with an Illumina GAIIx method to analyze the transcriptome from the whole bodies of *C. fluminea*. More than 62,250,336 high-quality reads were generated based on the raw data, and 134,684 unigenes with a mean length of 791 bp were assembled using the Velvet and Oases software. All of the assembly unigenes were annotated by running BLASTx and BLASTn similarity searches on the Nt, Nr, Swiss-Prot, COG and KEGG databases. In addition, the Clusters of Orthologous Groups (COGs), Gene Ontology (GO) terms and Kyoto Encyclopedia of Gene and Genome (KEGG) annotations were also assigned to each unigene transcript. To provide a preliminary verification of the assembly and annotation results, and search for potential environmental pollution biomarkers, 15 functional genes (five antioxidase genes, two cytochrome P450 genes, three GABA receptor-related genes and five heat shock protein genes) were cloned and identified. Expressions of the 15 selected genes following fluoxetine exposure confirmed that the genes are indeed linked to environmental stress.

**Conclusions/Significance:**

The *C. fluminea* transcriptome advances the underlying molecular understanding of this freshwater clam, provides a basis for further exploration of *C. fluminea* as an environmental test organism and promotes further studies on other bivalve organisms.

## Introduction

The freshwater bivalve *Corbicula fluminea* (Müller, 1774), commonly known as the Asian clam, is native to Southeast Asia [1]. In China, *C. fluminea* is widely distributed in rivers and lakes, such as the Yangtze River and the lakes Hongze (HZ), Poyang (PY) and Taihu (TH). As an important aqua-cultural shellfish, *C. fluminea* is currently one of the most economically important aquatic species in China with a yearly output of more than 12 million tons. *C. fluminea* meat is thought to have beneficial effects on the liver and is valued for its nutritional and pharmacological activity (e.g., the amelioration of hypercholesterolemia; hepato-protective, antioxidant and anticancer activities; protection against fatty liver induced by exposure to xenobiotics; and increased ethanol metabolism) [2,3]. This species underwent a massive global range expansion over the last century and is considered as an invasive species in North America and Europe because it can potentially endanger native aquatic communities [4-7].

Recently, many reports have studied this genus to analyze its growth, reproduction and immunity [8-11]. *C. fluminea* has also been used as a test organism in many field and laboratory studies for monitoring environmental changes and contamination [12-19]. As a benthic species, *C. fluminea* is a suspension feeder that filter-feeds on unicellular algae, bacteria and detrital particles and thus is extensively used as a bioindicator of water pollution [16]. Several genes related to pollution and stress response have been cloned as biomarkers [20,21]. However, the lack of genomic resources, such as a completed genome, expressed sequence tags (ESTs) or transcriptome sequences, limited research on *C. fluminea*. As of April 2013, there were only 12 *C. fluminea* ESTs available in the NCBI database and only 184 genes have been assembled and annotated (http://www.ncbi.nlm.nih.gov/). *De novo* transcriptome approaches are increasingly viable for organisms lacking a sequenced genome, although the sequencing of complex genomes remains expensive [22,23]. 

The fields of transcriptomics and genome characterization have developed rapidly with the advent of next-generation high-throughput sequencing technologies (such as the Illumina (Illumina), 454 (Roche) and SOLiD (ABI) platforms) in recent years [24-26]. Among the next-generation sequencing technologies, Illumina sequencing is cost-effective and yields longer sequencing reads and higher throughput than other methods in transcriptome analysis [27]. To date, next-generation sequencing methods have been applied to only a few mollusk species, such as *Mytilus galloprovincialis* [28], *Bathymodiolus azoricus* [29], Yesso scallop (*Patinopecten yessoensis*) [30] and *Ruditapes philippinarum* [31]. Most of these studies used the 454 method, and the studied species were marine bivalves. Furthermore, the data on mollusks in the NCBI database are mainly for marine bivalves, e.g., the blue mussel *Mytilus edulis* and the Pacific oyster *Crassostrea. gigas* [32]. 

 In the present study, we constructed a cDNA library from the *C. fluminea* adult and sequenced it using the Illumina GAIIx platform. More than 67.1M Illumina paired-end reads and 5.9 G of high-quality data were generated with 134,684 unigenes were assembled. We further annotated the unigenes by matching them against Nt, Nr, Swissprot, Clusters of Orthologous Groups of Proteins (COG), Gene ontology (GO), and Kyoto Encyclopedia of Gene and Genome (KEGG). A subset of these unigenes that are related to antioxidase, cytochrome P450, gamma-aminobutyric acid (GABA) receptor and heat shock protein (HSP) genes were further annotated and partially verified because they were determined to be useful as environmental pollution biomarkers. Our database is expected to provide an invaluable resource for studies of the genome and the functional genes of *C. fluminea* and for future comparative genomic studies of other bivalves.

## Materials and Methods

### RNA isolation and Illumina sequencing

The Asian clam (*C. fluminea*) was collected in June 2012 from the natural populations of Hongze Lake, China. According to the Chinese law, this area is not considered protected and the clam is not considered an endangered/protected species. It is a field aqua-cultural shellfish in China with a yearly output of more than 12 million tons. We did our study under the license of the local government (Xuyi country, Jiangsu province) and in accordance with the law. No specific permissions were required for these locations/activities. We confirm that the field studies did not involve endangered or protected species. All the experimental procedures involving clam (clam collection, housing, care and collection of tissue samples for use) were conducted in accordance with the International Guiding Principles for Biomedical Research Involving Animals 2012 (Council for International Organizations of Medical Sciences, http://www.cioms.ch) and approved by the institutions animal care and use committee of the Research Center for Eco-Environmental Sciences, Chinese Academy of Sciences.

Tissues including mantle, muscle, digestive gland, gonad and gill were dissected from adult clams (20.56±8.05 mm) (mean±SD, n=30). Total RNA was extracted from these samples using TRIzol reagent (Invitrogen) followed by RNA purification with the RNeasy MiniElute Cleanup Kit (Qiagen) according to the manufacturer’s protocol. The total RNA concentration and quality were determined and quantified using a QUBIT 2.0 fluorometer and an Agilent 2100 BioAnalyzer. A total RNA sample of approximately 0.1-0.4μg with RIN ≥8 and a 260/280 nm absorption ratio ≥1.8 was obtained.

For mRNA library construction and deep sequencing, RNA samples were prepared using the TruSeq RNA Sample Preparation Kit according to the manufacturer’s protocol. The library quality was assessed using an Agilent 2100 bioanalyzer and quantified using QUBIT and qPCR analyses. The cluster formation and sequencing on the GAIIx platform were performed according to the manufacturer’s standard cBot and sequencing protocols. For multiplex sequencing, 100 cycles of single read 1 were used to sequence the RNA followed by 7 cycles of index identification and 100 cycles of single read 2. Primary data analysis and base calling were performed by the Illumina instrument software.

### Transcriptome assembly and advanced data analysis

A custom Perl program was used to remove low-quality sequences from raw data sequences. The high quality reads were then assembled using Velvet (Velvet_1.1.07; kmer =57) and Oases to construct unique consensus sequences [33,34]. The trimmed Solexa transcriptome reads were mapped onto the unique consensus sequences using SOAP2 [35]. A custom Perl script was used to process the mapping results and generate the gene expression profile.

Nt and Nr: Unigenes were compared with the NCBI non-redundant nucleotide database (Nt, as of March 4^th^, 2012) and non-redundant protein database (Nr, as of March 4^th^, 2012) using BLASTn and BLASTx, respectively, with the same E-value cutoffs <1e^-5^ [36]. Swiss-Prot: Unigenes were identified by sequence similarity comparison against the Swiss-Prot database (Swiss-Prot downloaded from European Bioinformatics Institute as of March 4^th^, 2012) with BLAST at E values <1e^-10^ [37]. COG: Unigenes were assigned a functional annotation by sequence similarity comparison against the COG protein database using BLAST at E values <1e^-10^ [38,39]. A custom Perl script was used to assign a functional class to each unigene. KEGG: Unigenes were first compared with the Kyoto Encyclopedia of Genes and Genomes database (KEGG, release 58) using BLASTX at E values <1e^-10^ [40]. A Perl script was used to retrieve KEGG Orthology (KO) information from the BLAST result and then establish pathway associations between unigenes and the database. Interpro and gene ontology: InterPro domains were annotated using InterProScan Release 27.0, and functional assignments were mapped onto the GO structure [41]. WEGO was used to perform GO classifications and construct the GO tree [42]. 

### ORF identification and SSR discovery

The ORFs (Open reading frame) of the unigenes were predicted using the getorf program of EMBOSS to determine the longest ORF of each gene [43].The MISA program (http://gramene.agrinome.org/db/searches/ssrtool) was used to identify the simple sequence repeats (SSRs). All dinucleotide to hexanucleotide SSRs were searched using the default settings of the SSRIT tool.

### RT-PCR Assays

To verify the assembly and annotation results and to identify potential environmental pollution biomarkers, a total of 15 assembly sequences of the predicted transcripts were selected to be confirmed and analyzed using RT-PCR assays. For RT-PCR assays, the 15 genes were amplified with GoTaq® GreenMaster Mix (Promega, USA) using cDNA from *C. fluminea* digestive gland as template in a Veriti thermal cycle system (Life Technology, USA). The thermal cycle parameters were as follows: 94°C for 3 min followed by 35 cycles of 94°C for 30 s, 53°C for 30 s, and 72°C for 1 min and a final extension step at 72°C for 7 min. The PCR products were analyzed using agarose gel electrophoresis, and sub cloned into pGEM-T vector (Promega, USA) for Sanger sequencing.

### Fluoxetine Exposure and Real-time Quantitative PCR (RT-qPCR) verification

A fluoxetine exposure experiment was done to determine if the 15 genes are indeed linked to environmental stress. Fluoxetine is present in the antidepressant Prozac^®^, the most widely prescribed psychoactive drug in the market. Fluoxetine acts as a selective serotonin reuptake inhibitor (SSRI) and has been frequently reported to have disruptive effects in non-target species [44,45]. After 7 days acclimatizing in aerated natural water, the clams in the experimental groups were exposed to Fluoxetine hydrochloric (TCI, Tokyo Chemical Industry Co., Ltd., Japan; product number F0750, >98%; CAS: 56296-78-7). The final fluoxetine concentrations in the water were 0.05, 0.5 to 5 µg/L which were refer to the concentrations of the fluoxetine in the aquatic environment and correspond to concentrations used in previous studies examining the toxicity of fluoxetine to bivalves [46,47]. Three replicates were prepared for both the control and fluoxetine treated groups. The aquaria were kept at constant temperature (20±1°C), pH (7.8±0.2) and oxygen saturation (96±2%) in 30 L glass aquaria (30 individuals per aquaria).Water was changed daily and fresh doses of fluoxetine were added; clams were fed every 48h with *Chlorella vulgaris* and *Scenedesmus obliquus*. There was no observed mortality during the experimental period. After 30 days exposure, the digestive gland in the control and treated groups were collected. At least three independent biological replicates for each sample were harvested. Total RNA was isolated using SV Total RNA Isolation System (Promega, USA) according to the manufacturer’s protocols. The quantity and quality of total RNA was confirmed as before.

 Expressions of the 15 identified genes in the control and fluoxetine exposure clams were measured by real time quantitative PCR (RT-qPCR). Primers for each target gene were designed with Primer Express software v3.0.1 (Life Technology, USA). We used β-actin as an internal control [20]. Specificity of primers sets throughout the range of detection was confirmed by the observation of single amplification products of the expected size and Tm, and optimized by performing a standard curve for each primer pair. Over the detection range, the linear correlation (R^2^) between the mean Ct and the logarithm of cDNA dilution was >0.99 (range from 0.992 to 0.999) in each case, and efficiencies were between 93.1% to 106.8% [48]. The sequences, PCR product sizes, annealing temperatures and PCR efficiencies for each primer pair are shown in Table 1. The cDNA was synthesized according to manufacturer’s instructions from 2 µg of total RNA treated with RQ1 DNase (Promega, USA), using random hexamers and M-MLV reverse transcriptase (Promega). A total of 2 μg RNA from each tissue was reverse transcribed in a final volume of 25 μL and incubated for 1 h at 37°C after an initial denaturation step at 70°C for 10 min. The cDNAs were stored at -20°C until further use. qPCR amplification was performed using an ABI 7500 real-time quantitative PCR system (Life Technology, USA) in a total volume of 25 µL, consisting of the GoTaq® SYBR Green qPCR Master Mix (Promega, USA). The thermal cycle parameters used were as follows: 2 min at 95°C, 40 cycles of 15 s at 95°C and 1 min at 60°C. To confirm that only one PCR product was amplified and detected, a melting curve analysis of amplification products was performed at the end of each PCR reaction. Results were analyzed based on the delta-delta Ct method [49]. Experiments were performed in triplicate and repeated three times with similar results. Statistical analysis was performed with the SPSS (version 16.0; USA) and OriginPro^®^ (version 8.0). One-way ANOVA (*p*<0.05) was performed using OriginPro^®^ to test the differences of gene expressions between control and fluoxetine treated clams.

**Table 1:** Analyzed genes and their specific primers.

Genes	Accession No.	Realtime-PCR Primer sequence (5'-3')	Product Size(bp)	Annealing Temperatures(°C)	PCR efficiency
*Cfβ-actin*	EF446608.1	F:CGCCATCCAGGCTGTGCTTTCA	123	55.0	97.0%
		R:ATGGCGTGTGGAAGGGCGTA			
*Cf(Cu/Zn)SOD*	KF218347	F:CCACCTTGTCTGGATTGTAGTG	98	55.0	98.5%
		R:GTCTGGCTGCTGGAGAACAT			
*CfGPx-A*	KF218345	F:TCCTGTGATGATGTGGAGAGC	80	55.0	106.1%
		R:TGGGAAGTCAAACTGGGACG			
*CfGST-mu*	KF218346	F:GGATGGTGAAACAGAGAGGGA	88	55.0	93.1%
		R:AACAGAGCCTAACAACGCCG			
*CfTPX1*	KF218348	F:ATCGCTGGGTCACTCTTTACA	188	55.0	103.1%
		R:AAATGCCACAACTTCAGTCGG			
*CfTPX2*	KF218349	F:TGCTCTGAATGGAACTCCCTC	104	58.0	95.5%
		R:GGTGGTCTGGGCAGTATGAA			
*CfCYP4*	KF218340	F:GCTGAAACCAAGCGAGAGGT	130	56.0	95.2%
		R:ATCCCATAACAACTGCGGGT			
*CfCYP30*	KF218341	F:CCAGGAGAGACGACAAGACA	108	55.5	96.1%
		R:GGGTTGCTGCTTCAGGTTCA			
*CfGABAT1*	KF218344	F:ACGAGATTCCAGTAAGGCAGTT	166	58.0	99.4%
		R:ACGCACCTCCTCCGTTTT			
*CfGABARAP*	KF218343	F:AAAGCCCGTGTAGGAGACTTG	150	56.5	106.8%
		R:AAGAGATCCCATAGTCGCACT			
*CfGABARAPL2*	KF218342	F:TGTAGAGCGAGATCCAAAGTCA	197	55.0	93.6%
		R:AGAAATCCATCGTCATCCTTGT			
*CfHsp22*	KF218338	F:CTTTCAACCTGGAGCATTGTG	154	57.0	96.1%
		R:CGTGATTTGTTTTCGGACTG			
*CfHsp40*	KF218339	F:ACAAGCAGCATCCCAGATTCC	82	56.5	99.9%
		R:TCCAGTCAGAGCCTTTCCCA			
*CfHsp60*	KC979065	F:GGGACAACAGAAAGAACACCC	120	55.0	97.9%
		R:CCGACACGACCAAAGTCATT			
*CfHsp70*	KC979064	F:CGCCGACGCTGATTACCTTA	84	55.0	95.6%
		R:AAACGGTTGATAGGACGCAAG			
*CfHsp90*	KC979063	F:ATGGTGCTGGAATCTCTCAGG	246	55.0	101.6%
		R:GCGTCACAAAGGAAGGACTG			

## Results and Discussion

### Illumina sequencing and assembly

**Figure 1:**
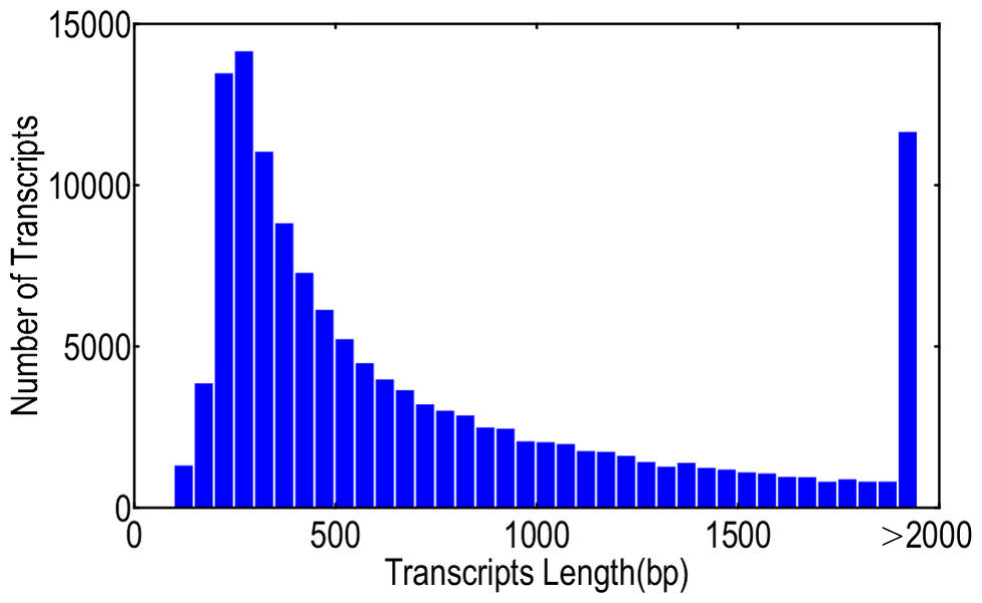
Assembly length statistic. Among these unigenes, 65,979 (49.0%) were longer than 500 bp, and 34,248 (25.4%) of this subset were longer than 1,000 bp.

We performed Illumina GAIIx platform sequencing of a normalized cDNA library prepared from different tissues of multiple *C. fluminea* individuals to develop a comprehensive understanding of the molecular mechanisms governing *C. flumineas* genome biology and to obtain as many gene transcripts as possible. Sequencing generated 67,087,130 transcriptomic reads consisting of 6,708,713,000 bp of raw data. From the reads, 62,250,336 high-quality reads and 5,898,595,168 bp (5.9G) of high-quality data (87.92% of raw data) were generated based on the raw data under the standard of Q20 (Q20 is the quality scores of the Illumina sequencing, sequencing error rate <1%). The high-quality data were aligned and *de novo* assembled using Velvet and Oases into 134,684 unigenes consisting of 106,542,508 bp. Unigenes ranged in size from 100 to 12,367 bp with an average length of 791 bp and N50 length of 1,264 bp (Table 2). Among these unigenes, 65,979 (49.0%) were longer than 500 bp, and 34,248 (25.4%) of this subset were longer than 1,000 bp (Figure 1). The reads were submitted to the Sequence Read Archive (SRA) at NCBI under the accession number SRA062349.

**Table 2:** Summary of sequence assembly.

No. Unigenes	Total length (bp)	Average length (bp)	N50 (bp)	Max length (bp)	Min length (bp)
134,684	106,542,508	791.06	1,264	12,367	100

The Illumina GAIIx method has been successfully used for the *de novo* assembly of transcriptomes in many species [50-54]. As compared with other recent studies, our results indicated that the Illumina GAIIx platform can provide much more data than the traditional Sanger sequencing method. The average size of the unigenes in our present study were 791 bp larger than those produced in previous studies using Illumina and 454 technologies (e.g., 618 [30], 546 [31], 367 [50], 396 [51], 223 [52] and 474 [53]). 

### Annotation of unigenes

To provide putative annotations for the assembly, all of the assembled unigenes were evaluated using BLASTx and BLASTn similarity searches against the Nt, Nr, Swiss-Prot, COG and KEGG databases (Table 3 and Table S1). A total of 24,767 unigenes (18.4% of the total) were matched in the Nt databases, and 38,985 unigenes (28.9% of the total) matched in the Nr databases with an E value <1e^-5^. Additionally, 27,849 unigenes (20.7% of the total) can be matched to the Swiss-Prot databases with an E value <1e^-10^ (Table 3).

**Table 3:** Unigene annotation statistics of the *C. fluminea* transcriptome.

Database	# of Annotated	%	E Cutoff	Database Version
Total Unigenes	134,684	100		
Nt	24,767	18.4	1 x 10^-5^	20120304
Nr	38,985	28.9	1 x 10^-5^	20120304
Swiss-Prot	27,849	20.7	1 x 10^-10^	20120304
COG	14,035	10.4	1 x 10^-10^	No version
KEGG	32,042	23.8	1 x 10^-10^	Release 58
Interpro	25,408	18.9	Interproscan 4.8	v36
GO	20,686	15.4	Interproscan 4.8	v36

Annotation information could not be assigned for a large percentage of the sequences obtained in this study. The poor annotation efficiency could have been due to the lack of sequences in public databases for species that are phylogenetically closely related to *C. fluminea* [30]. Only 40 (0.03%) unigenes were matched to *C. fluminea*. For matches to the Bivalve class in the Nr database, the greatest number of the matched unigenes (0.4%) showed similarities with *Ruditapes philippinarum* followed by *M. galloprovincialis* (0.39%), *Crassostrea gigas* (0.35%), *Chlamys farreri* (0.21%) and *Haliotis discus discus* (0.17%). The low number of matches indicates a lack of bivalve data in public databases.

### COG, CO and KEGG classification

**Figure 2:**
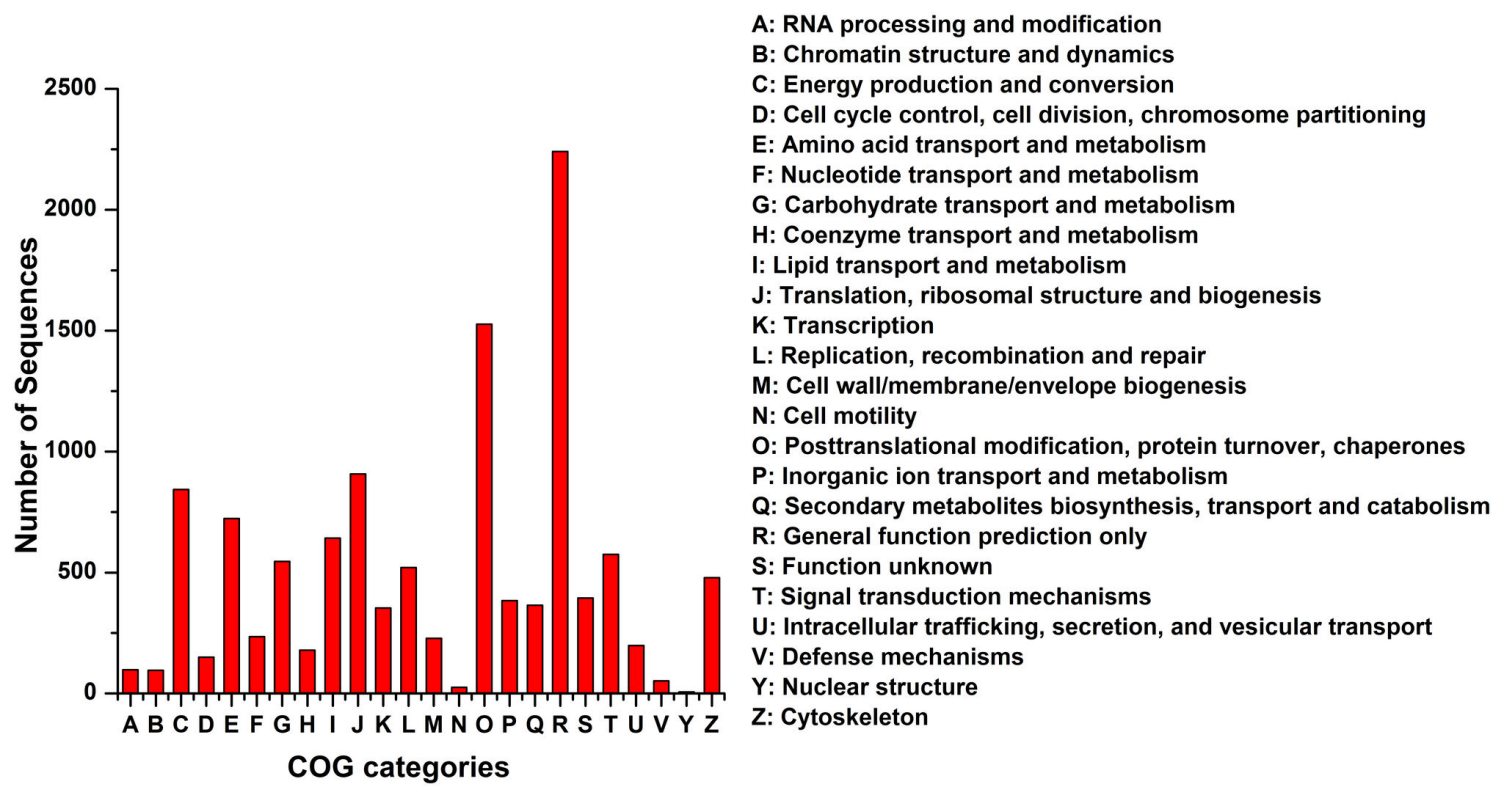
COG classification of the unigenes. Possible functions of 11,771 unigenes were classified and subdivided into 24 COG categories.

The Clusters of Orthologous Groups (COGs) of proteins were generated by comparing the protein sequences of complete genomes. Each cluster contains proteins or groups of paralogs from at least three lineages [38]. The current COG database contains both prokaryotic clusters and eukaryotic clusters [39]. We aligned the unigenes to the COG databases to find homologous genes and classify possible functions of the unigenes (Figure 2). A total of 14,035 unigenes (10.4% of the total) had a match in COG database with an E value <1e^-10^ (Table 3). The possible functions of 11,771 (83.87% of COG matched) unigenes were classified and subdivided into 24 COG categories (Table S2). The largest group was ‘General function prediction only’ (2241, 19.04%), followed by ‘Post-translational modification, protein turnover, chaperones’ (1527, 12.97%) and ‘Translation, ribosomal structure and biogenesis’ (908, 7.71%). 

**Figure 3:**
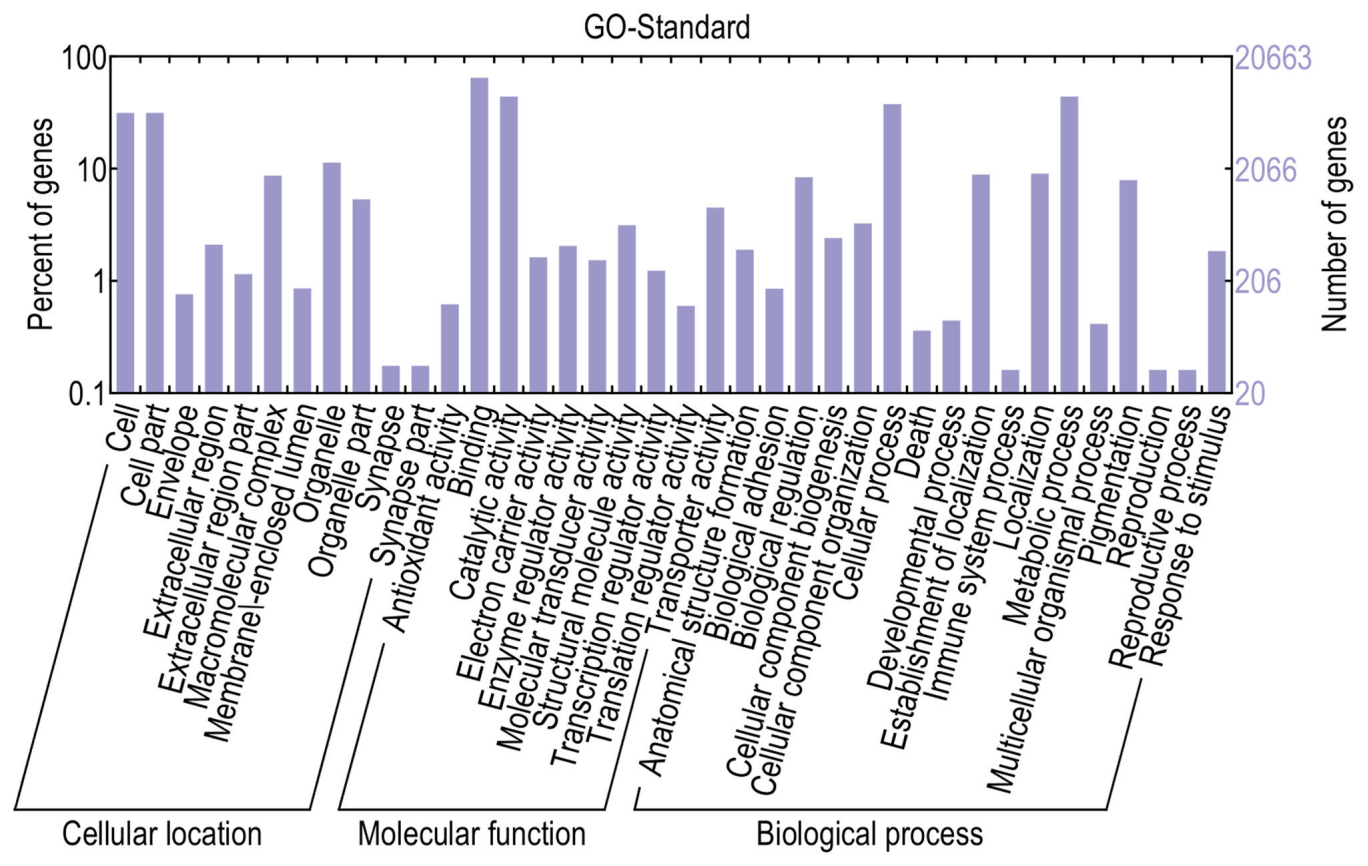
Classification of *C. fluminea* sequences based on predicted Gene Ontology (GO) terms. In total, 20,286 unigenes were assigned GO terms in 46 functional groups and three categories, including 19,167 unigenes at the cellular component level, 25,414 unigenes at the molecular function level and 26,279 unigenes at the biological process level.

GO is an international standardized gene functional classification system and covers three domains: cellular component, molecular function and biological process. The InterPro domains were annotated by InterProScan Release 27.0, and functional assignments were mapped onto the GO structures. In total, 20,686 unigenes were matched to a GO annotation (Table 3). We used WEGO to perform the GO classifications and draw the GO tree to facilitate the classification of the *C. fluminea* transcripts into putative functional groups. In total, 20,286 unigenes were assigned GO terms in 46 functional groups and three categories (Table S3), including 19,167 unigenes at the cellular component level, 25,414 unigenes at the molecular function level and 26,279 unigenes at the biological process level (Figure 3). Within the cellular component category, cell (6,447) and cell part (6,447) were the most highly represented groups. Binding (13,252) and catalytic activity (9,019) were most abundant groups within the molecular function category. A total of 22 GO functional groups were assigned into the biological process category, among which metabolic process (9,021) and cellular process (7,726) were the most highly represented.

Based on comparative analyses using the KEGG database, 32,042 unigenes (23.8% of the total) were found to have a match with an E value <1e^-10^ using BLASTx (Table 3). We used a Perl script to retrieve KO information from the BLAST result, establish pathway associations between unigenes and the database and then match these 32,042 sequences to 253 different KEGG pathways (Table S4). Of these 32,042 sequences with KEGG annotation, 10,389 were classified into metabolism groups, with most of them involved in amino acid metabolism, carbohydrate metabolism, lipid metabolism and energy metabolism. The greatest number of sequences were classified into the genetic information processing pathways (9,373), followed by human diseases (6,036), cellular processes (4,862) and environmental information processing (3,199).

Over all, the possible functions of the assembled unigenes were assessed by similarity matches with the COG, CO and KEGG databases. The results of these databases searches help us better understand the biological features of *C. fluminea*. The patterns of the *C. fluminea* found in this study were common and similar to other organisms [23,30,31,50]. 

### ORF identification and SSR discovery

**Figure 4:**
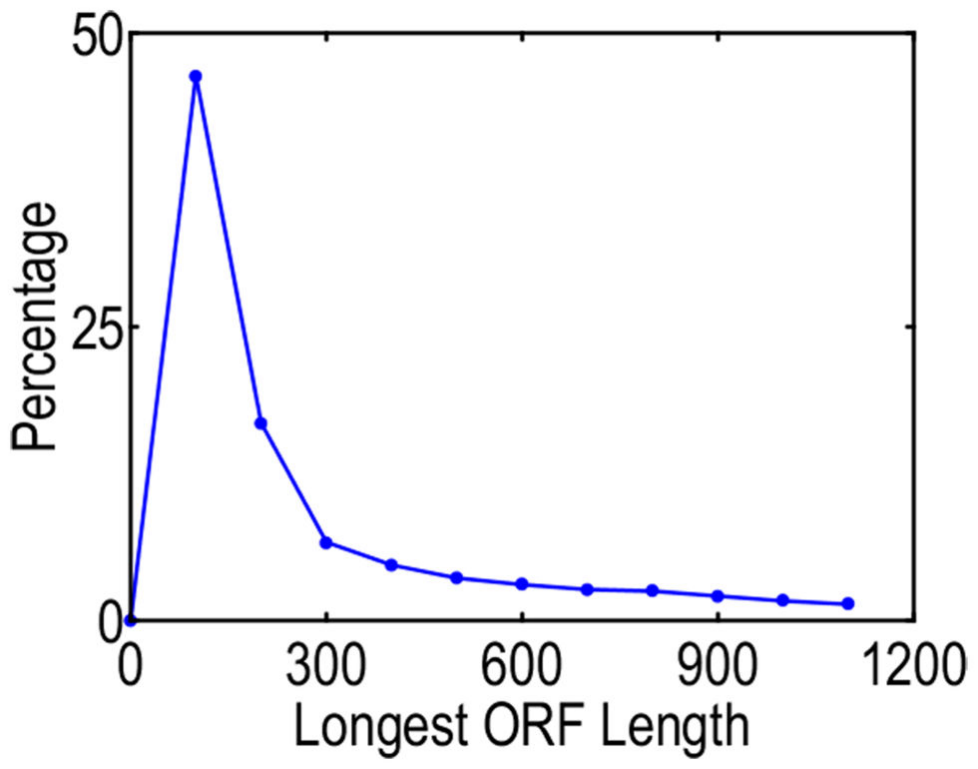
ORF lengths of the *C. fluminea* transriptome.

The “getorf” function of EMBOSS software was used to identify the ORFs of the assembled sequences. Of the 134,684 assembled *C. fluminea* unigene sequences, 105,737 (78.50%) had an ORF longer than 100 bp, with an average length of 445 bp (min length = 102, max length = 11,592, Figure 4). 

 The application of marker-assisted selection (MAS) or genome-wide marker-assisted selection (G-MAS) can be employed in the *C. fluminea* breeding program. Currently, a limited collection of genetic markers is available for *C. fluminea*. In this study, simple sequence repeats (SSRs) were identified. The putative and filtered SSRs *C. fluminea* are shown in Table S5. In total, 2,151 SSRs were identified from the assembled sequences (Table 4). Of 1,547 SSR-containing sequences, 340 SSRs were present in compound form, and 452 sequences contained more than one SSR. The most frequent repeat motifs were tri-nucleotides, which accounted for 57.83% of all SSRs, followed by di-nucleotides (31.38%), tetra-nucleotides (7.53%), penta-nucleotides (3.11%) and hexa-nucleotides (0.14%). These SSRs would be top candidates for marker development and very helpful for further research involving population genetic structuring, relatedness, genetic or genomic studies on this species.

**Table 4:** Summary of simple sequence repeat (SSR) types in the *C. fluminea* transcriptome.

**SSR Type**	**No. of SSRs**	**% of total SSRs**
Di-nucleotides	675	31.38
Tri-nucleotides	1244	57.83
Tetra-nucleotides	162	7.53
Penta-nucleotides	67	3.11
Hexa-nucleotides	3	0.14
Total	2151	100%

### Identification and validation of putative biomarker transcriptomic sequences

To verify the assembly and annotation results and to identify potential environmental pollution biomarkers, 15 related assembled unigenes, including five antioxidase genes (Cu/Zn superoxide dismutase, *Cu/Zn SOD*), glutathione peroxidase A (*GPx-A*) and mu glutathione S-transferase (*GST-mu*), thioredoxin peroxidase 1(*TPX1*) and thioredoxin peroxidase 2 (*TPX2*), two cytochrome P450 genes (*CYP4* and *CYP30*), three GABA receptor-related genes (GABA neurotransmitter transporter 1, *GABAT1*; GABA_A_ receptor-associated protein, *GABARAP*; and GABA_A_ receptor-associated protein-like 2, *GABARAPL2*) and five HSP genes (*Hsp22*, *Hsp40*, *Hsp60*, *Hsp70* and *Hsp90*), were selected and subjected to RT-PCR and real-time PCR analyses (Table S6).

The antioxidant defense system (including SOD, GPx, GST and TPX) is very effective at reducing damage when oxidative stress has occurred. SOD catalyzes the transformation of superoxide radicals to H_2_O_2_ and O_2_ and represents the initial response to oxyradicals. GPx then catalyzes the reduction of H_2_O_2_. GST belongs to a multifunctional family of cytosolic enzymes involved in phase II biotransformation, which plays an important role in protecting tissues from oxidative stress. Eight classes of GST isoenzymes can be distinguished based on their substrate specificity, immunological properties and protein sequences homology [55]. Thioredoxin peroxidase (TPX) is a member of proteins that are conserved from yeast to mammals and to which natural killer enhancing factor belongs. These proteins are antioxidants that function as peroxidases only when coupled to a sulfhydryl reducing system.  Previous studies have suggested that *SOD*, *CAT, Se-GPx* and *GST-pi* transcript levels in *Corbicula fluminea* could be used as biomarkers of Cu and Cd exposure in aquatic environments [55,56]. The identification of *Cu/Zn SOD*, *GPx-A*, *GST-mu*, *TPX1* and *TPX2* genes in the present study would allow for the use of *C. fluminea* antioxidant defense system assays in pollutant monitoring.

The cytochrome P450s (CYPs) comprise one of the largest and most versatile protein families in living organisms and are involved in a variety of detoxification and endogenous functions [57,58]. In bivalve mollusks, the CYPs are poorly studied, as there only a few *CYP* genes with known regulatory mechanisms that have been identified in mussel and oyster, including *CYP1-like* and *CYP3-like* genes. The study of *CYP1A* in *C. fluminea* showed that this gene was a useful biomarker for exposure to PCBs [59]. The *CfCYP4* and *CfCYP30* genes were identified based on transcript sequences obtained using specific primers in this present work. The identification of these genes will facilitate an improved understanding of CYPs in bivalves.

GABARAP, an important protein in the autophagy process, is evolutionarily conserved and is involved in innate immunity in eukaryotic cells. In invertebrates, GABARAP is the major inhibitory neurotransmitter in synapses of both the central and peripheral nervous systems [60]. The GABARAP subfamily consists of GABARAP, GABARAPL1, GABARAPL2 and GABARAPL3. Recently, the GABARAP and related genes have been isolated from several mollusks, such as *Lymnaea stagnalis* and *Haliotis diversicolor* [61,62]. However, the actual biological functions of GABARAP remain elusive [62]. In this study, we identified three *C. fluminea GABARAPs*: *GABAT1*, *GABARAP* and *GABARAPL2*. These results will help us gain a better understanding of the molecular and physiological processes involving GABARAPs in mollusks.

HSPs are commonly used as biomarkers of exposure to various stressors (such as temperature, metal toxicity, toxic exposure and infection) [63]. These proteins comprise a group of highly conserved, yet highly diversified, proteins that primarily function as molecular chaperones, stabilizing protein folding and preventing indiscriminate protein interactions by sequestering unfolded proteins, which can be found in diverse organisms from bacteria to mammals [64]. The major HSP families are Hsp60, Hsp70, Hsp90 and the small HSPs family (sHsp) [65]. The sHsp family is a heterogeneous group of proteins of intermediate molecular weight (12-43kDa), such as Hsp22 and Hsp40.The Hsp70 protein family is considered to be the major HSP family and has been the most extensively studied. Under adverse environmental conditions, Hsp70 can improve expression levels and takes part in the defense, repair or detoxification machinery of the cell by tightly binding denatured proteins. In bivalve species, many studies have shown that the transcription of Hsp70 is simultaneously and differentially modulated upon exposure to environmental stressors [66,67]. Recent studies reported that Hsp90 can be regulated by a range of stressors such as food deprivation, heavy metal exposure and thermal shock [68-70]. In this study, we sought to identify sequences in the *C. fluminea* transcriptome that encode *Hsp22*, *Hsp40*, *Hsp60*, *Hsp70* and *Hsp90*. Based on the results of unigenes annotation (BLASTx with the E <1e^-5^), we identified five putative *HSP* (22, 40, 60, 70 and 90) sequences corresponding to unigenes loci.

**Figure 5:**
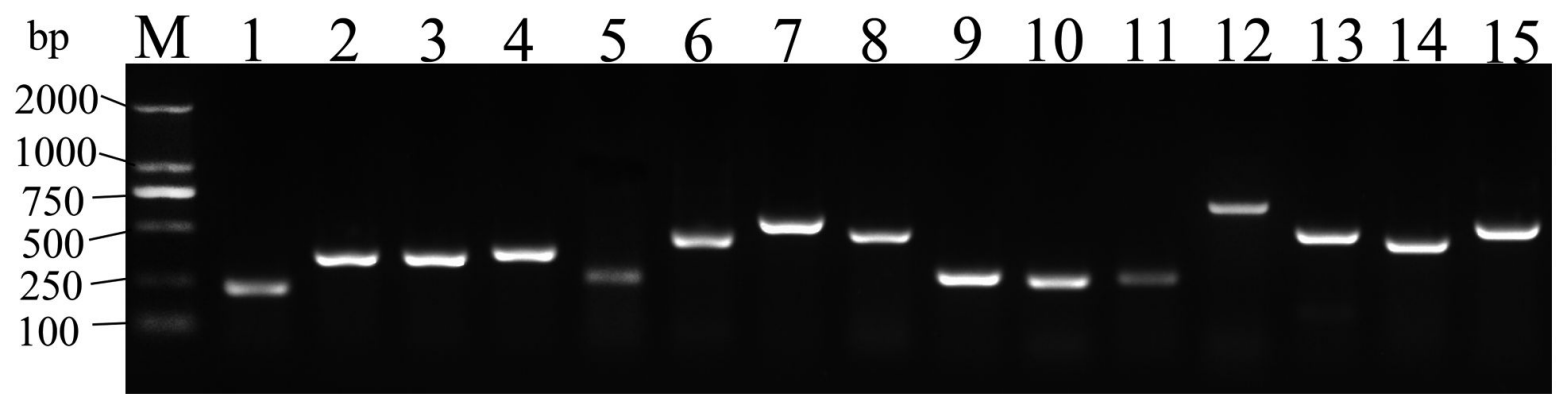
RT-PCR analyses of the 15 potential *C. fluminea* genes. PCR was performed using the cDNA prepared from adult clams. 1: Cf(Cu/Zn)*SOD*,234bp;2: *CfGPx-A*,372bp;3:*CfGST-mu*,369bp;4:*CfTPX1*,393bp;5:*CfTPX2*,273bp;6:*CfCYP4*,459bp;7:*CfCYP30*,612bp;8: *CfGABAT1*,510bp;9:*CfGABARAP*,276bp;10:*CfGABARAPL2*,264bp;11:*CfHsp22*,282bp;12: *CfHsp40*,672bp;13:*CfHsp60*,477bp;14:*CfHsp70*,467bp;15:*CfHsp90*,570bp.

Partial sequences for all 15 of the aforementioned potential biomarker genes were cloned from adult female *C. fluminea* digestive gland cDNAs and compared with the assembled sequences using RT-PCR. The products of RT-PCR were analyzed using agarose gel electrophoresis (Figure 5), RT-PCR primers and Sanger sequencing data are shown in Table S6. The consensus sequence data for the selected genes have been deposited with GenBank under the accession numbers shown in Table 1. The sequence variation was minimal (>99% nucleotide identity) and was associated with either the heterogeneity of the *C. fluminea* colony, which was annually outbred with local conspecifics, or with sequencing errors introduced during Sanger sequencing of the RT-PCR products. 

### Real-time Quantitative PCR (RT-qPCR) verification

**Figure 6:**
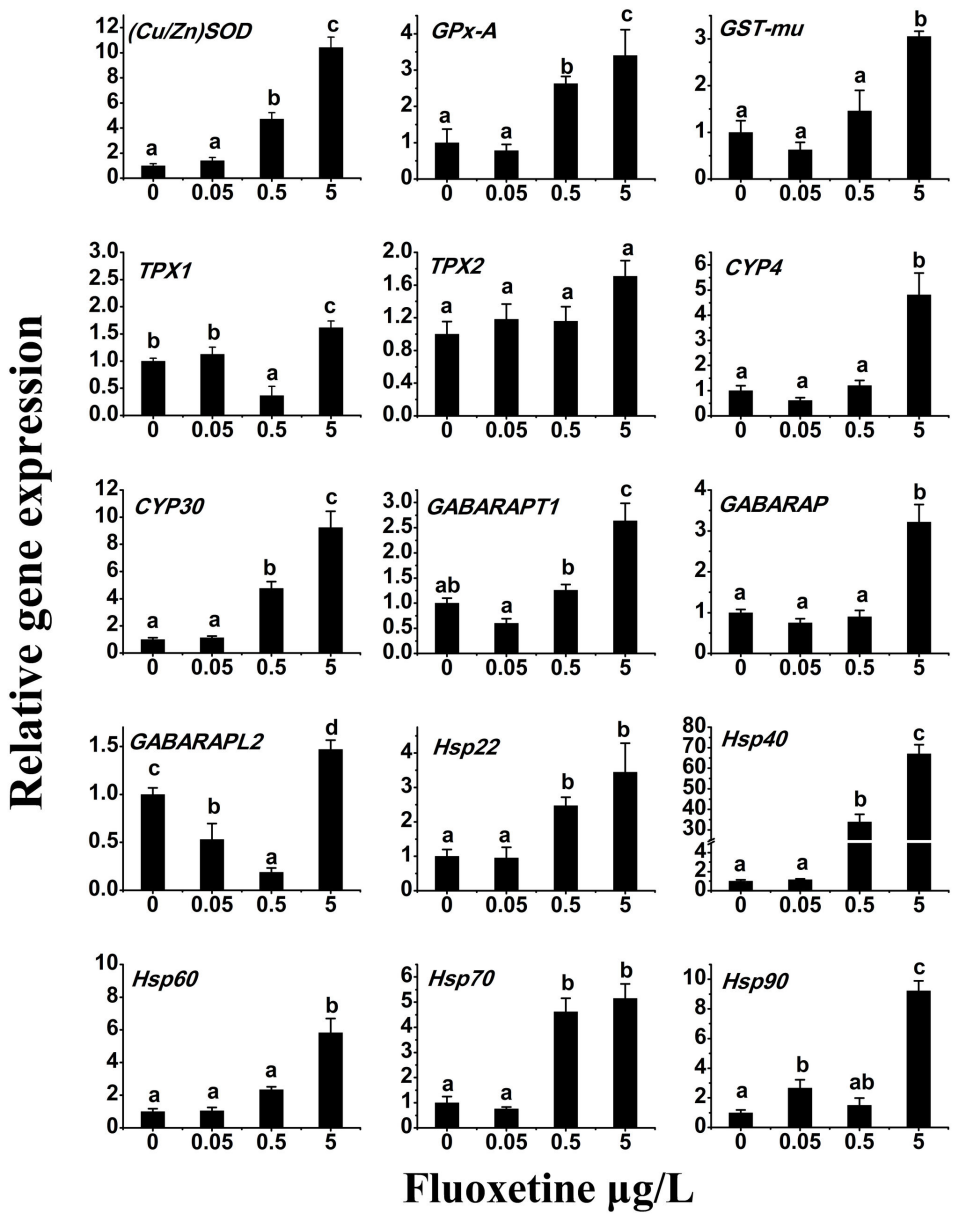
Real-time qPCR analyses of expression profiles of the 15 functional genes after 30 days fluoxetine exposure. Experiments were performed in triplicate and repeated three times with similar results. Bars display mean±S.D. One-way ANOVA (*p*<0.05) was performed using OriginPro^®^ to test the differences of gene expressions between control and fluoxetine treated clams.

Amplification products for the 15 aforementioned potential biomarker genes in the digestive gland of the *C. fluminea* following 30 days exposure to 0.05, 0.5 and 5 μg/L fluoxetine exposure are shown in Figure 6. 

For antioxidase genes, the mRNA expression levels of *(Cu/Zn*)* SOD* and *GPx-A* were significantly up-regulated in the 0.5 and 5 μg/L (*p*<0.05) fluoxetine groups. *GST-mu* was significantly increased (*p*<0.05) at 5 μg/L, while *TPX1* was only up-regulated (*p*<0.05) at the 5 μg/L. No significant change was observed in *TPX2* (Figure 6), indicating that *TPX2* gene is not sensitive to fluoxetine. Previous studies have reported that low concentration of fluoxetine (0.075 μg/L) exposure can affect antioxidant system in *M. galloprovincialis* [46]. In the present study, 0.5 and 5 μg/L fluoxetine exposure led to significant upregulation of the antioxidase genes (except *TPX2*) suggesting that the antioxidase genes will be useful biomarkers for exposure to environmental stress. 

*CYP4* expression peaked following 5 μg/L fluoxetine exposure with a 4.81-fold increase in transcript levels. *CYP30* was significantly (*p*<0.05) up-regulated at the 0.5 and 5 μg/L by 4.76-fold and 9.24-fold, respectively (Figure 6). As was previously reported, the *CYP3-like-2* gene was up-regulated in the digestive gland of *M. edulis* by PCB126 [58]. Our study found that the gene expressions of *CYP4* and *CYP30* were up regulated in digestive after fluoxetine exposure. Therefore, further studies are needed to discover the function of CYPs in the *C. fluminea*.

In this study, the *C. fluminea GABARAPs* genes were significantly up-regulated (*p*<0.05) with 5 μg/L fluoxetine exposure, while *GABARAPL2* was significantly decreased (*p*<0.05) (Figure 6). Although the actual biological functions of GABARAPs remain elusive [62], the results of our study showed the putative function of GABARAPs in *C. fluminea*.

Our present study found that the mRNA transcript for *Hsp22*, *Hsp40*, *Hsp60* and *Hsp90* were significantly increased by 0.5 and 5 μg/L with increases of 2.47-fold and 3.45-fold, 33.93-fold and 67.01-fold, 2.34-fold and 5.83-fold, 4.61-fold and 5.14-fold, respectively. The gene expression of *Hsp90* was significantly increased in the 0.05 μg/L (*p*<0.05) and 5 μg/L (*p*<0.05) fluoxetine exposure groups. No significant differences were observed in the most of the genes (except *Hsp90*) at the 0.05 μg/L fluoxetine exposure group (Figure 6). Many previous studies reported that various stressors (such as temperature, metal toxicity and infection) can affect the expression of *Hsp70* and *Hsp90* [66-70], However, the small Hsps (*Hsp22*, *Hsp40*) and *Hsp60* have been poorly studied. The present study showed that small Hsps, and *Hsp60* were also useful biomarkers when exposure to fluoxetine in *C. fluminea*.

Over all, the RT-qPCR results of the 15 selected genes after fluoxetine exposure confirmed that the 15 functional genes are linked to environmental stress. We can use the 15 genes as environmental biomarkers to monitor the environmental pollutants in the future. However, further research is needed to better understand the molecular mechanisms of *C. fluminea* following the contamination exposure.

## Conclusions

The currently available genetic information on *C. fluminea*, an important freshwater clam, is very limited. In the present study, we report the transcriptome sequencing and *de novo* genetic analysis of this species using the Illumina GAIIx method. More than 62,250,336 high-quality reads were generated based on the raw data, and 134,684 unigenes with a mean length of 791 bp were assembled. This transcriptional information provides a more nuanced understanding of the underlying biological and physiological mechanisms that govern *C. fluminea* biology. To our knowledge, this is the first report of transcriptome sequencing in this species. In addition, the identification of 15 putative environmental pollution biomarkers, and the gene expression results following fluoxetine exposure are further expected to promote research on the use of *C. fluminea* in environmental toxicology.

### Data Deposition

The Illumina GAIIx reads of *C. fluminea* were submitted to the NCBI Sequence Read Archive under accession number SRA062349. 

## Supporting Information

Table S1 **The total expressed unigenes and Annotation of Nt, Nr, SWISS-PROT, COG and KEGG databases.**
(XLSX)

Table S2 **COG Classification of the unigenes.**
(ZIP)

Table S3 **GO categories of the unigenes.**
(ZIP)

Table S4 **KEGG Classification of the unigenes.**
(ZIP)

Table S5 **The putative and filtered SSRs for *C. fluminea*.**
(ZIP)

Table S6 **The RT-PCR primers and Sanger sequencing of the 15 analyzed genes.**
(ZIP)
