# Supplementary figures and images for: Sequencing and De Novo Assembly of the Asian Clam (Corbicula fluminea) Transcriptome Using the Illumina GAIIx Method (part 2 of 2)
Source: PLoS One. 2013 Nov 7;8(11):e79516. doi: 10.1371/journal.pone.0079516 (PMC3820681; doi:10.1371/journal.pone.0079516)

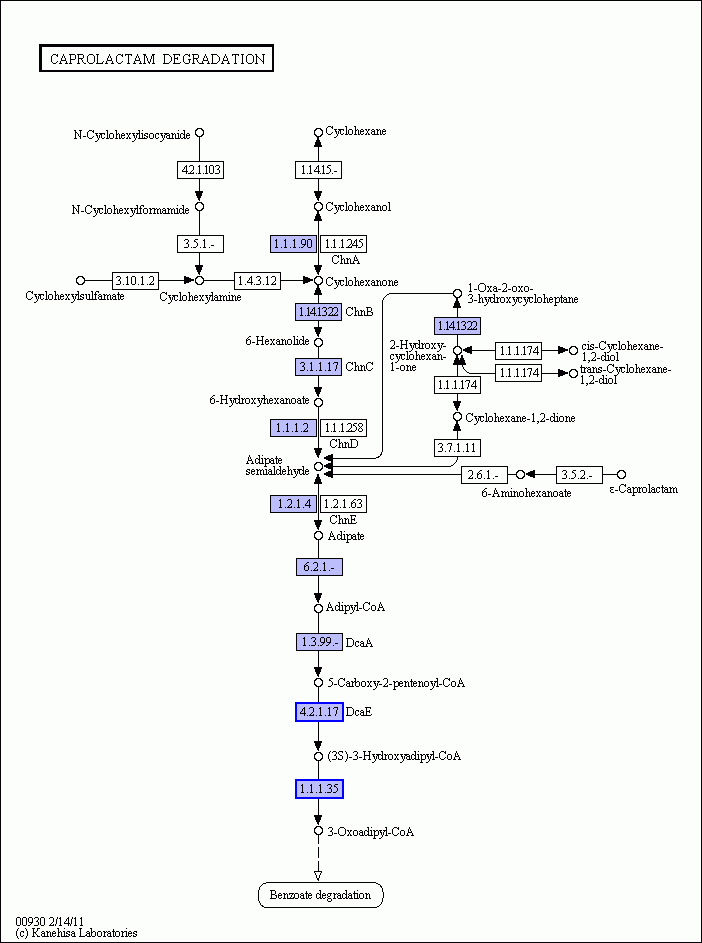

Supplement: Table S4 — KEGG Classification of the unigenes. (ZIP) [file pone.0079516.s004.zip › Kegg/Pathway_Map/ko00930.png]

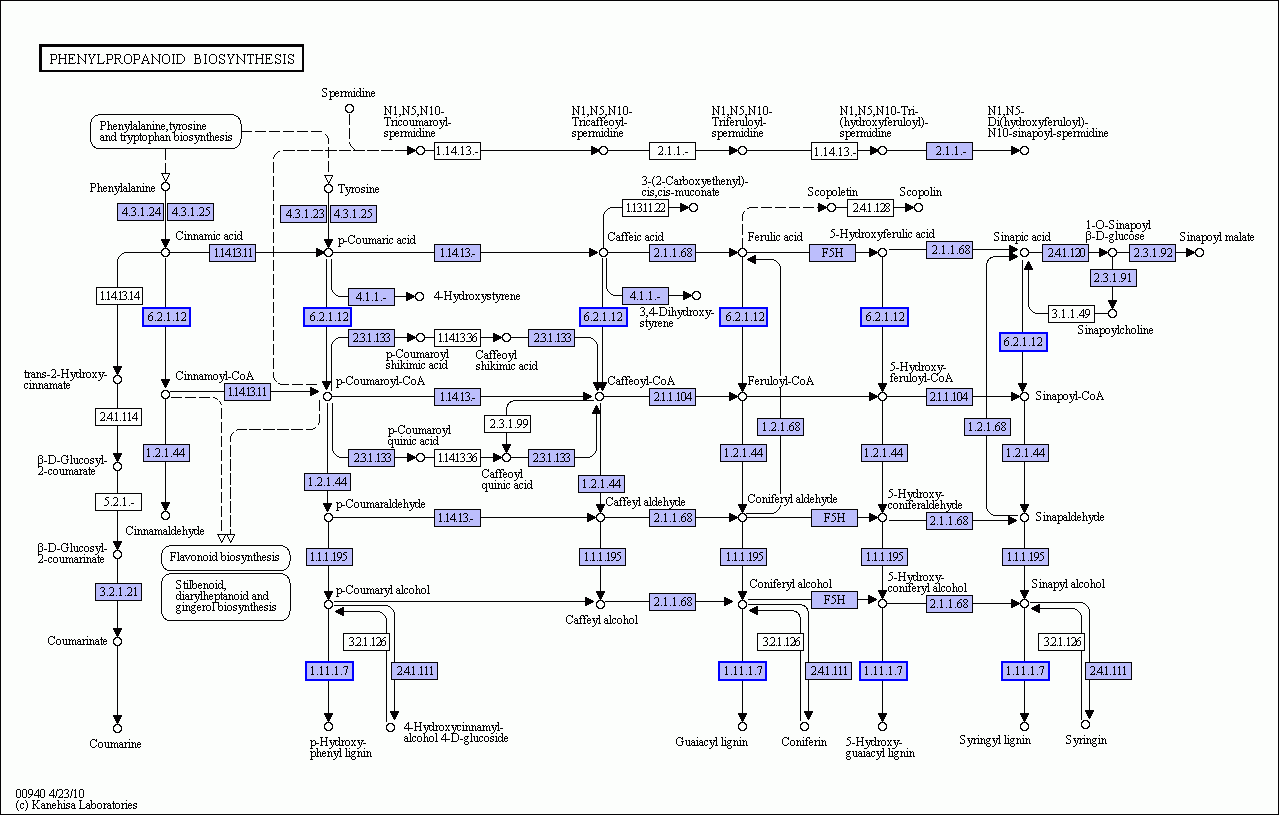

Supplement: Table S4 — KEGG Classification of the unigenes. (ZIP) [file pone.0079516.s004.zip › Kegg/Pathway_Map/ko00940.png]

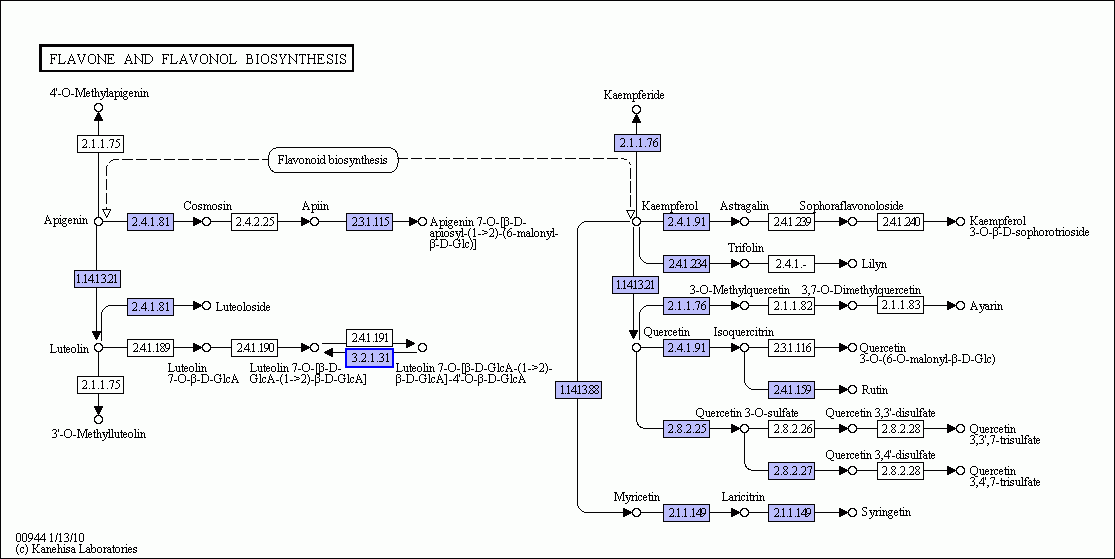

Supplement: Table S4 — KEGG Classification of the unigenes. (ZIP) [file pone.0079516.s004.zip › Kegg/Pathway_Map/ko00944.png]

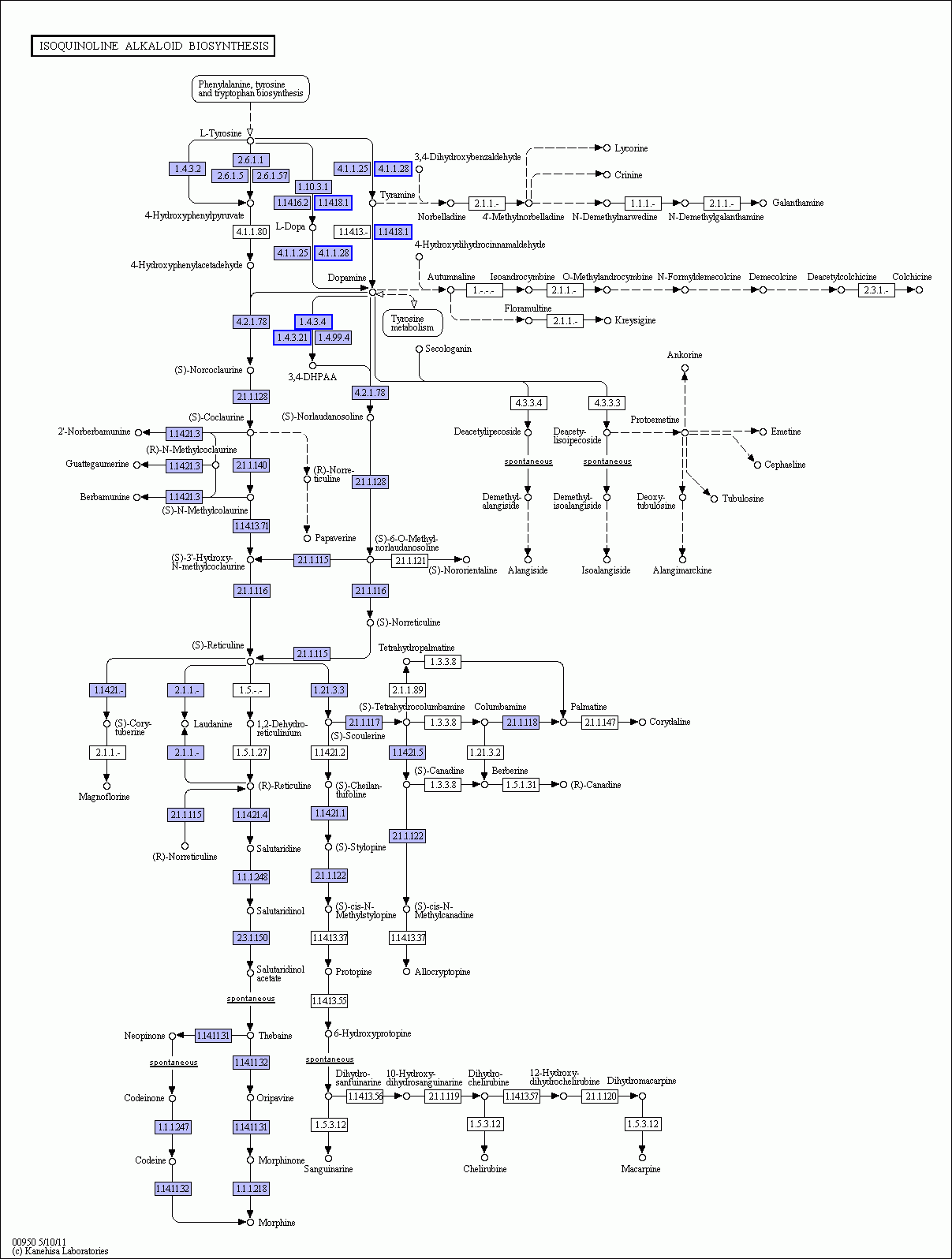

Supplement: Table S4 — KEGG Classification of the unigenes. (ZIP) [file pone.0079516.s004.zip › Kegg/Pathway_Map/ko00950.png]

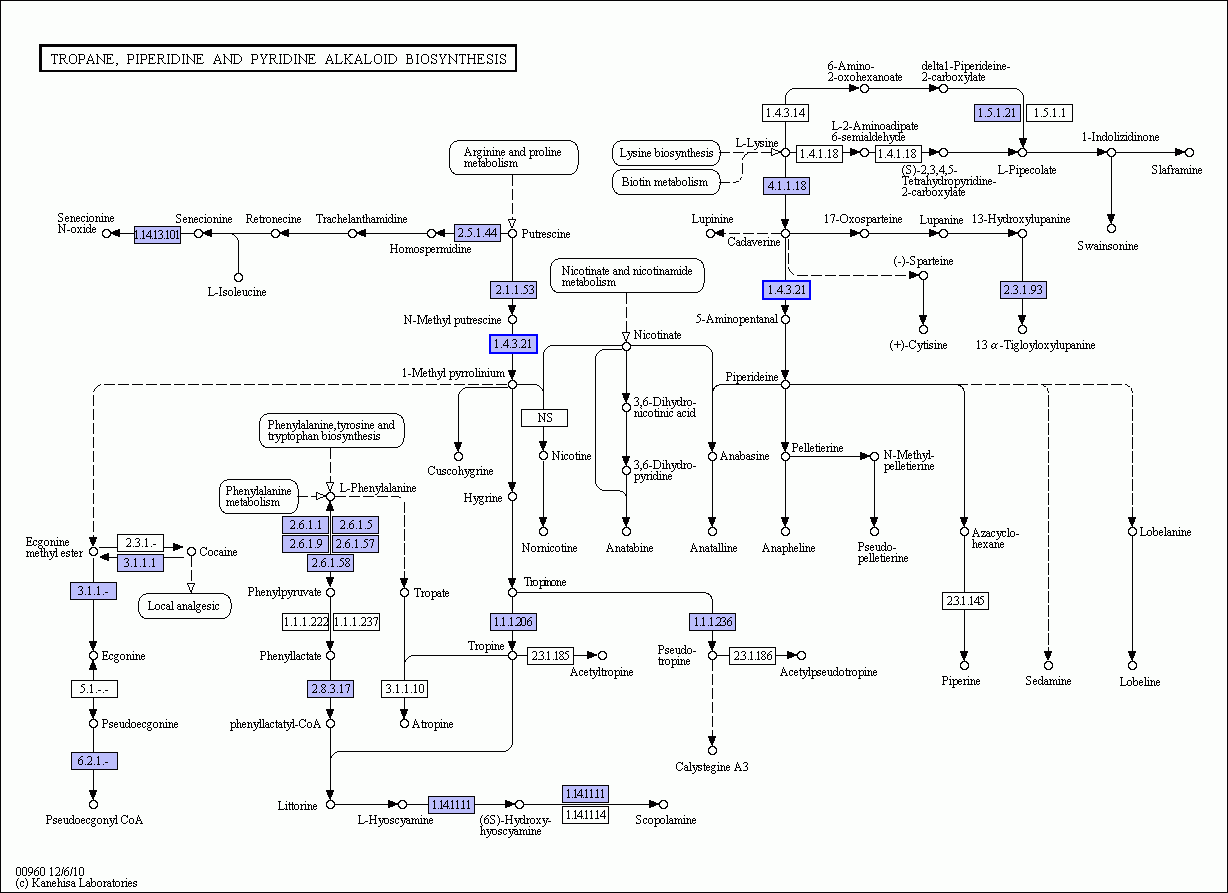

Supplement: Table S4 — KEGG Classification of the unigenes. (ZIP) [file pone.0079516.s004.zip › Kegg/Pathway_Map/ko00960.png]

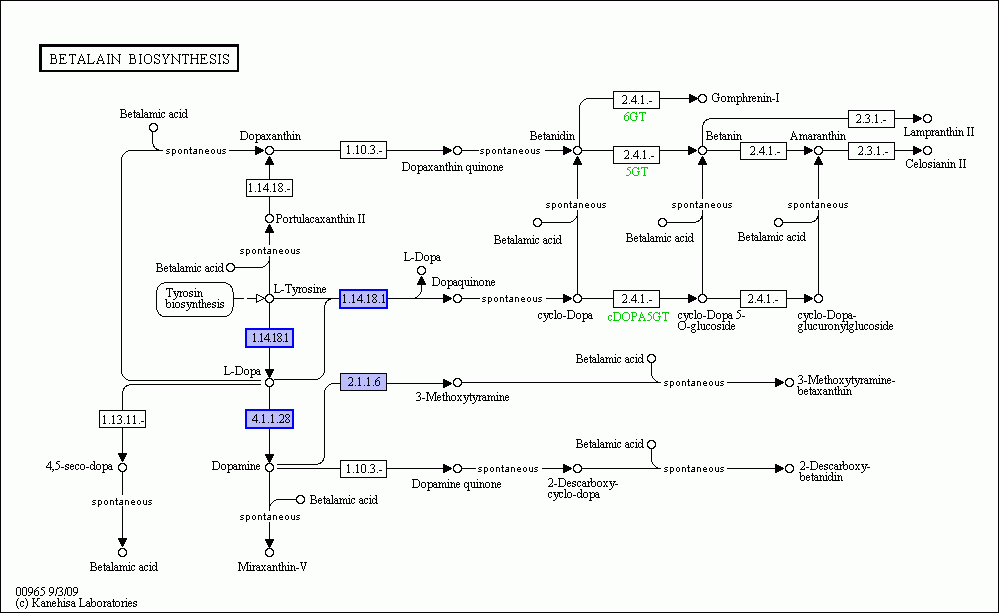

Supplement: Table S4 — KEGG Classification of the unigenes. (ZIP) [file pone.0079516.s004.zip › Kegg/Pathway_Map/ko00965.png]

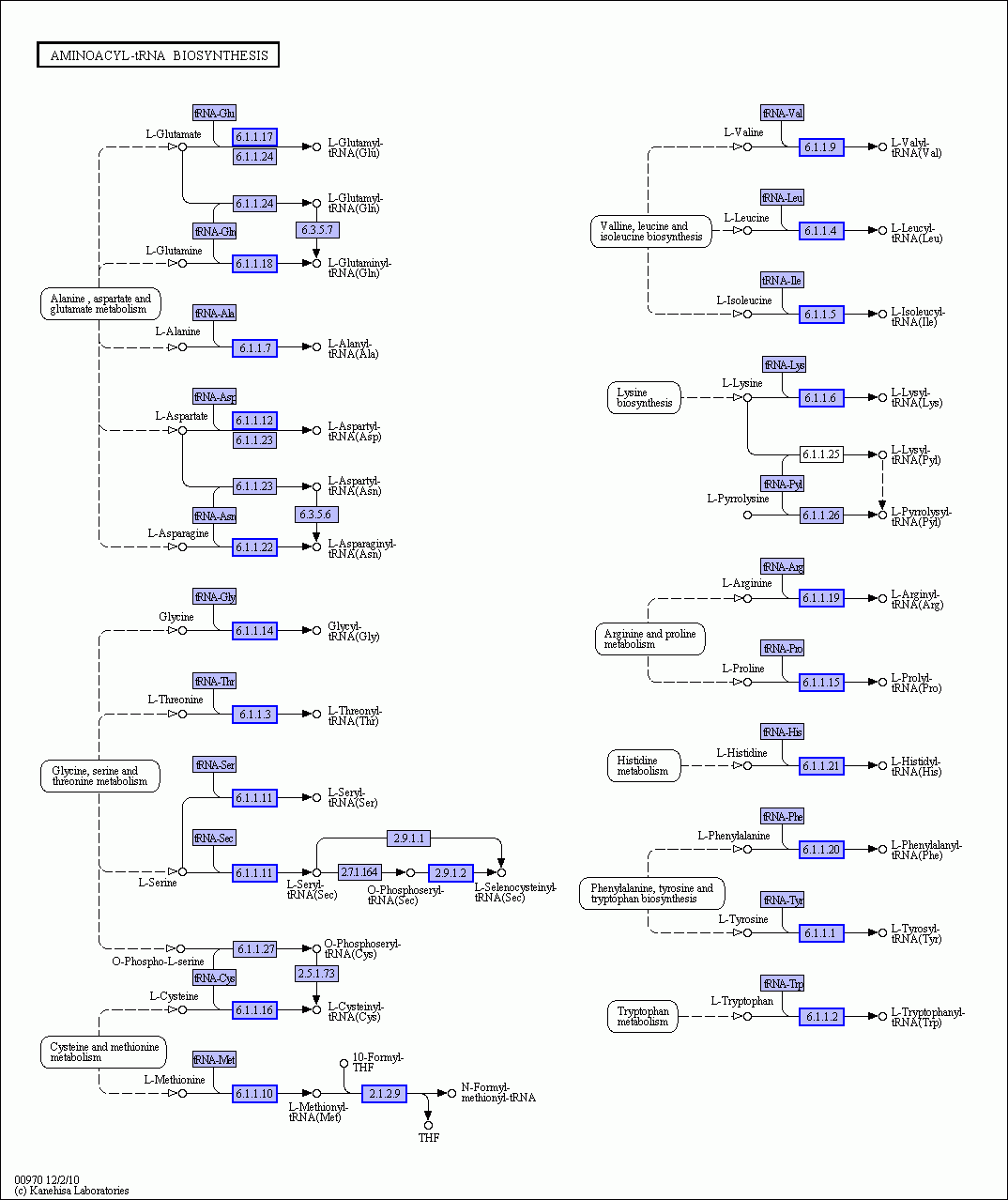

Supplement: Table S4 — KEGG Classification of the unigenes. (ZIP) [file pone.0079516.s004.zip › Kegg/Pathway_Map/ko00970.png]

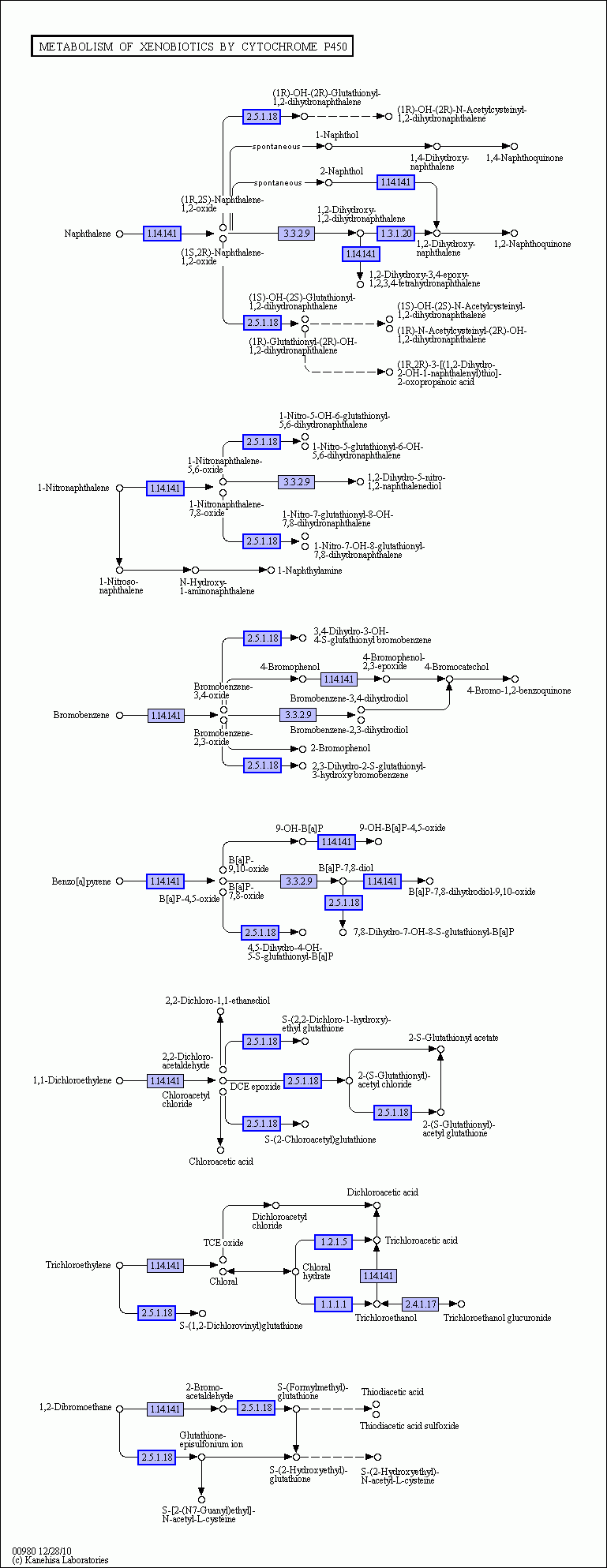

Supplement: Table S4 — KEGG Classification of the unigenes. (ZIP) [file pone.0079516.s004.zip › Kegg/Pathway_Map/ko00980.png]

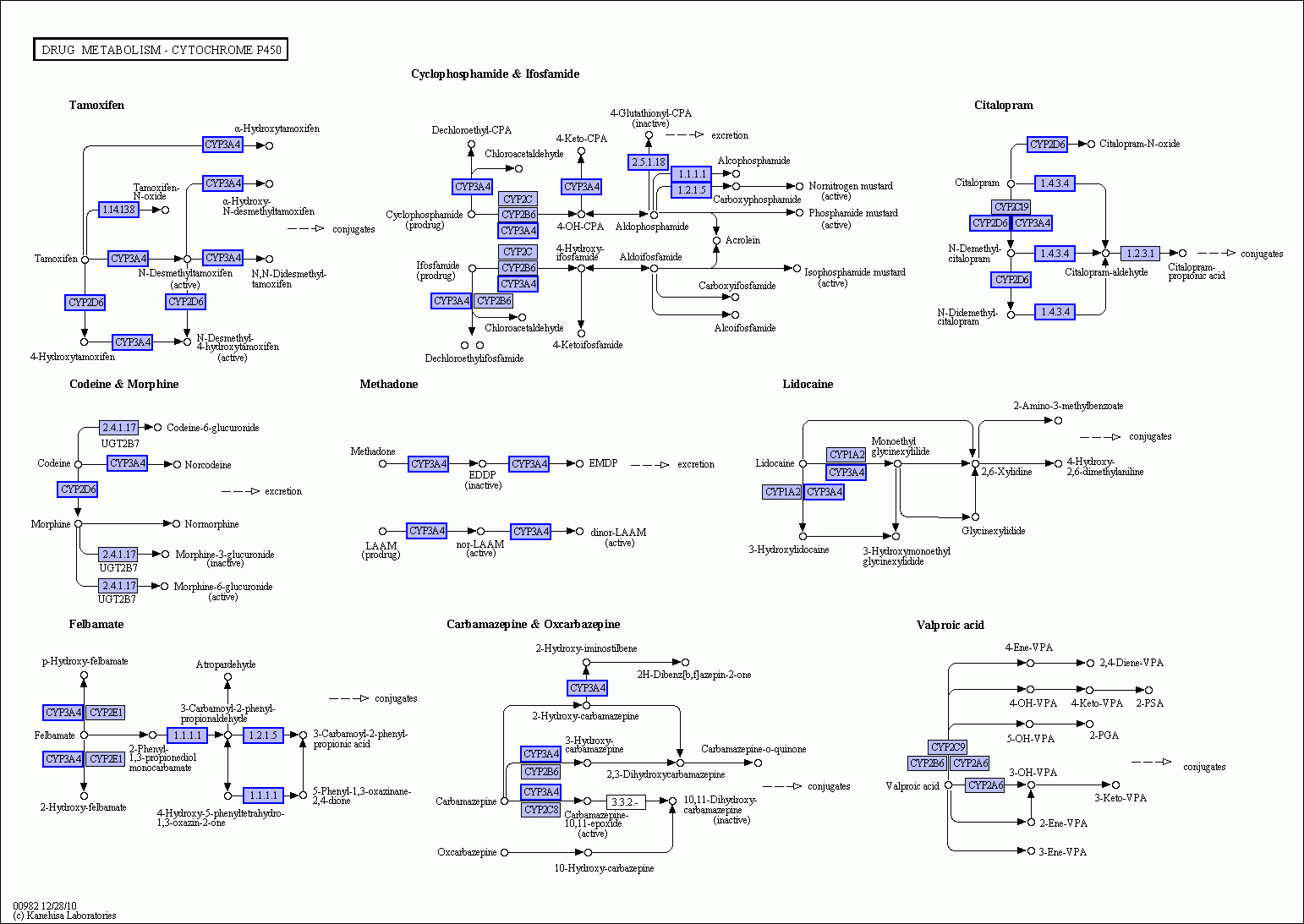

Supplement: Table S4 — KEGG Classification of the unigenes. (ZIP) [file pone.0079516.s004.zip › Kegg/Pathway_Map/ko00982.png]

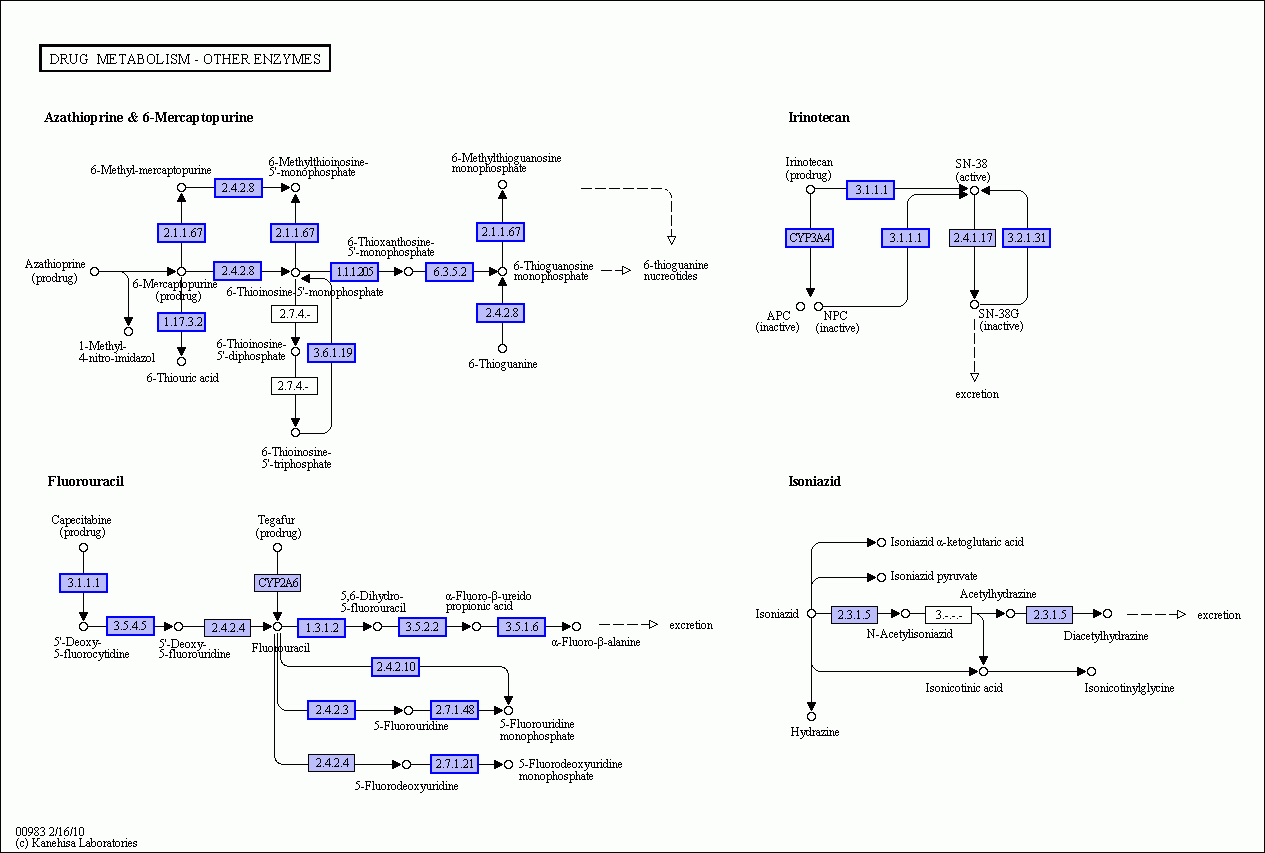

Supplement: Table S4 — KEGG Classification of the unigenes. (ZIP) [file pone.0079516.s004.zip › Kegg/Pathway_Map/ko00983.png]

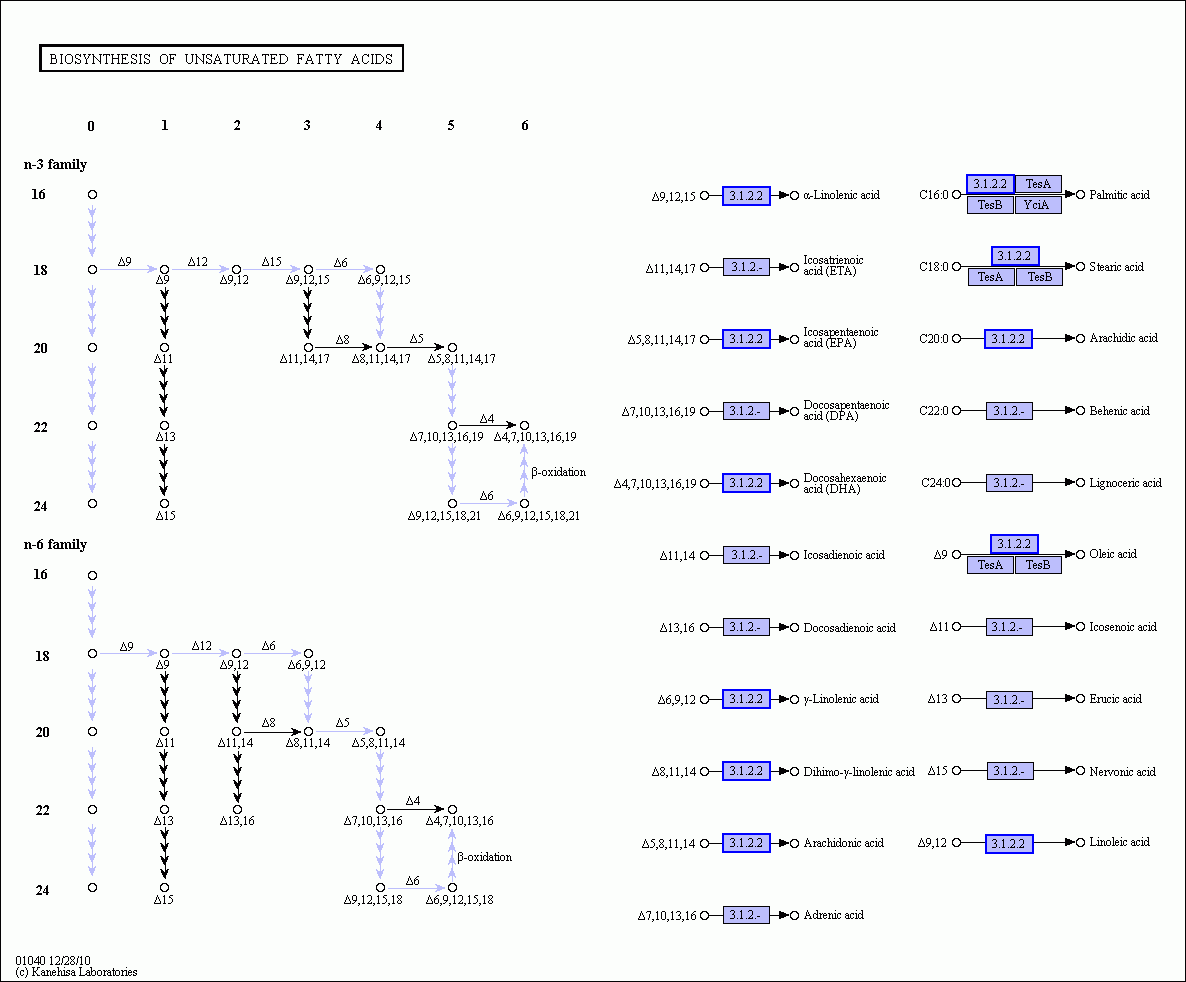

Supplement: Table S4 — KEGG Classification of the unigenes. (ZIP) [file pone.0079516.s004.zip › Kegg/Pathway_Map/ko01040.png]

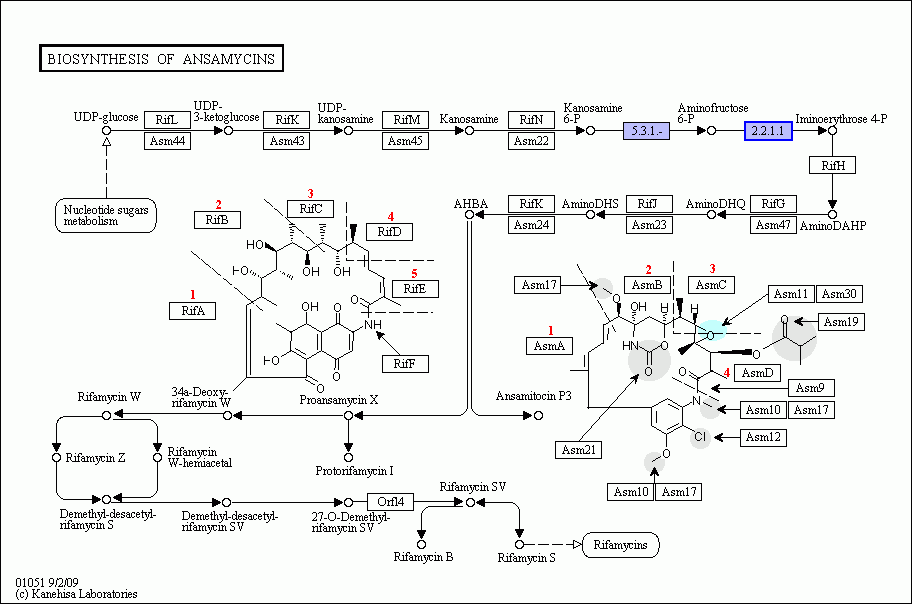

Supplement: Table S4 — KEGG Classification of the unigenes. (ZIP) [file pone.0079516.s004.zip › Kegg/Pathway_Map/ko01051.png]

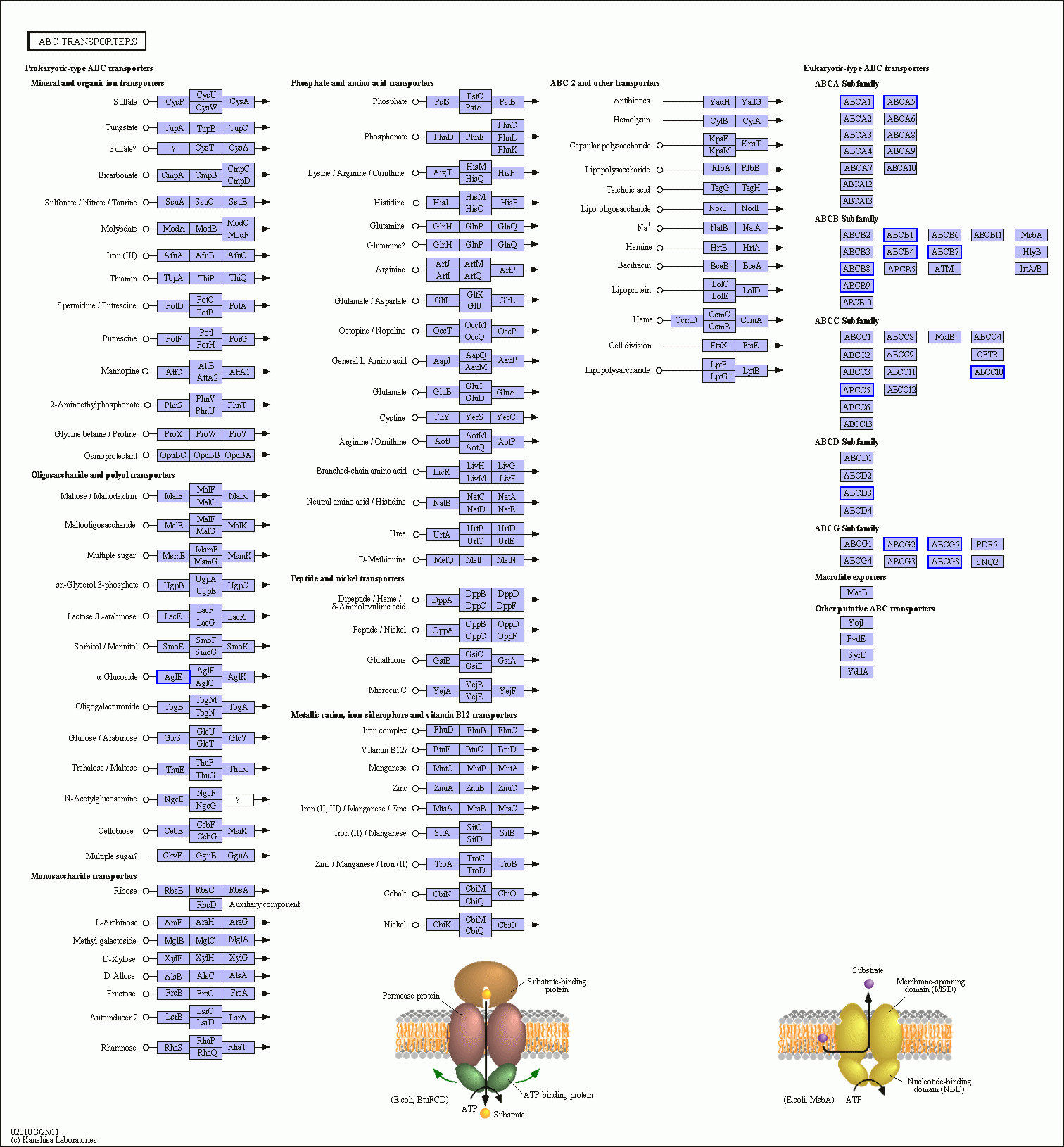

Supplement: Table S4 — KEGG Classification of the unigenes. (ZIP) [file pone.0079516.s004.zip › Kegg/Pathway_Map/ko02010.png]

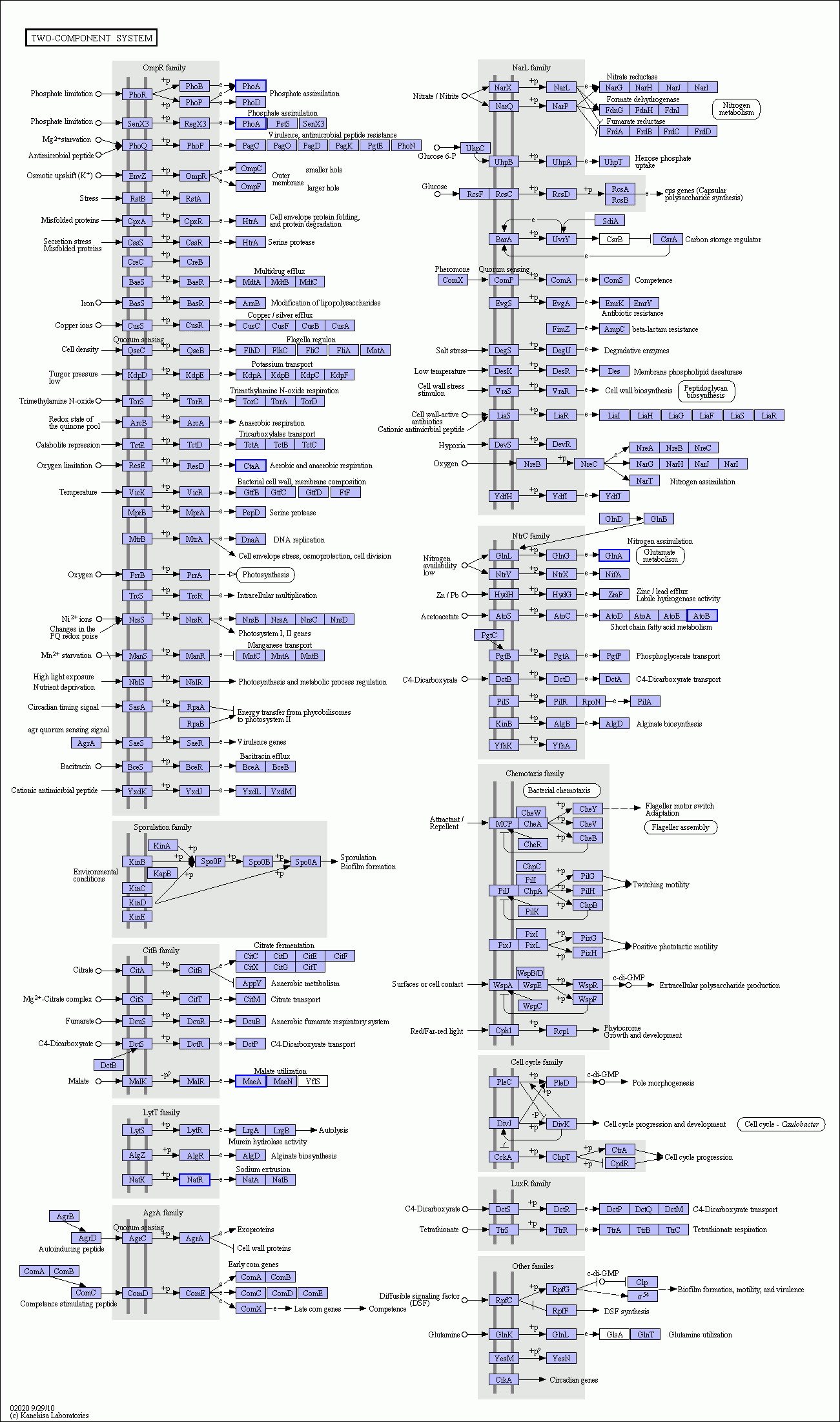

Supplement: Table S4 — KEGG Classification of the unigenes. (ZIP) [file pone.0079516.s004.zip › Kegg/Pathway_Map/ko02020.png]

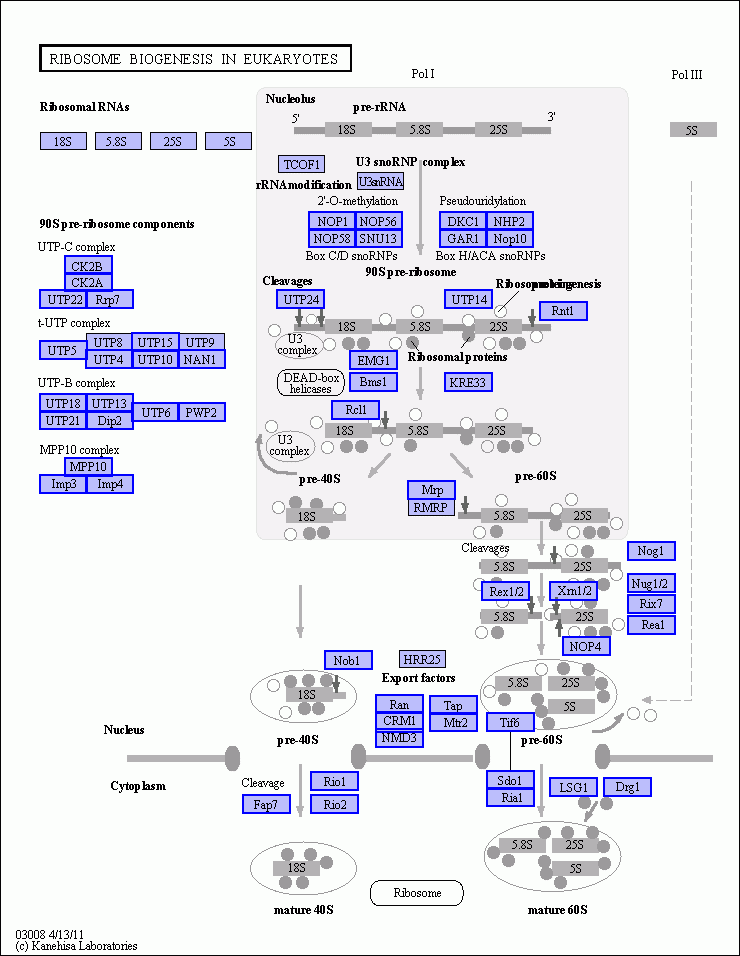

Supplement: Table S4 — KEGG Classification of the unigenes. (ZIP) [file pone.0079516.s004.zip › Kegg/Pathway_Map/ko03008.png]

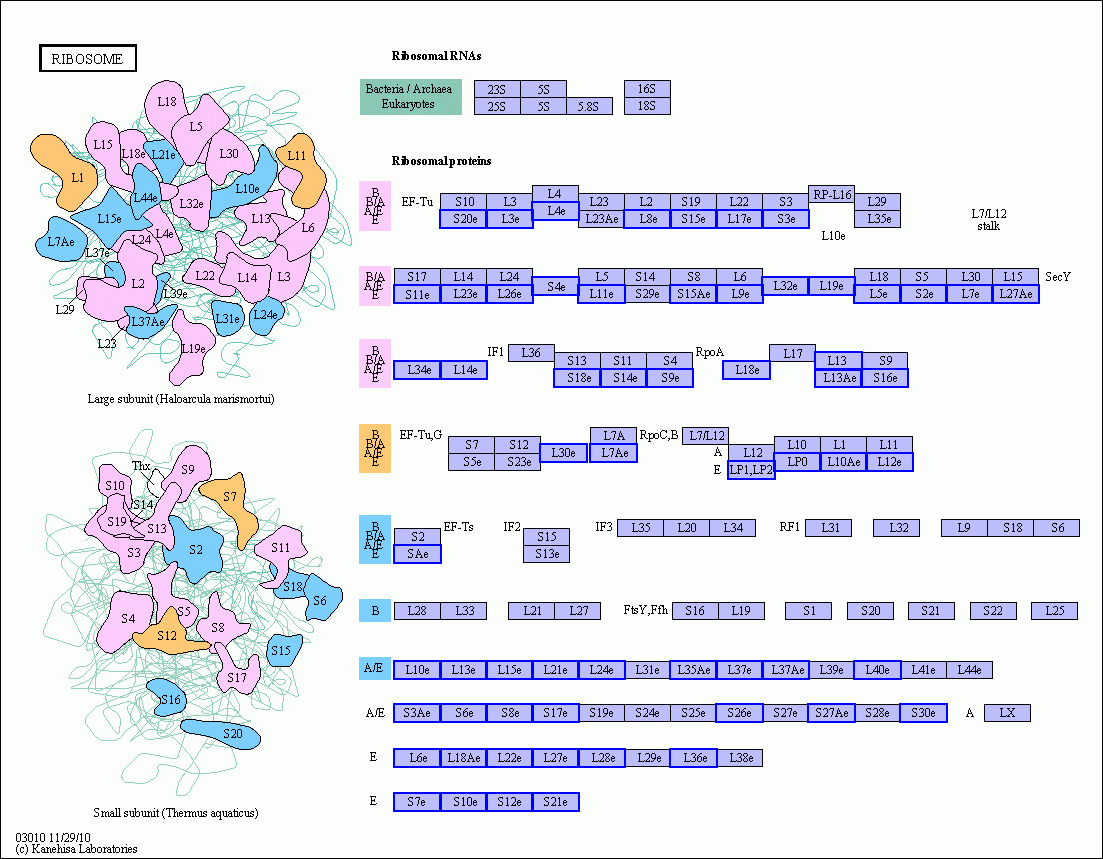

Supplement: Table S4 — KEGG Classification of the unigenes. (ZIP) [file pone.0079516.s004.zip › Kegg/Pathway_Map/ko03010.png]

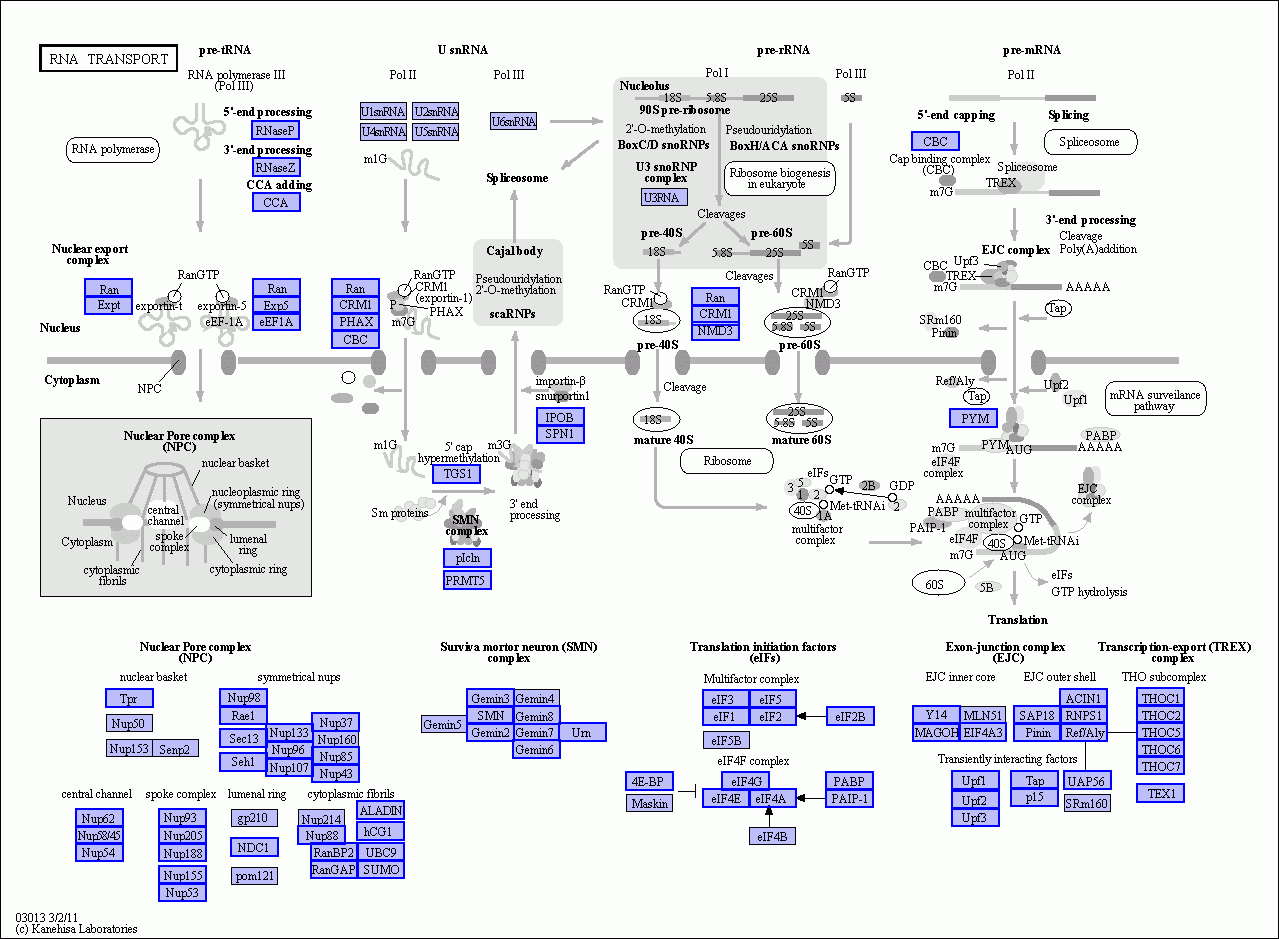

Supplement: Table S4 — KEGG Classification of the unigenes. (ZIP) [file pone.0079516.s004.zip › Kegg/Pathway_Map/ko03013.png]

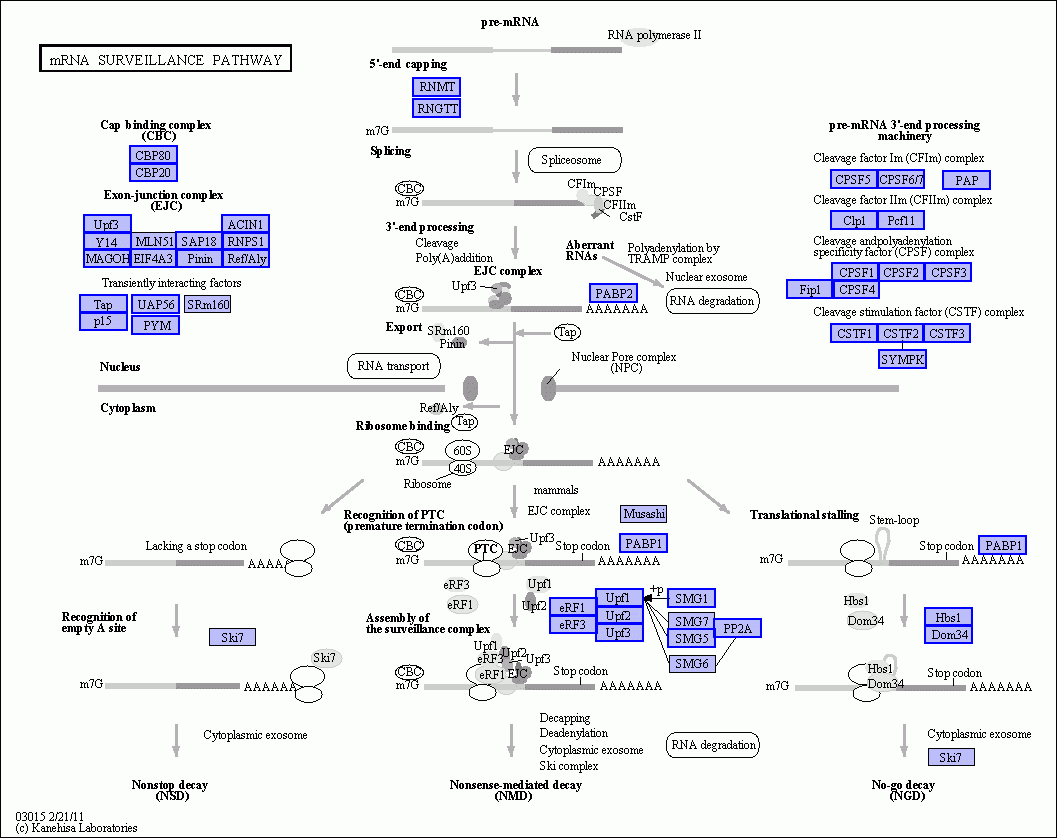

Supplement: Table S4 — KEGG Classification of the unigenes. (ZIP) [file pone.0079516.s004.zip › Kegg/Pathway_Map/ko03015.png]

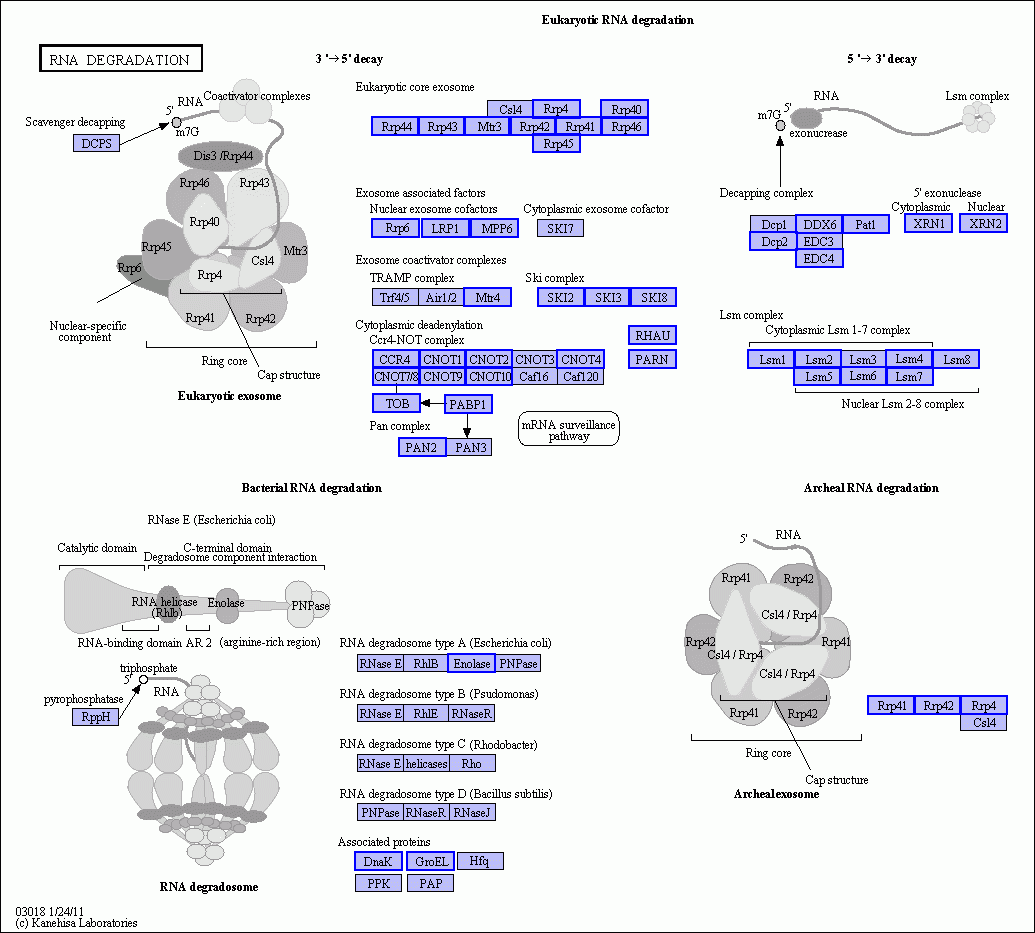

Supplement: Table S4 — KEGG Classification of the unigenes. (ZIP) [file pone.0079516.s004.zip › Kegg/Pathway_Map/ko03018.png]

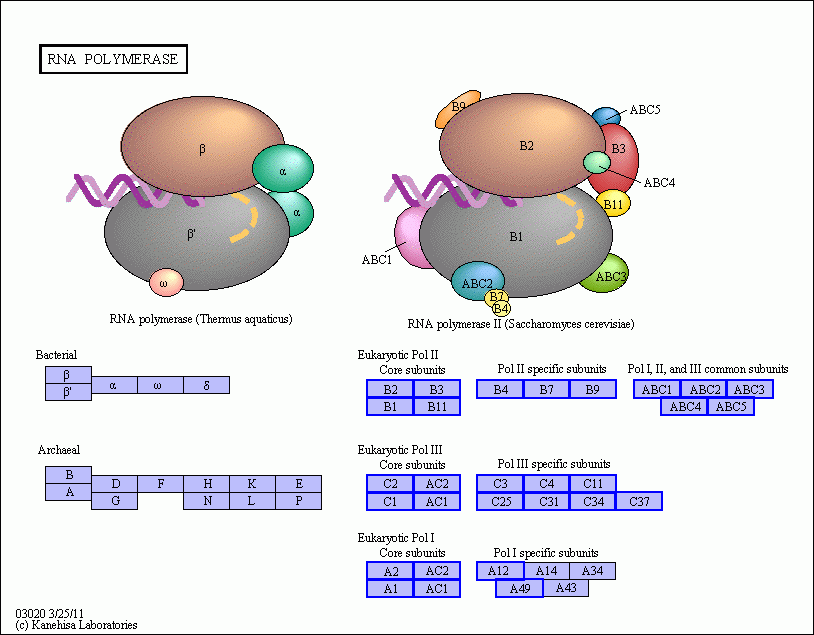

Supplement: Table S4 — KEGG Classification of the unigenes. (ZIP) [file pone.0079516.s004.zip › Kegg/Pathway_Map/ko03020.png]

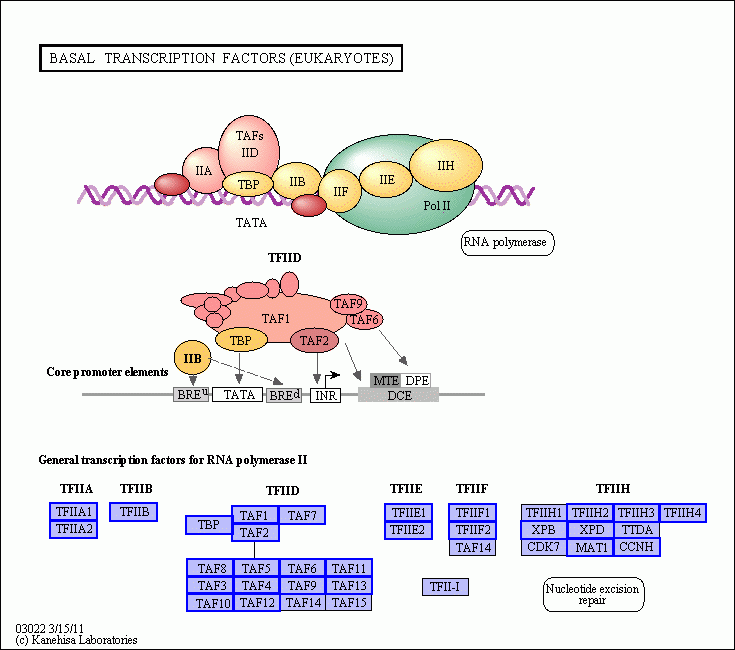

Supplement: Table S4 — KEGG Classification of the unigenes. (ZIP) [file pone.0079516.s004.zip › Kegg/Pathway_Map/ko03022.png]

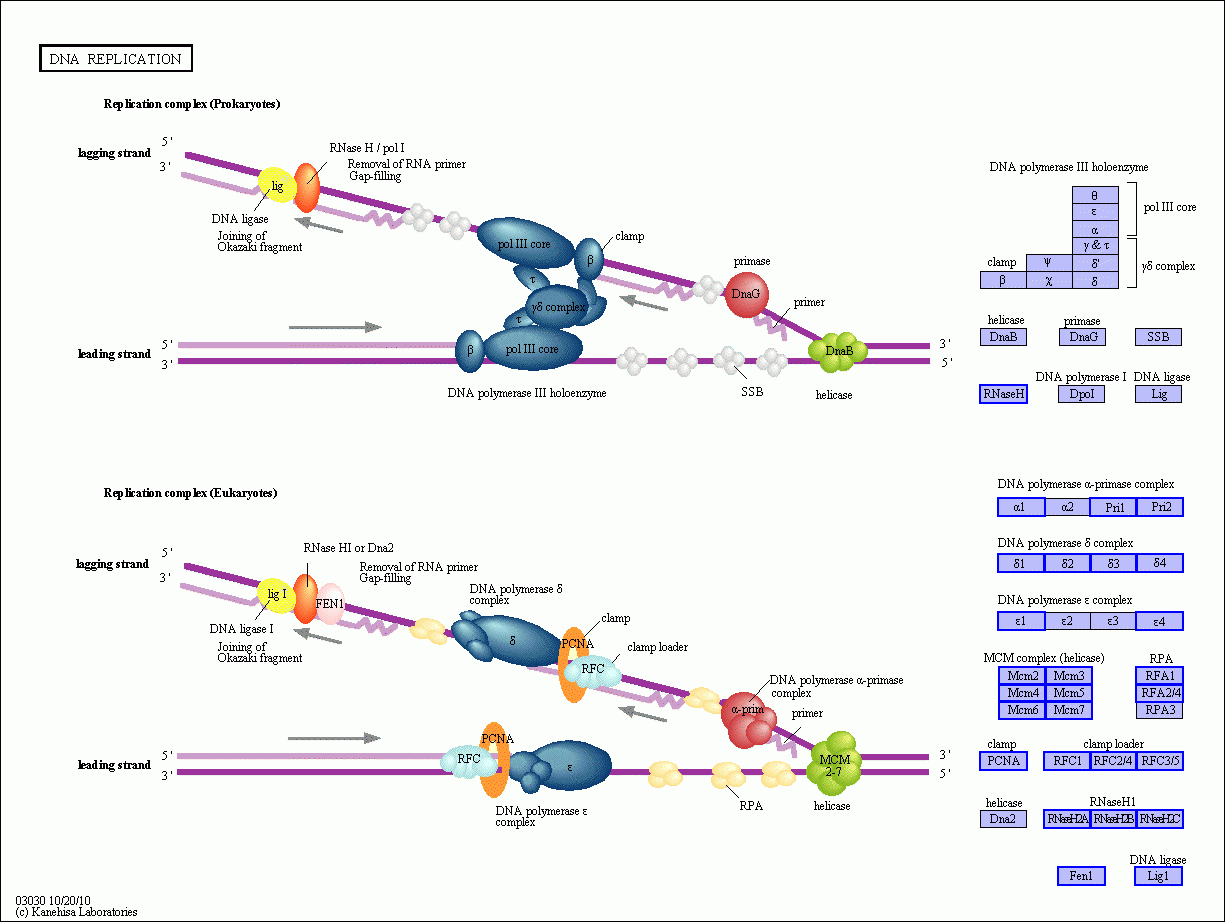

Supplement: Table S4 — KEGG Classification of the unigenes. (ZIP) [file pone.0079516.s004.zip › Kegg/Pathway_Map/ko03030.png]

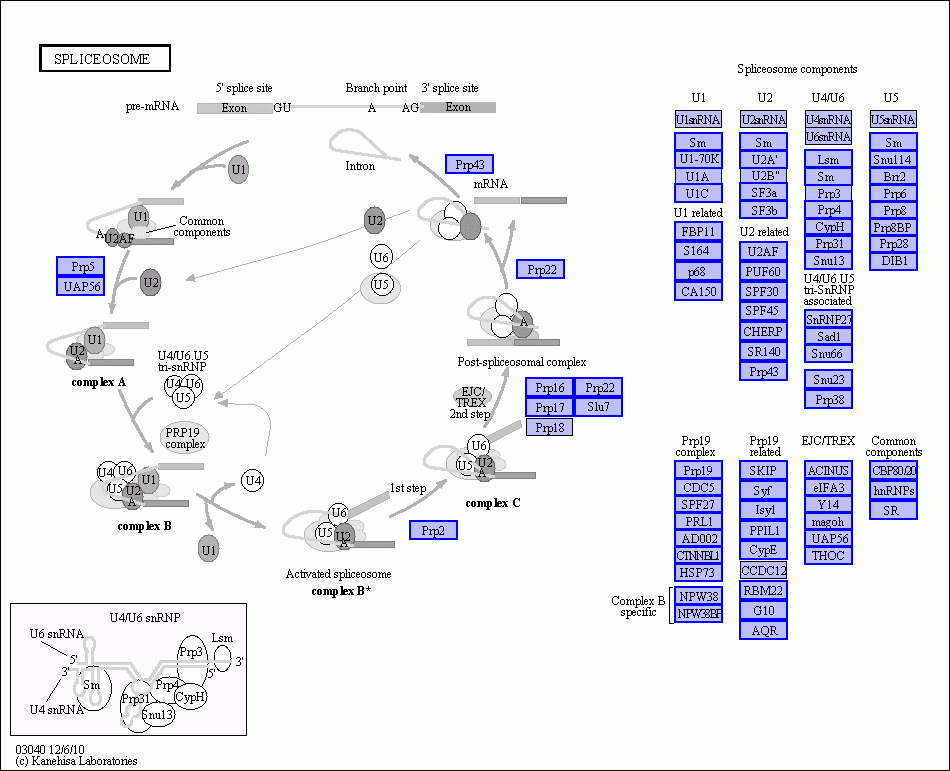

Supplement: Table S4 — KEGG Classification of the unigenes. (ZIP) [file pone.0079516.s004.zip › Kegg/Pathway_Map/ko03040.png]

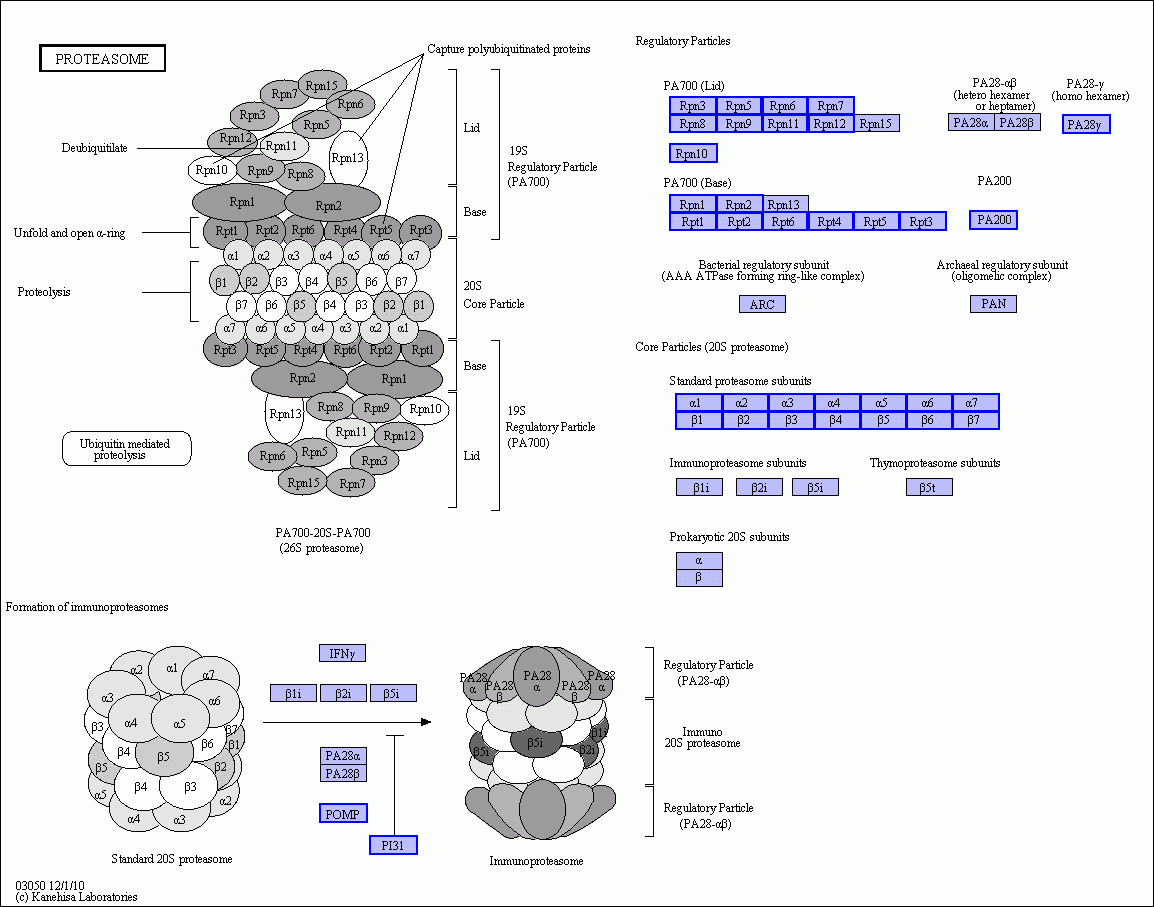

Supplement: Table S4 — KEGG Classification of the unigenes. (ZIP) [file pone.0079516.s004.zip › Kegg/Pathway_Map/ko03050.png]

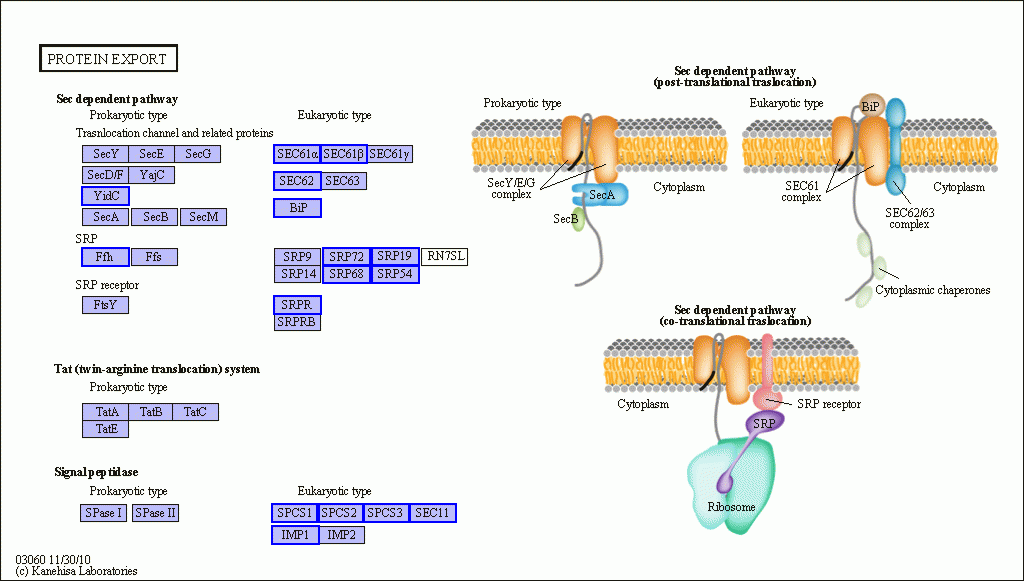

Supplement: Table S4 — KEGG Classification of the unigenes. (ZIP) [file pone.0079516.s004.zip › Kegg/Pathway_Map/ko03060.png]

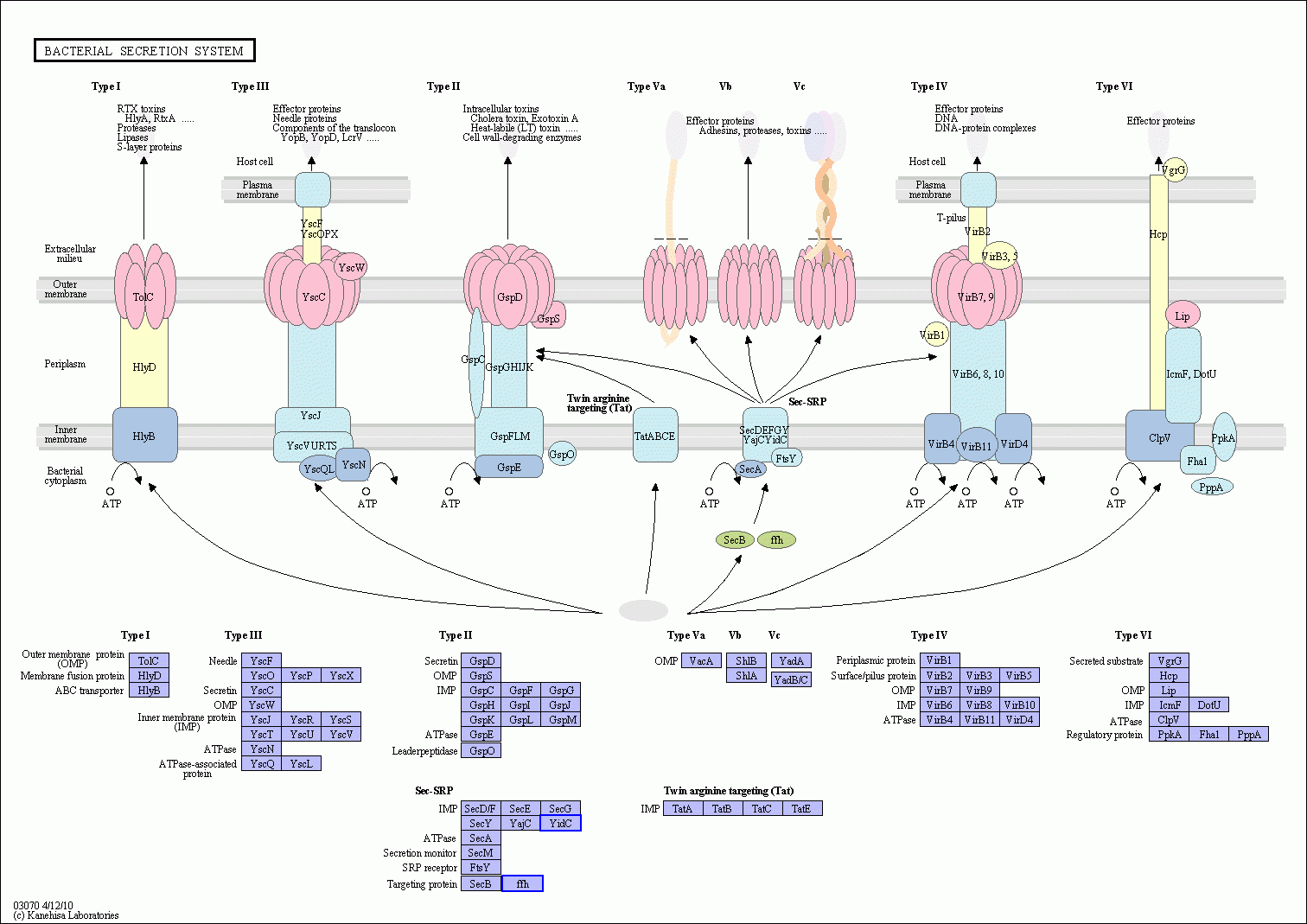

Supplement: Table S4 — KEGG Classification of the unigenes. (ZIP) [file pone.0079516.s004.zip › Kegg/Pathway_Map/ko03070.png]

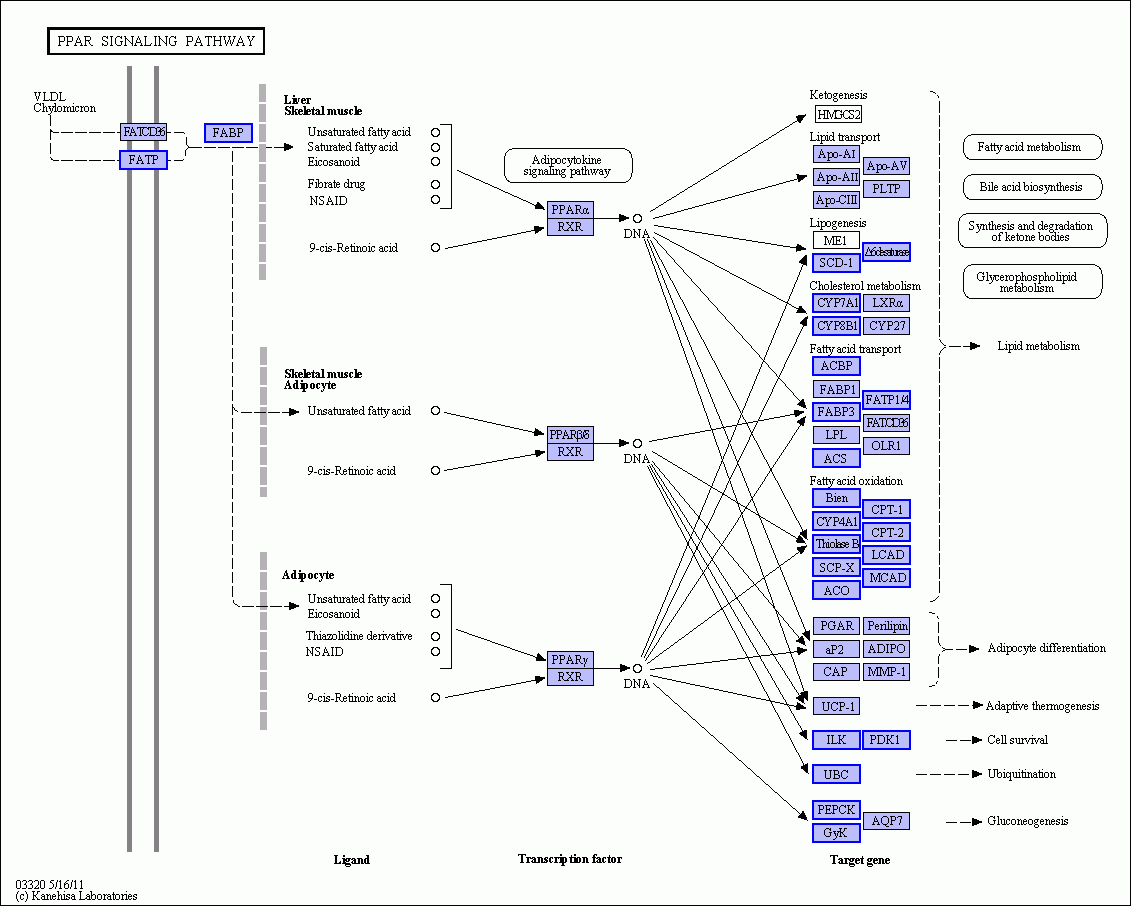

Supplement: Table S4 — KEGG Classification of the unigenes. (ZIP) [file pone.0079516.s004.zip › Kegg/Pathway_Map/ko03320.png]

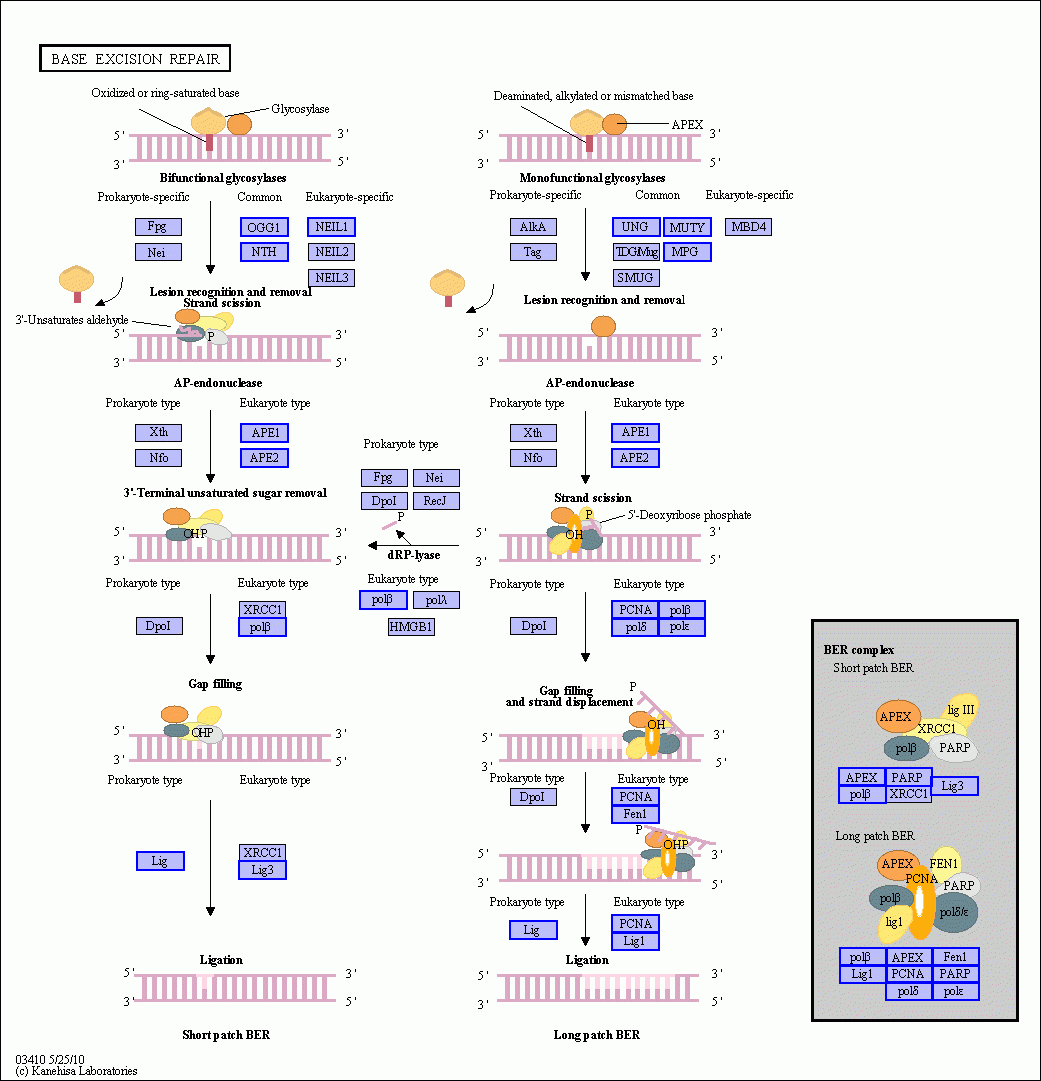

Supplement: Table S4 — KEGG Classification of the unigenes. (ZIP) [file pone.0079516.s004.zip › Kegg/Pathway_Map/ko03410.png]

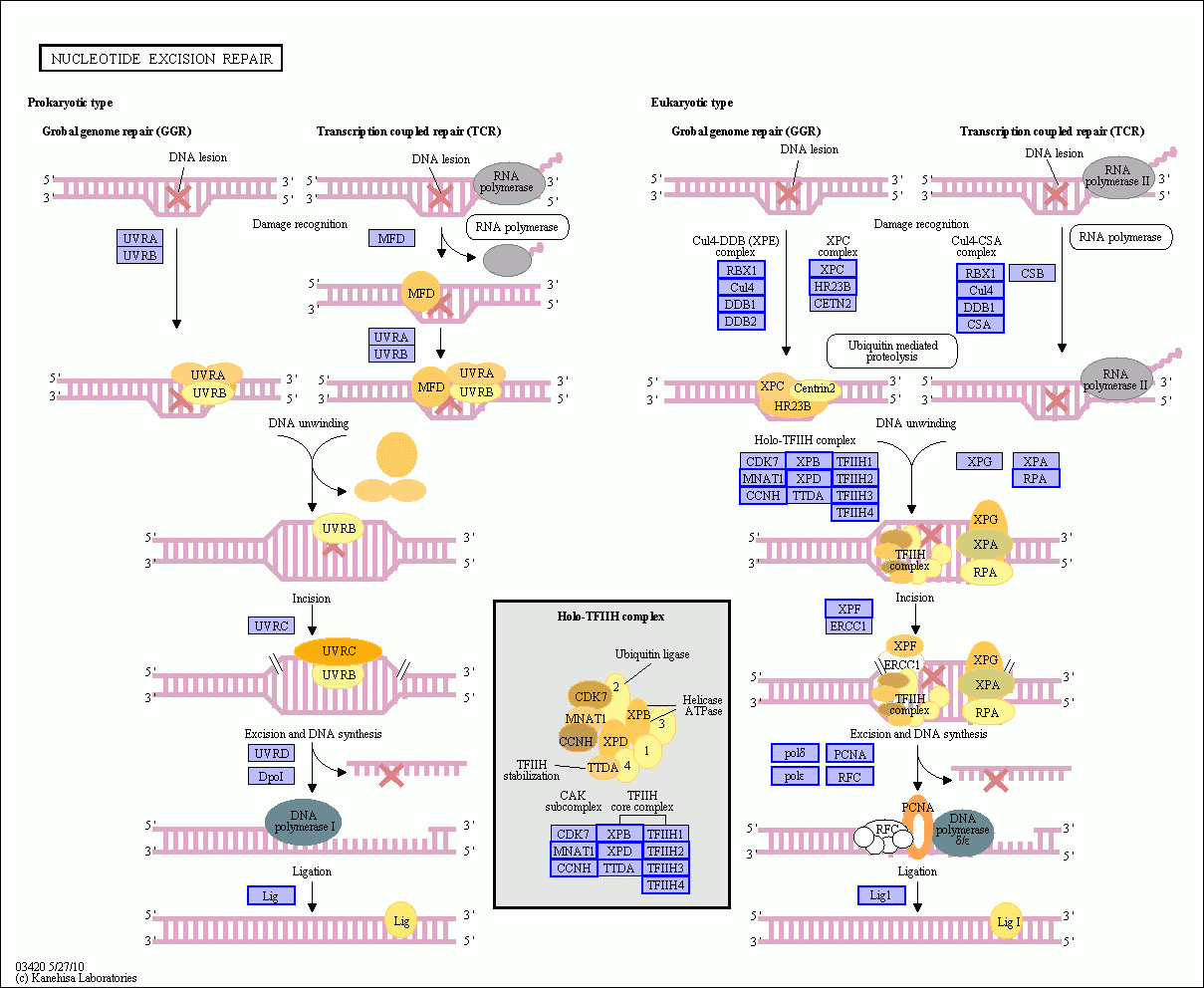

Supplement: Table S4 — KEGG Classification of the unigenes. (ZIP) [file pone.0079516.s004.zip › Kegg/Pathway_Map/ko03420.png]

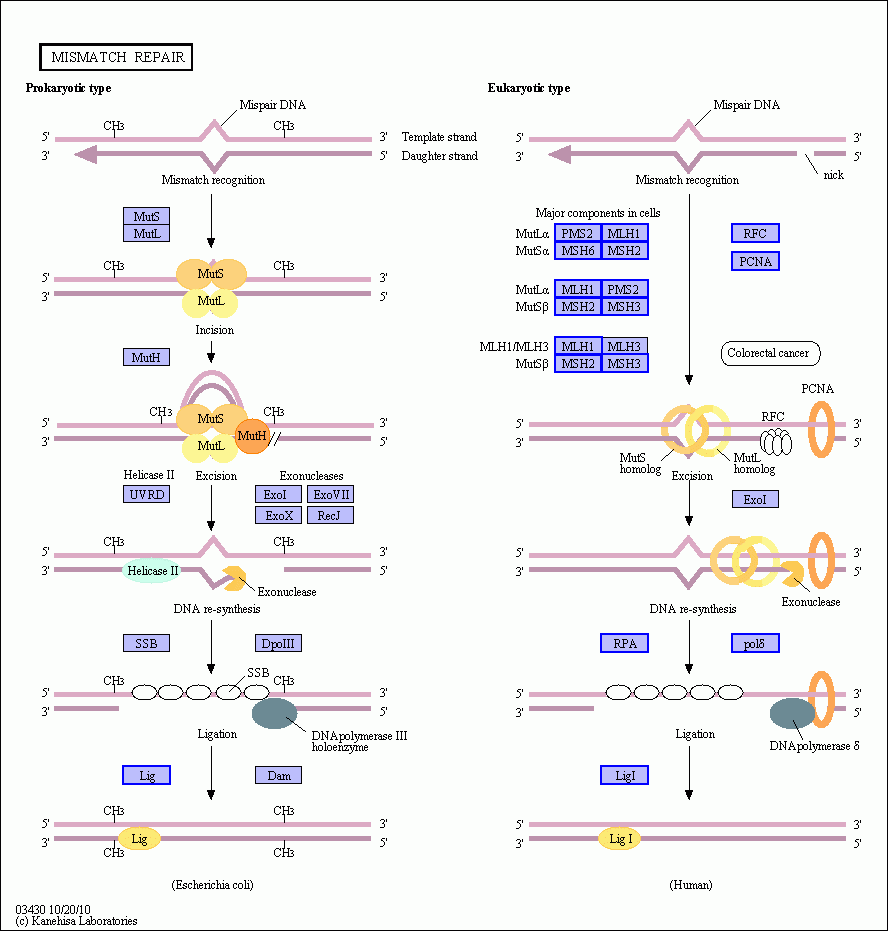

Supplement: Table S4 — KEGG Classification of the unigenes. (ZIP) [file pone.0079516.s004.zip › Kegg/Pathway_Map/ko03430.png]

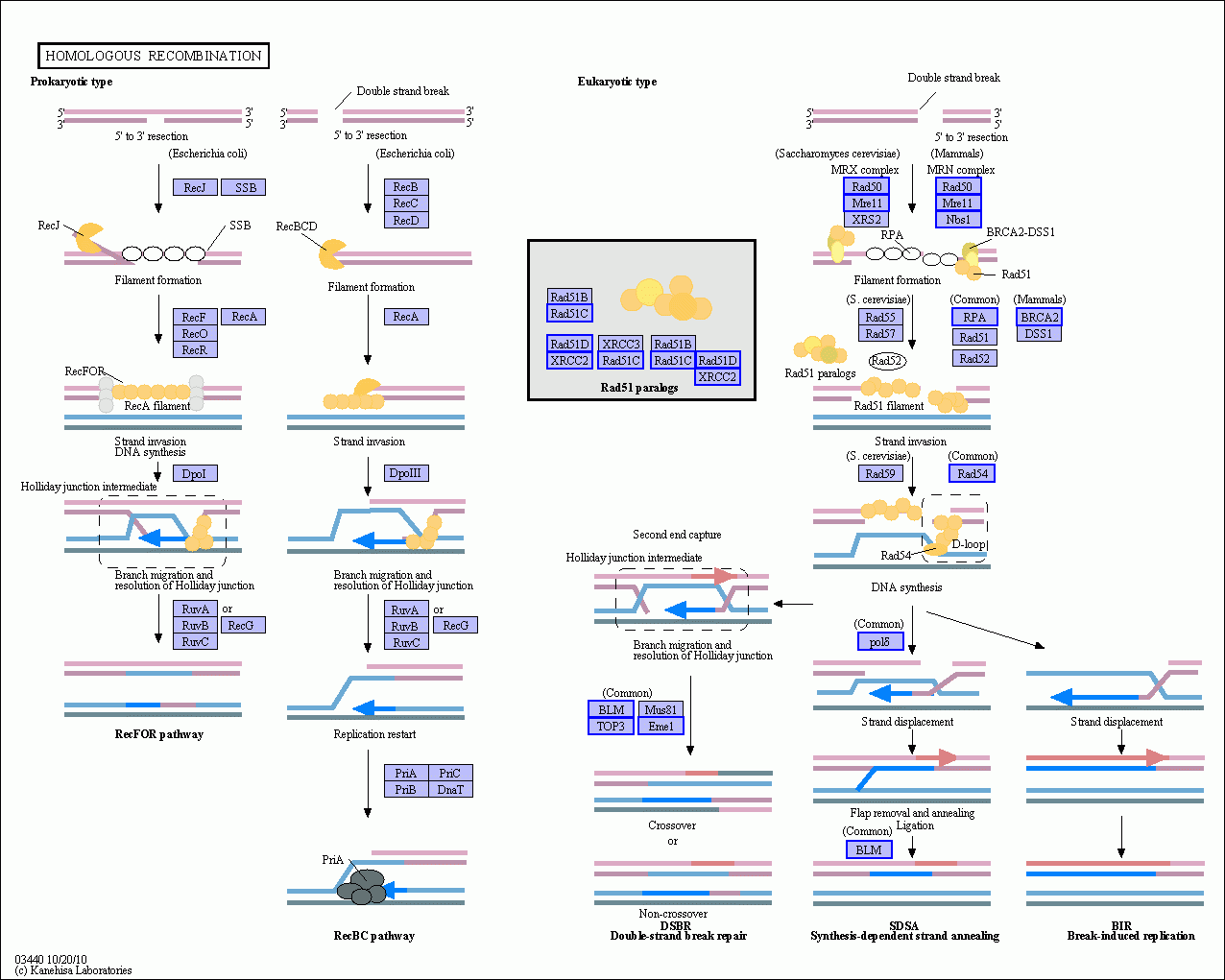

Supplement: Table S4 — KEGG Classification of the unigenes. (ZIP) [file pone.0079516.s004.zip › Kegg/Pathway_Map/ko03440.png]

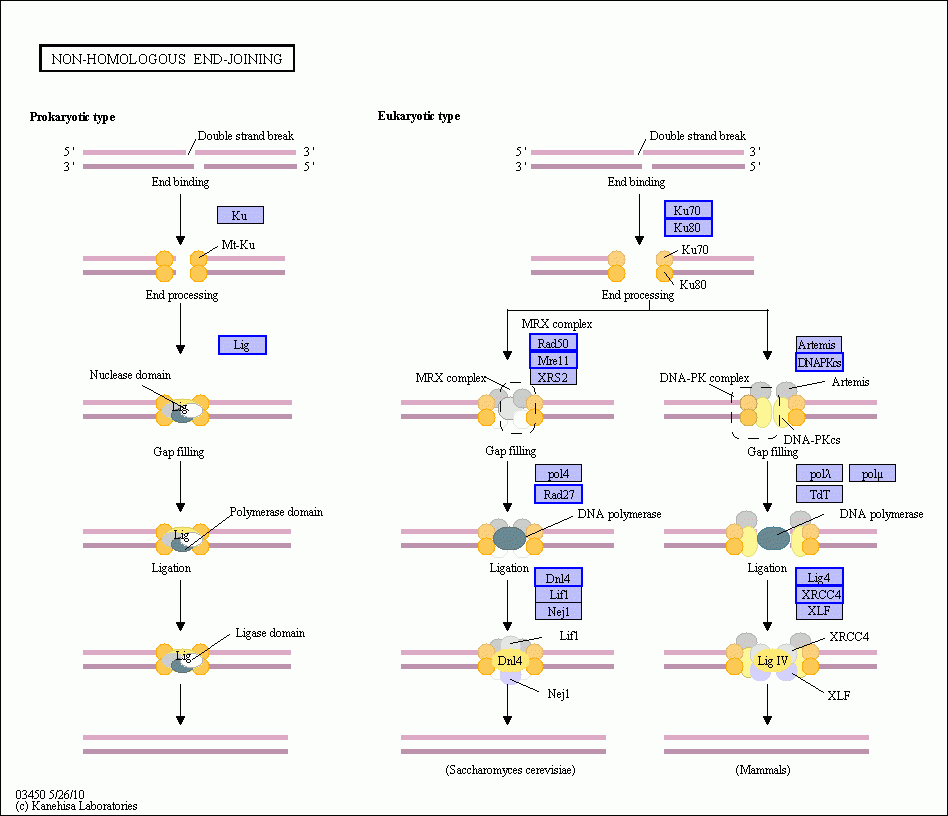

Supplement: Table S4 — KEGG Classification of the unigenes. (ZIP) [file pone.0079516.s004.zip › Kegg/Pathway_Map/ko03450.png]

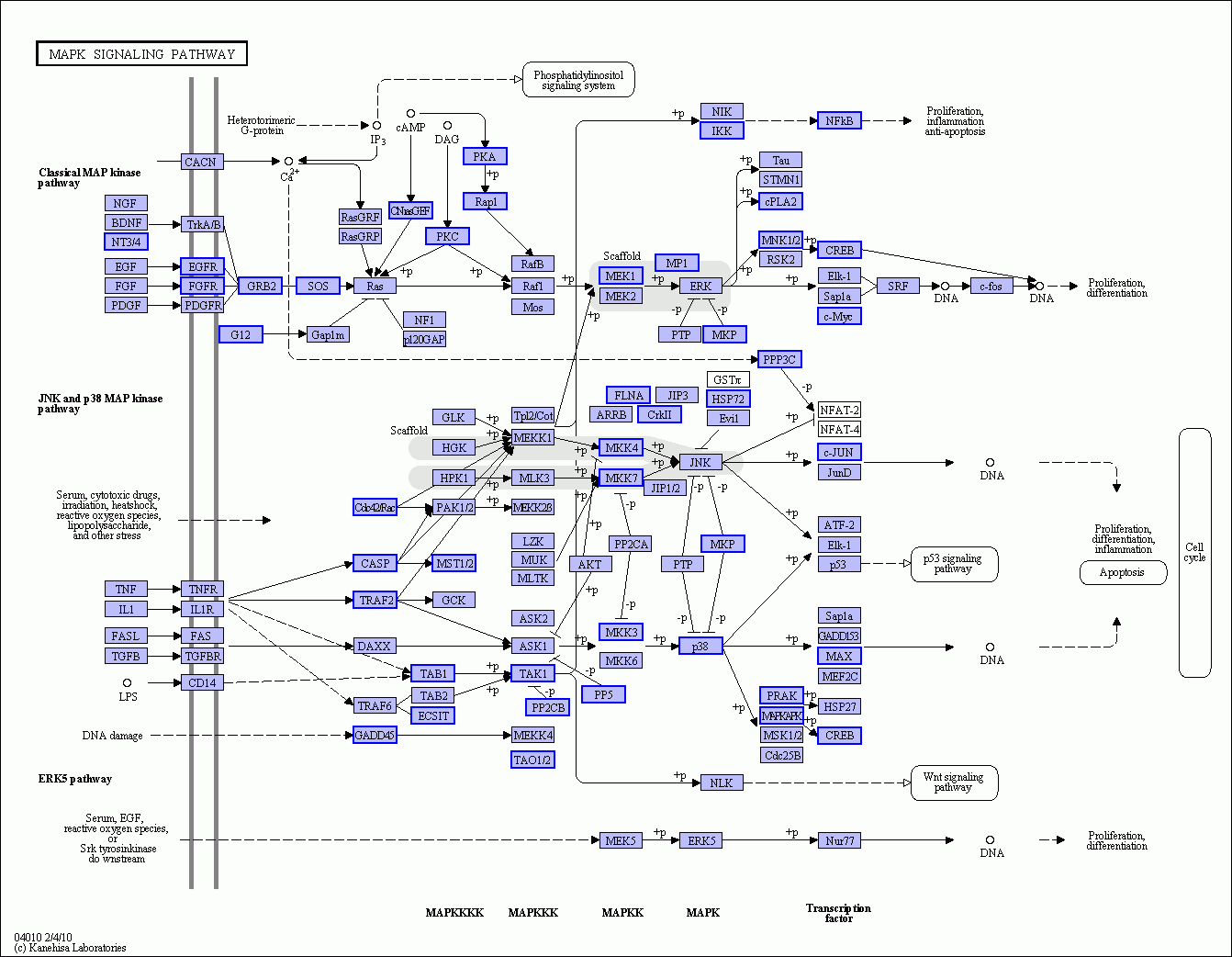

Supplement: Table S4 — KEGG Classification of the unigenes. (ZIP) [file pone.0079516.s004.zip › Kegg/Pathway_Map/ko04010.png]

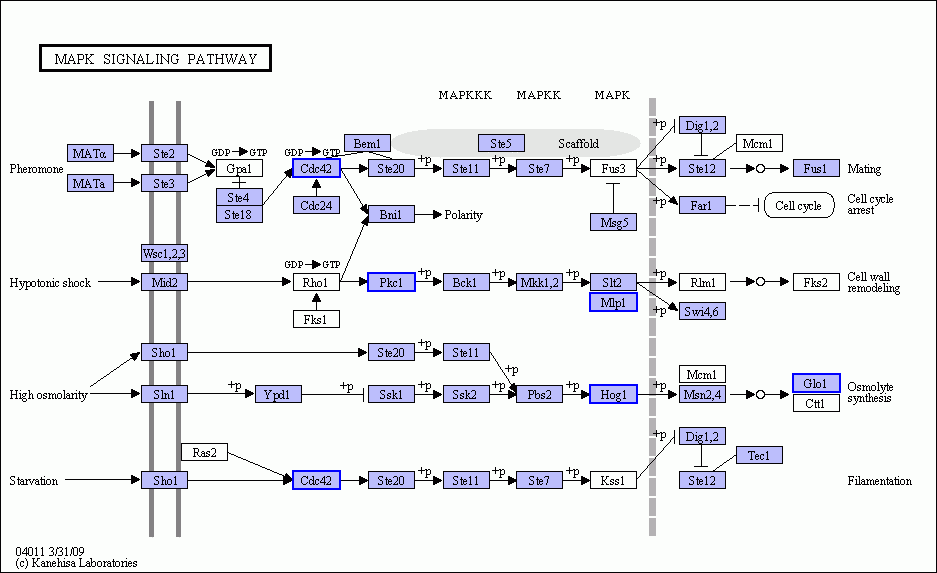

Supplement: Table S4 — KEGG Classification of the unigenes. (ZIP) [file pone.0079516.s004.zip › Kegg/Pathway_Map/ko04011.png]

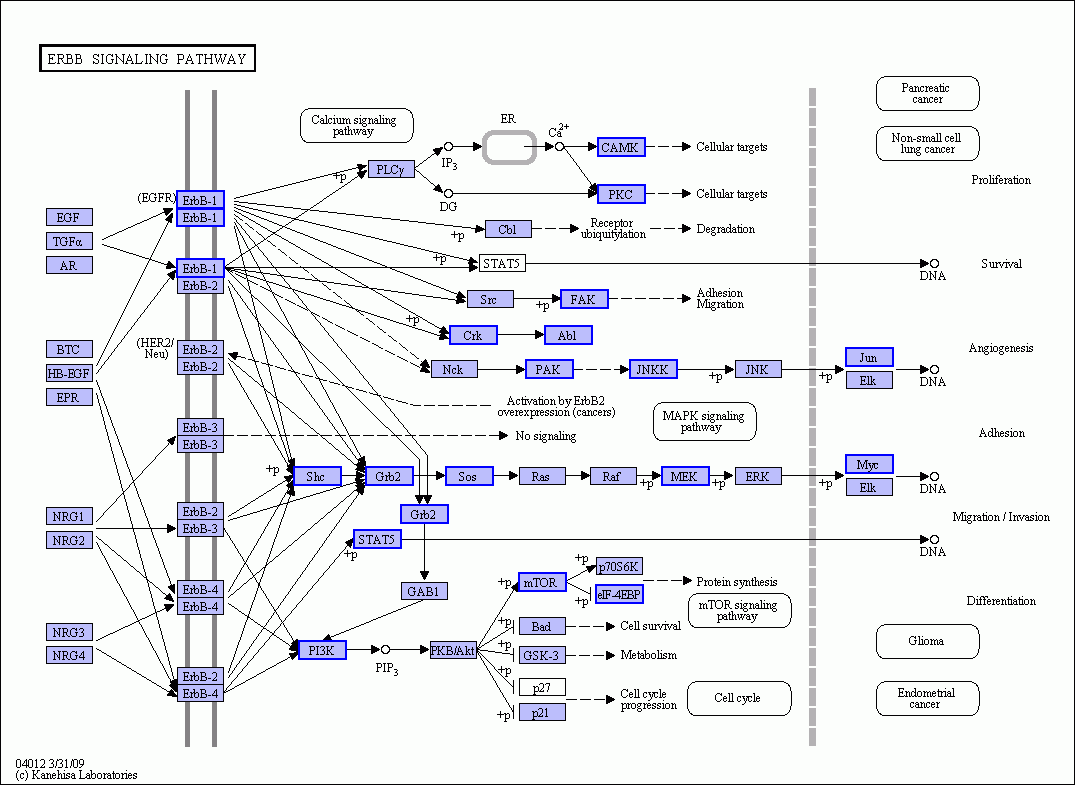

Supplement: Table S4 — KEGG Classification of the unigenes. (ZIP) [file pone.0079516.s004.zip › Kegg/Pathway_Map/ko04012.png]

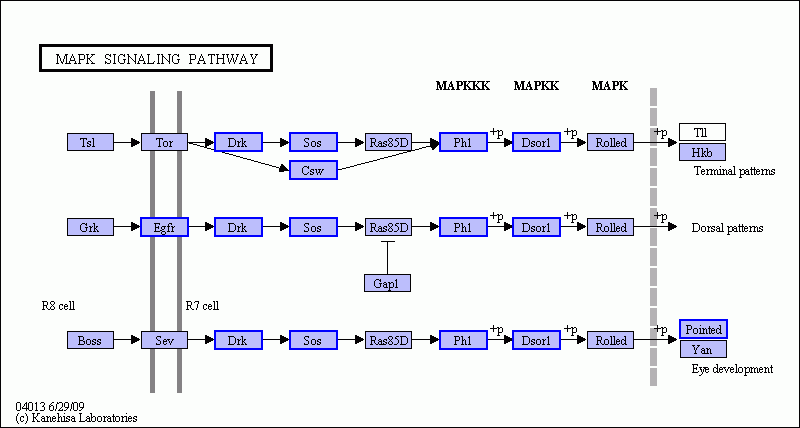

Supplement: Table S4 — KEGG Classification of the unigenes. (ZIP) [file pone.0079516.s004.zip › Kegg/Pathway_Map/ko04013.png]

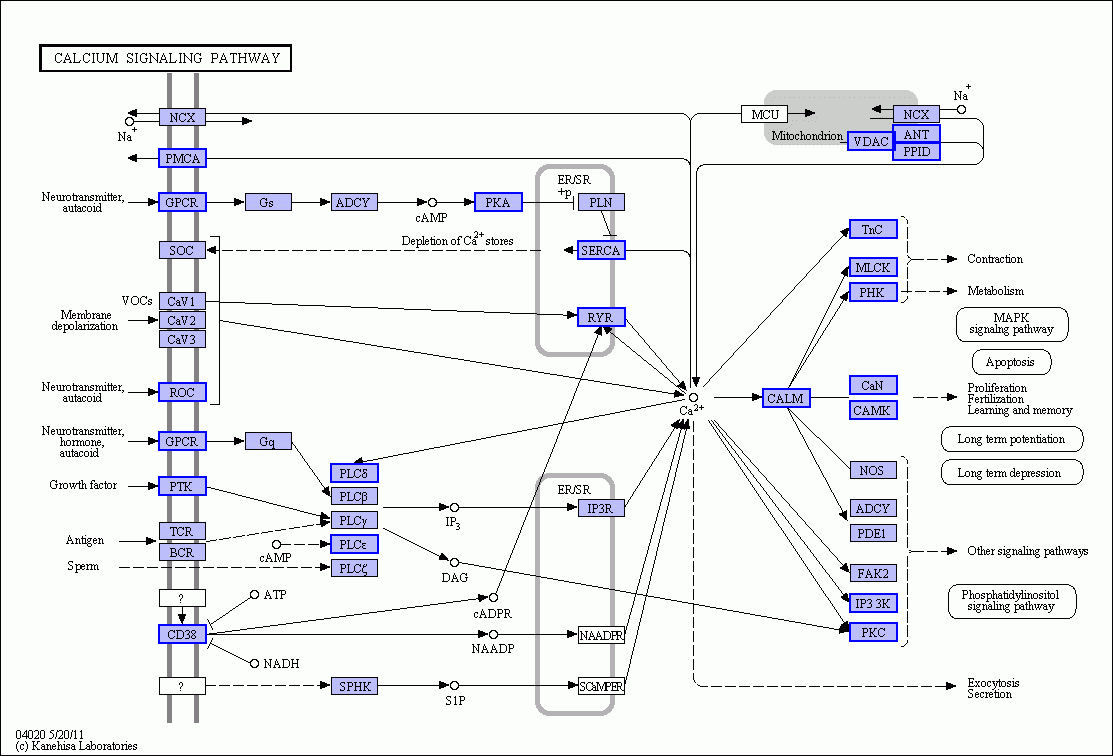

Supplement: Table S4 — KEGG Classification of the unigenes. (ZIP) [file pone.0079516.s004.zip › Kegg/Pathway_Map/ko04020.png]

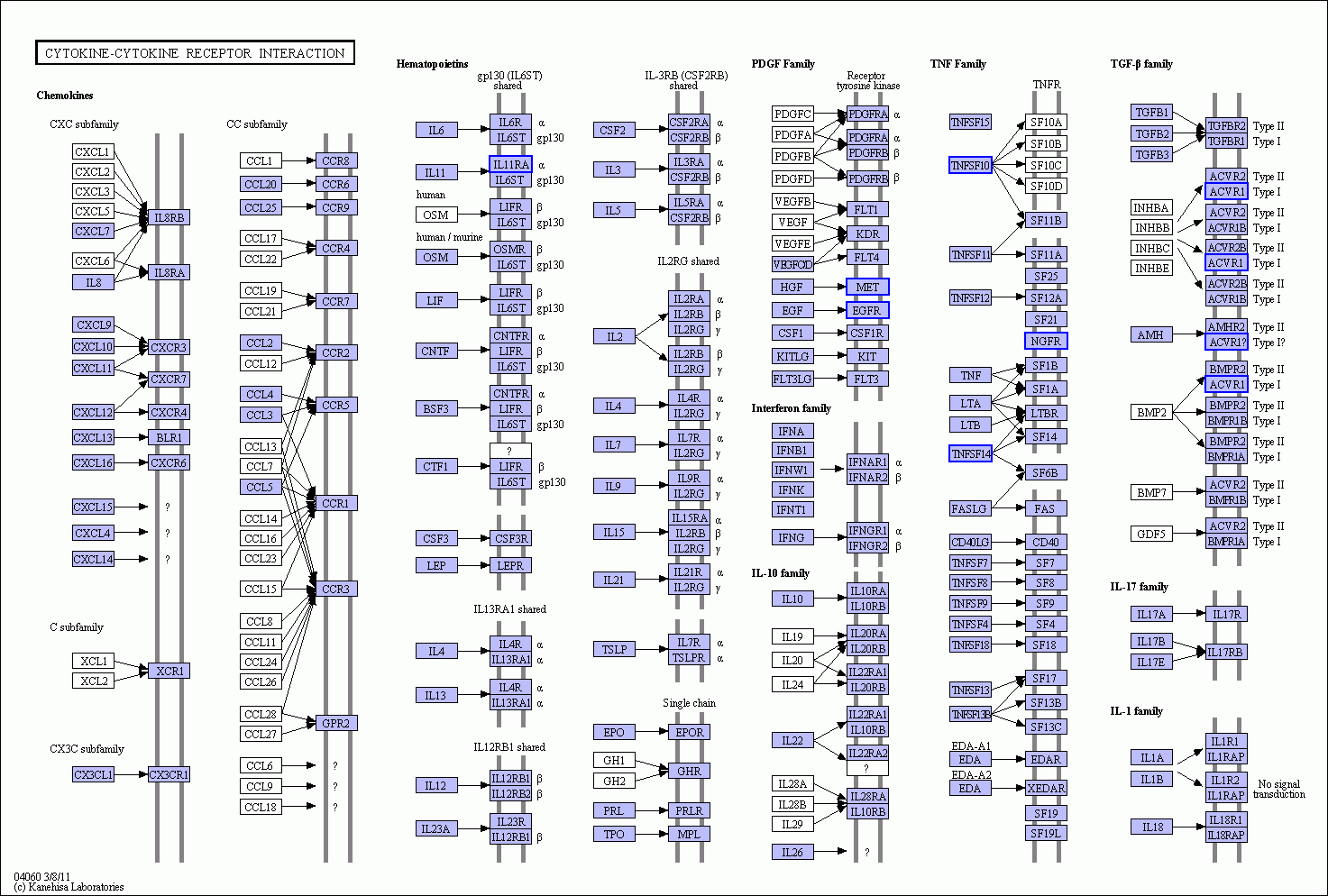

Supplement: Table S4 — KEGG Classification of the unigenes. (ZIP) [file pone.0079516.s004.zip › Kegg/Pathway_Map/ko04060.png]

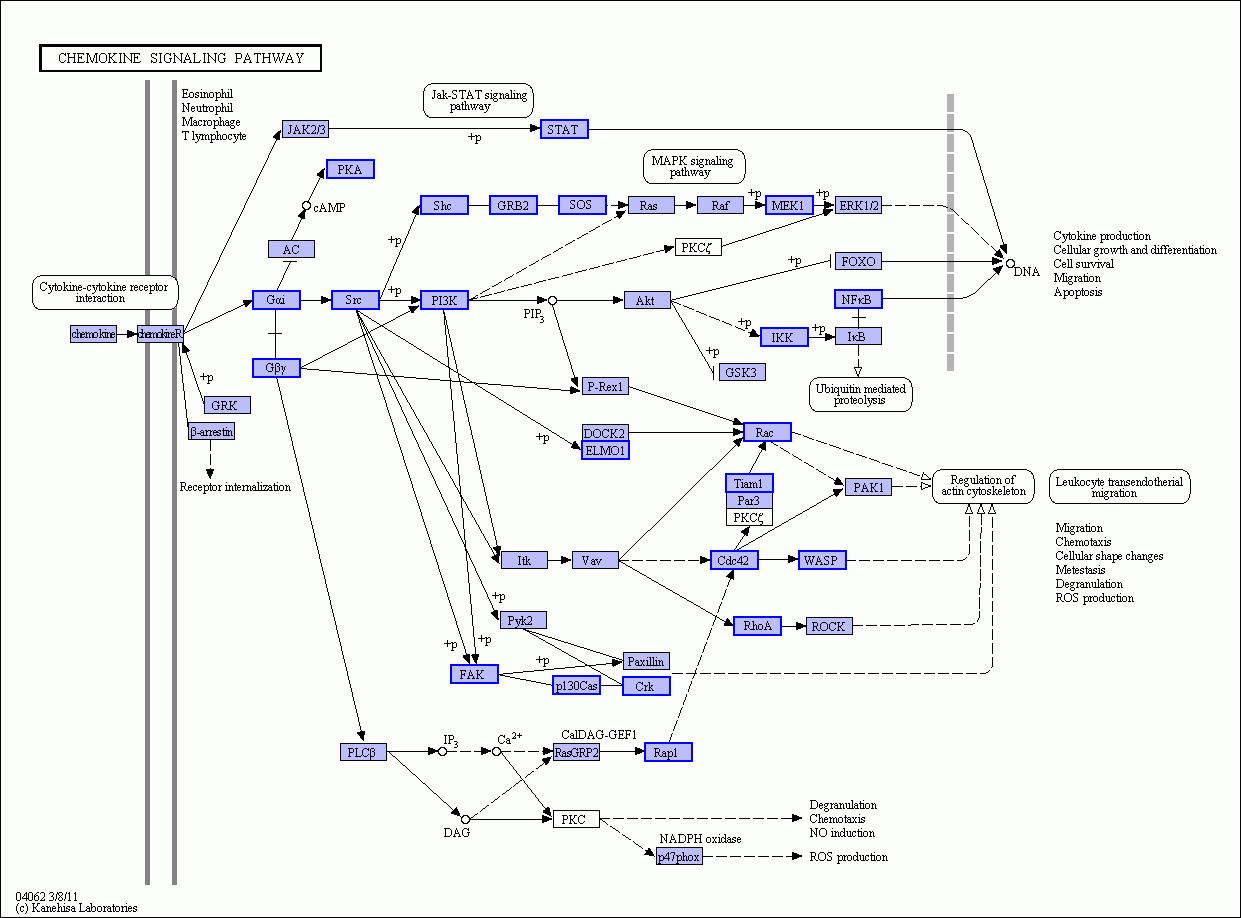

Supplement: Table S4 — KEGG Classification of the unigenes. (ZIP) [file pone.0079516.s004.zip › Kegg/Pathway_Map/ko04062.png]

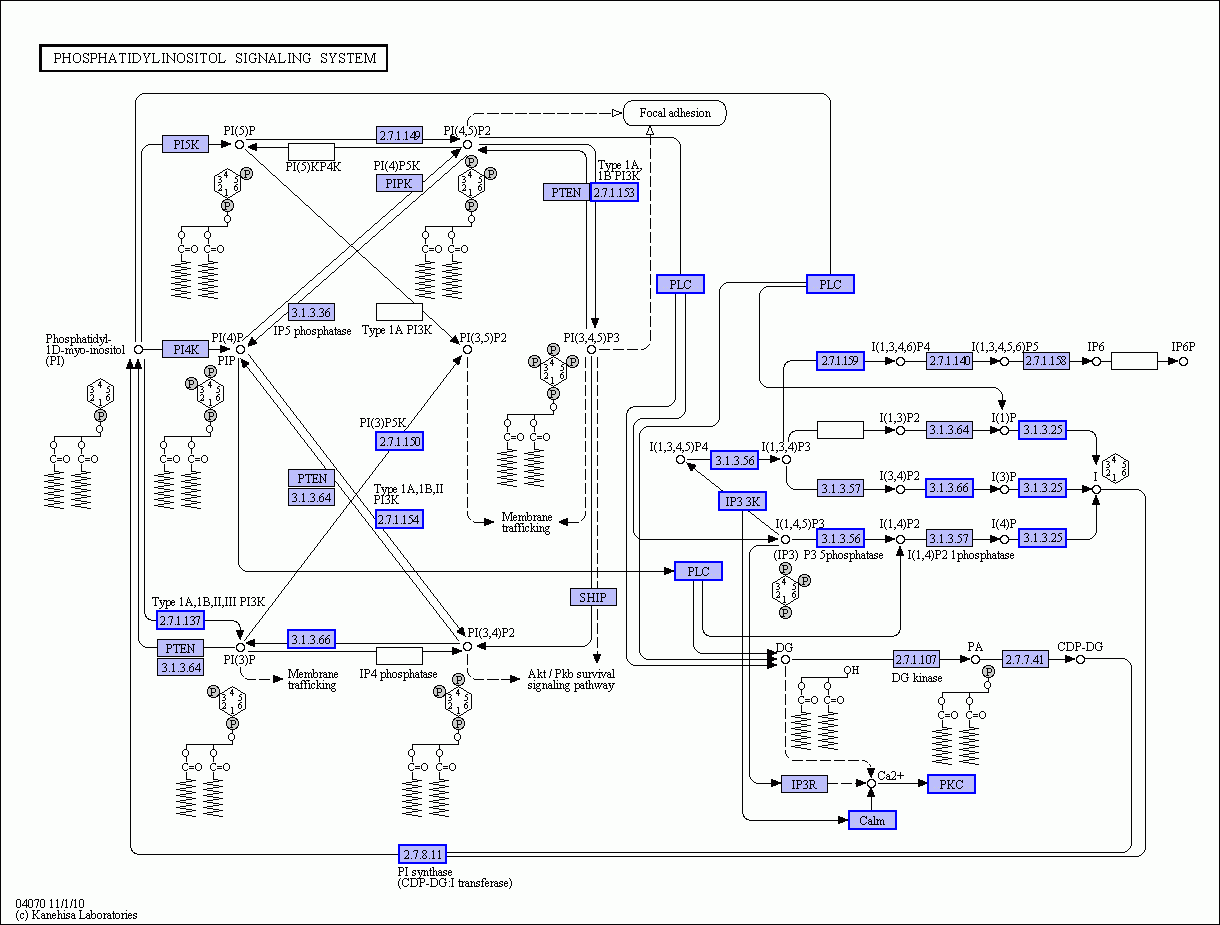

Supplement: Table S4 — KEGG Classification of the unigenes. (ZIP) [file pone.0079516.s004.zip › Kegg/Pathway_Map/ko04070.png]

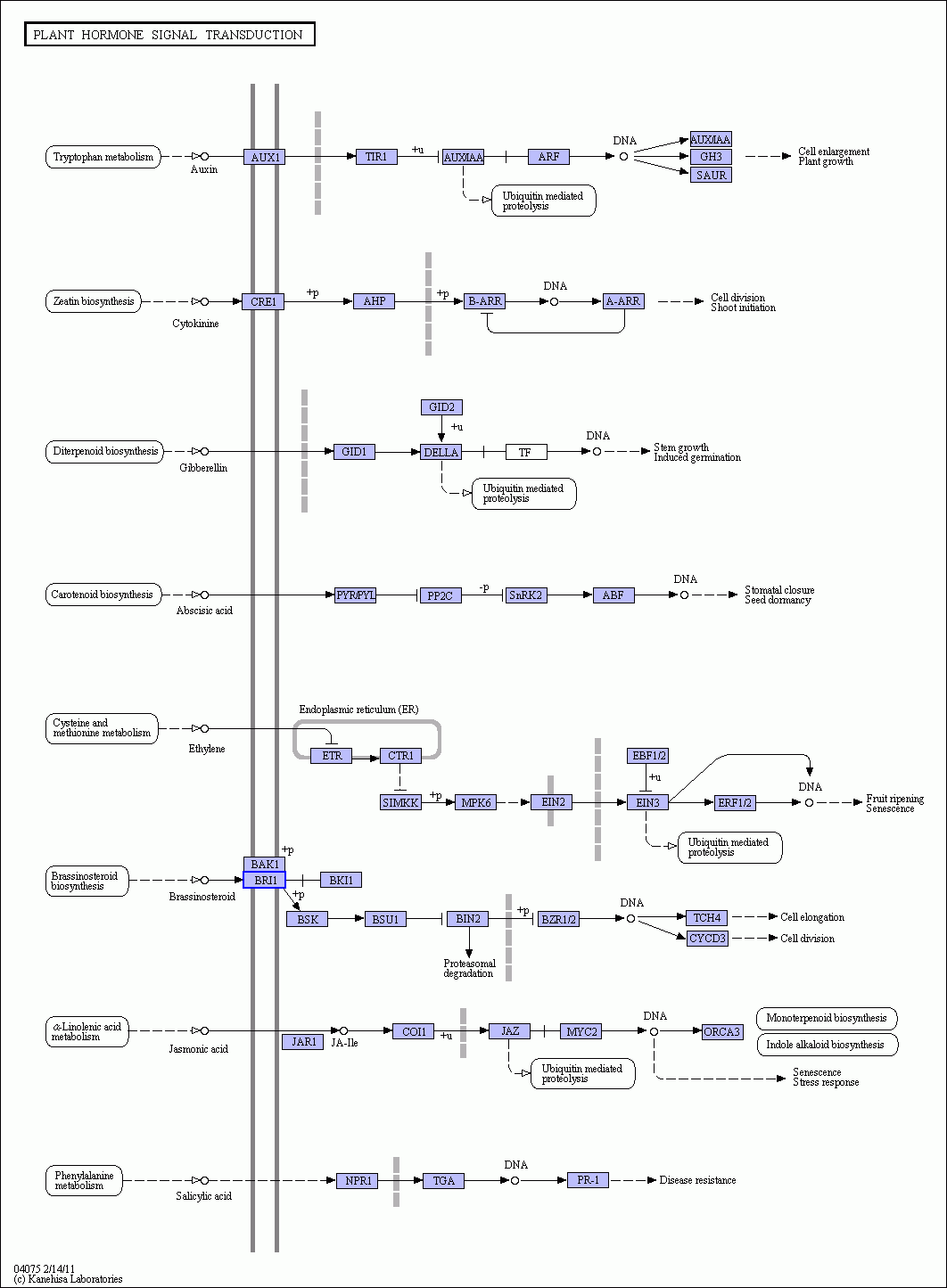

Supplement: Table S4 — KEGG Classification of the unigenes. (ZIP) [file pone.0079516.s004.zip › Kegg/Pathway_Map/ko04075.png]

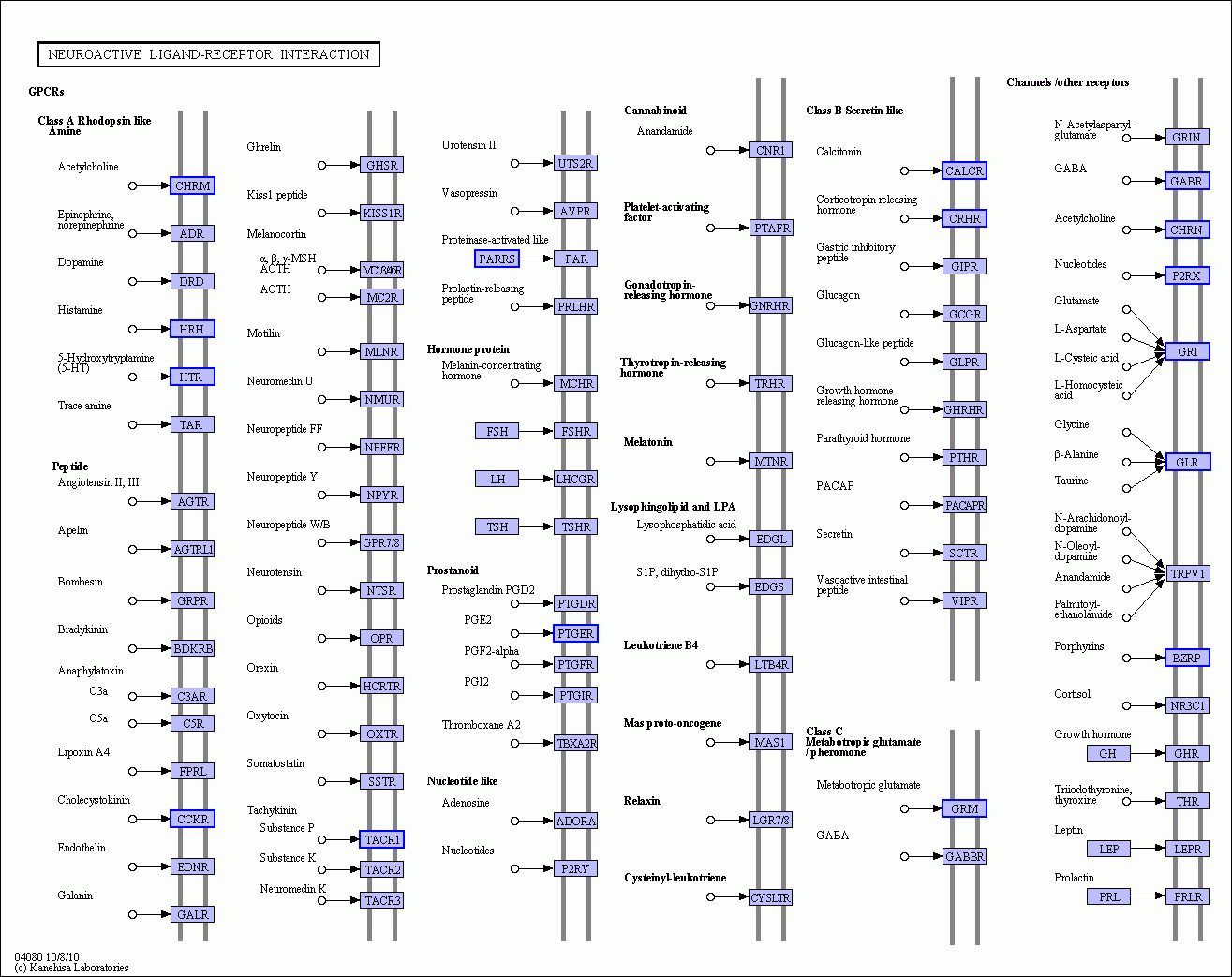

Supplement: Table S4 — KEGG Classification of the unigenes. (ZIP) [file pone.0079516.s004.zip › Kegg/Pathway_Map/ko04080.png]

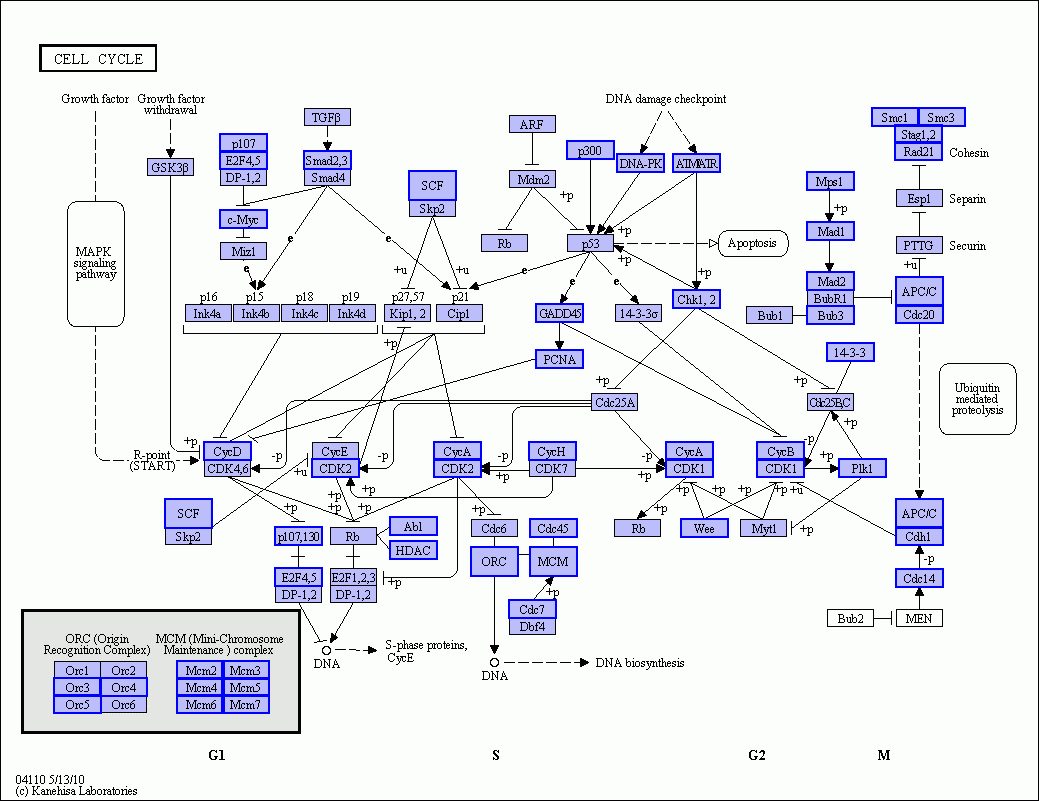

Supplement: Table S4 — KEGG Classification of the unigenes. (ZIP) [file pone.0079516.s004.zip › Kegg/Pathway_Map/ko04110.png]

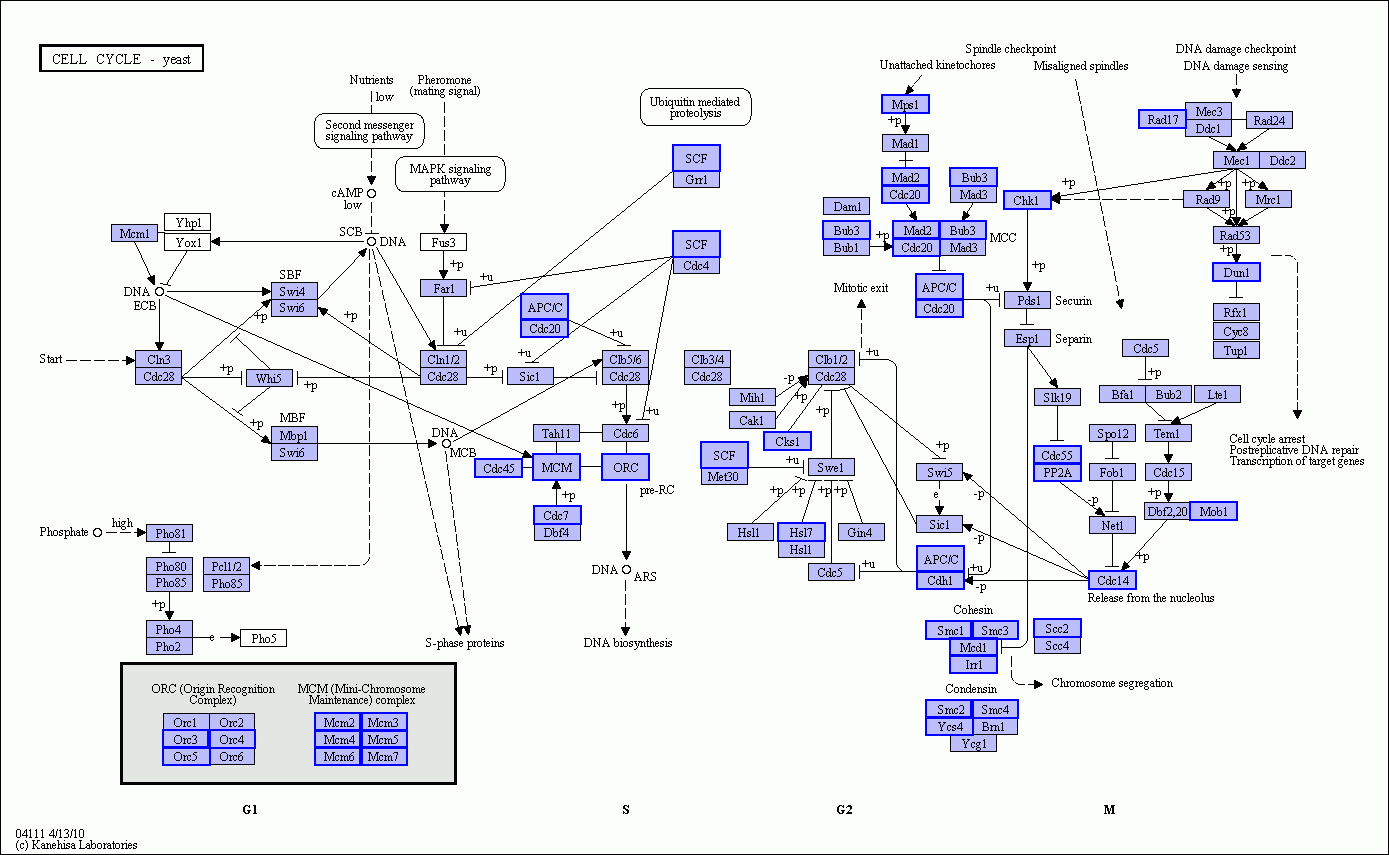

Supplement: Table S4 — KEGG Classification of the unigenes. (ZIP) [file pone.0079516.s004.zip › Kegg/Pathway_Map/ko04111.png]

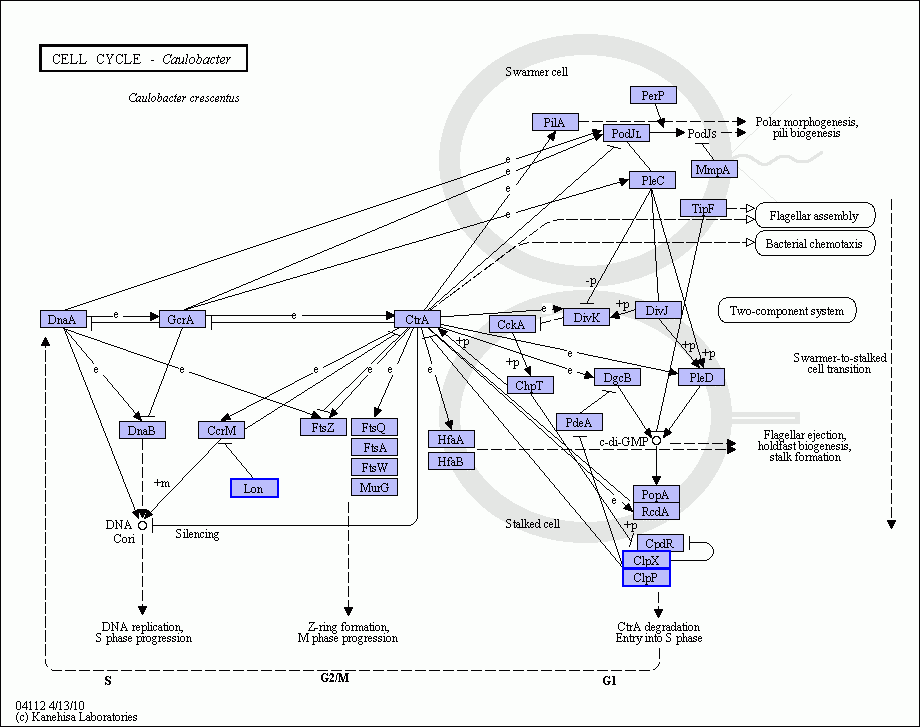

Supplement: Table S4 — KEGG Classification of the unigenes. (ZIP) [file pone.0079516.s004.zip › Kegg/Pathway_Map/ko04112.png]

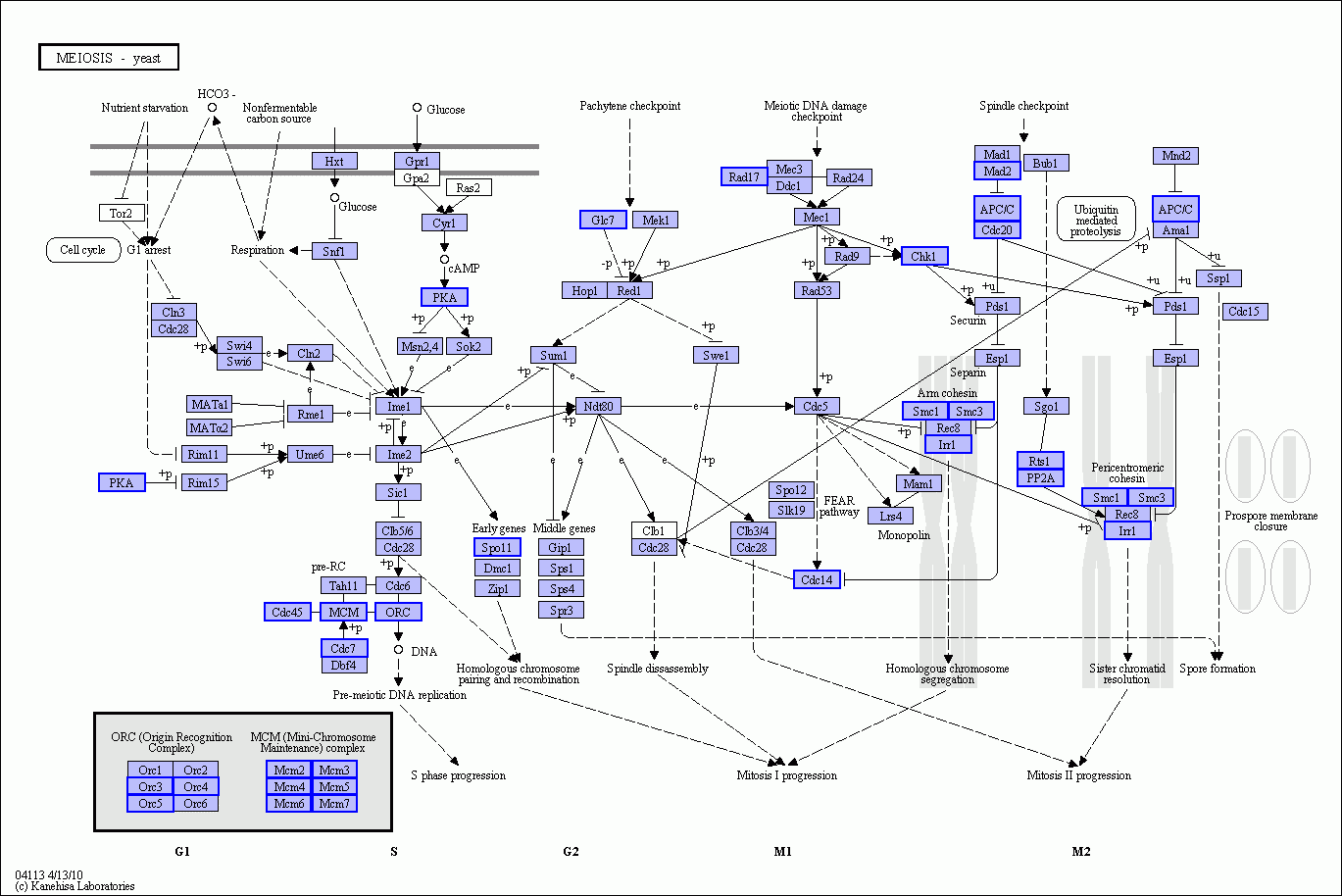

Supplement: Table S4 — KEGG Classification of the unigenes. (ZIP) [file pone.0079516.s004.zip › Kegg/Pathway_Map/ko04113.png]

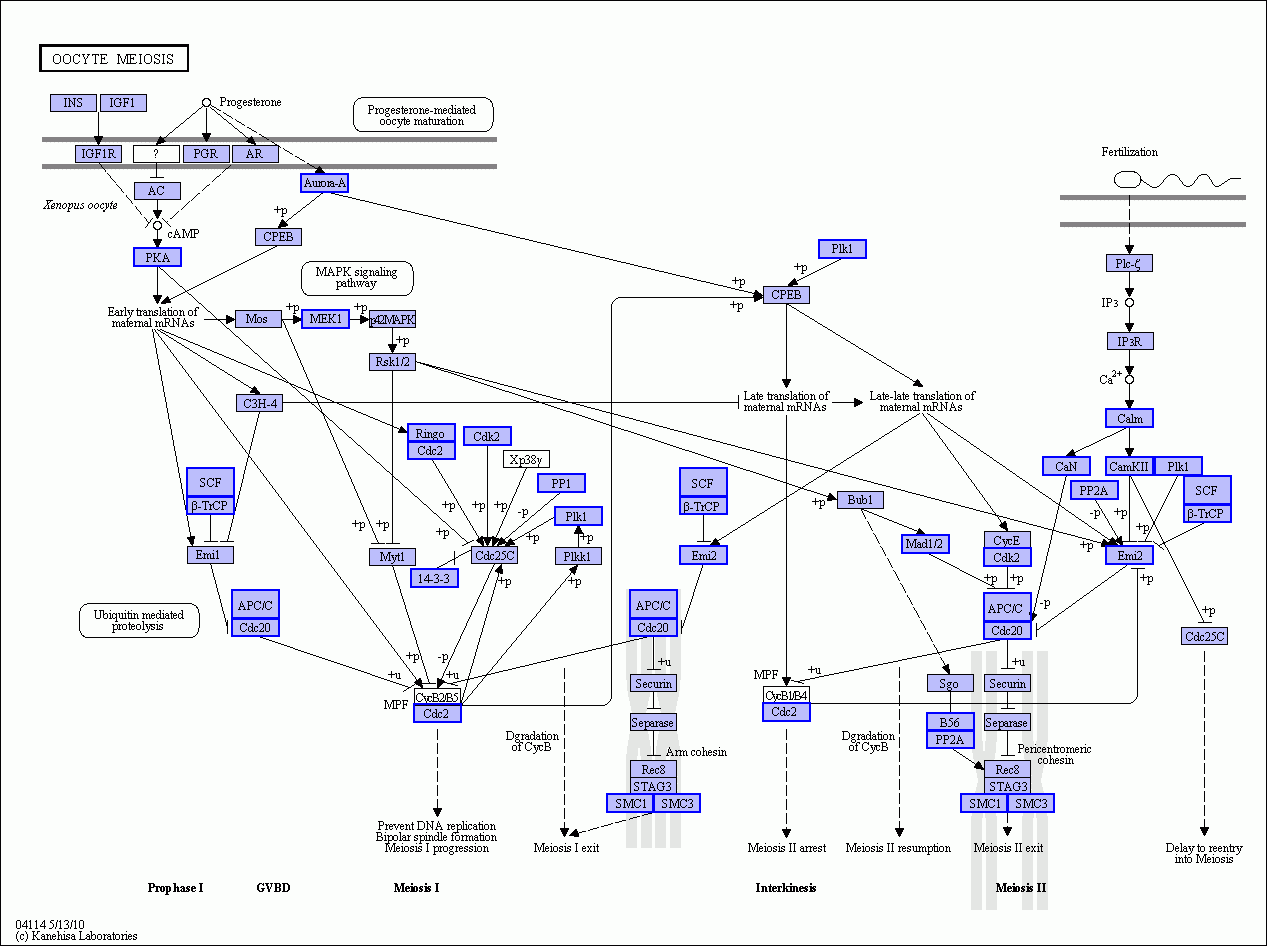

Supplement: Table S4 — KEGG Classification of the unigenes. (ZIP) [file pone.0079516.s004.zip › Kegg/Pathway_Map/ko04114.png]

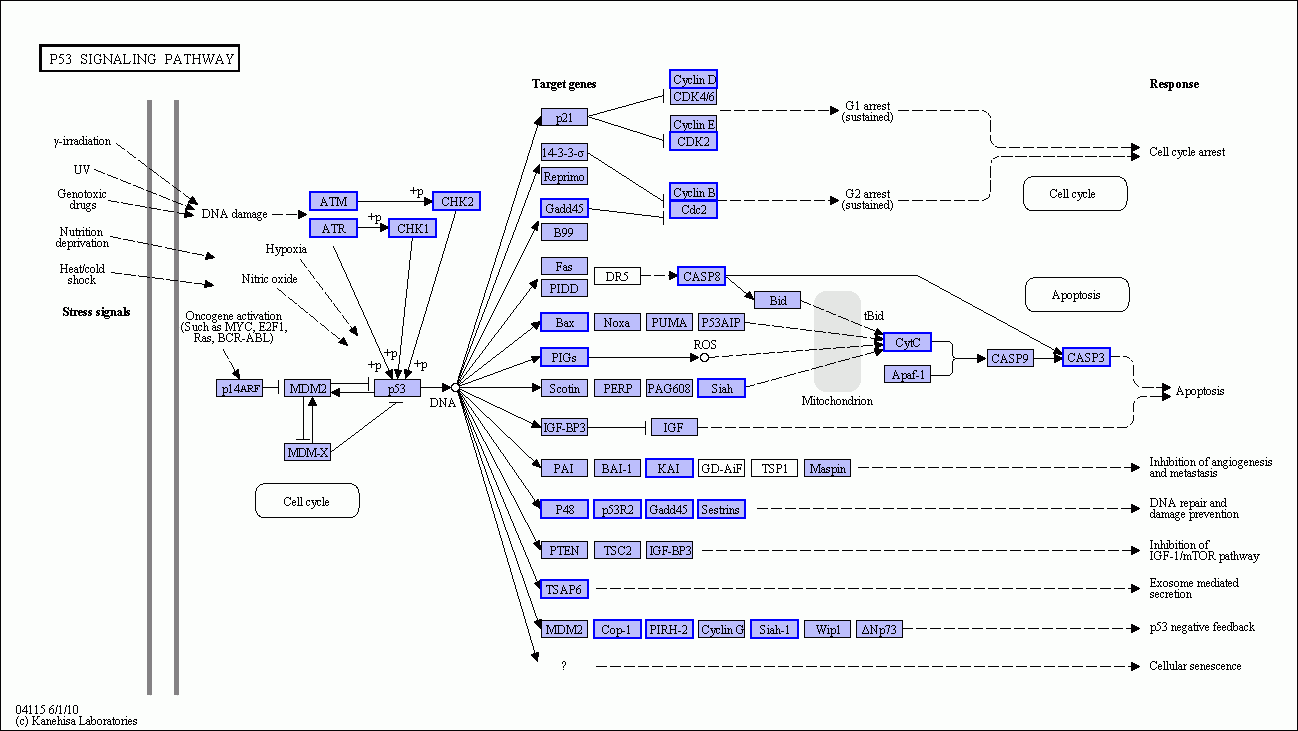

Supplement: Table S4 — KEGG Classification of the unigenes. (ZIP) [file pone.0079516.s004.zip › Kegg/Pathway_Map/ko04115.png]

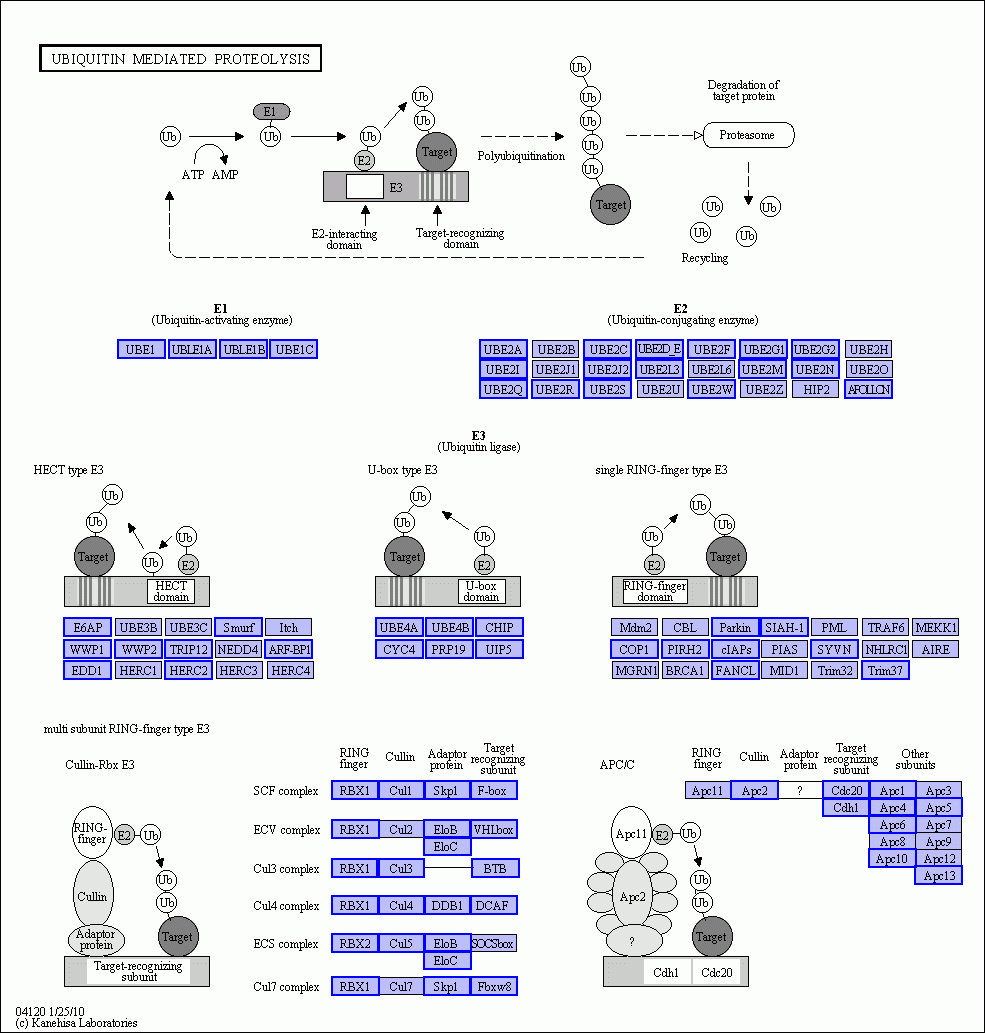

Supplement: Table S4 — KEGG Classification of the unigenes. (ZIP) [file pone.0079516.s004.zip › Kegg/Pathway_Map/ko04120.png]

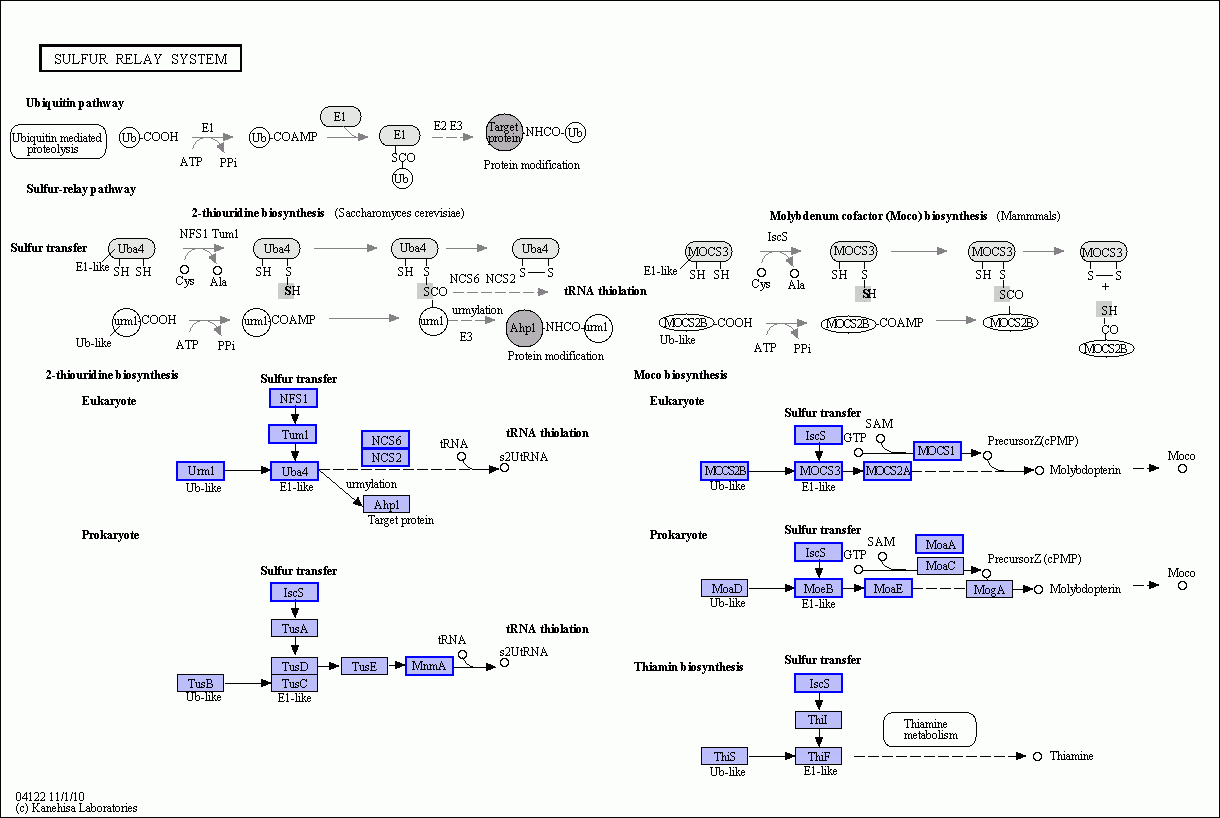

Supplement: Table S4 — KEGG Classification of the unigenes. (ZIP) [file pone.0079516.s004.zip › Kegg/Pathway_Map/ko04122.png]

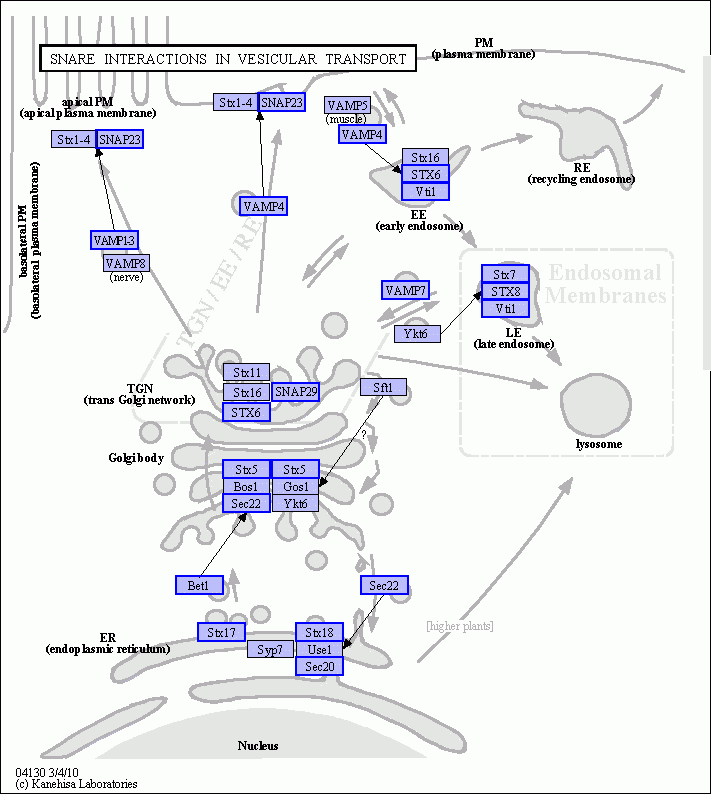

Supplement: Table S4 — KEGG Classification of the unigenes. (ZIP) [file pone.0079516.s004.zip › Kegg/Pathway_Map/ko04130.png]

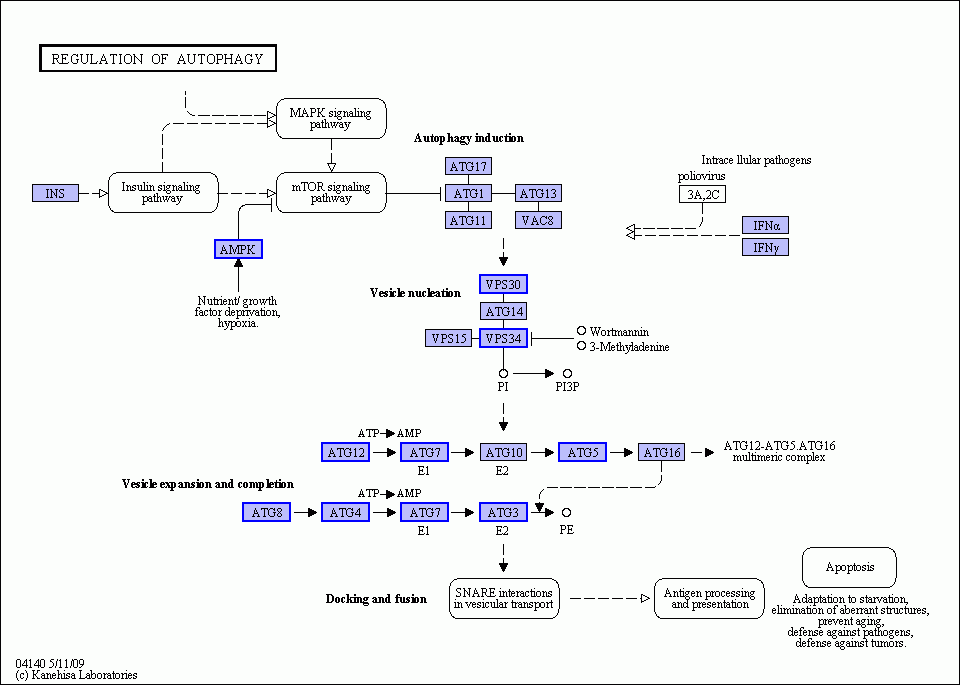

Supplement: Table S4 — KEGG Classification of the unigenes. (ZIP) [file pone.0079516.s004.zip › Kegg/Pathway_Map/ko04140.png]

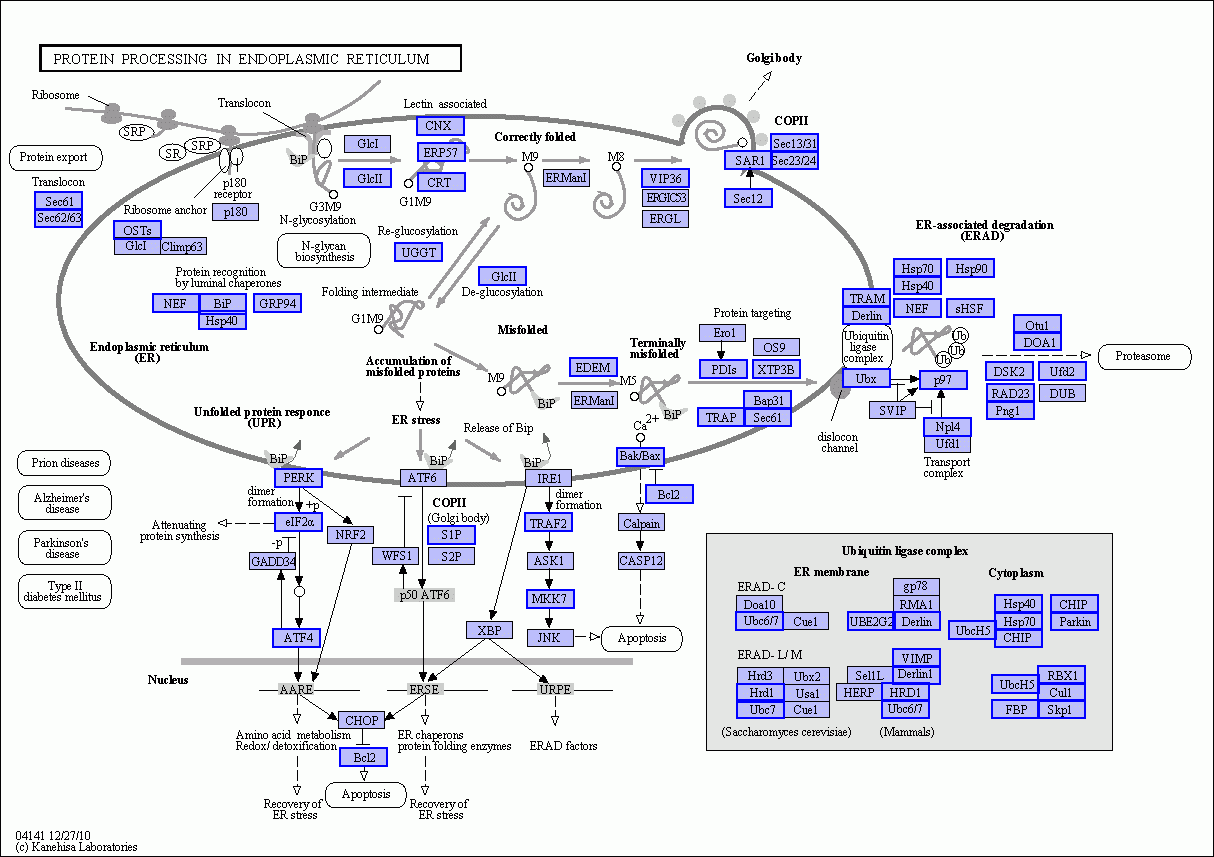

Supplement: Table S4 — KEGG Classification of the unigenes. (ZIP) [file pone.0079516.s004.zip › Kegg/Pathway_Map/ko04141.png]

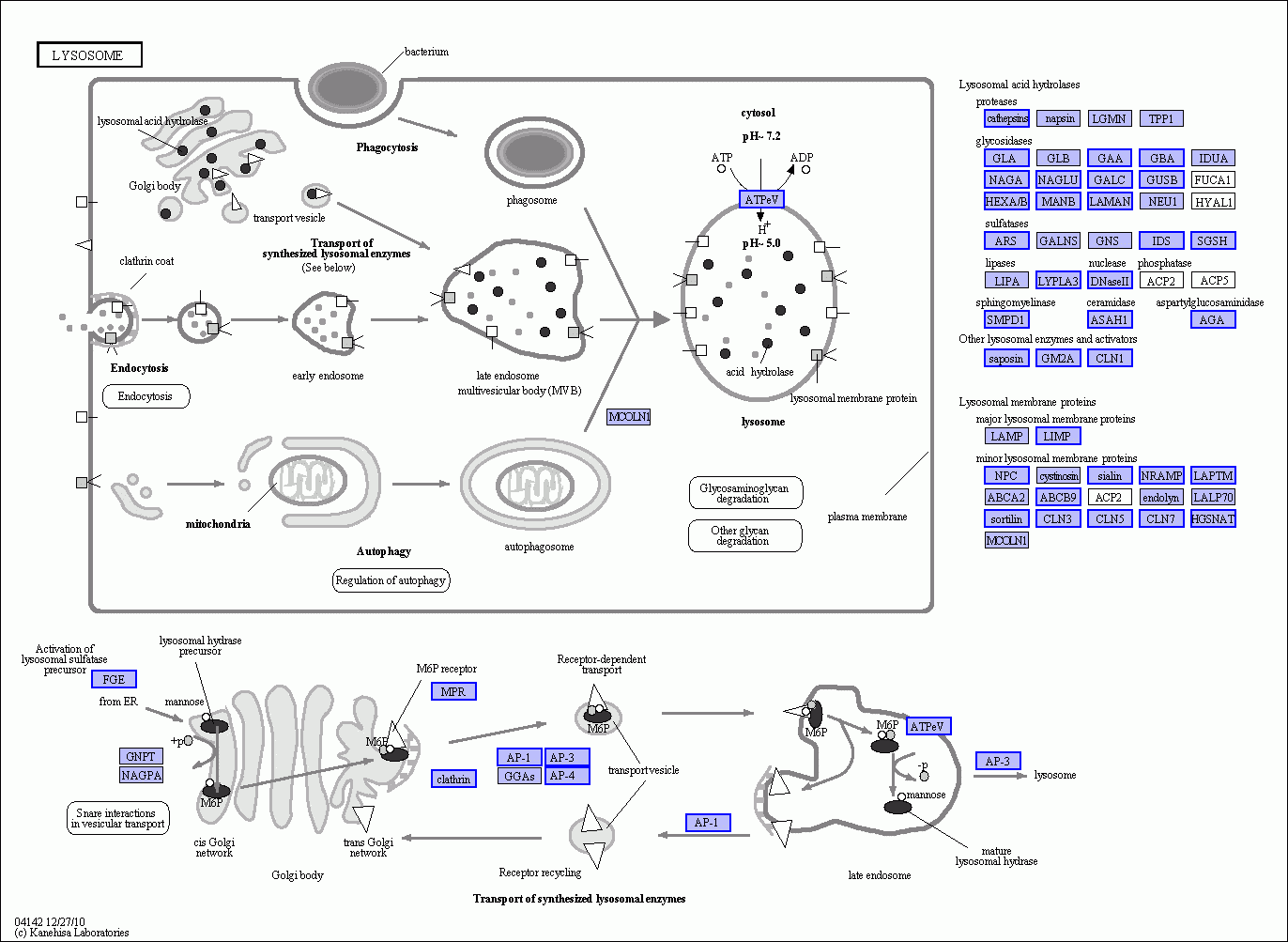

Supplement: Table S4 — KEGG Classification of the unigenes. (ZIP) [file pone.0079516.s004.zip › Kegg/Pathway_Map/ko04142.png]

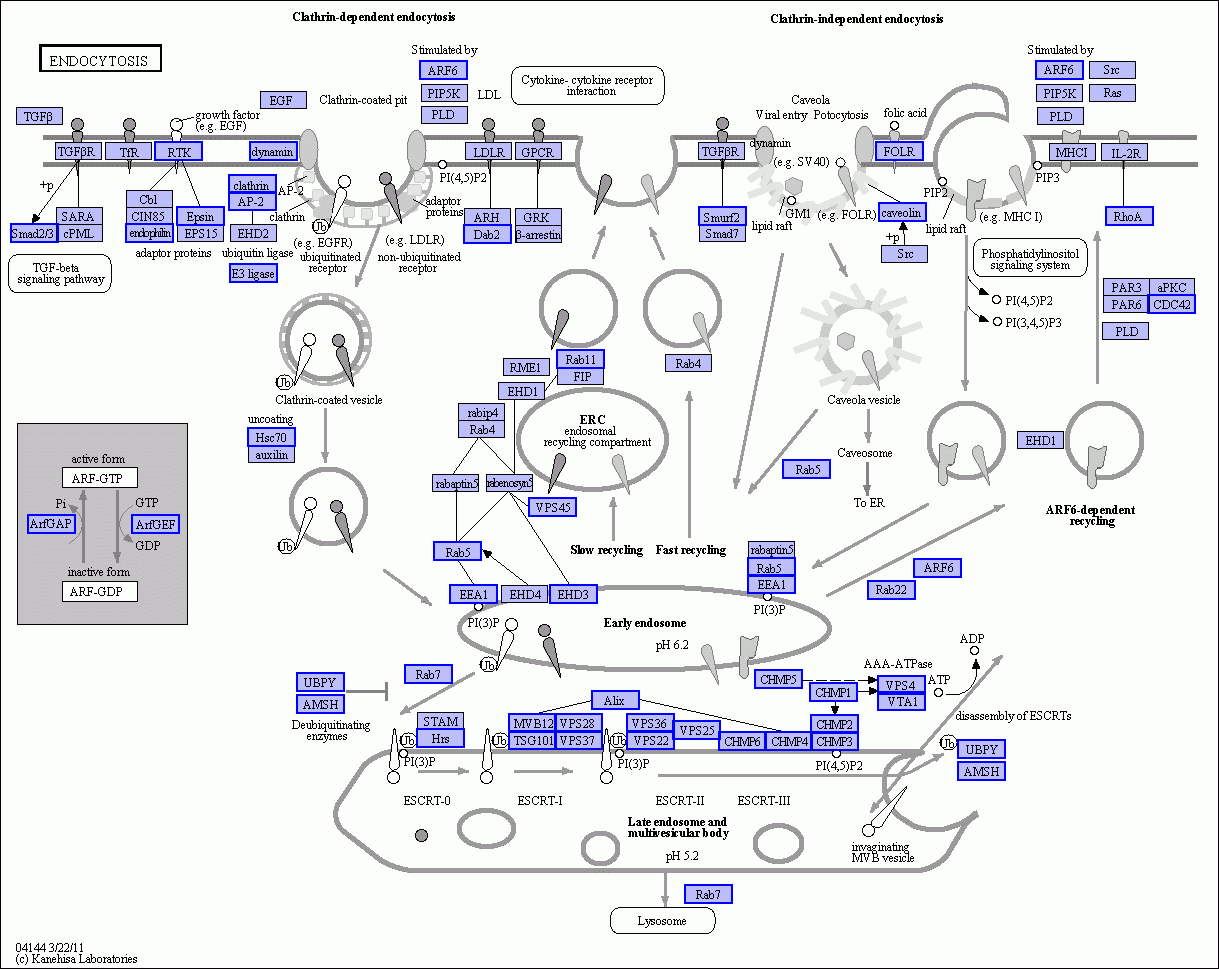

Supplement: Table S4 — KEGG Classification of the unigenes. (ZIP) [file pone.0079516.s004.zip › Kegg/Pathway_Map/ko04144.png]

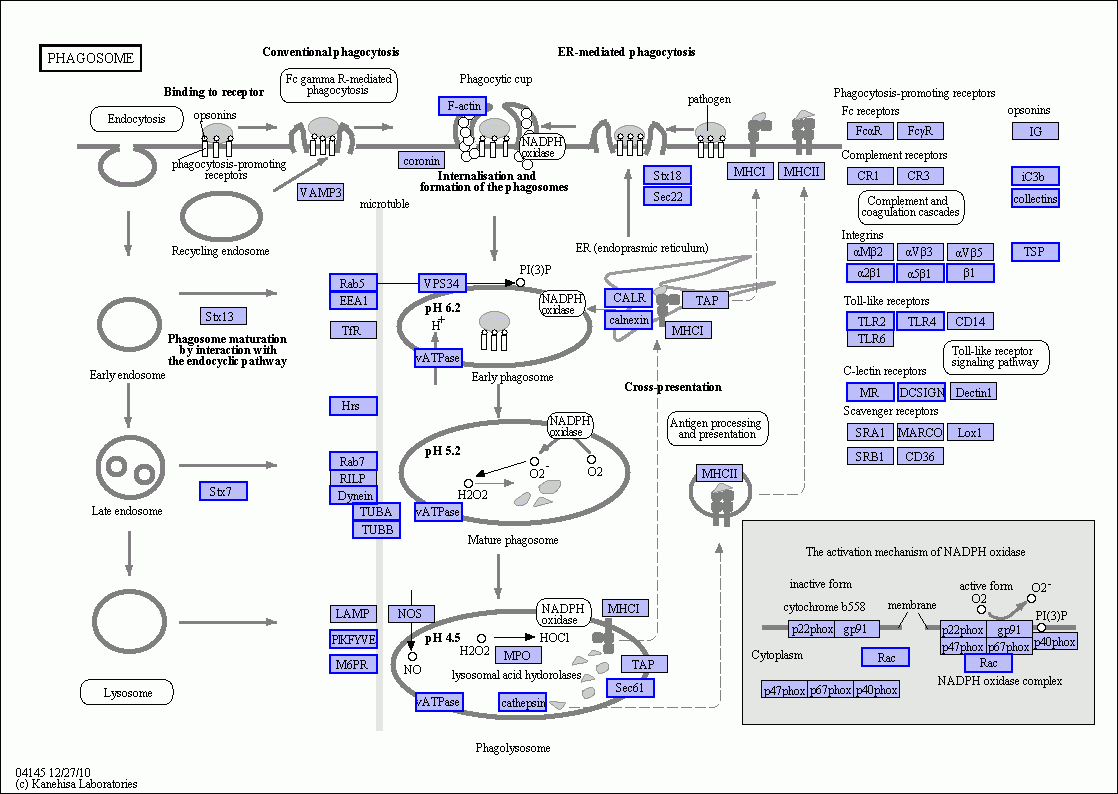

Supplement: Table S4 — KEGG Classification of the unigenes. (ZIP) [file pone.0079516.s004.zip › Kegg/Pathway_Map/ko04145.png]

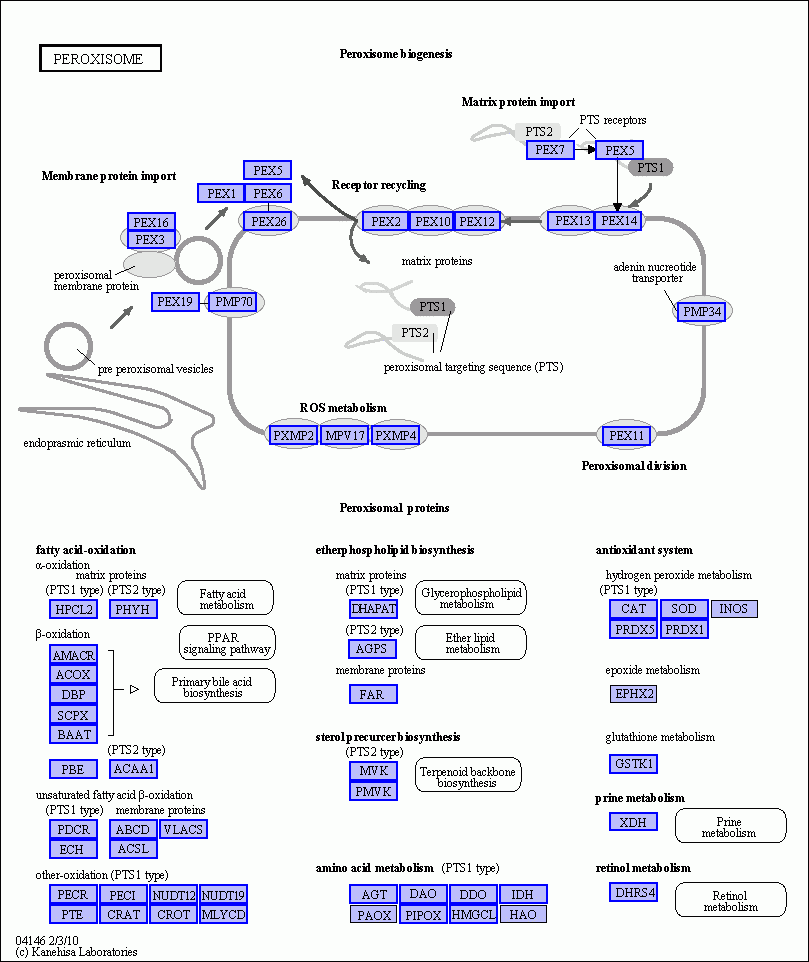

Supplement: Table S4 — KEGG Classification of the unigenes. (ZIP) [file pone.0079516.s004.zip › Kegg/Pathway_Map/ko04146.png]

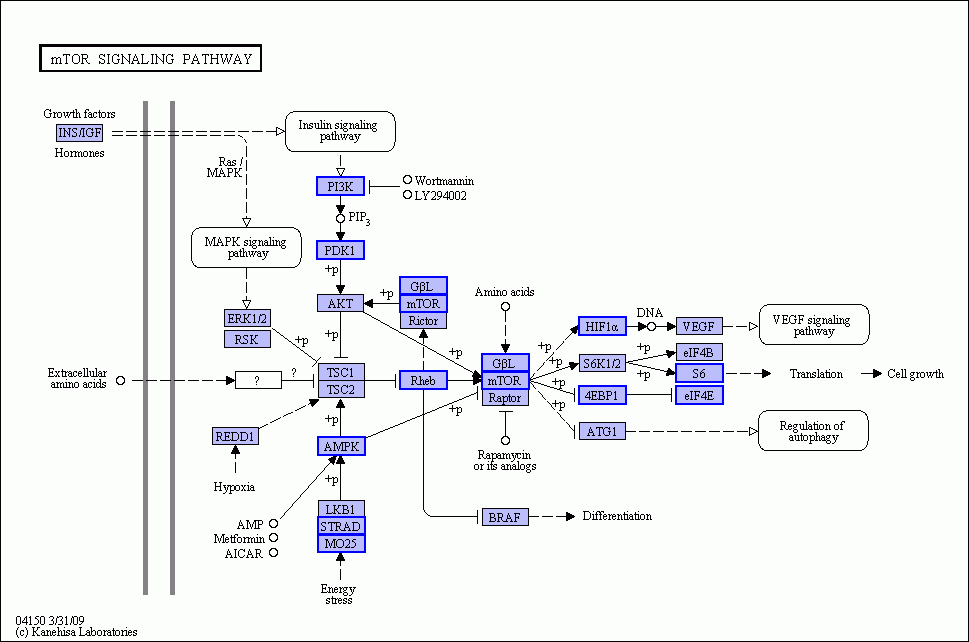

Supplement: Table S4 — KEGG Classification of the unigenes. (ZIP) [file pone.0079516.s004.zip › Kegg/Pathway_Map/ko04150.png]

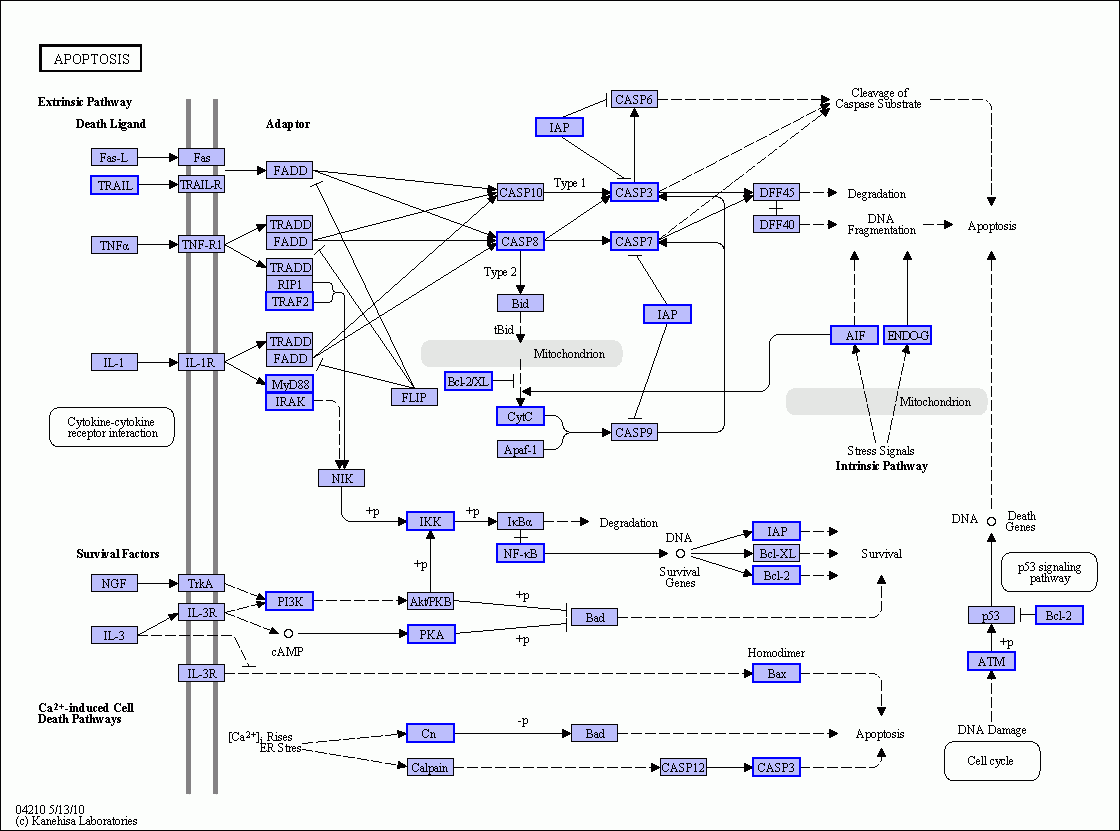

Supplement: Table S4 — KEGG Classification of the unigenes. (ZIP) [file pone.0079516.s004.zip › Kegg/Pathway_Map/ko04210.png]

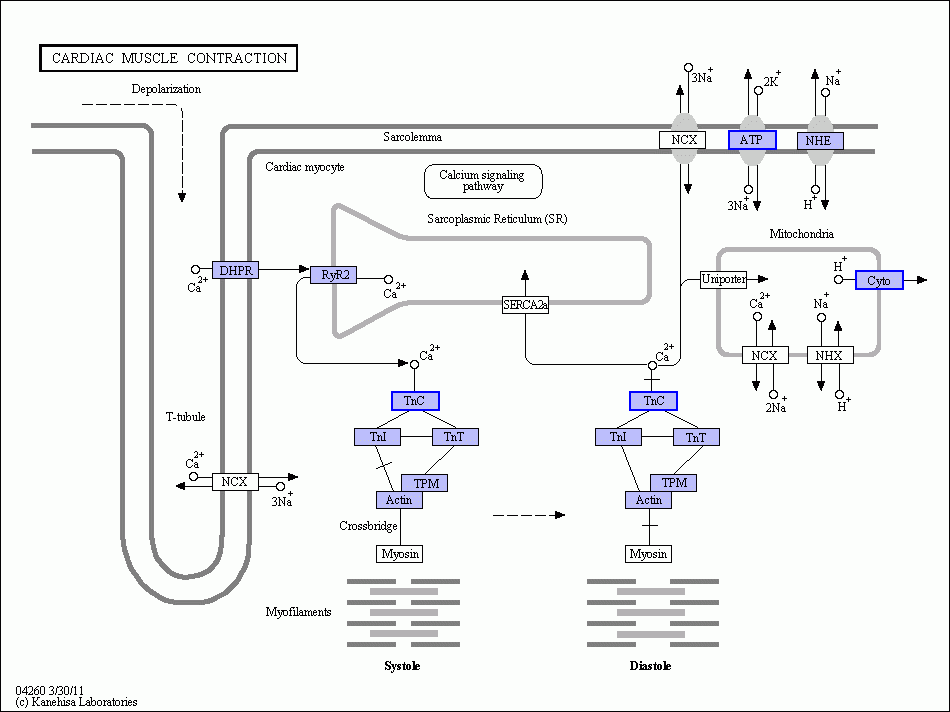

Supplement: Table S4 — KEGG Classification of the unigenes. (ZIP) [file pone.0079516.s004.zip › Kegg/Pathway_Map/ko04260.png]

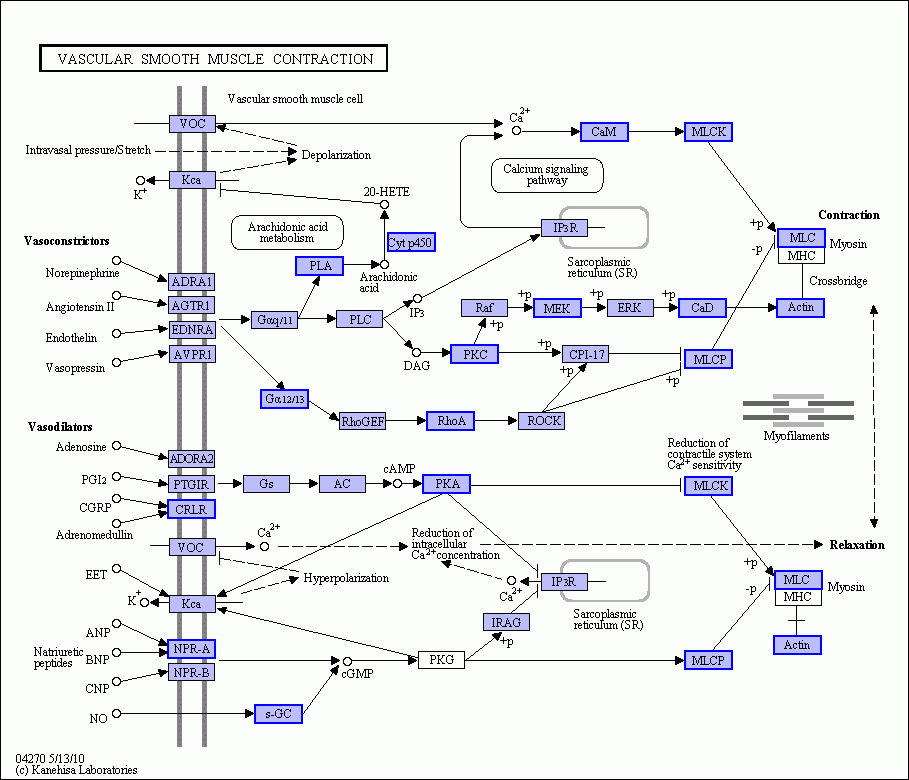

Supplement: Table S4 — KEGG Classification of the unigenes. (ZIP) [file pone.0079516.s004.zip › Kegg/Pathway_Map/ko04270.png]

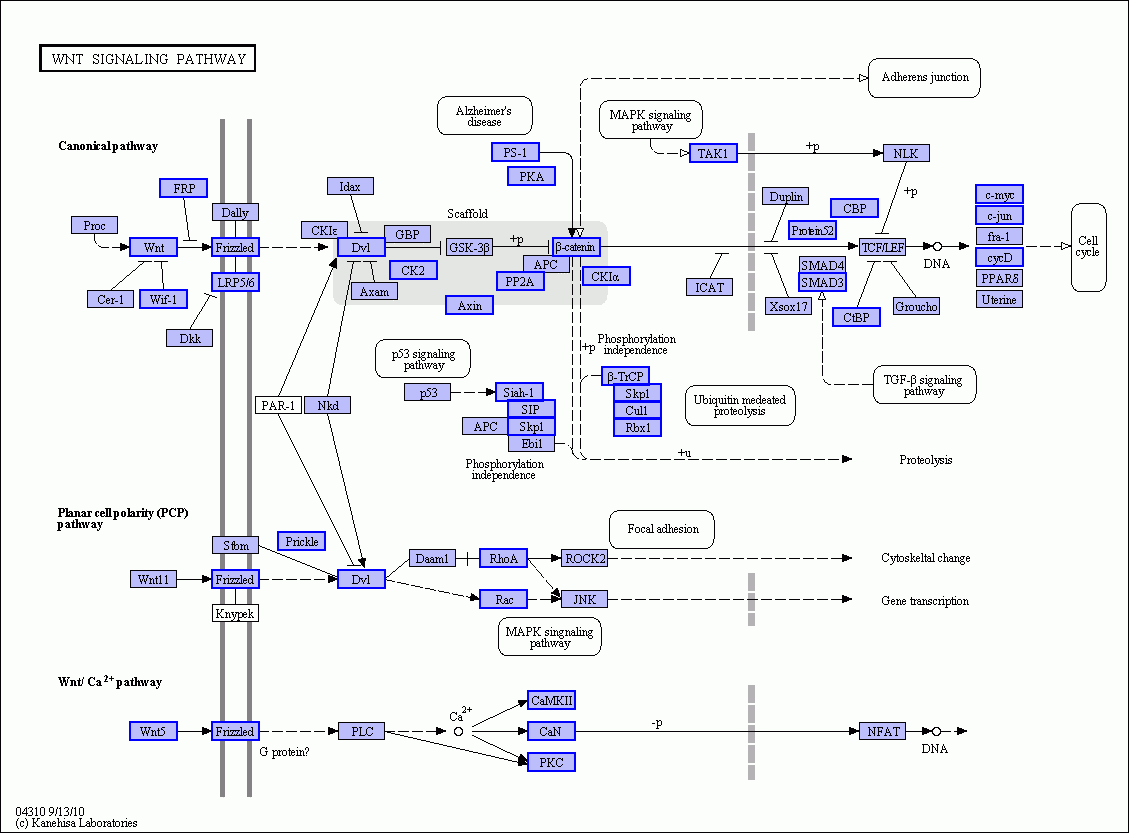

Supplement: Table S4 — KEGG Classification of the unigenes. (ZIP) [file pone.0079516.s004.zip › Kegg/Pathway_Map/ko04310.png]

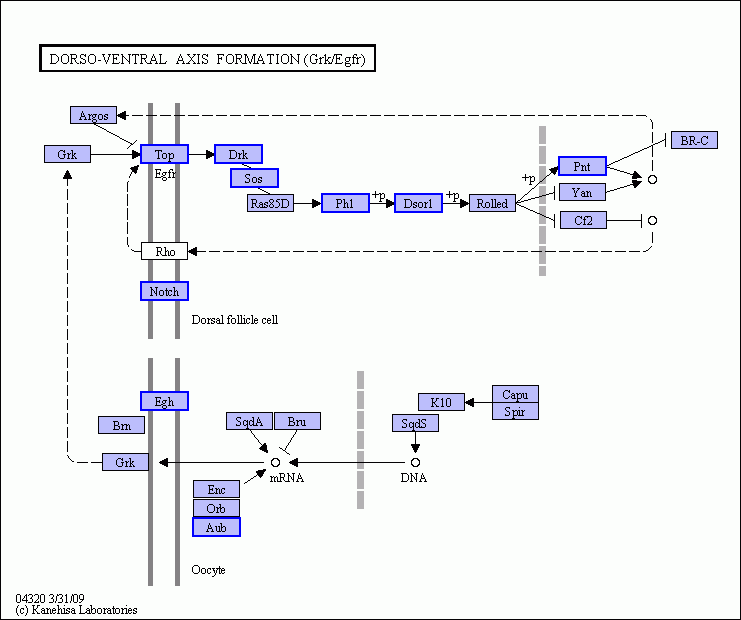

Supplement: Table S4 — KEGG Classification of the unigenes. (ZIP) [file pone.0079516.s004.zip › Kegg/Pathway_Map/ko04320.png]

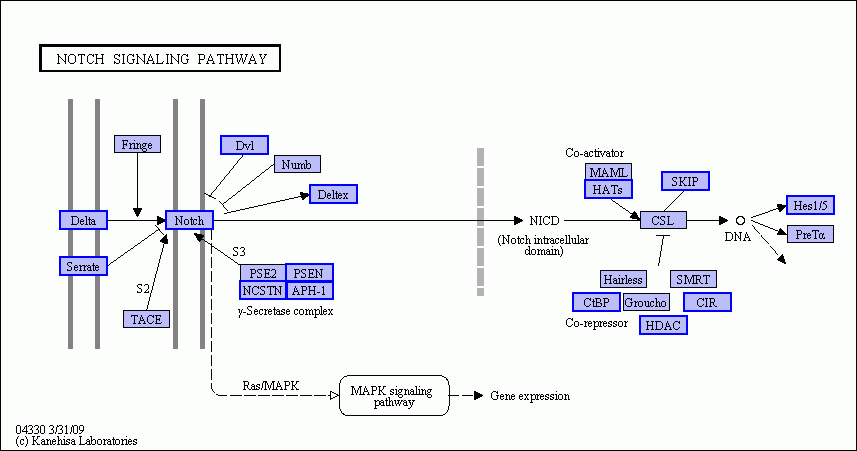

Supplement: Table S4 — KEGG Classification of the unigenes. (ZIP) [file pone.0079516.s004.zip › Kegg/Pathway_Map/ko04330.png]

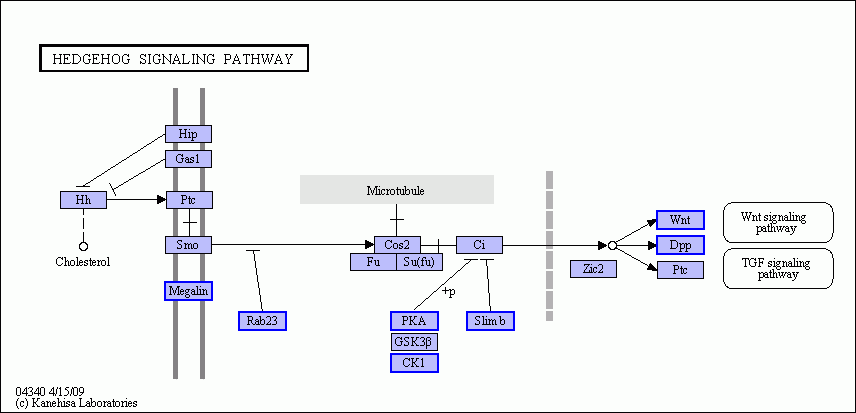

Supplement: Table S4 — KEGG Classification of the unigenes. (ZIP) [file pone.0079516.s004.zip › Kegg/Pathway_Map/ko04340.png]

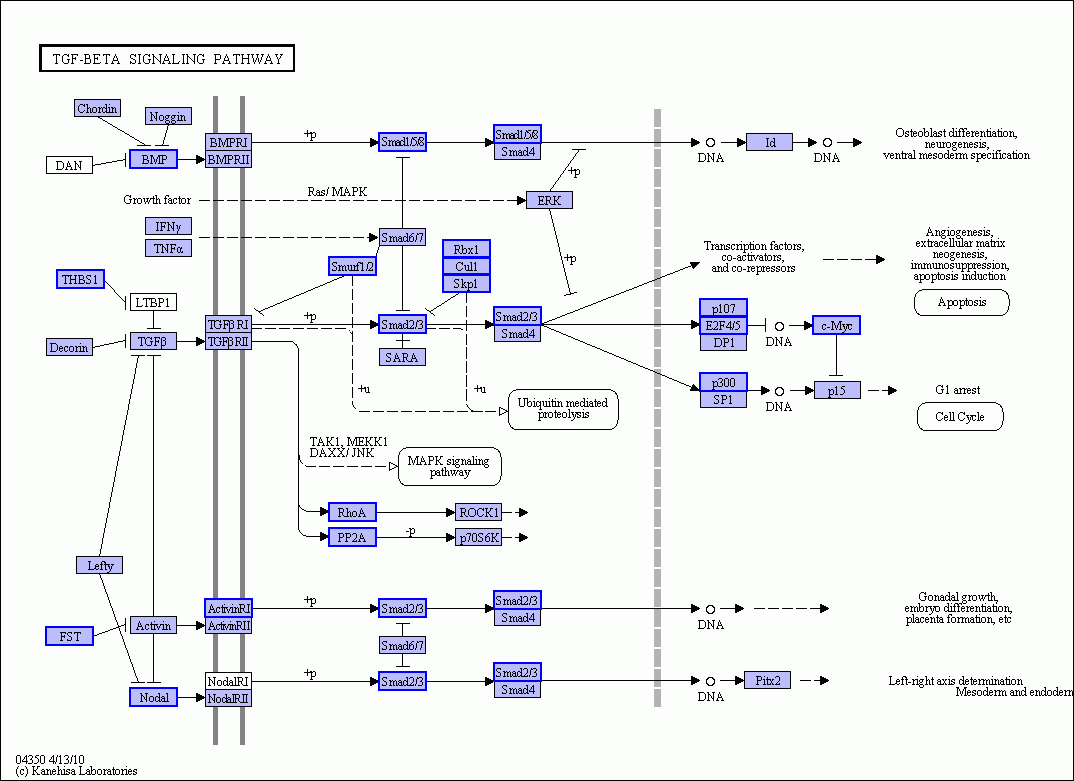

Supplement: Table S4 — KEGG Classification of the unigenes. (ZIP) [file pone.0079516.s004.zip › Kegg/Pathway_Map/ko04350.png]

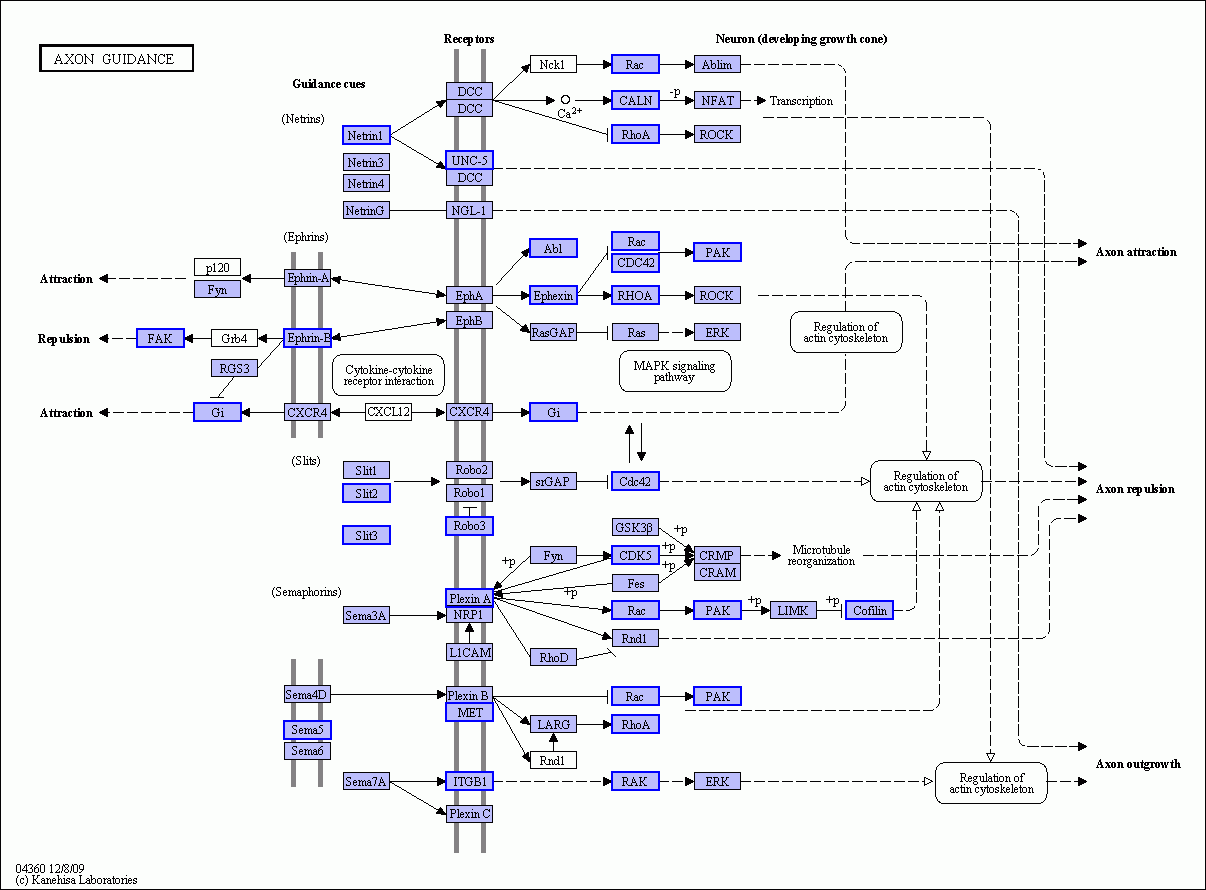

Supplement: Table S4 — KEGG Classification of the unigenes. (ZIP) [file pone.0079516.s004.zip › Kegg/Pathway_Map/ko04360.png]

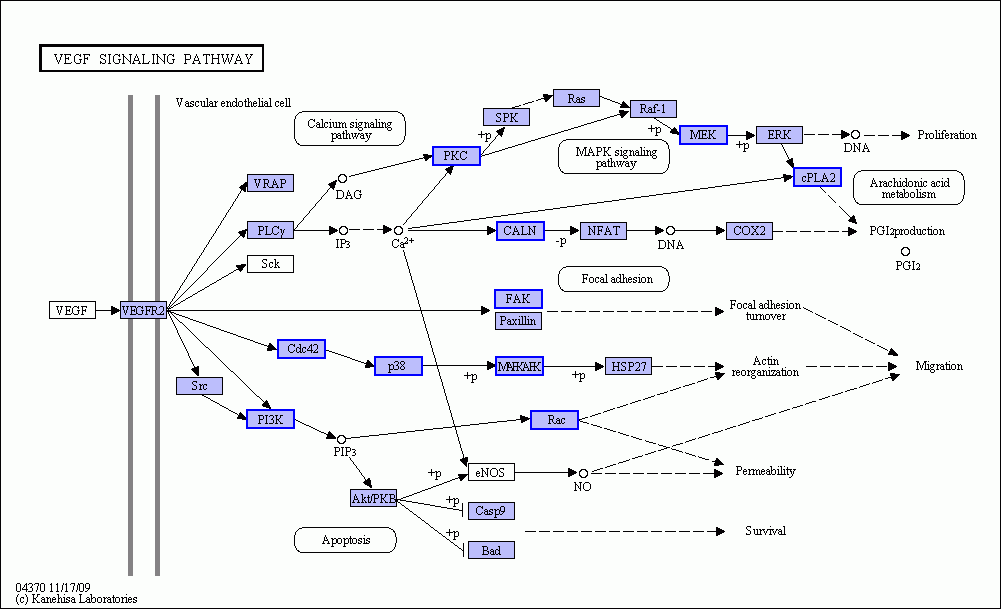

Supplement: Table S4 — KEGG Classification of the unigenes. (ZIP) [file pone.0079516.s004.zip › Kegg/Pathway_Map/ko04370.png]

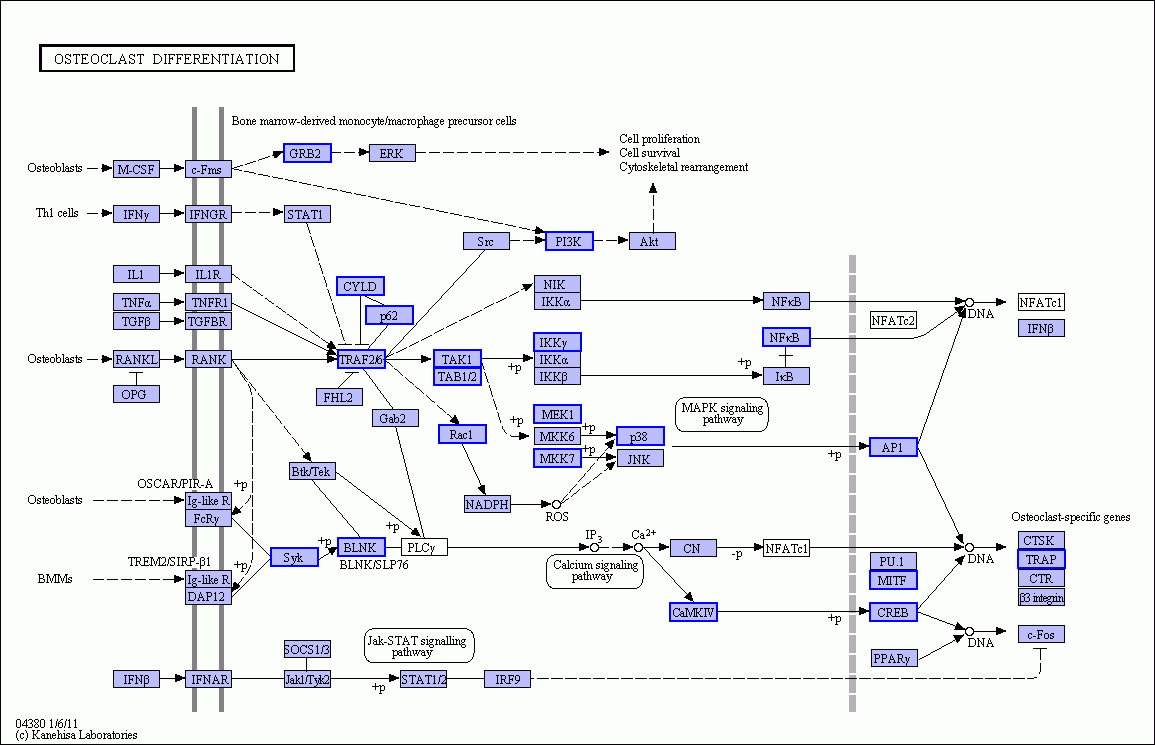

Supplement: Table S4 — KEGG Classification of the unigenes. (ZIP) [file pone.0079516.s004.zip › Kegg/Pathway_Map/ko04380.png]

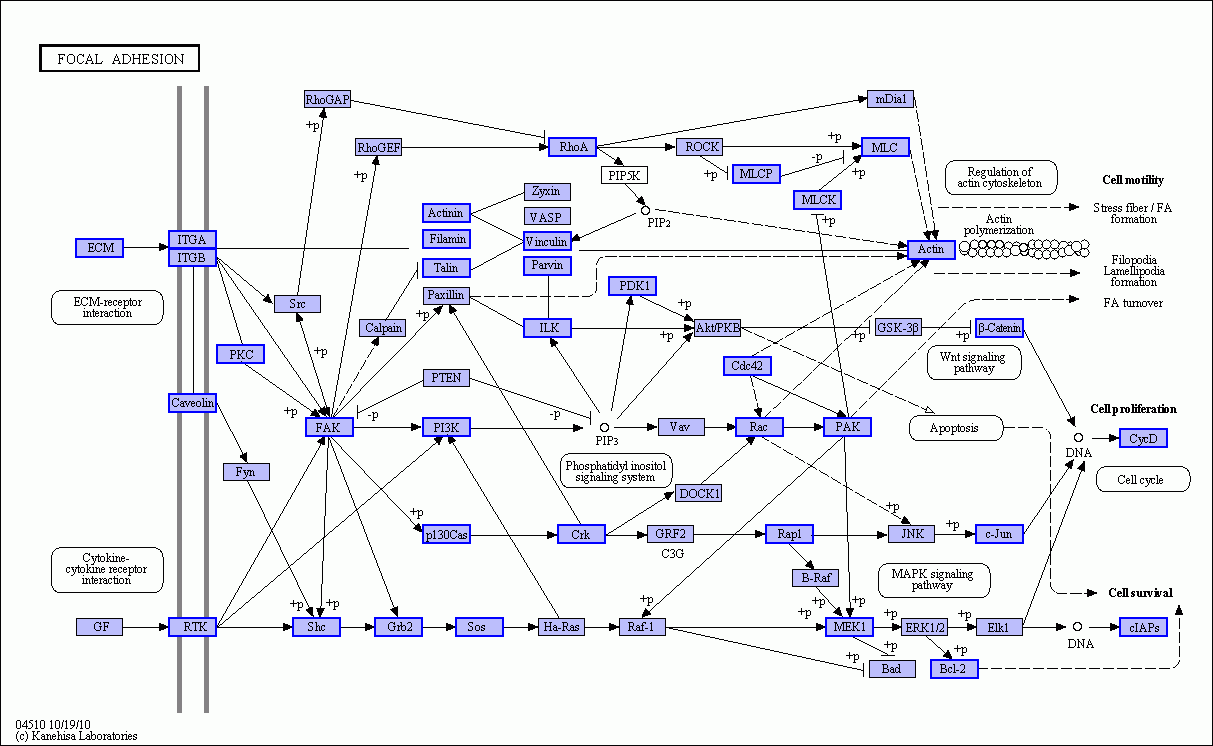

Supplement: Table S4 — KEGG Classification of the unigenes. (ZIP) [file pone.0079516.s004.zip › Kegg/Pathway_Map/ko04510.png]

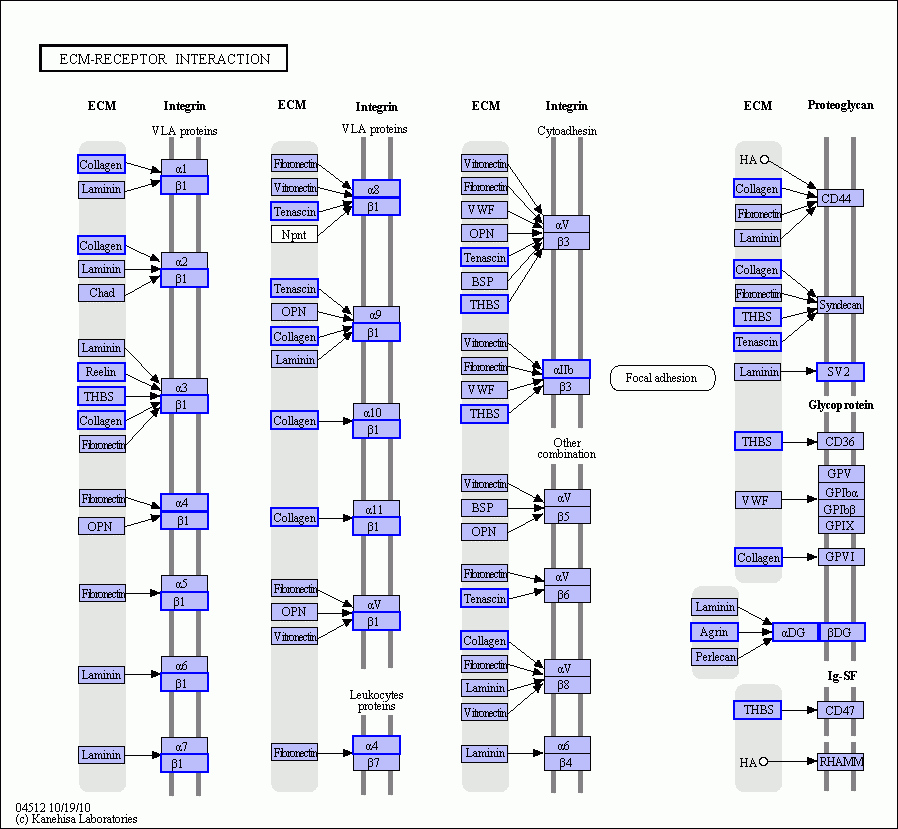

Supplement: Table S4 — KEGG Classification of the unigenes. (ZIP) [file pone.0079516.s004.zip › Kegg/Pathway_Map/ko04512.png]

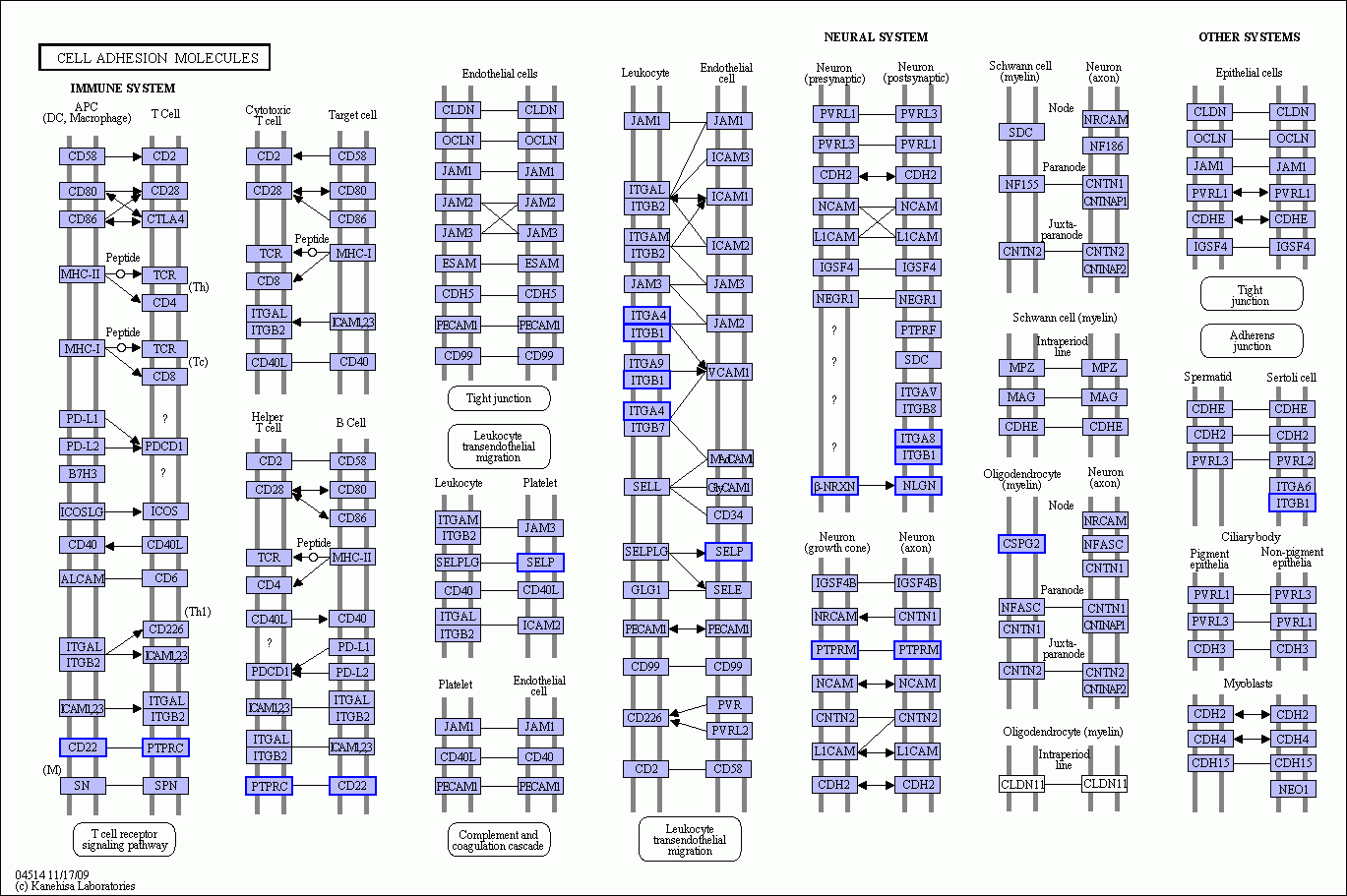

Supplement: Table S4 — KEGG Classification of the unigenes. (ZIP) [file pone.0079516.s004.zip › Kegg/Pathway_Map/ko04514.png]

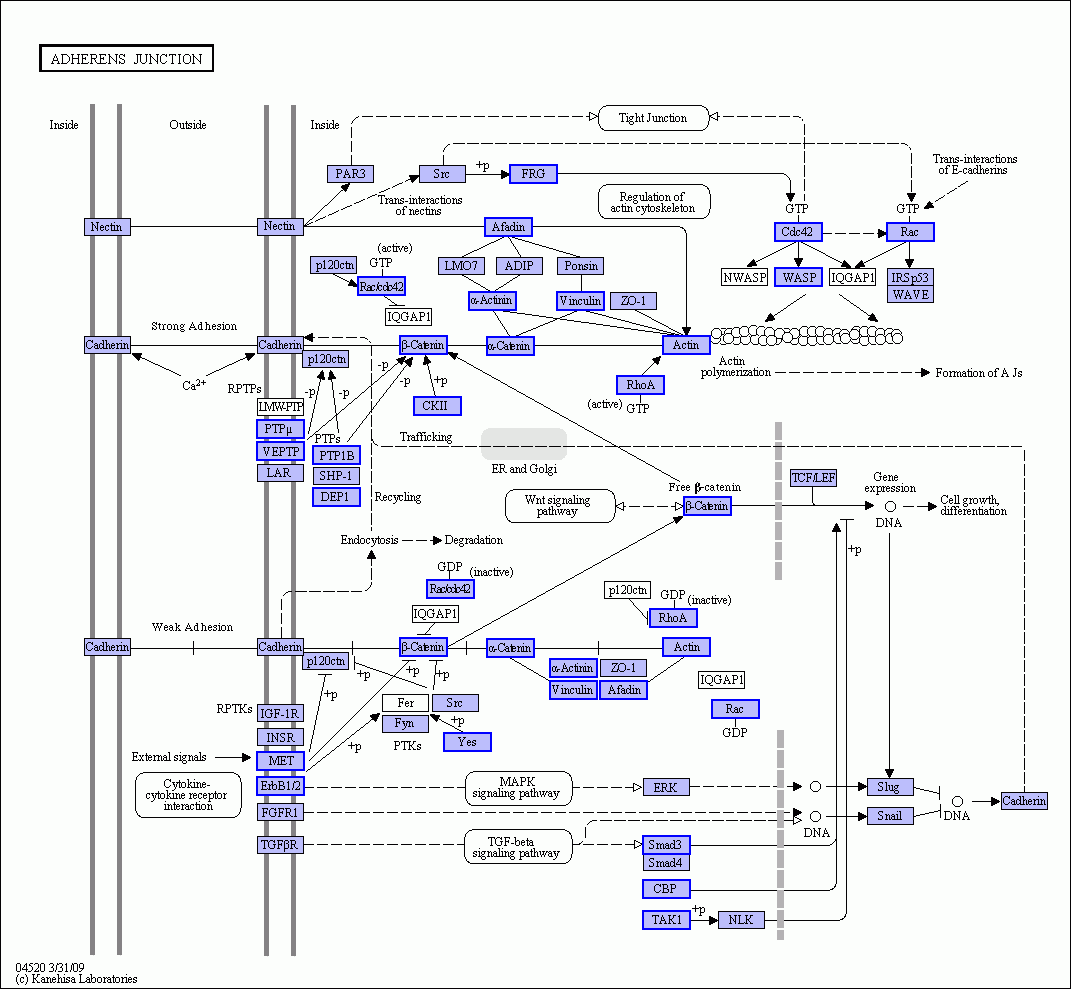

Supplement: Table S4 — KEGG Classification of the unigenes. (ZIP) [file pone.0079516.s004.zip › Kegg/Pathway_Map/ko04520.png]

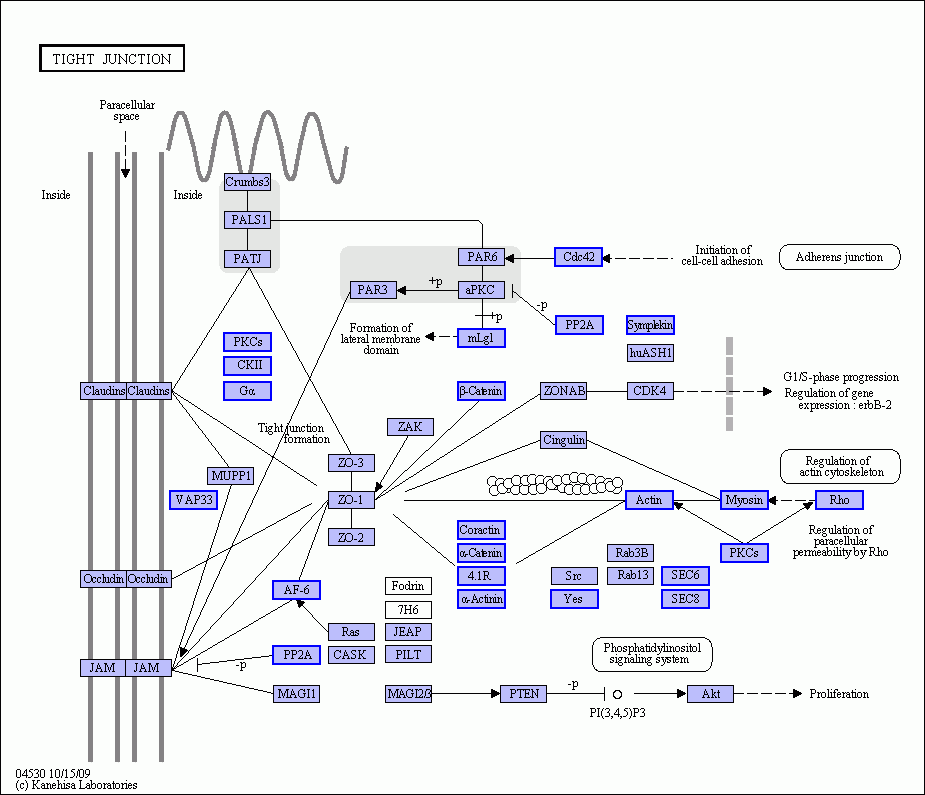

Supplement: Table S4 — KEGG Classification of the unigenes. (ZIP) [file pone.0079516.s004.zip › Kegg/Pathway_Map/ko04530.png]

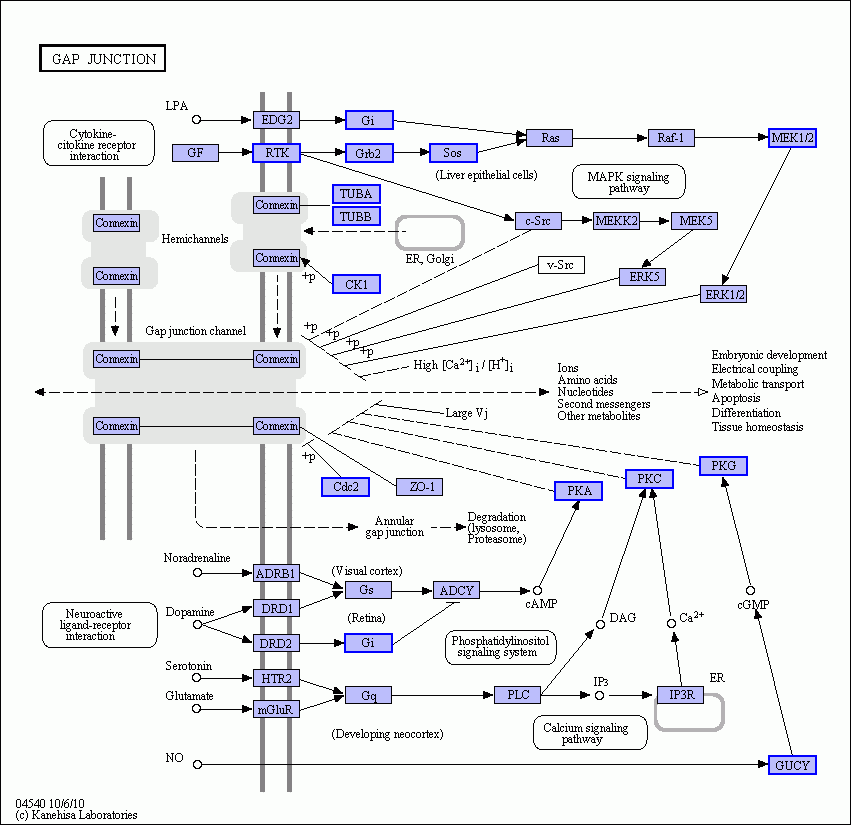

Supplement: Table S4 — KEGG Classification of the unigenes. (ZIP) [file pone.0079516.s004.zip › Kegg/Pathway_Map/ko04540.png]

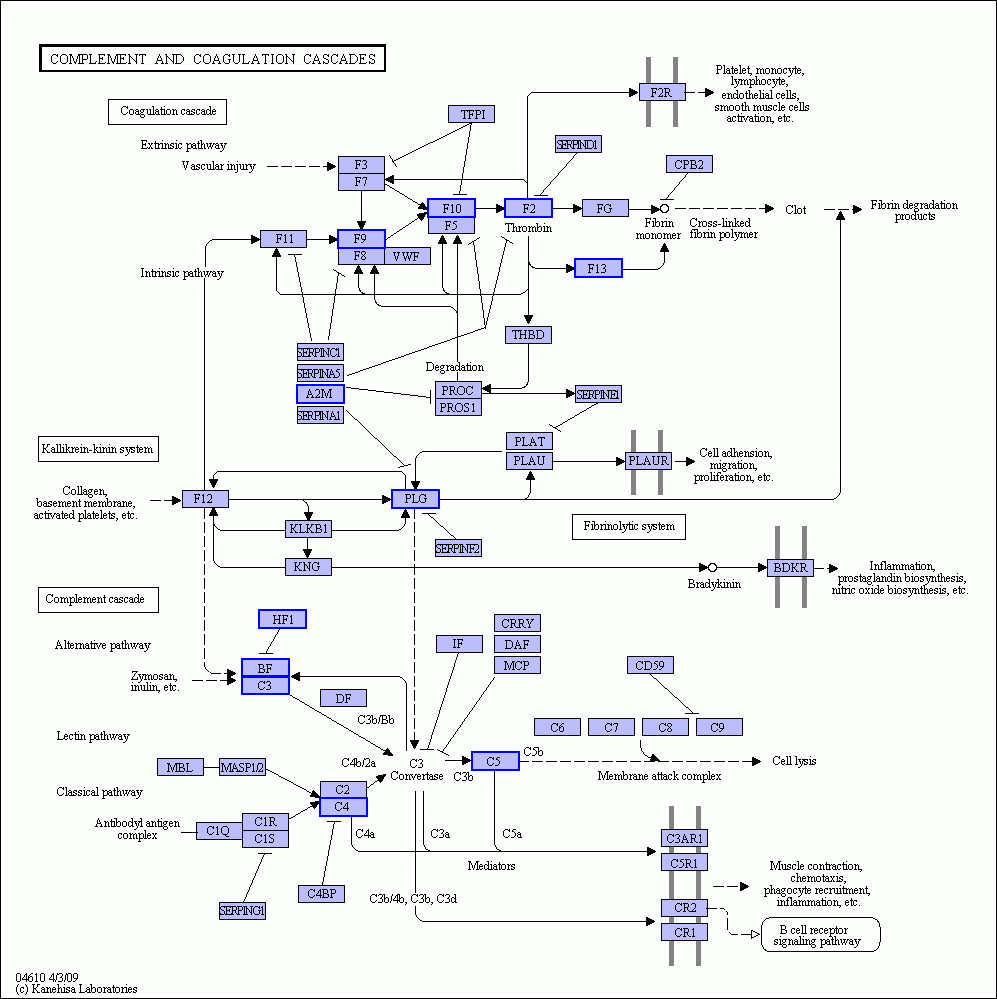

Supplement: Table S4 — KEGG Classification of the unigenes. (ZIP) [file pone.0079516.s004.zip › Kegg/Pathway_Map/ko04610.png]

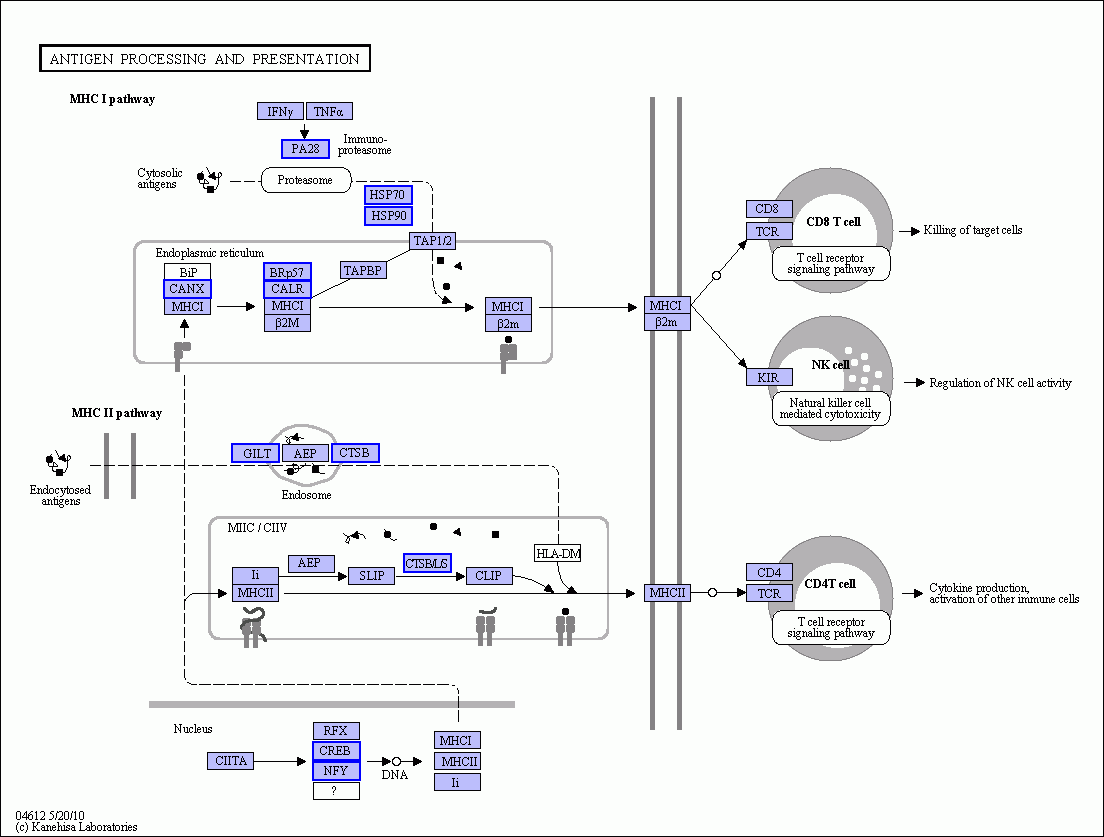

Supplement: Table S4 — KEGG Classification of the unigenes. (ZIP) [file pone.0079516.s004.zip › Kegg/Pathway_Map/ko04612.png]

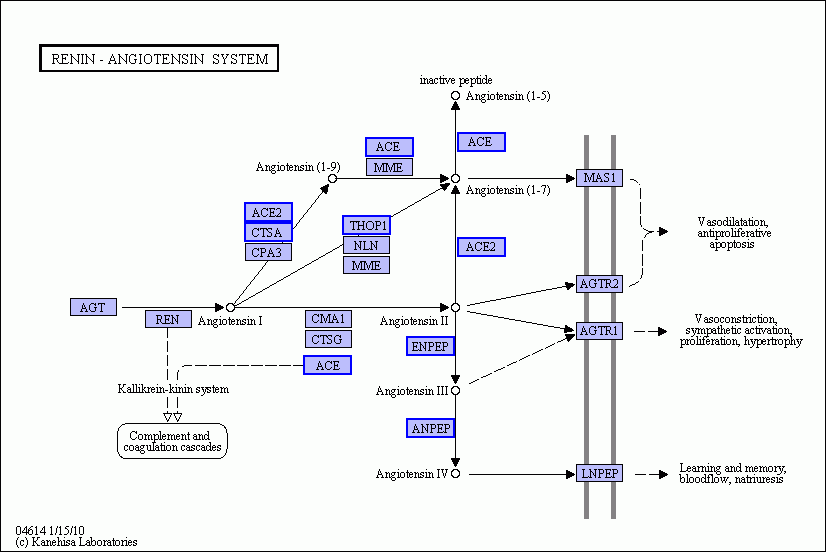

Supplement: Table S4 — KEGG Classification of the unigenes. (ZIP) [file pone.0079516.s004.zip › Kegg/Pathway_Map/ko04614.png]

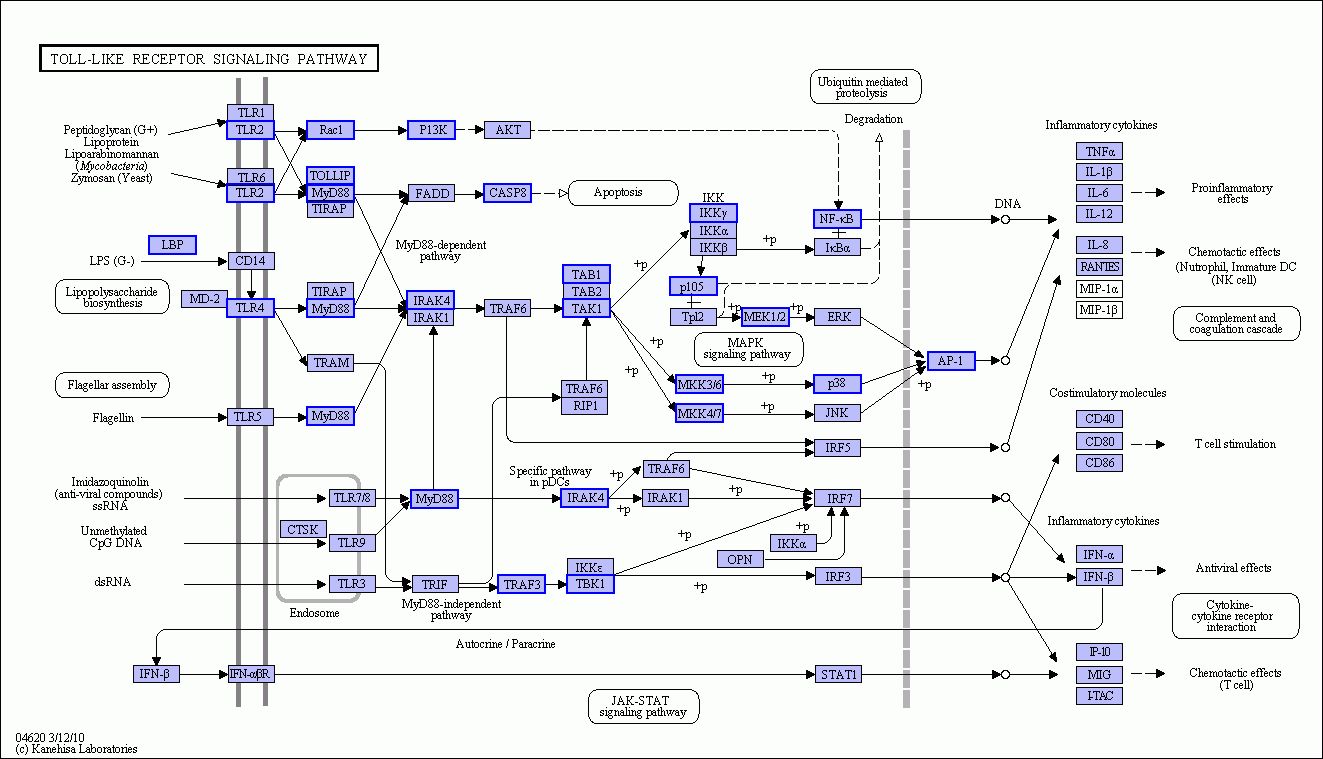

Supplement: Table S4 — KEGG Classification of the unigenes. (ZIP) [file pone.0079516.s004.zip › Kegg/Pathway_Map/ko04620.png]

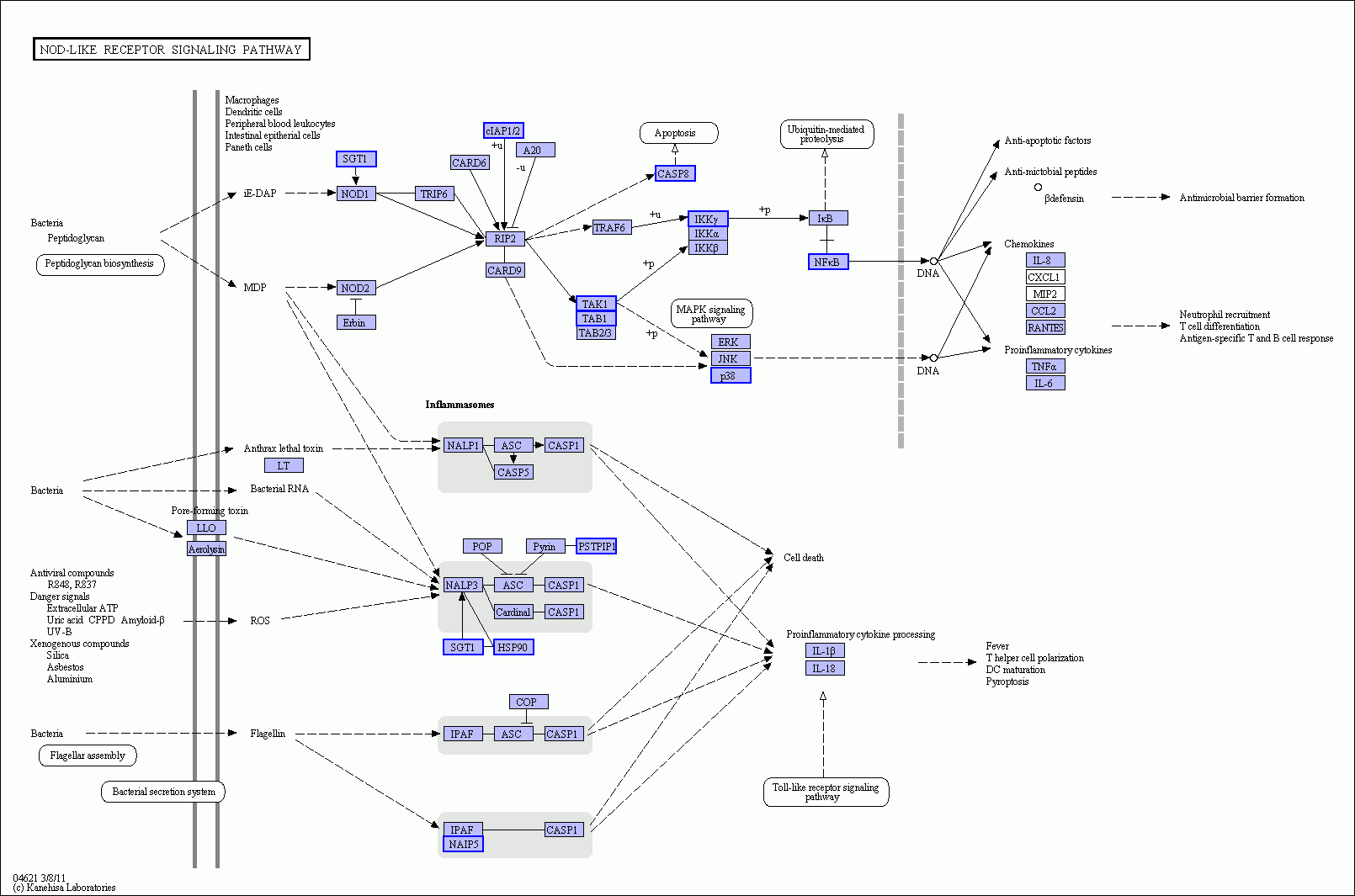

Supplement: Table S4 — KEGG Classification of the unigenes. (ZIP) [file pone.0079516.s004.zip › Kegg/Pathway_Map/ko04621.png]

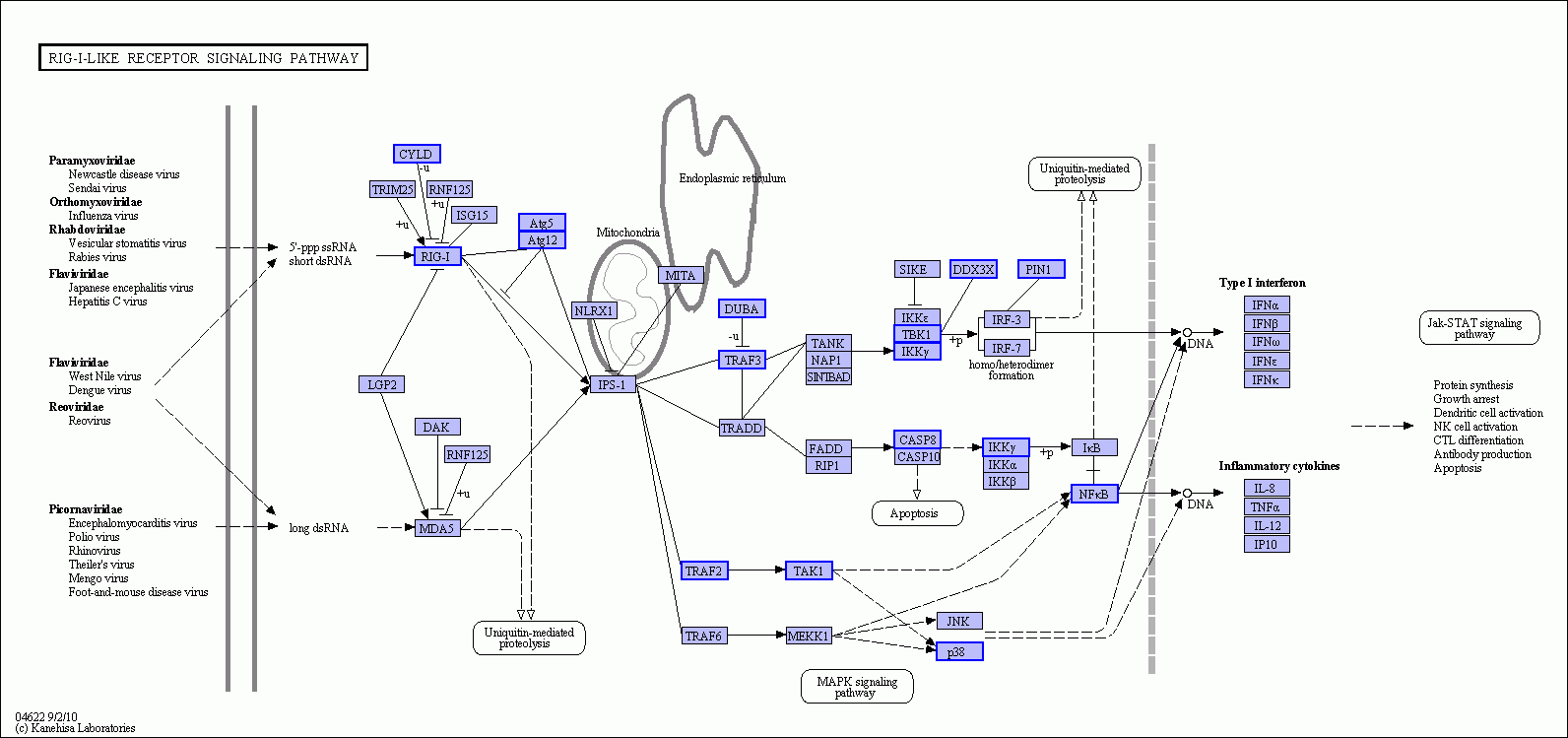

Supplement: Table S4 — KEGG Classification of the unigenes. (ZIP) [file pone.0079516.s004.zip › Kegg/Pathway_Map/ko04622.png]

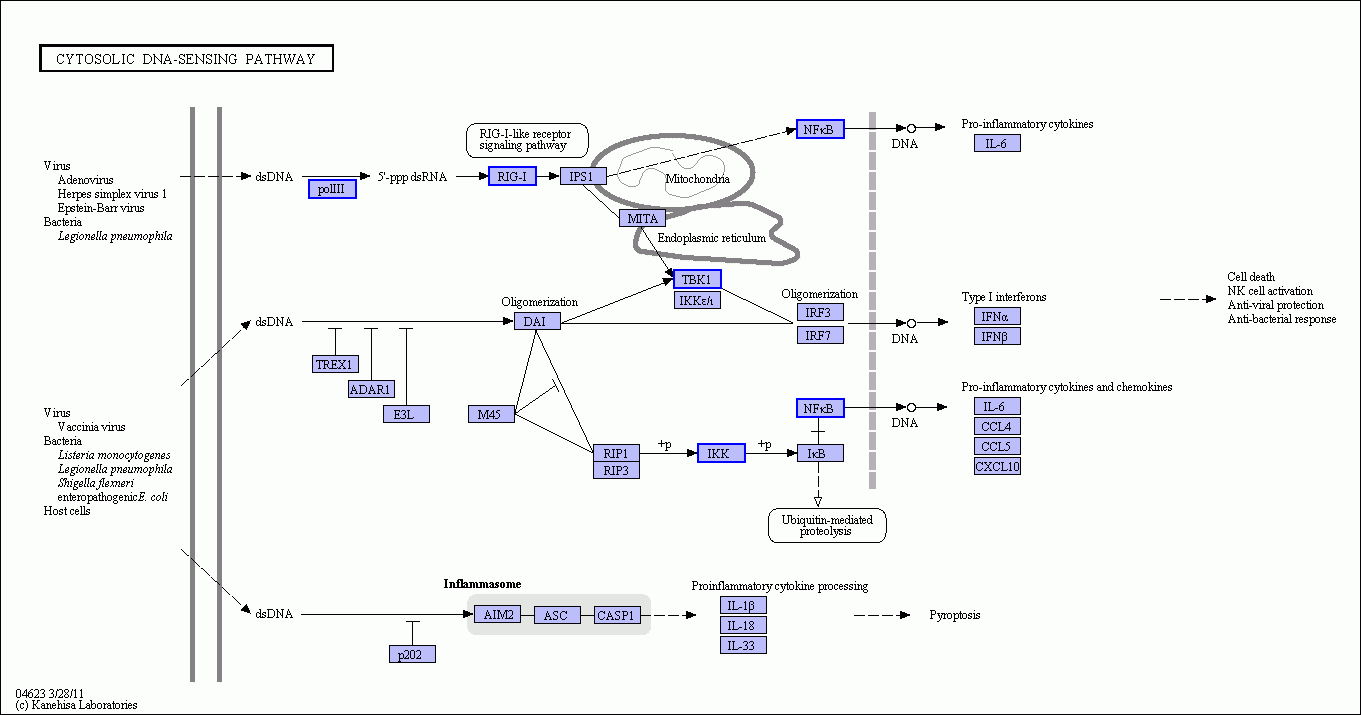

Supplement: Table S4 — KEGG Classification of the unigenes. (ZIP) [file pone.0079516.s004.zip › Kegg/Pathway_Map/ko04623.png]

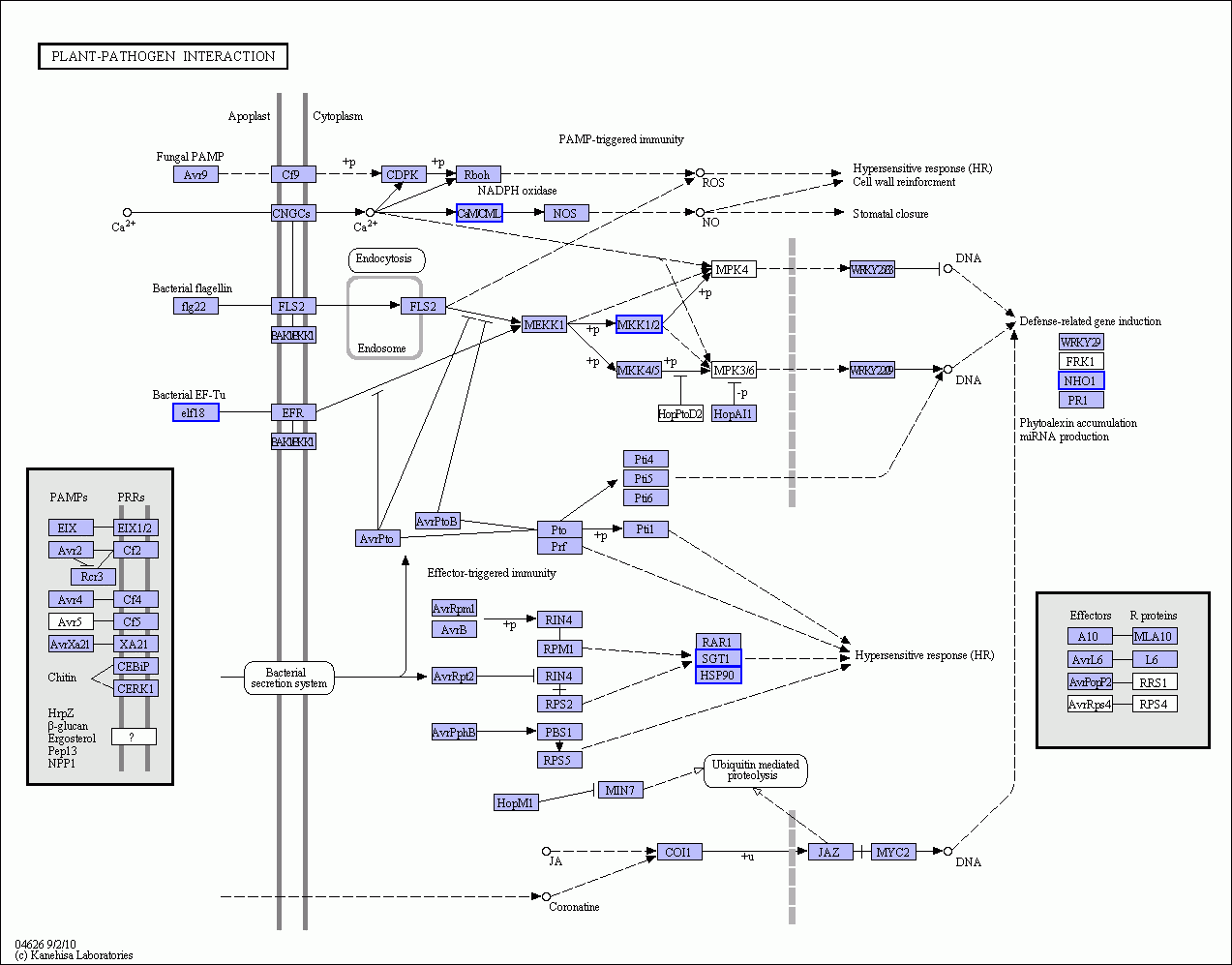

Supplement: Table S4 — KEGG Classification of the unigenes. (ZIP) [file pone.0079516.s004.zip › Kegg/Pathway_Map/ko04626.png]

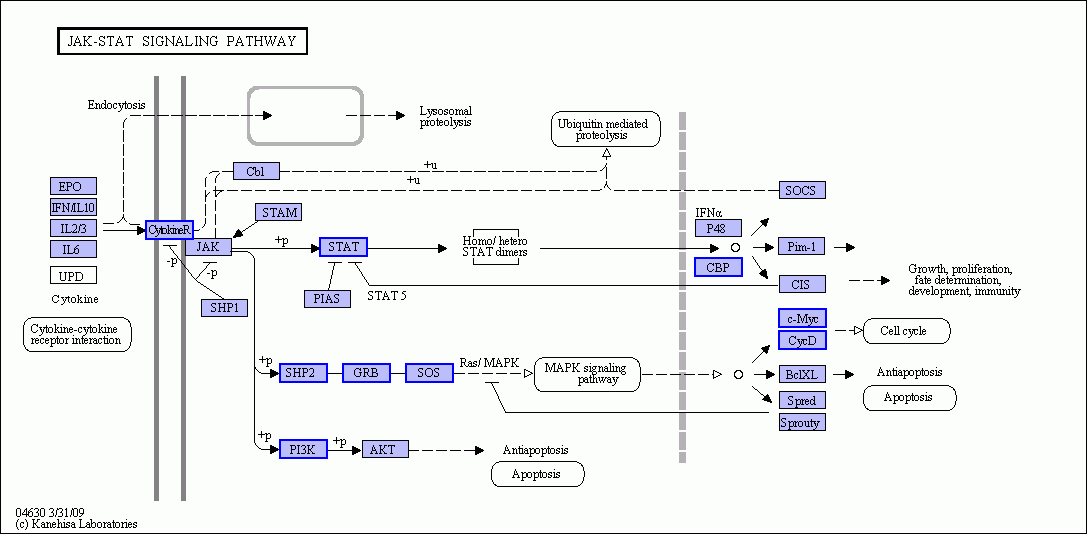

Supplement: Table S4 — KEGG Classification of the unigenes. (ZIP) [file pone.0079516.s004.zip › Kegg/Pathway_Map/ko04630.png]

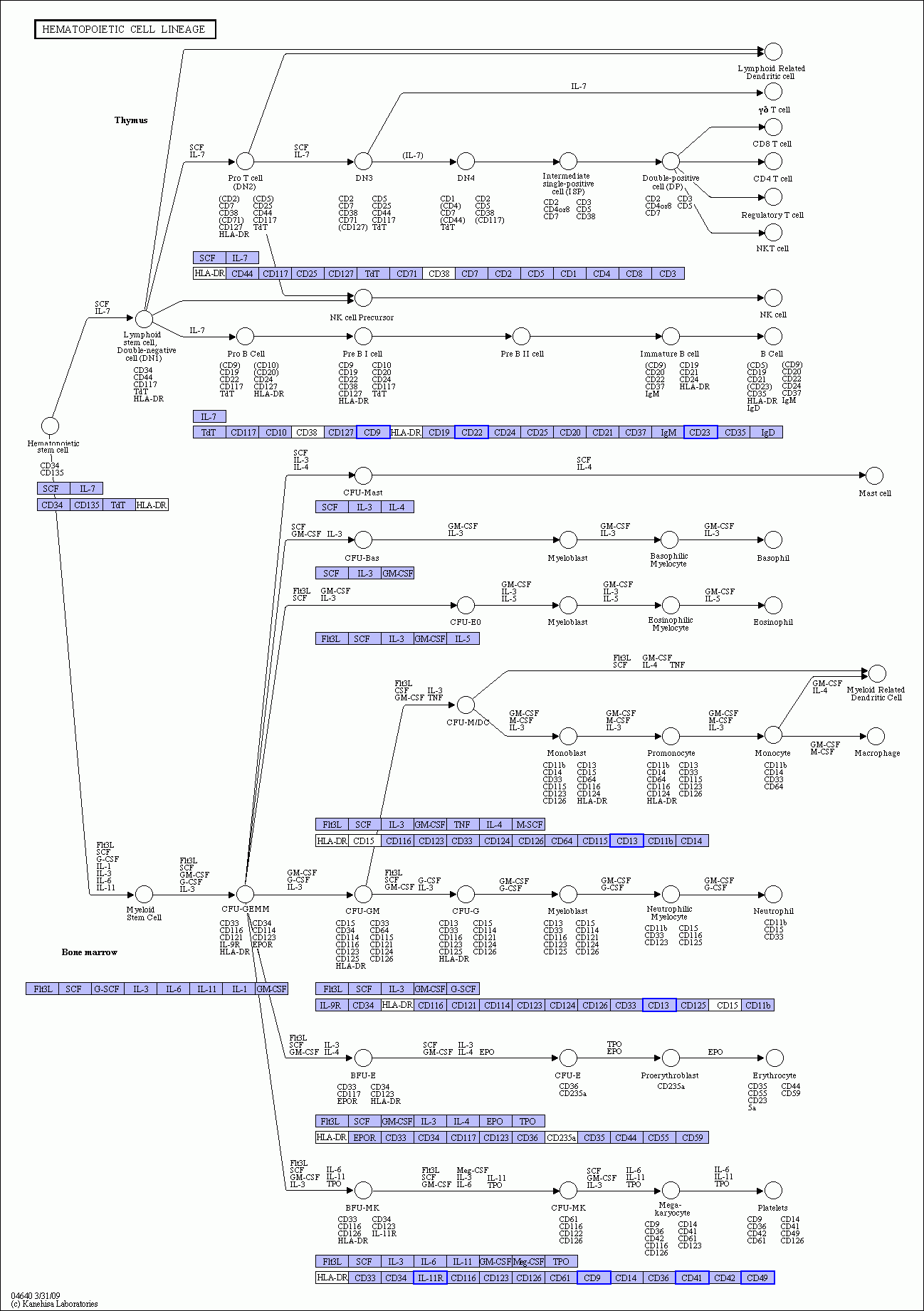

Supplement: Table S4 — KEGG Classification of the unigenes. (ZIP) [file pone.0079516.s004.zip › Kegg/Pathway_Map/ko04640.png]

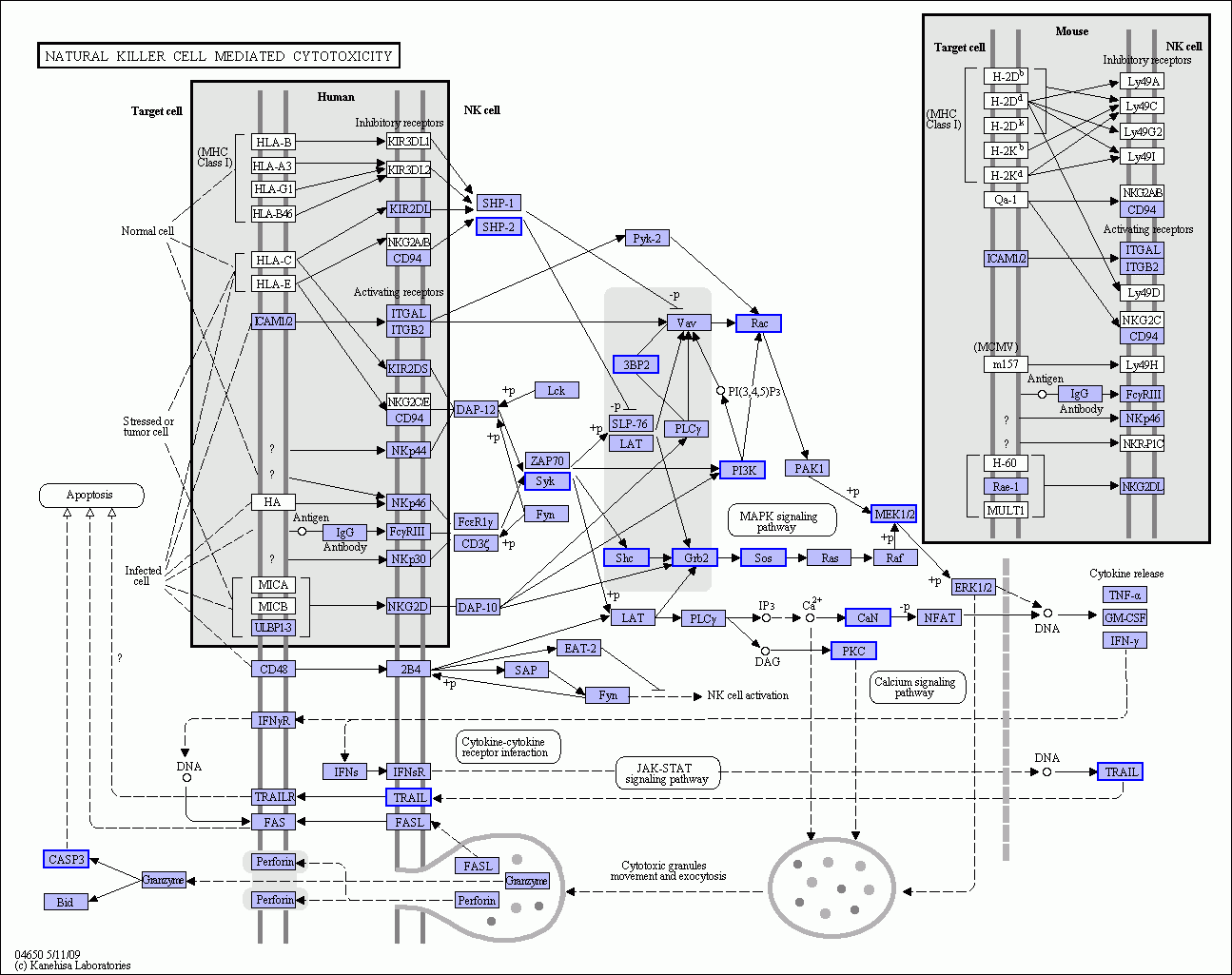

Supplement: Table S4 — KEGG Classification of the unigenes. (ZIP) [file pone.0079516.s004.zip › Kegg/Pathway_Map/ko04650.png]

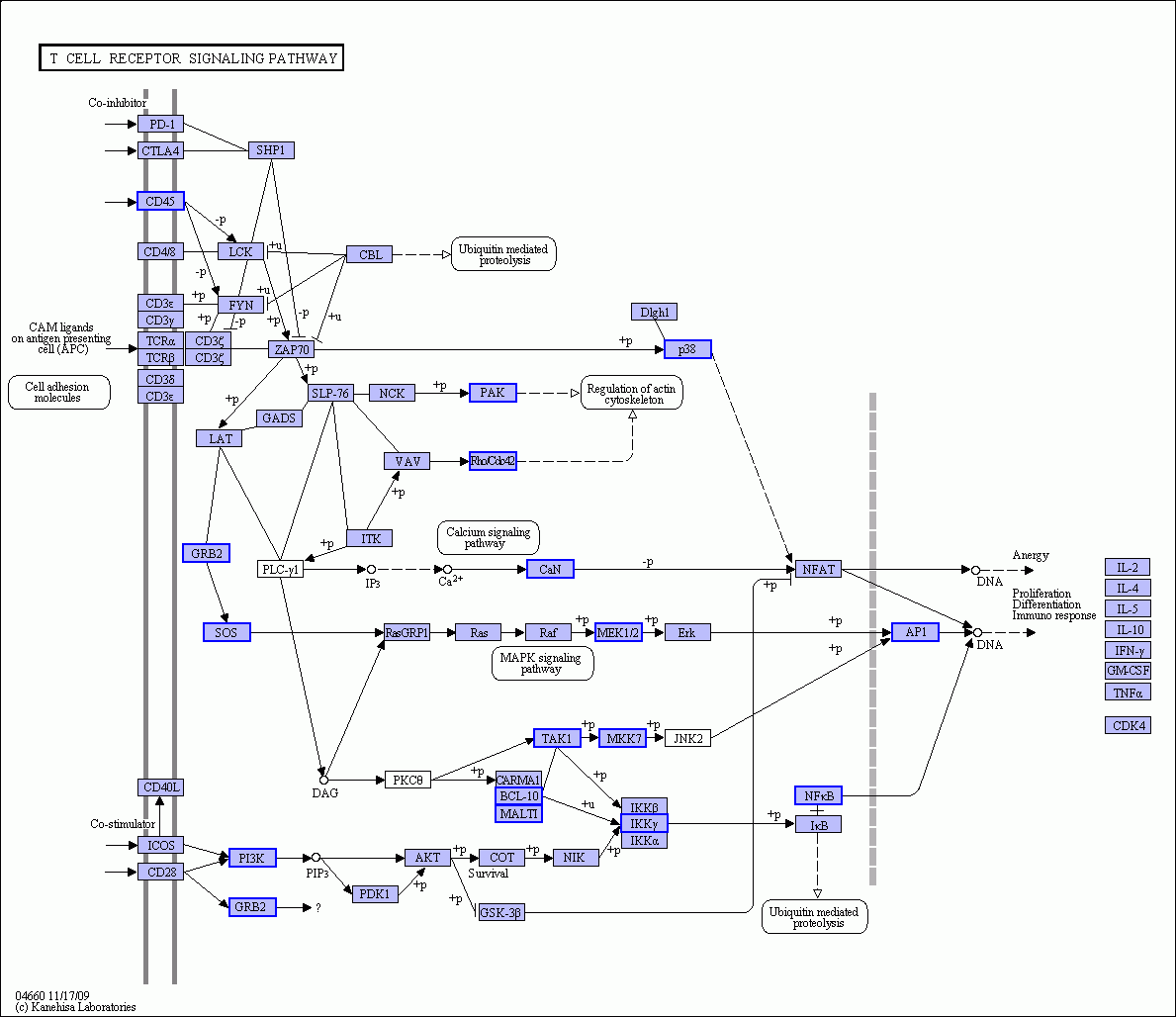

Supplement: Table S4 — KEGG Classification of the unigenes. (ZIP) [file pone.0079516.s004.zip › Kegg/Pathway_Map/ko04660.png]

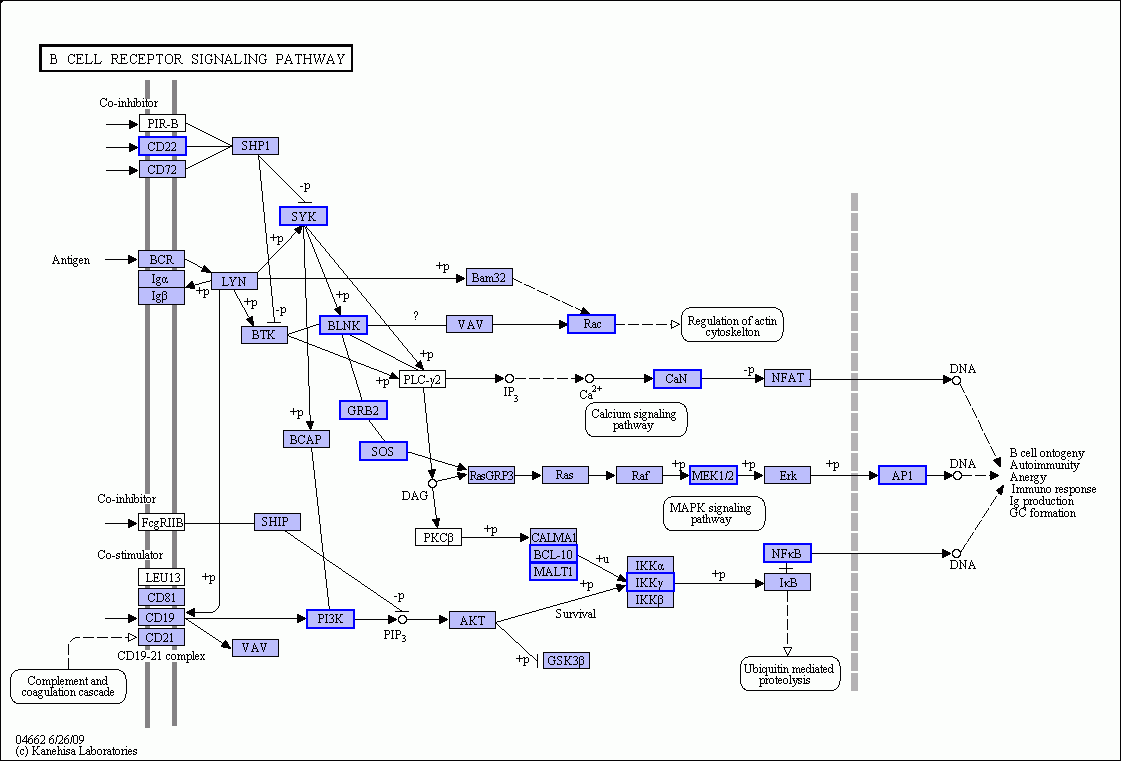

Supplement: Table S4 — KEGG Classification of the unigenes. (ZIP) [file pone.0079516.s004.zip › Kegg/Pathway_Map/ko04662.png]

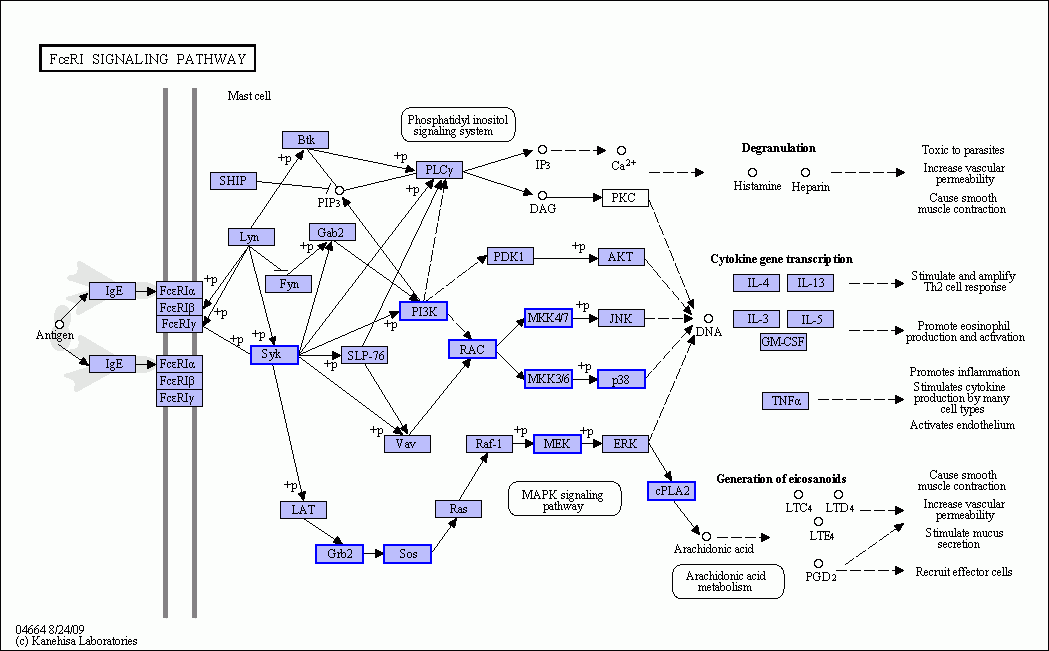

Supplement: Table S4 — KEGG Classification of the unigenes. (ZIP) [file pone.0079516.s004.zip › Kegg/Pathway_Map/ko04664.png]

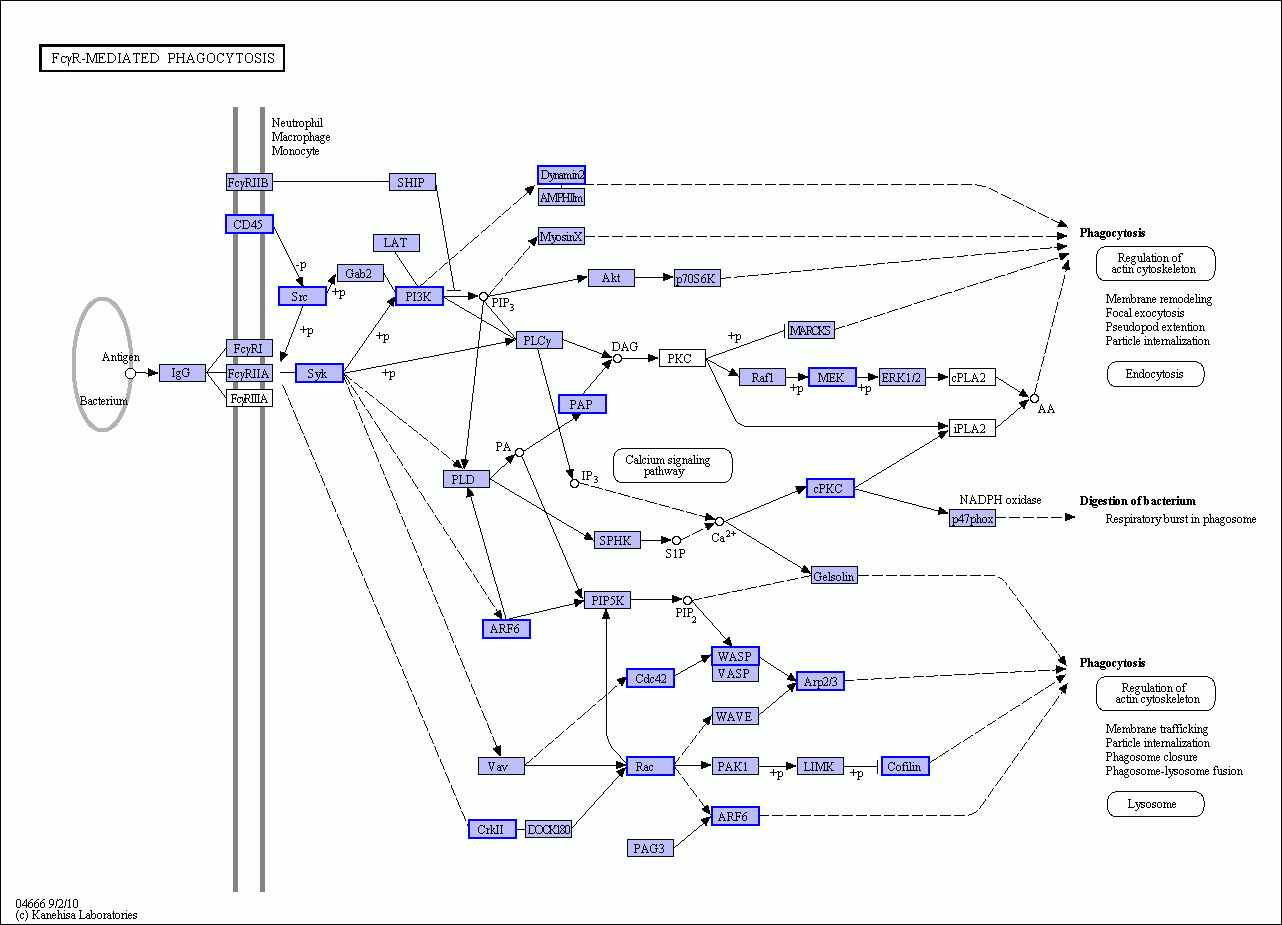

Supplement: Table S4 — KEGG Classification of the unigenes. (ZIP) [file pone.0079516.s004.zip › Kegg/Pathway_Map/ko04666.png]

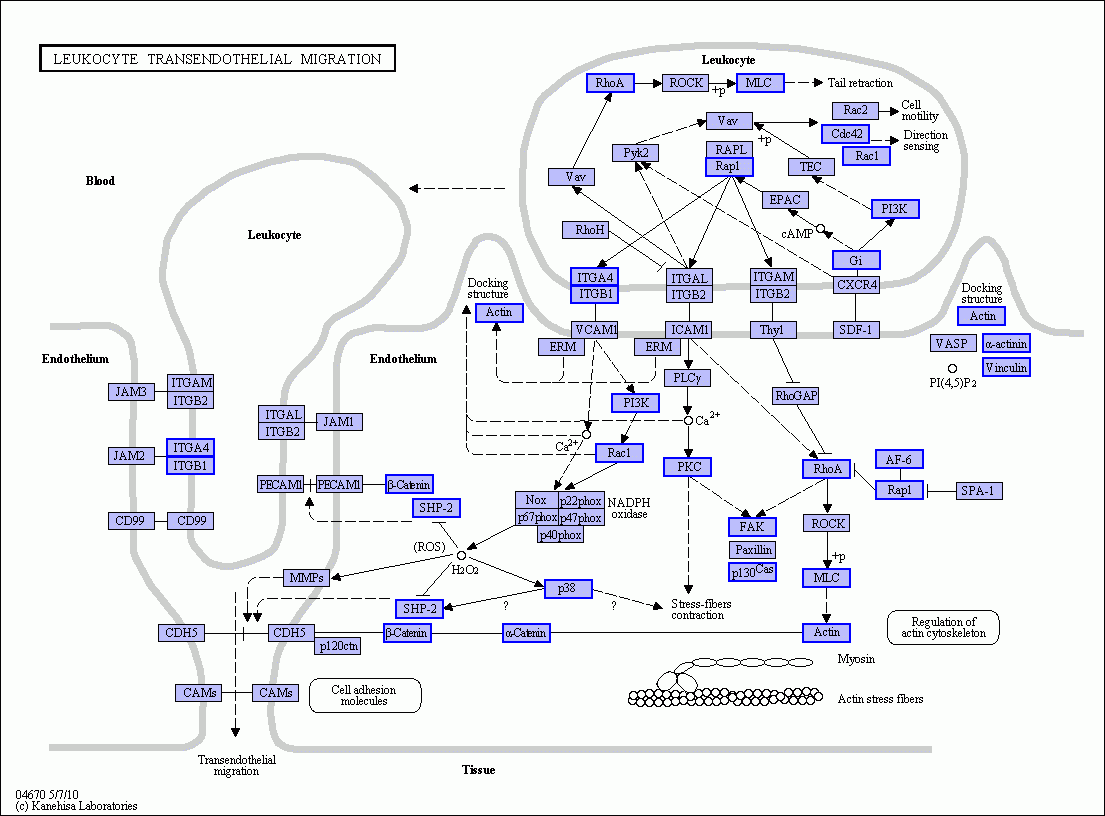

Supplement: Table S4 — KEGG Classification of the unigenes. (ZIP) [file pone.0079516.s004.zip › Kegg/Pathway_Map/ko04670.png]

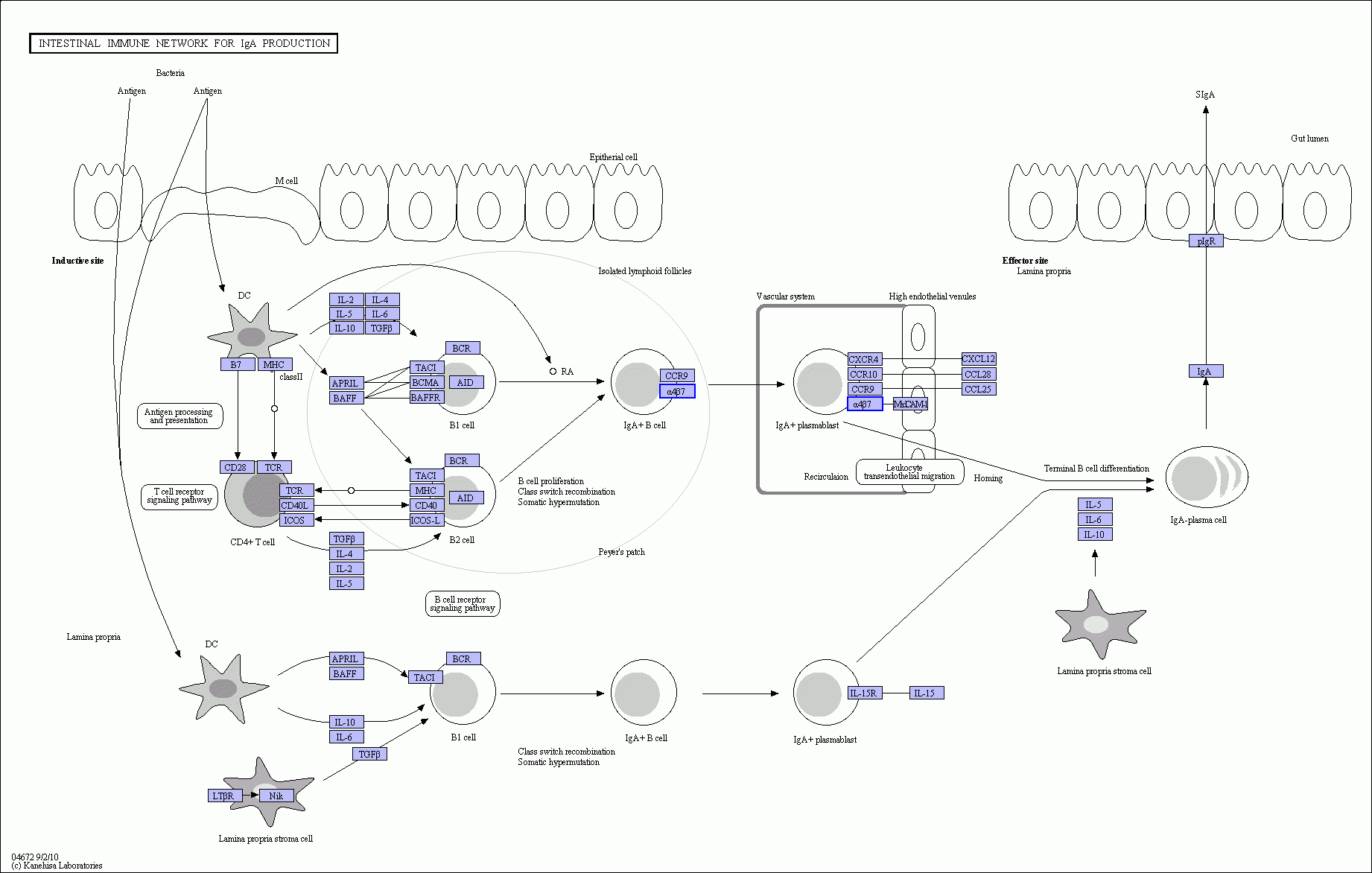

Supplement: Table S4 — KEGG Classification of the unigenes. (ZIP) [file pone.0079516.s004.zip › Kegg/Pathway_Map/ko04672.png]

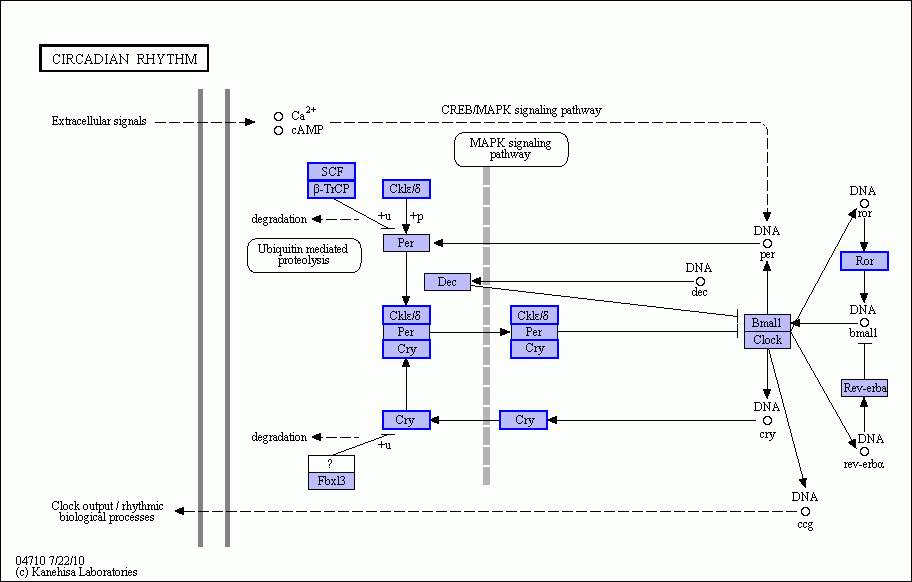

Supplement: Table S4 — KEGG Classification of the unigenes. (ZIP) [file pone.0079516.s004.zip › Kegg/Pathway_Map/ko04710.png]

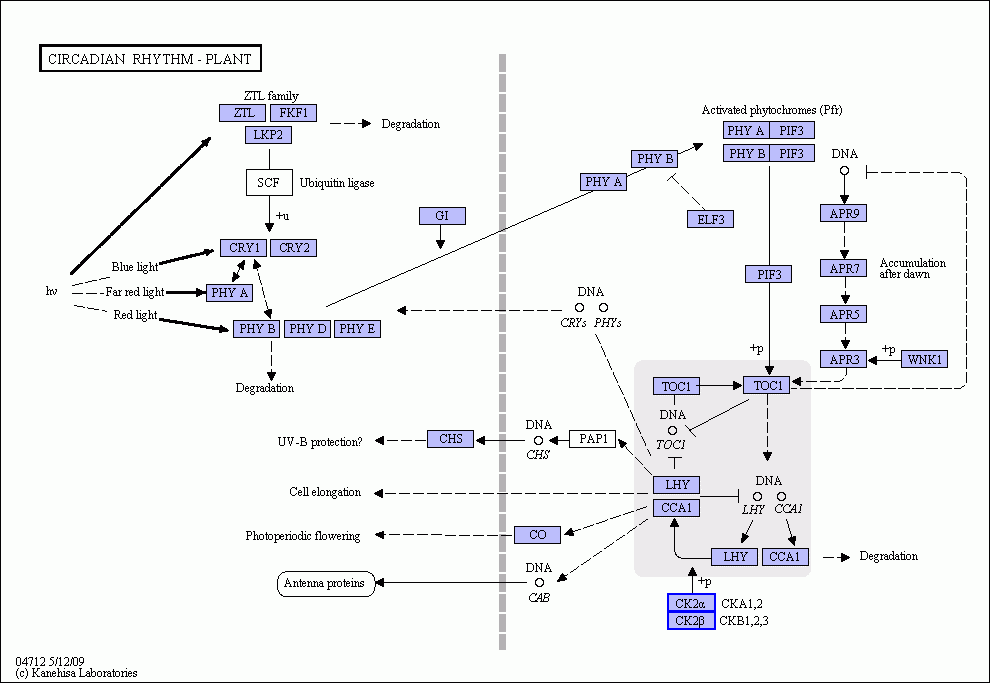

Supplement: Table S4 — KEGG Classification of the unigenes. (ZIP) [file pone.0079516.s004.zip › Kegg/Pathway_Map/ko04712.png]

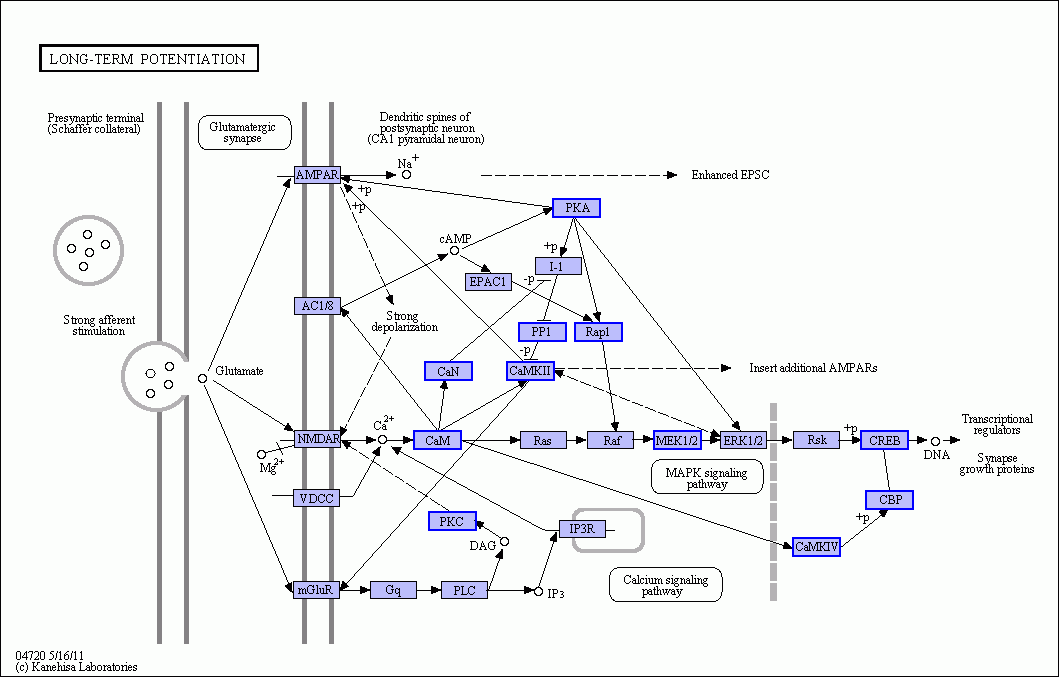

Supplement: Table S4 — KEGG Classification of the unigenes. (ZIP) [file pone.0079516.s004.zip › Kegg/Pathway_Map/ko04720.png]

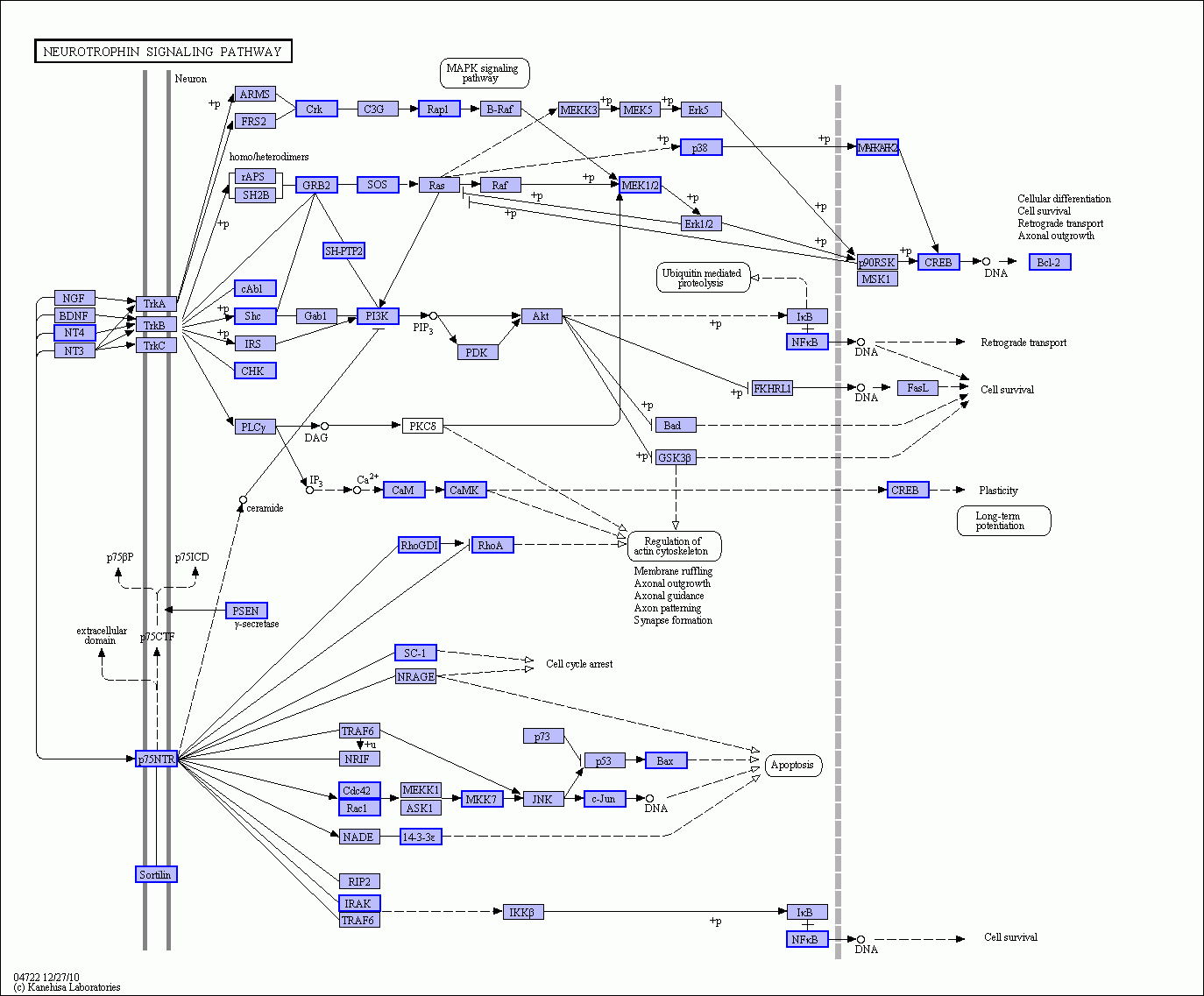

Supplement: Table S4 — KEGG Classification of the unigenes. (ZIP) [file pone.0079516.s004.zip › Kegg/Pathway_Map/ko04722.png]

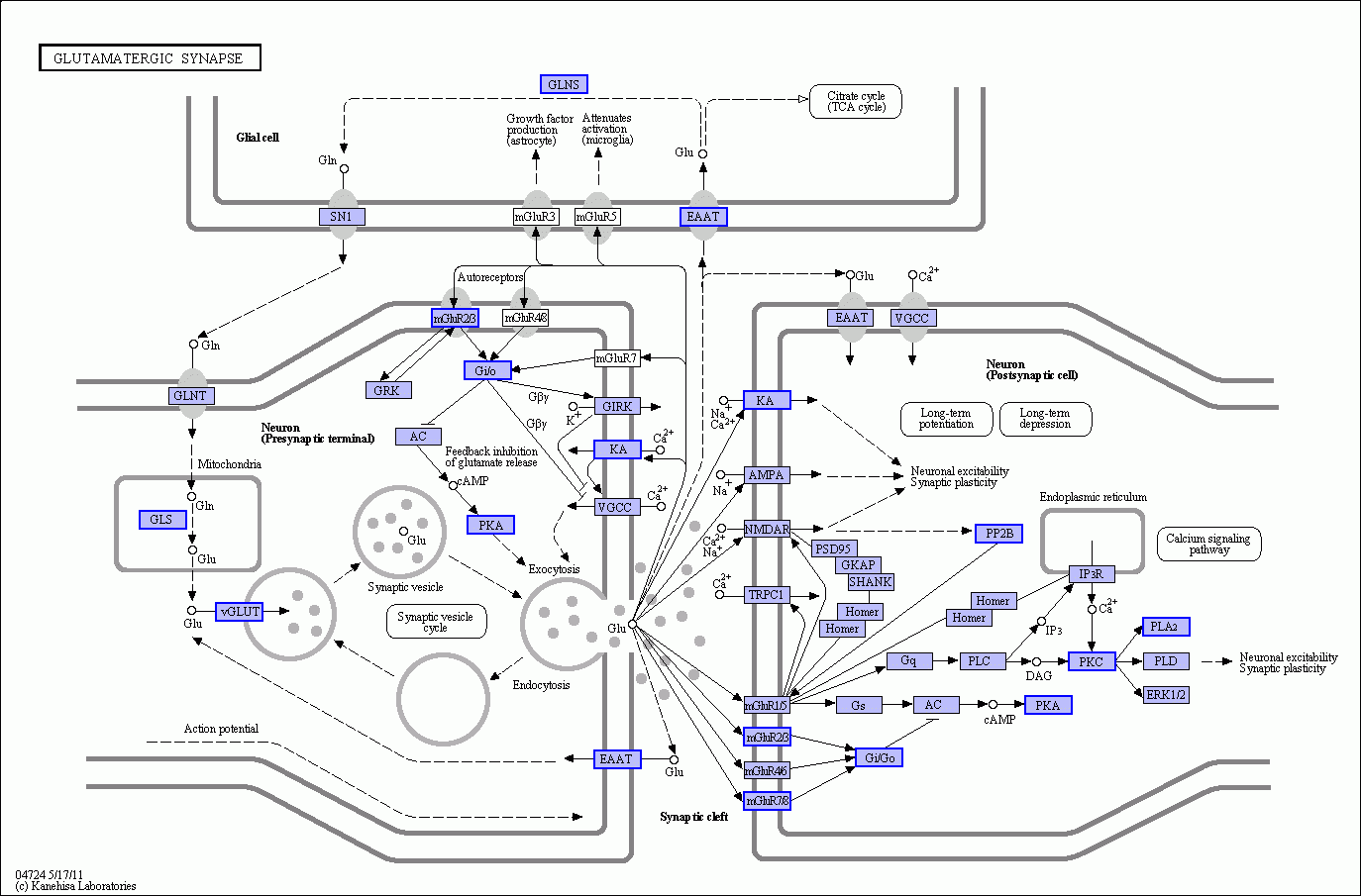

Supplement: Table S4 — KEGG Classification of the unigenes. (ZIP) [file pone.0079516.s004.zip › Kegg/Pathway_Map/ko04724.png]

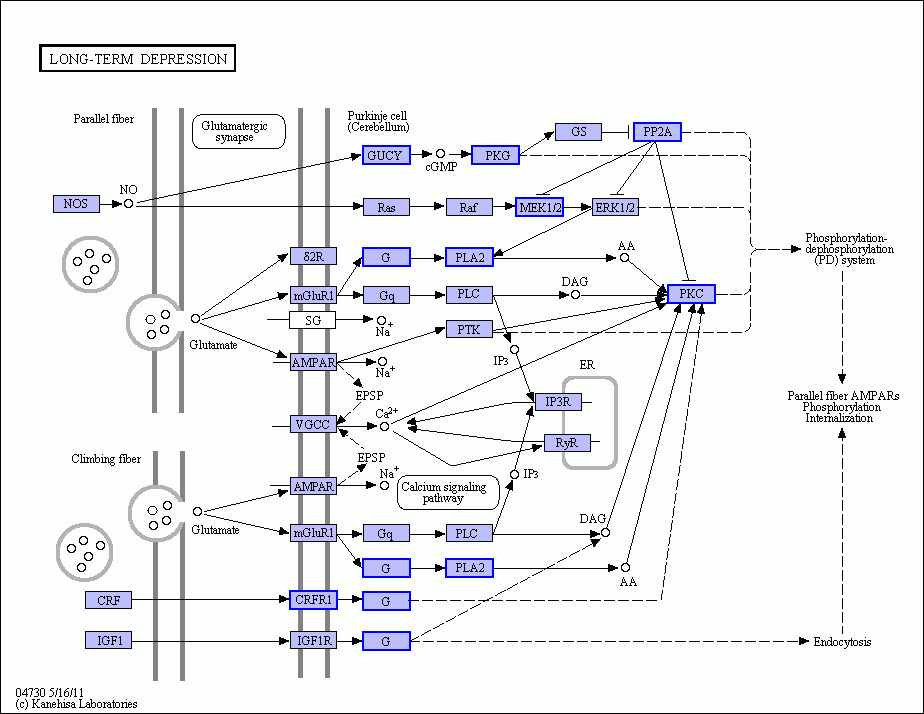

Supplement: Table S4 — KEGG Classification of the unigenes. (ZIP) [file pone.0079516.s004.zip › Kegg/Pathway_Map/ko04730.png]

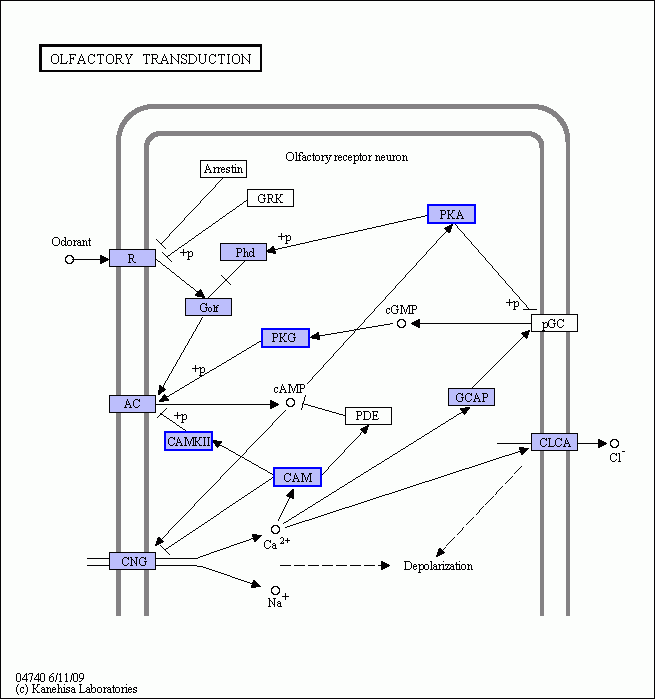

Supplement: Table S4 — KEGG Classification of the unigenes. (ZIP) [file pone.0079516.s004.zip › Kegg/Pathway_Map/ko04740.png]
