# Supplementary figures and images for: Sequencing and De Novo Assembly of the Asian Clam (Corbicula fluminea) Transcriptome Using the Illumina GAIIx Method (part 1 of 2)
Source: PLoS One. 2013 Nov 7;8(11):e79516. doi: 10.1371/journal.pone.0079516 (PMC3820681; doi:10.1371/journal.pone.0079516)

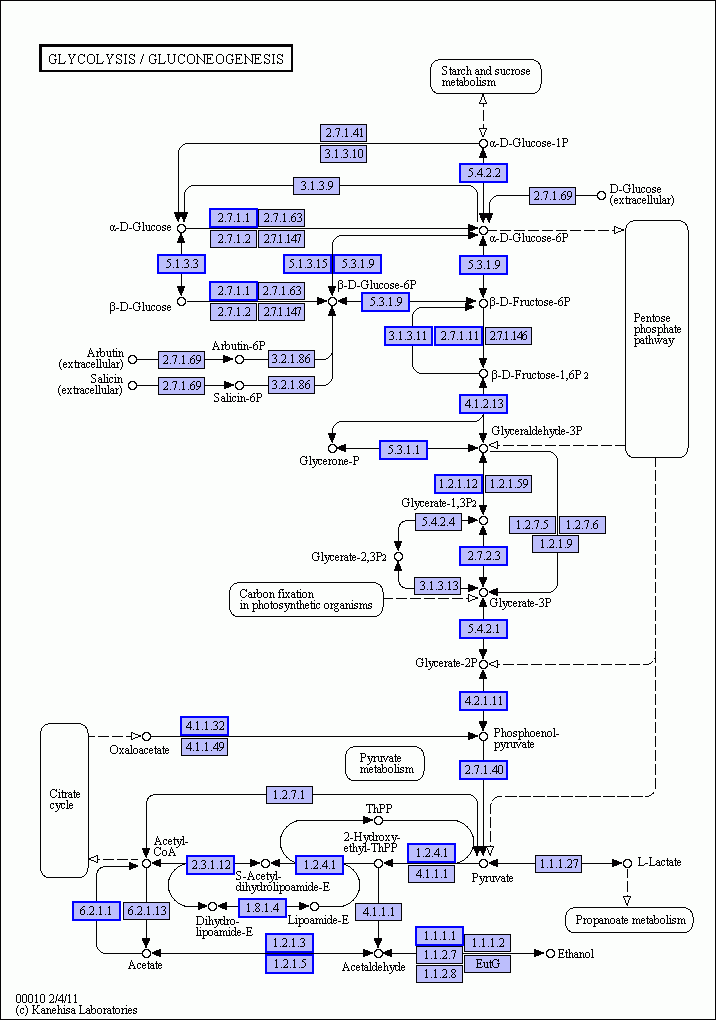

Supplement: Table S4 — KEGG Classification of the unigenes. (ZIP) [file pone.0079516.s004.zip › Kegg/Pathway_Map/ko00010.png]

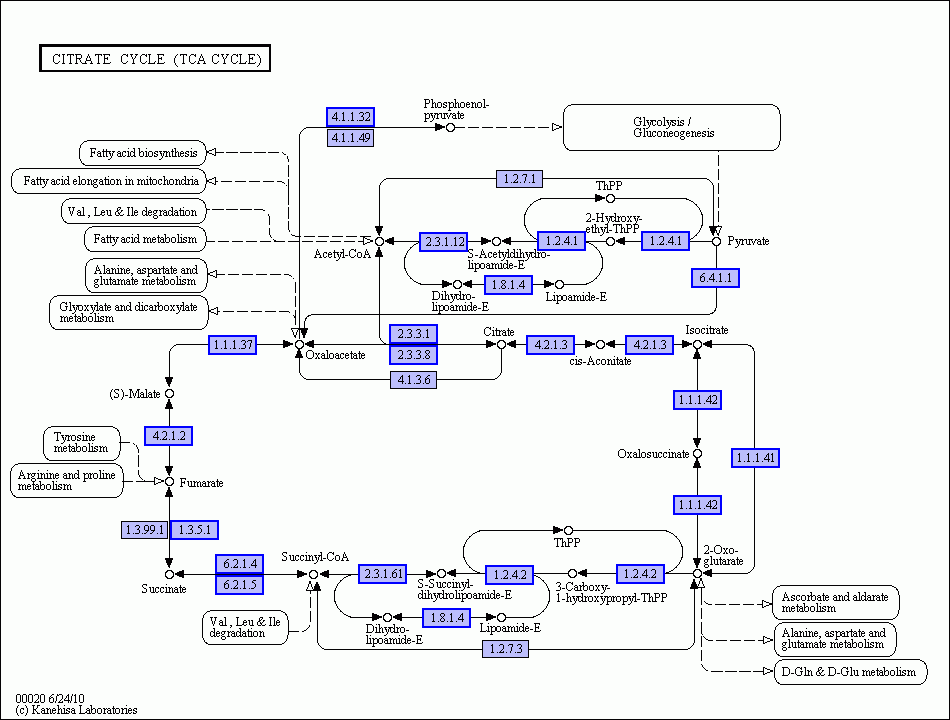

Supplement: Table S4 — KEGG Classification of the unigenes. (ZIP) [file pone.0079516.s004.zip › Kegg/Pathway_Map/ko00020.png]

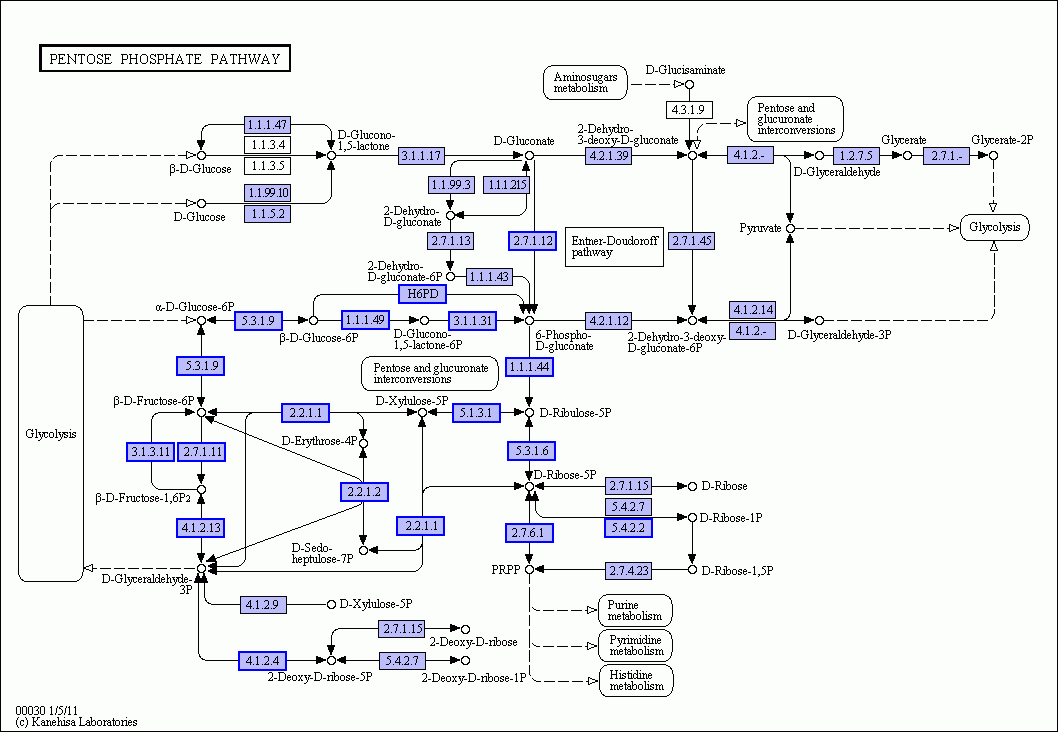

Supplement: Table S4 — KEGG Classification of the unigenes. (ZIP) [file pone.0079516.s004.zip › Kegg/Pathway_Map/ko00030.png]

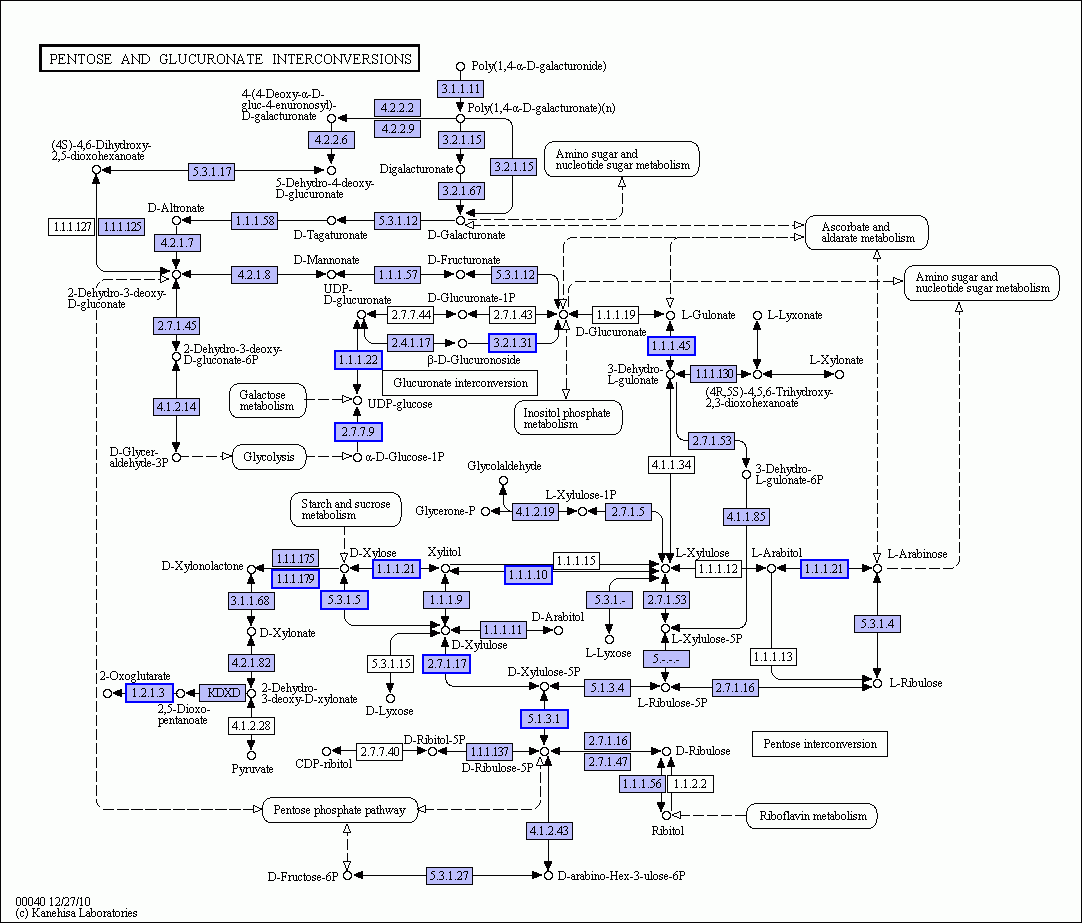

Supplement: Table S4 — KEGG Classification of the unigenes. (ZIP) [file pone.0079516.s004.zip › Kegg/Pathway_Map/ko00040.png]

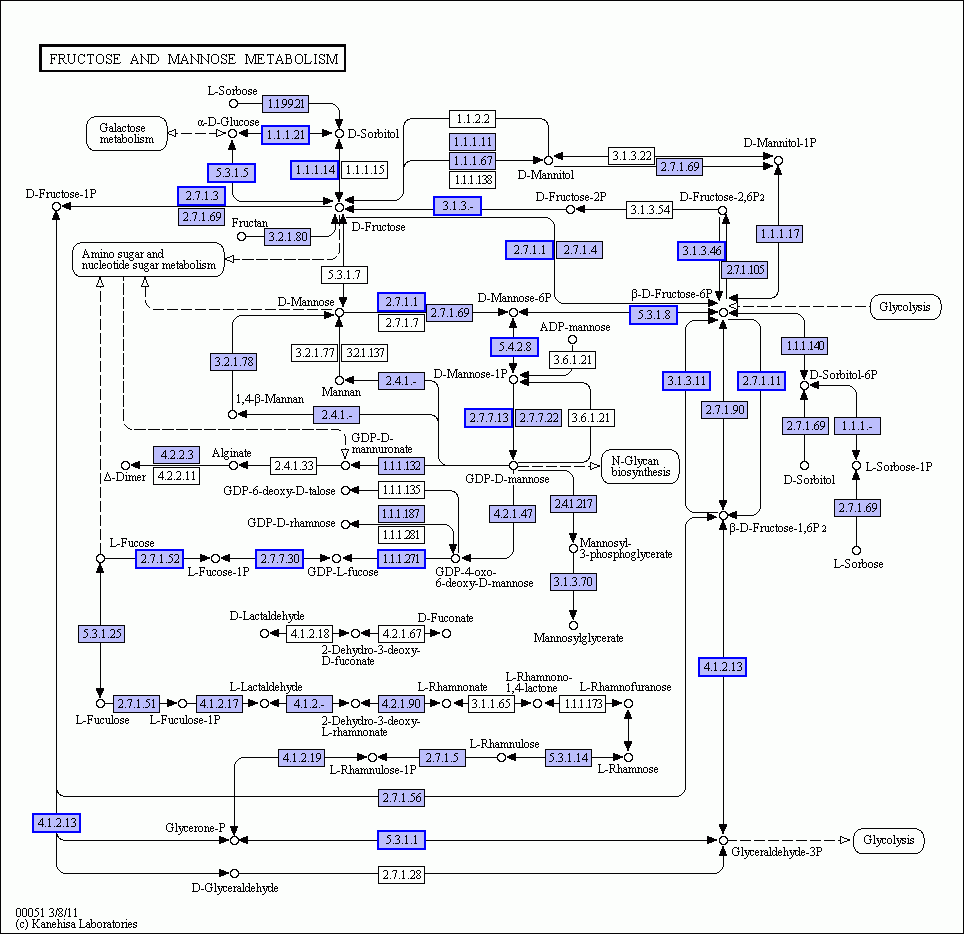

Supplement: Table S4 — KEGG Classification of the unigenes. (ZIP) [file pone.0079516.s004.zip › Kegg/Pathway_Map/ko00051.png]

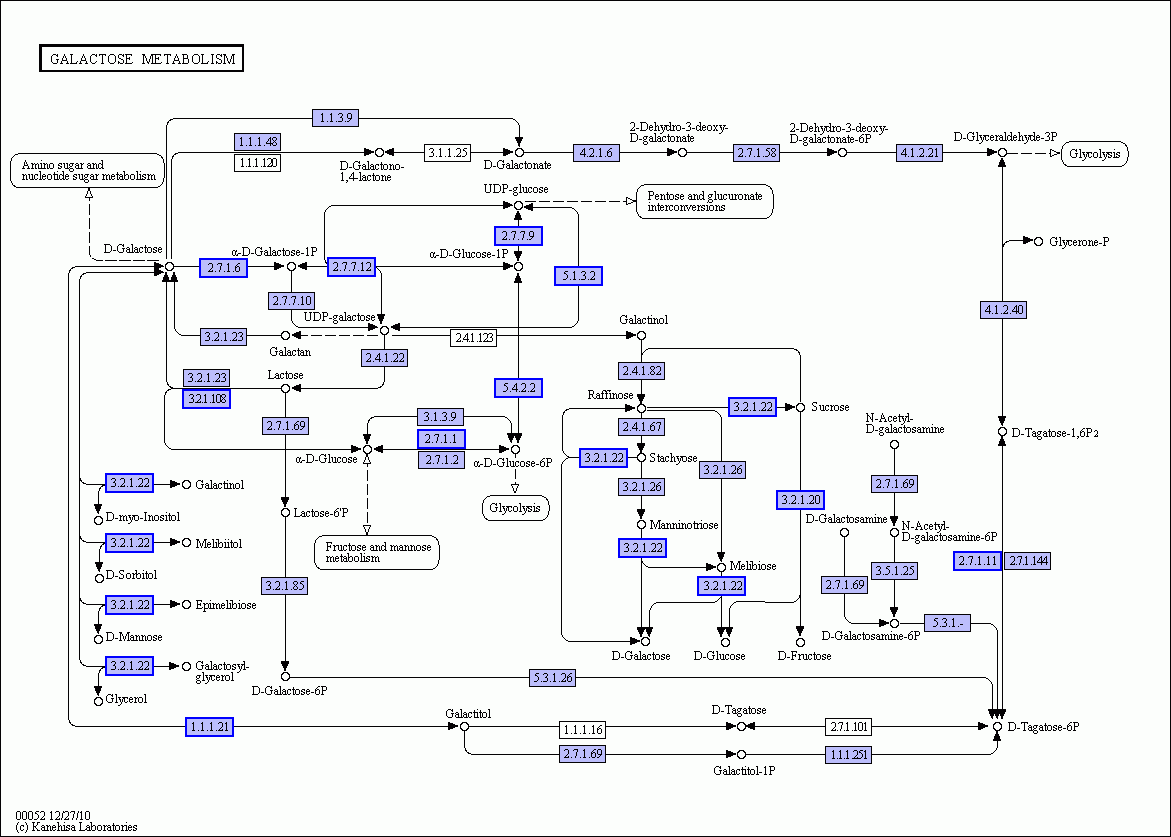

Supplement: Table S4 — KEGG Classification of the unigenes. (ZIP) [file pone.0079516.s004.zip › Kegg/Pathway_Map/ko00052.png]

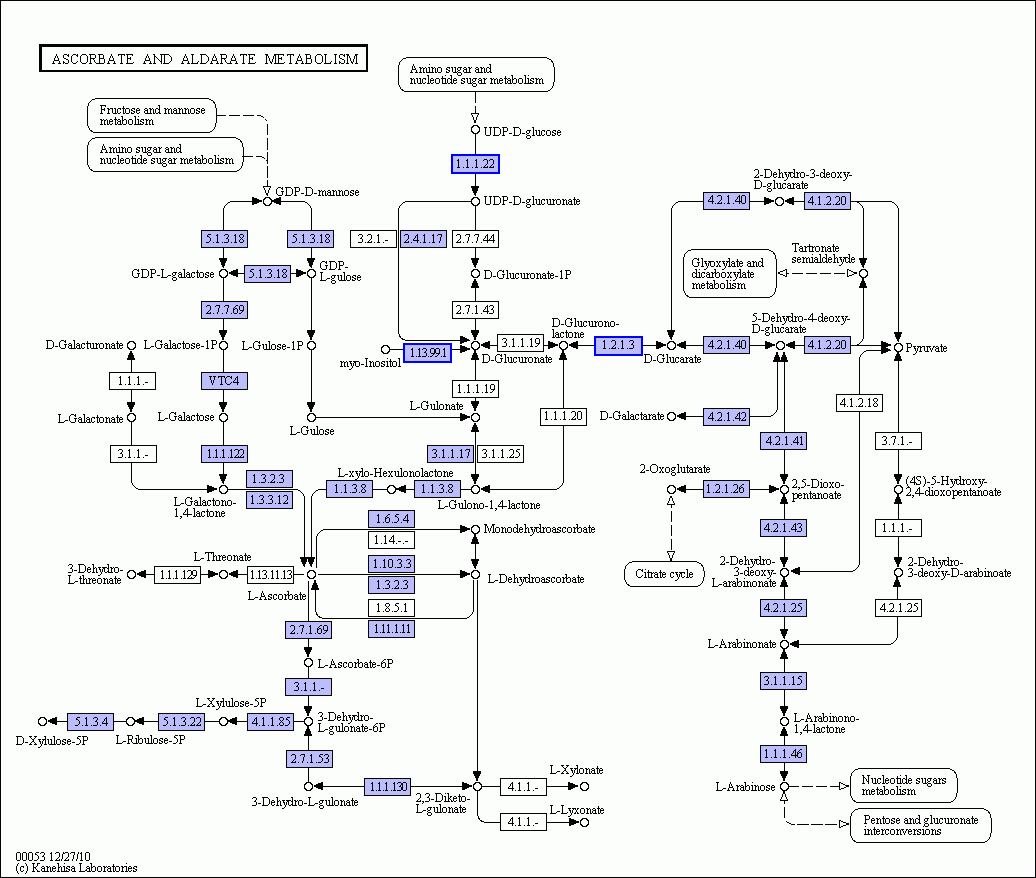

Supplement: Table S4 — KEGG Classification of the unigenes. (ZIP) [file pone.0079516.s004.zip › Kegg/Pathway_Map/ko00053.png]

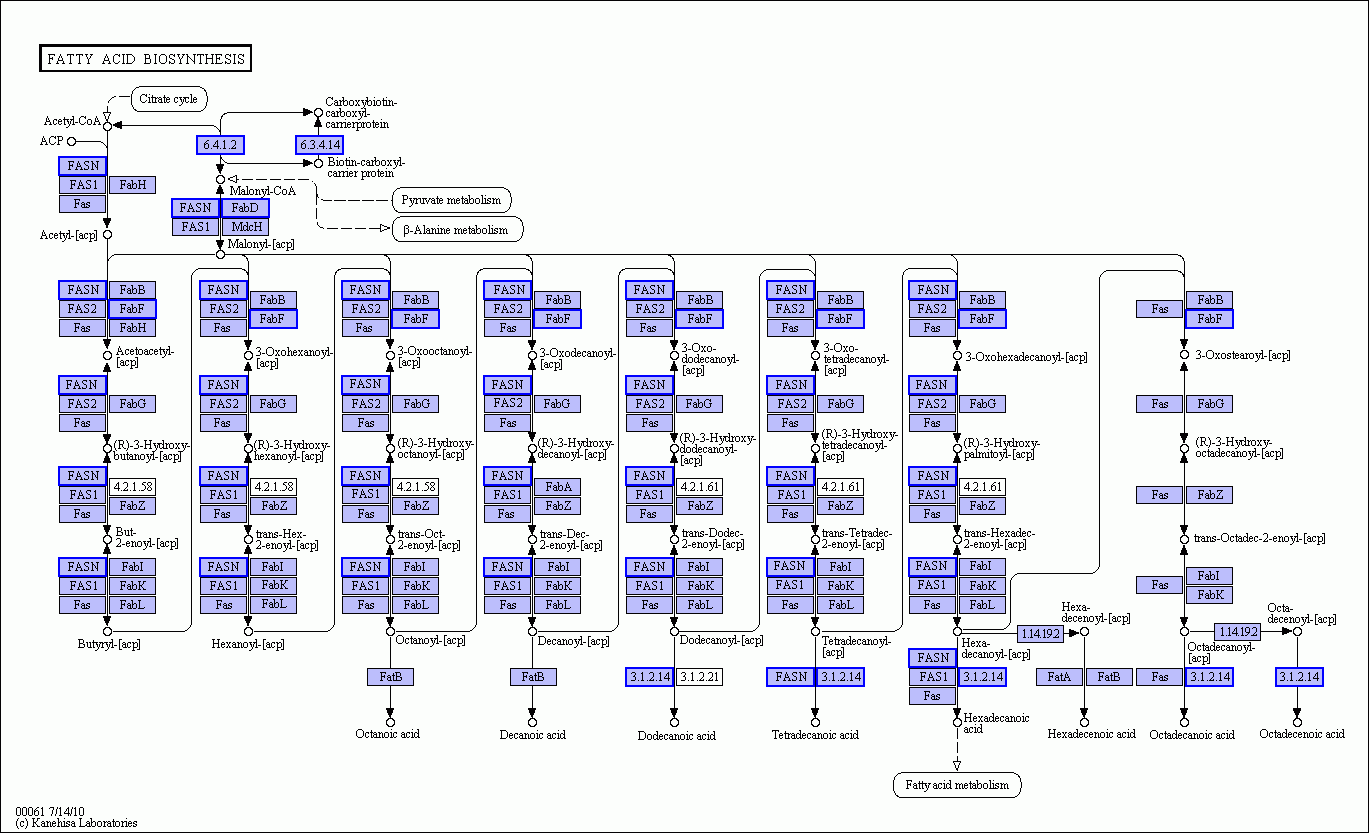

Supplement: Table S4 — KEGG Classification of the unigenes. (ZIP) [file pone.0079516.s004.zip › Kegg/Pathway_Map/ko00061.png]

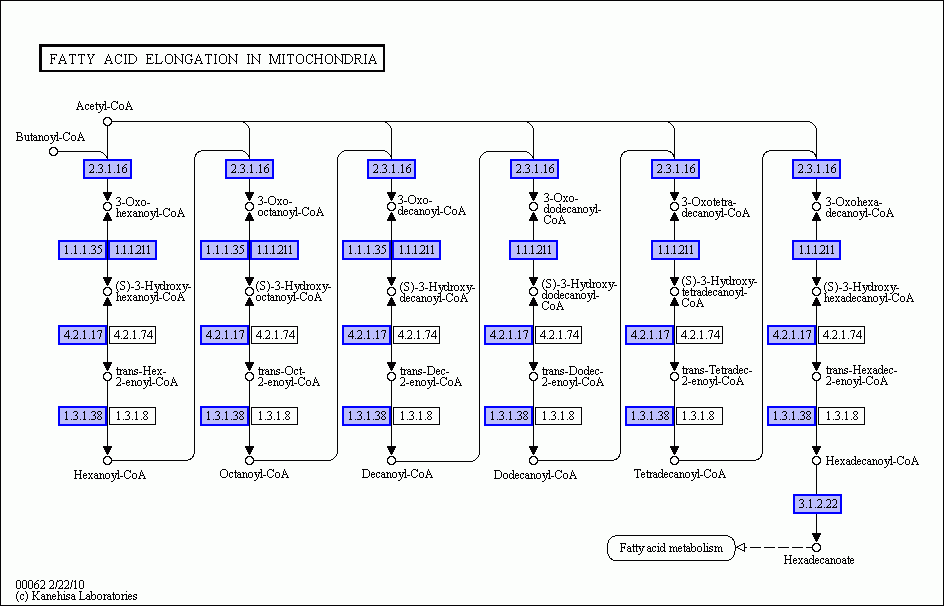

Supplement: Table S4 — KEGG Classification of the unigenes. (ZIP) [file pone.0079516.s004.zip › Kegg/Pathway_Map/ko00062.png]

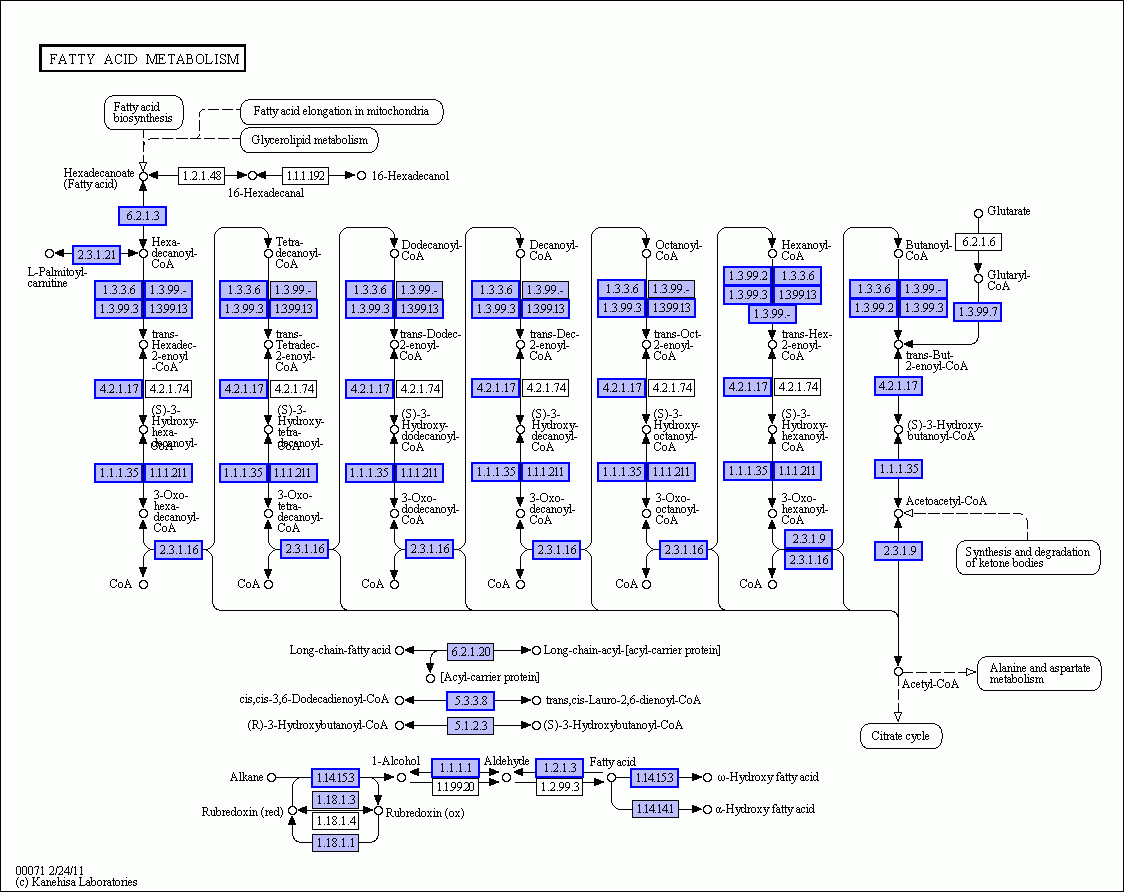

Supplement: Table S4 — KEGG Classification of the unigenes. (ZIP) [file pone.0079516.s004.zip › Kegg/Pathway_Map/ko00071.png]

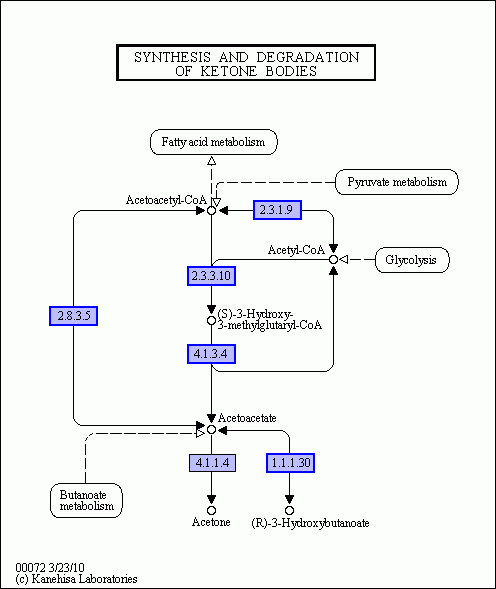

Supplement: Table S4 — KEGG Classification of the unigenes. (ZIP) [file pone.0079516.s004.zip › Kegg/Pathway_Map/ko00072.png]

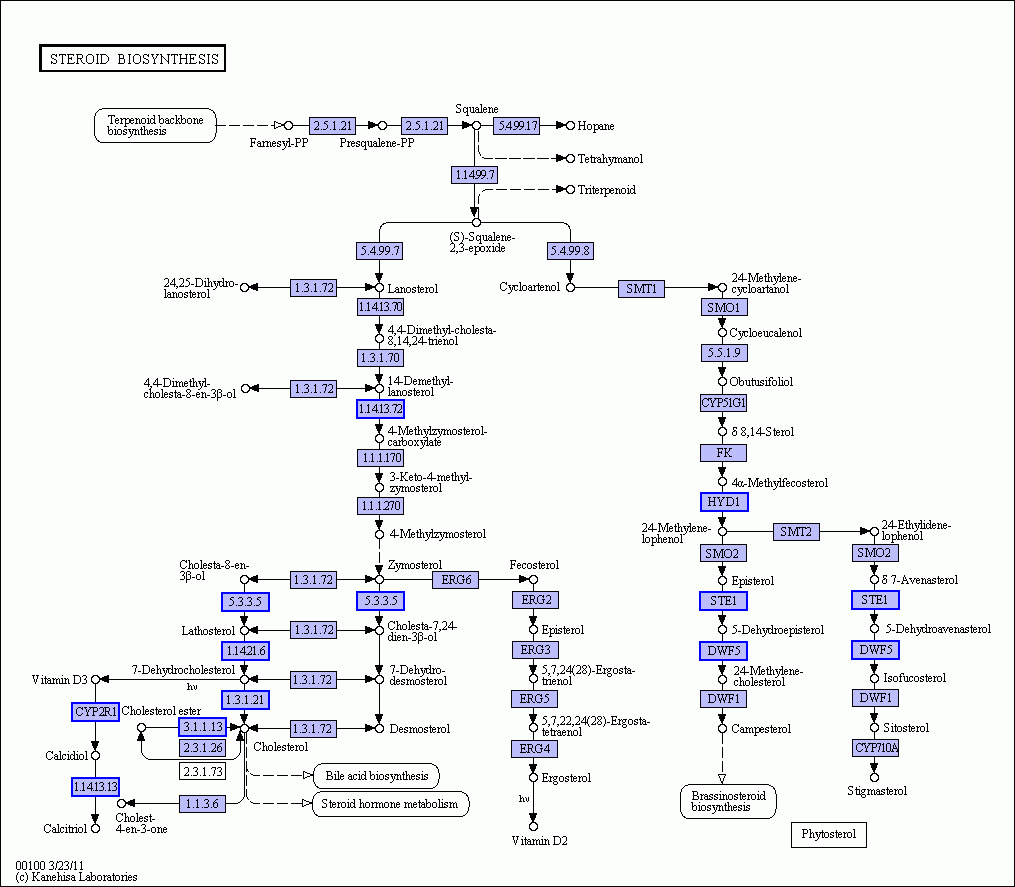

Supplement: Table S4 — KEGG Classification of the unigenes. (ZIP) [file pone.0079516.s004.zip › Kegg/Pathway_Map/ko00100.png]

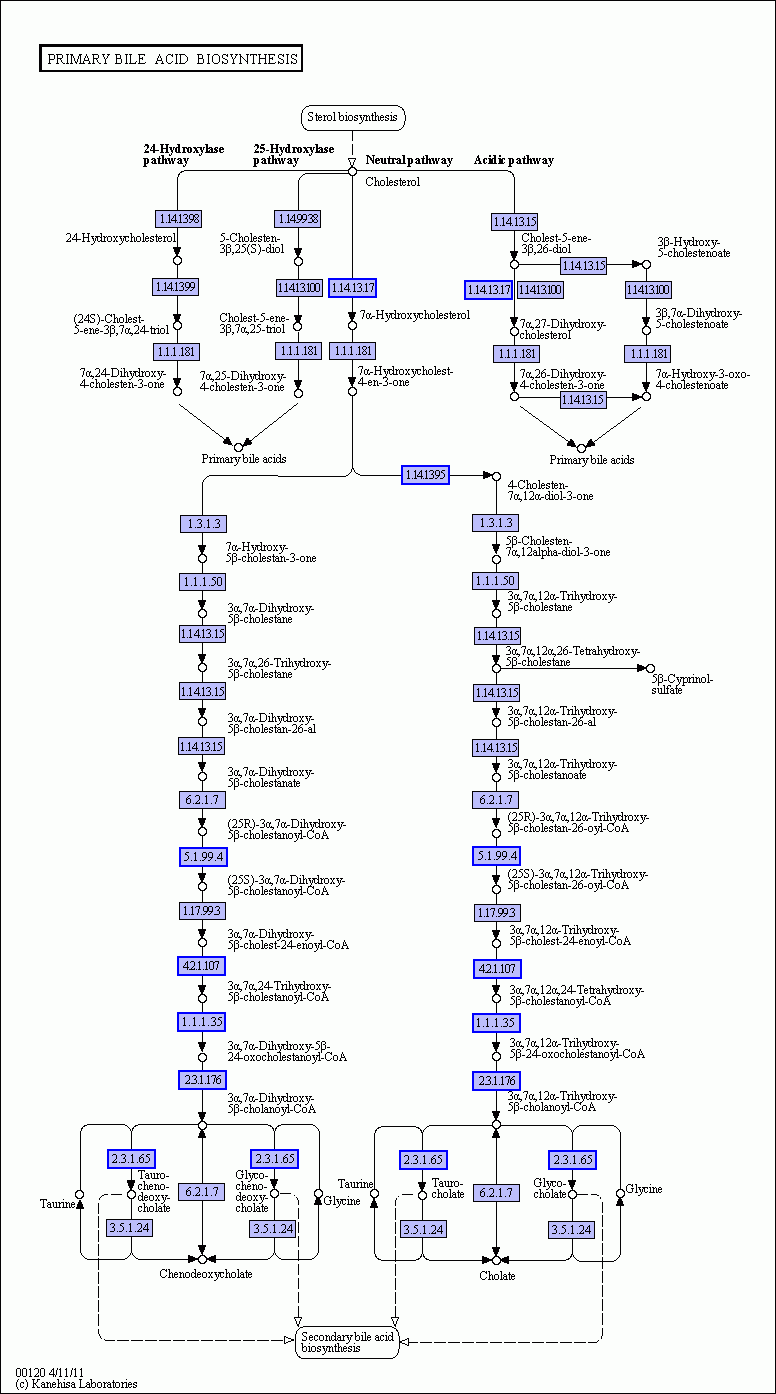

Supplement: Table S4 — KEGG Classification of the unigenes. (ZIP) [file pone.0079516.s004.zip › Kegg/Pathway_Map/ko00120.png]

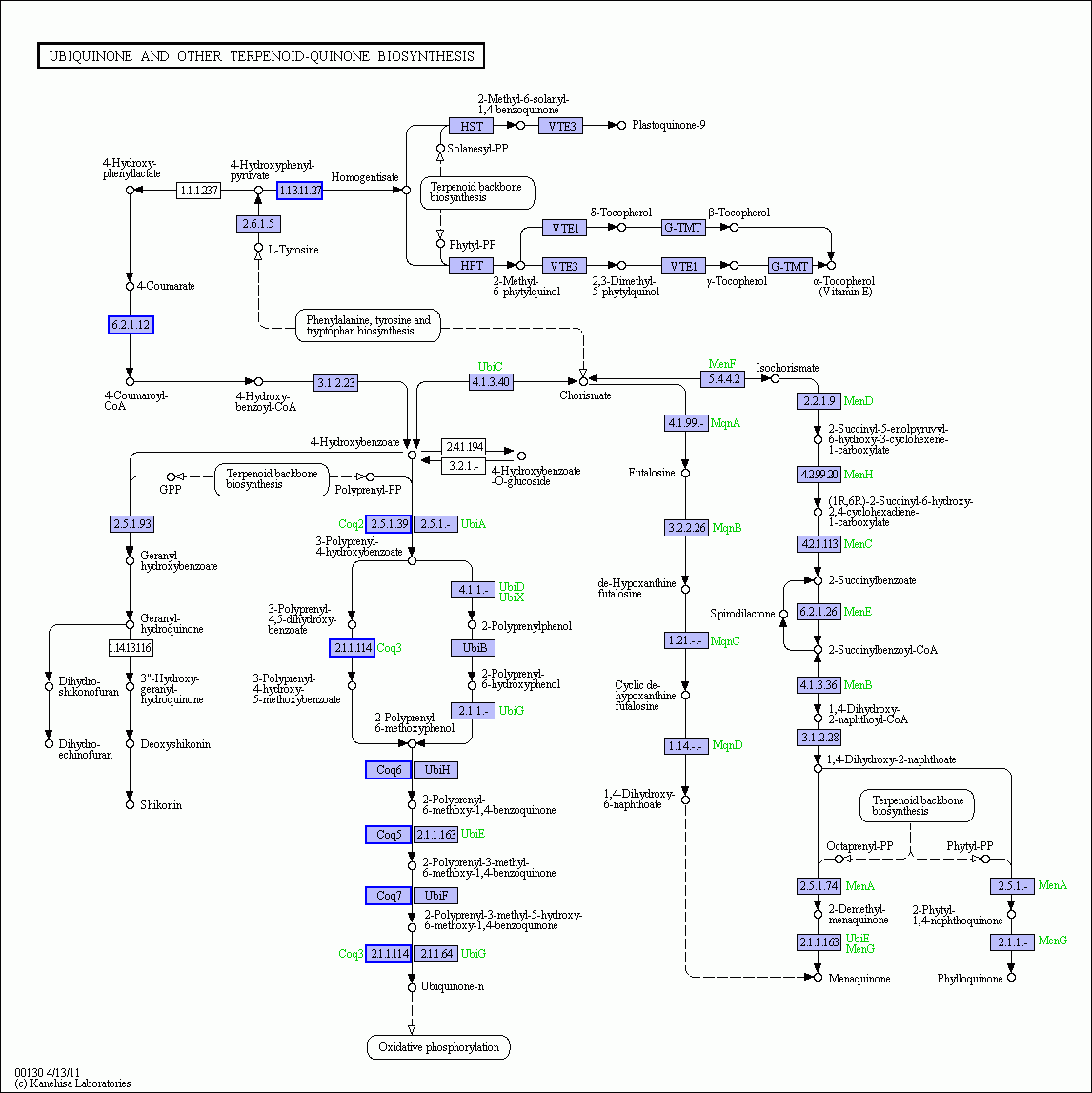

Supplement: Table S4 — KEGG Classification of the unigenes. (ZIP) [file pone.0079516.s004.zip › Kegg/Pathway_Map/ko00130.png]

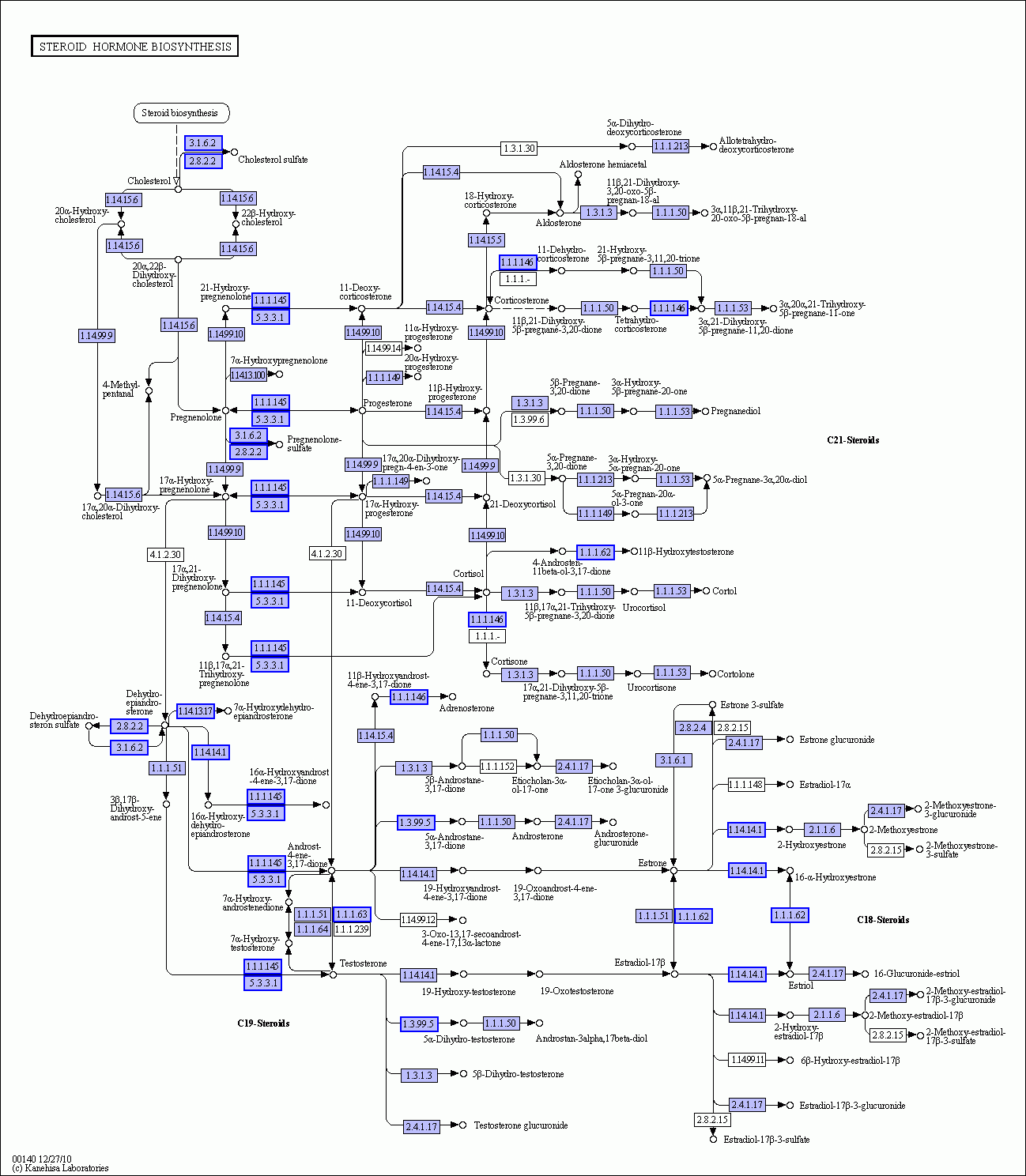

Supplement: Table S4 — KEGG Classification of the unigenes. (ZIP) [file pone.0079516.s004.zip › Kegg/Pathway_Map/ko00140.png]

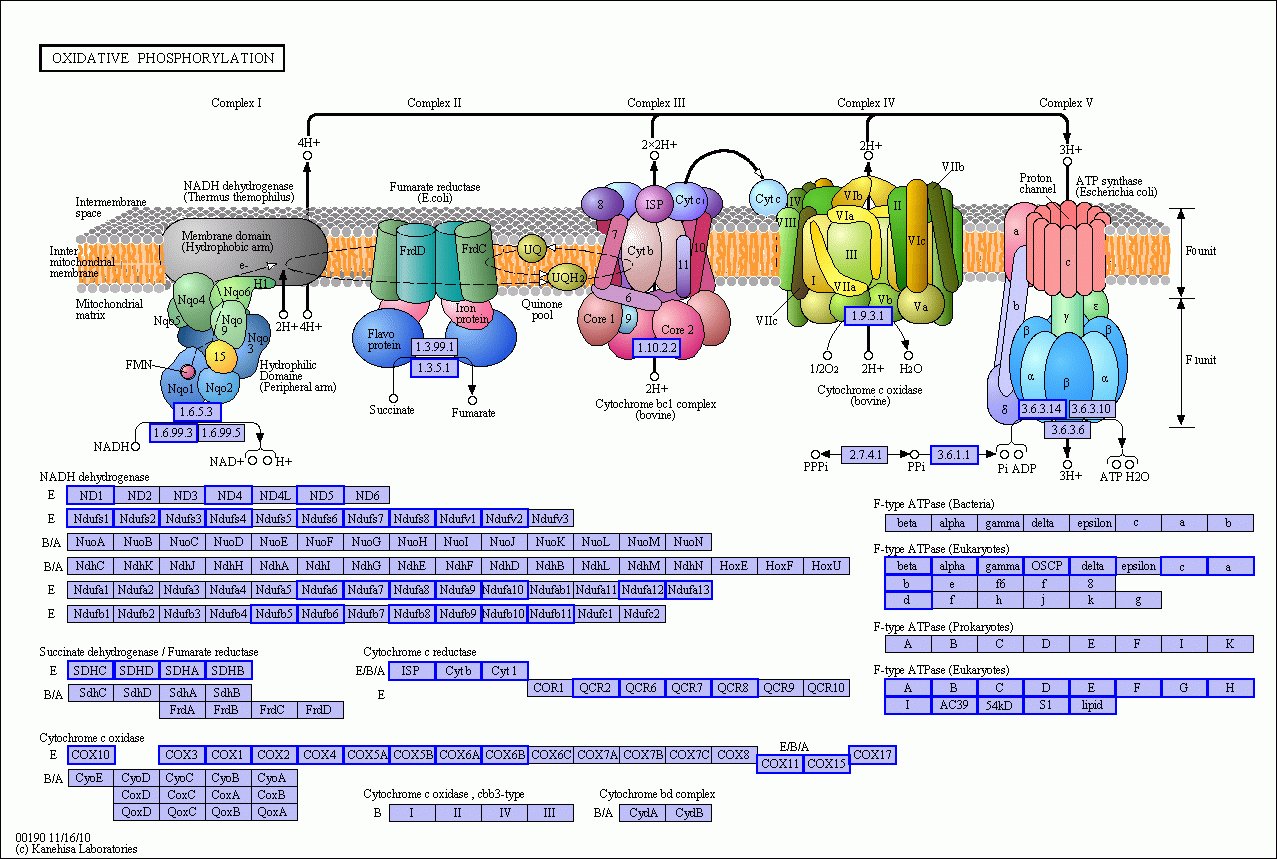

Supplement: Table S4 — KEGG Classification of the unigenes. (ZIP) [file pone.0079516.s004.zip › Kegg/Pathway_Map/ko00190.png]

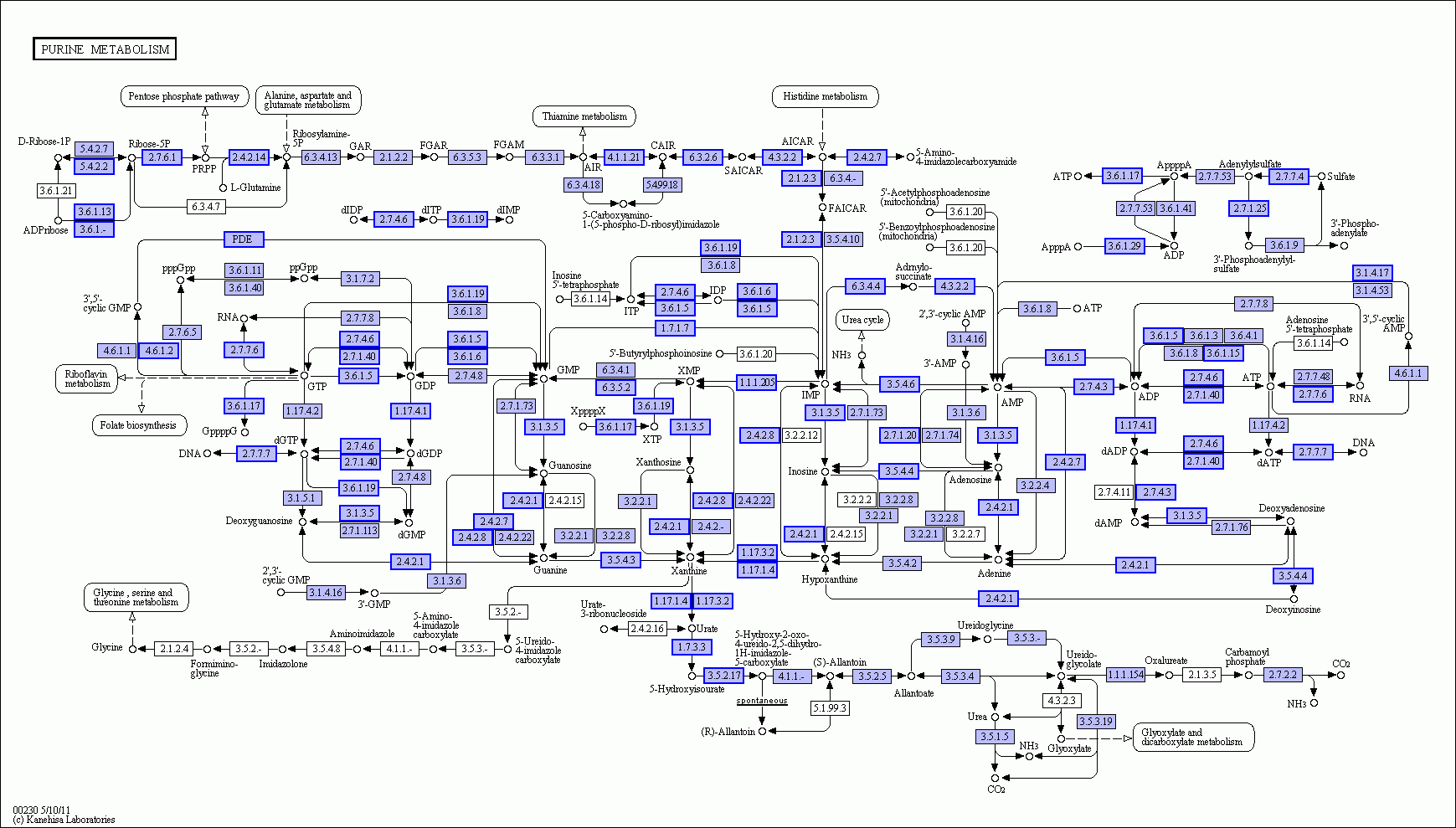

Supplement: Table S4 — KEGG Classification of the unigenes. (ZIP) [file pone.0079516.s004.zip › Kegg/Pathway_Map/ko00230.png]

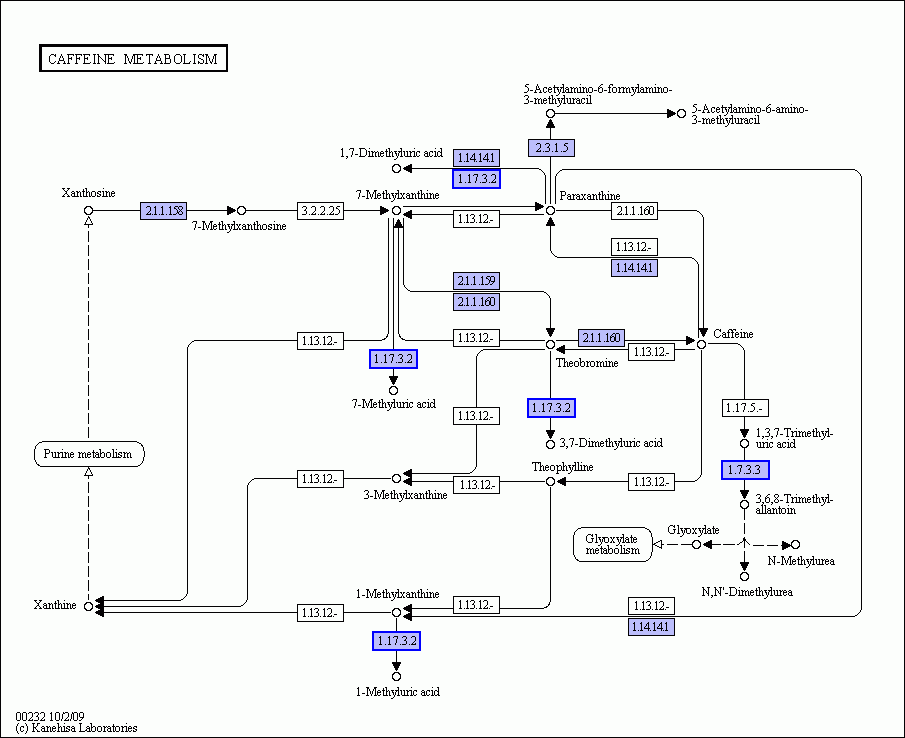

Supplement: Table S4 — KEGG Classification of the unigenes. (ZIP) [file pone.0079516.s004.zip › Kegg/Pathway_Map/ko00232.png]

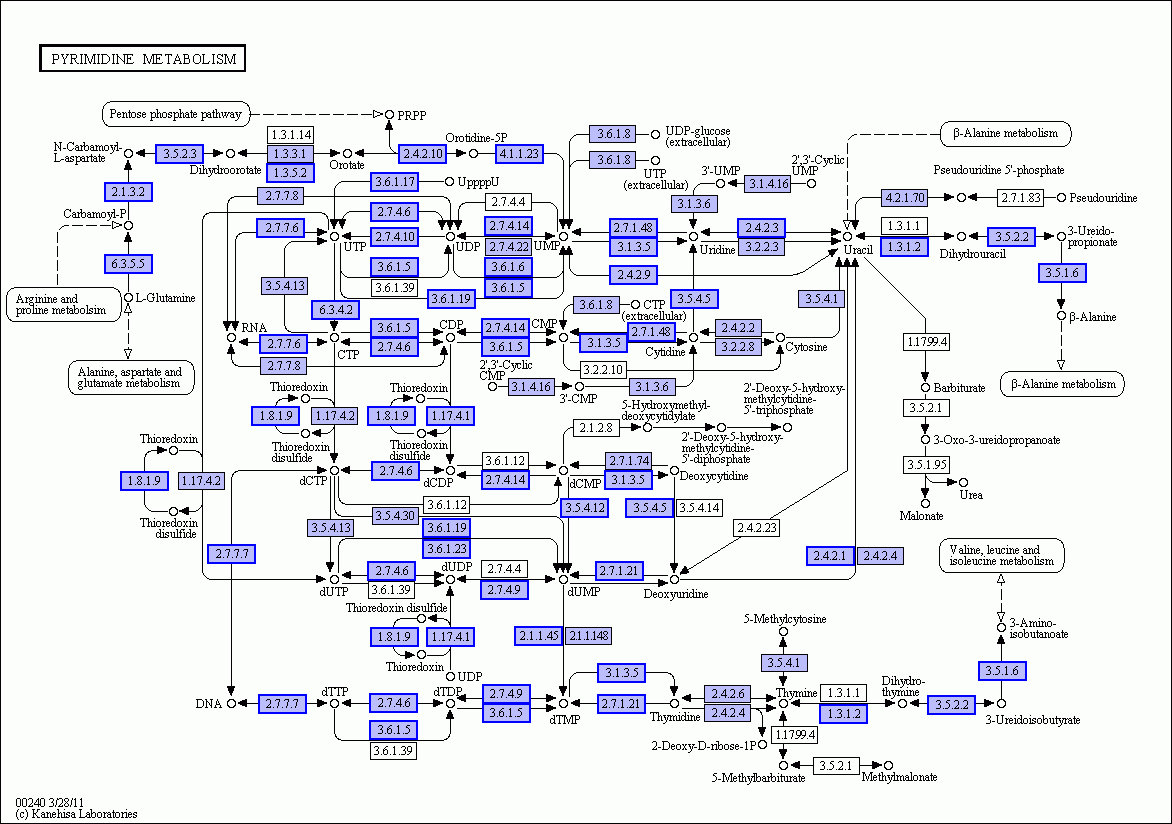

Supplement: Table S4 — KEGG Classification of the unigenes. (ZIP) [file pone.0079516.s004.zip › Kegg/Pathway_Map/ko00240.png]

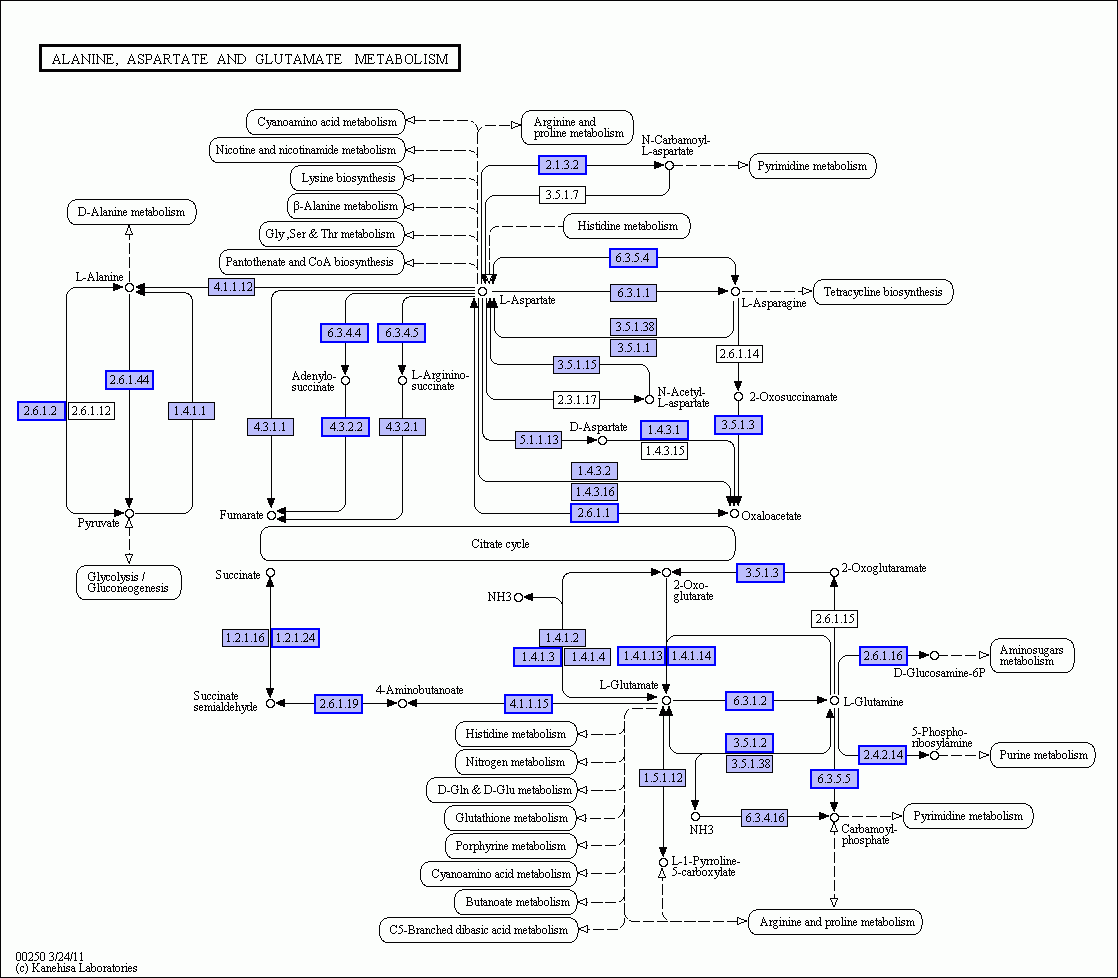

Supplement: Table S4 — KEGG Classification of the unigenes. (ZIP) [file pone.0079516.s004.zip › Kegg/Pathway_Map/ko00250.png]

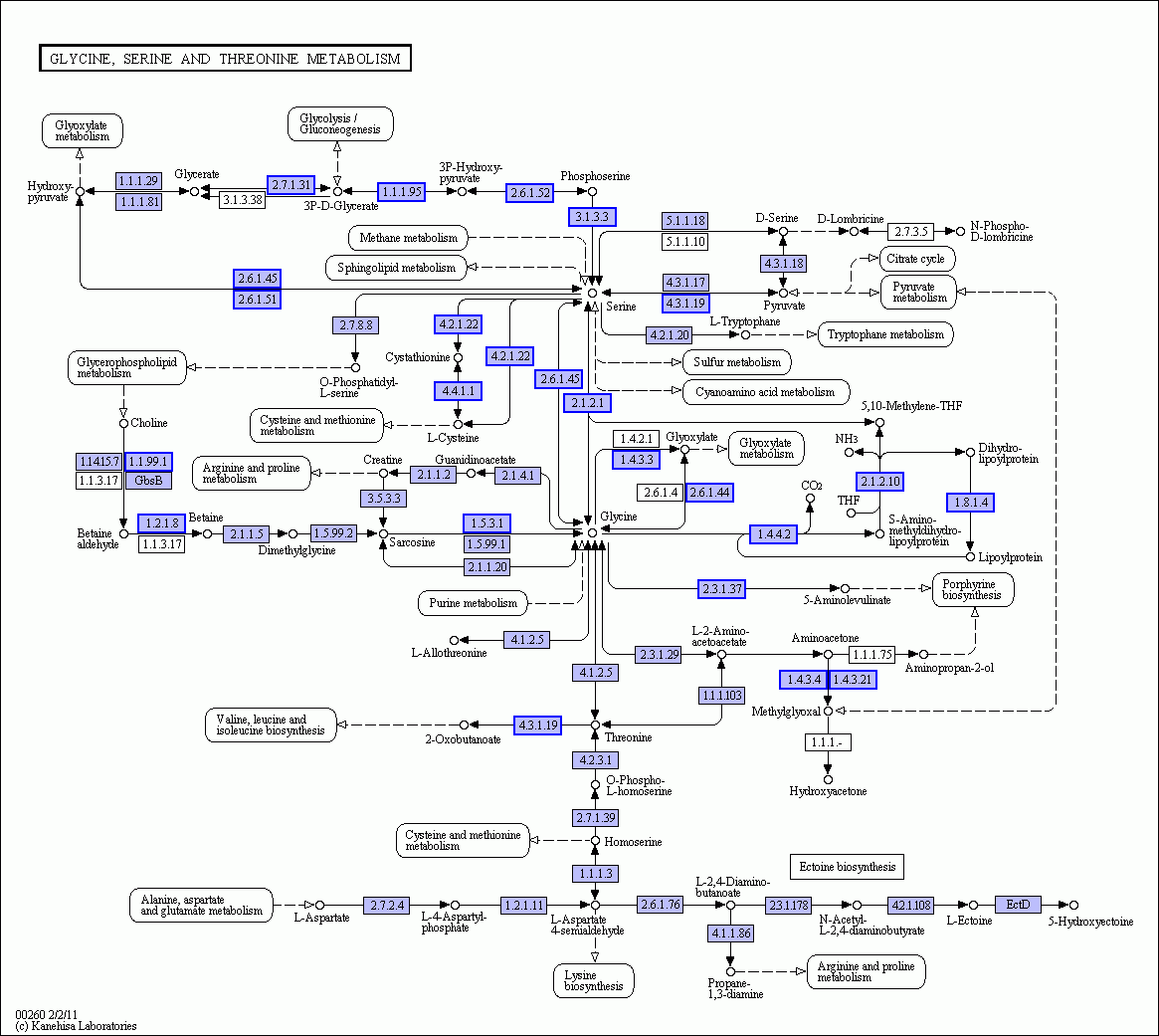

Supplement: Table S4 — KEGG Classification of the unigenes. (ZIP) [file pone.0079516.s004.zip › Kegg/Pathway_Map/ko00260.png]

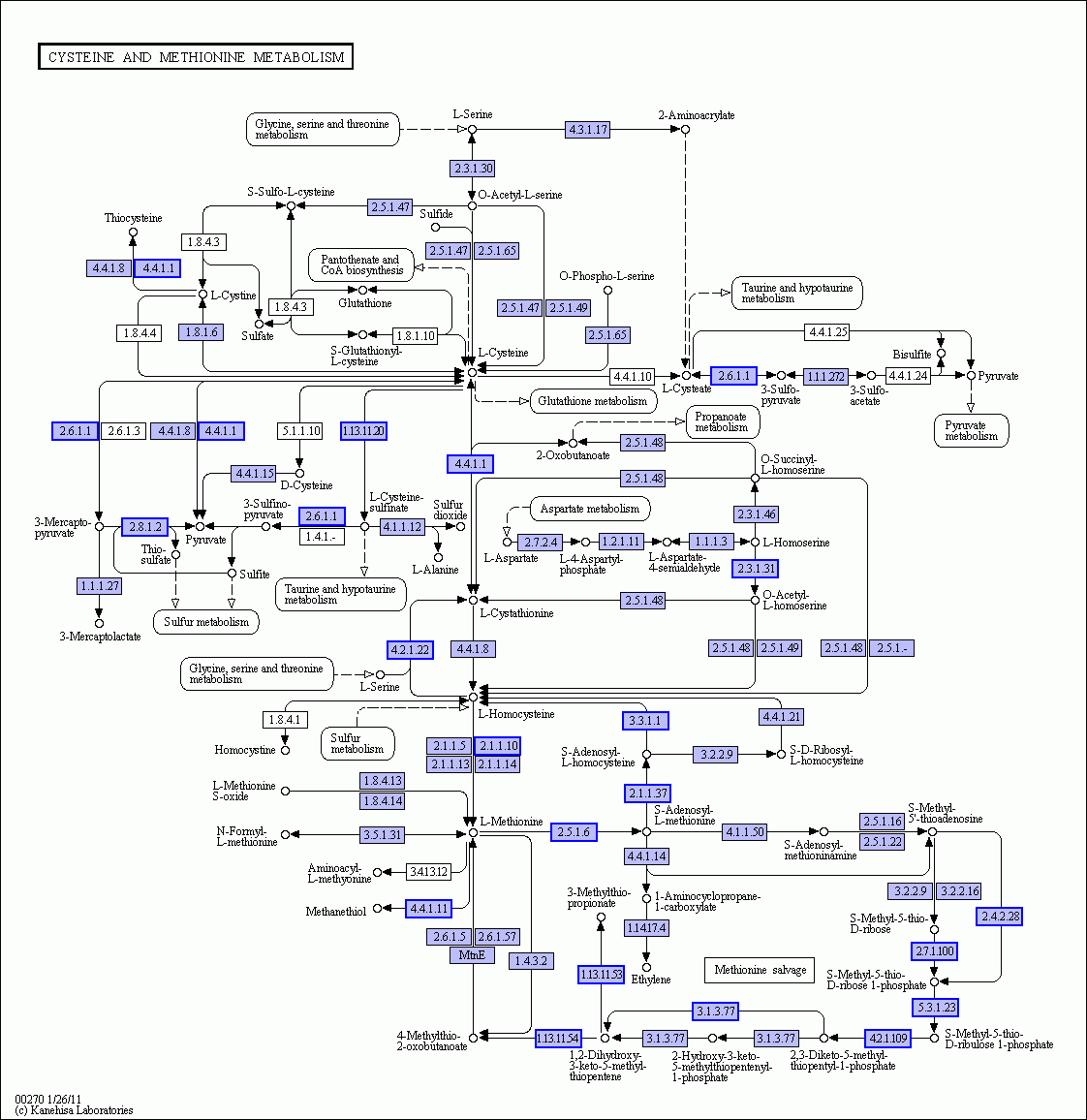

Supplement: Table S4 — KEGG Classification of the unigenes. (ZIP) [file pone.0079516.s004.zip › Kegg/Pathway_Map/ko00270.png]

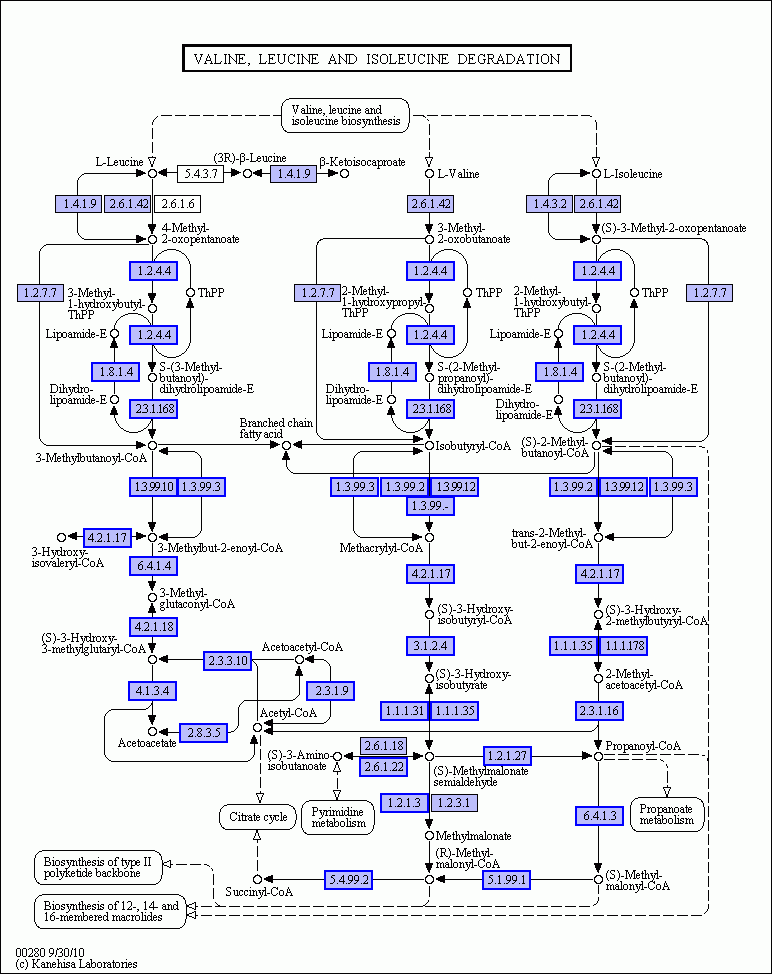

Supplement: Table S4 — KEGG Classification of the unigenes. (ZIP) [file pone.0079516.s004.zip › Kegg/Pathway_Map/ko00280.png]

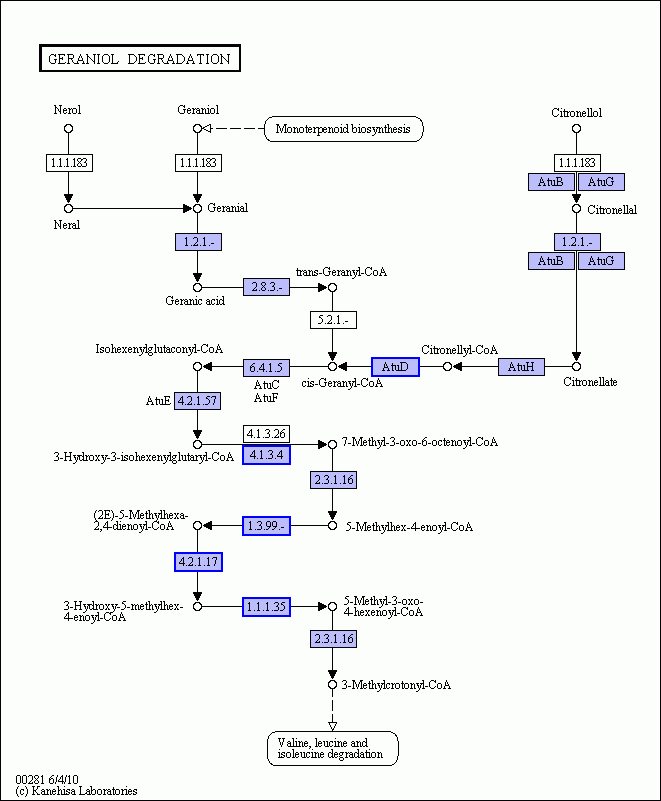

Supplement: Table S4 — KEGG Classification of the unigenes. (ZIP) [file pone.0079516.s004.zip › Kegg/Pathway_Map/ko00281.png]

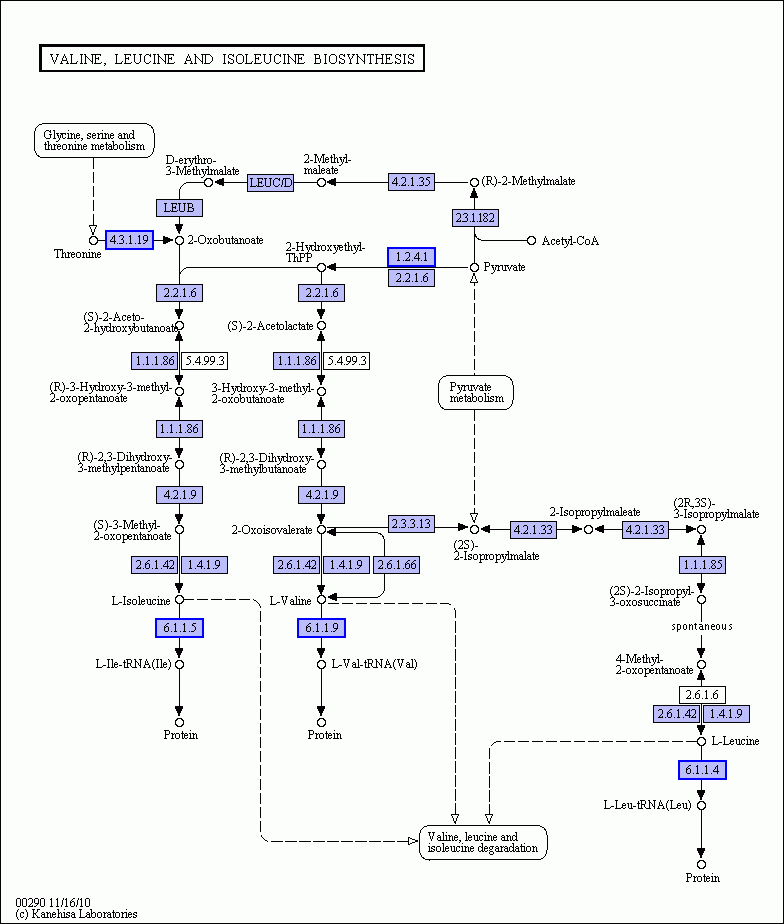

Supplement: Table S4 — KEGG Classification of the unigenes. (ZIP) [file pone.0079516.s004.zip › Kegg/Pathway_Map/ko00290.png]

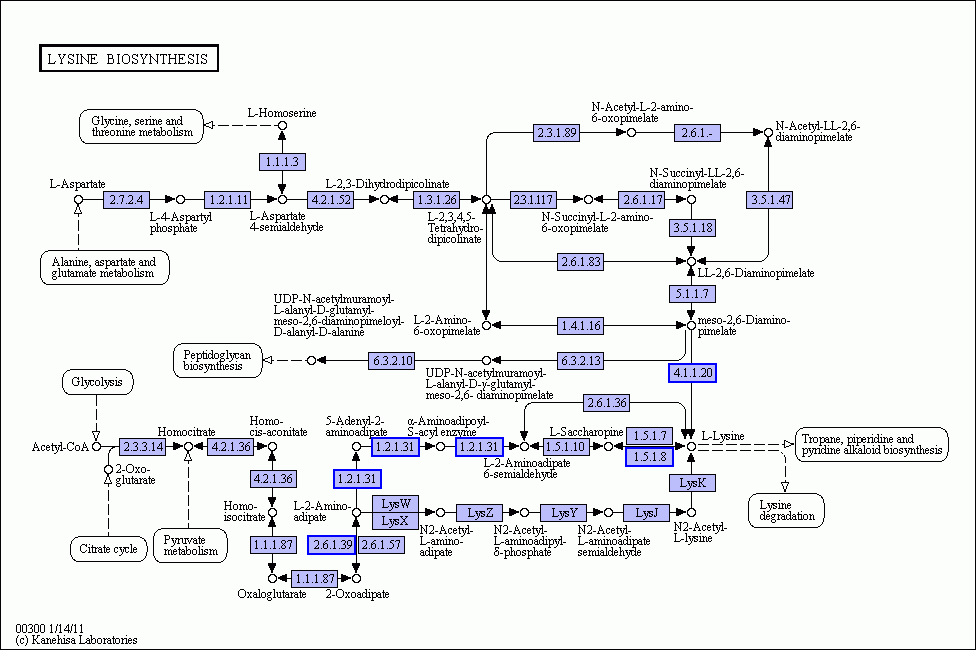

Supplement: Table S4 — KEGG Classification of the unigenes. (ZIP) [file pone.0079516.s004.zip › Kegg/Pathway_Map/ko00300.png]

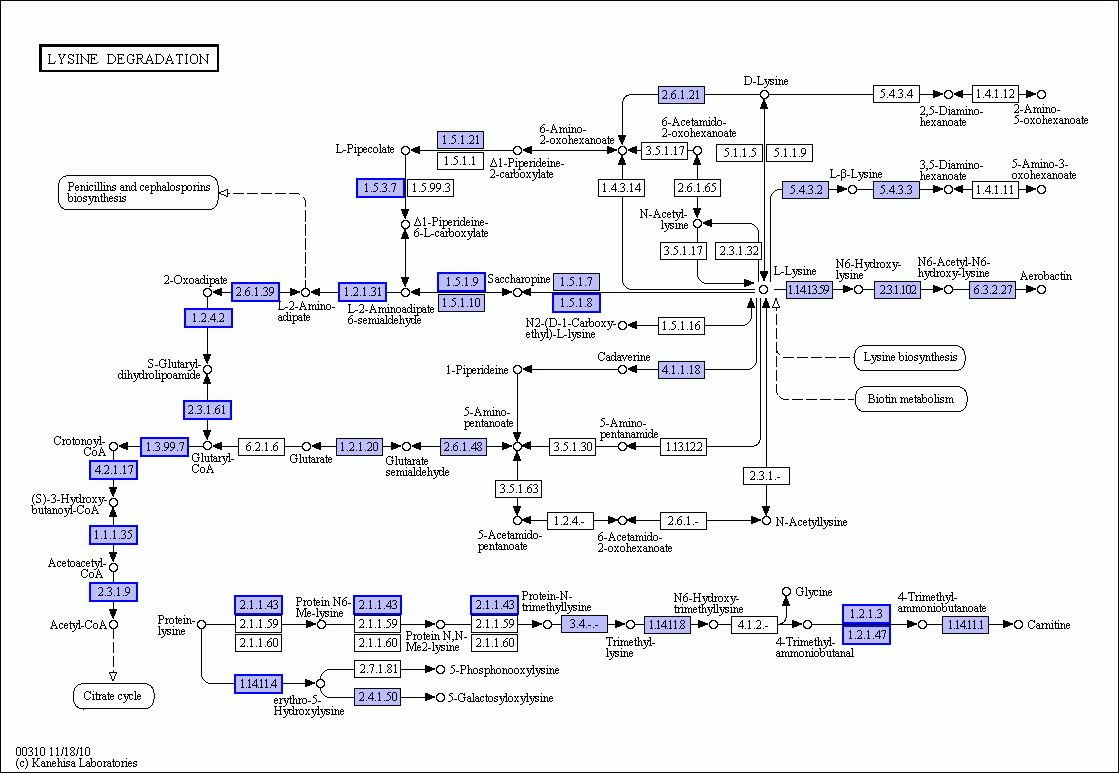

Supplement: Table S4 — KEGG Classification of the unigenes. (ZIP) [file pone.0079516.s004.zip › Kegg/Pathway_Map/ko00310.png]

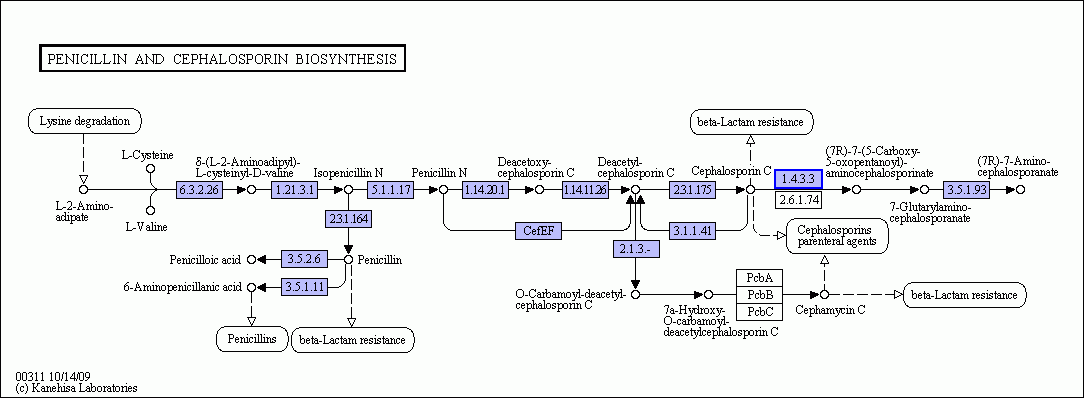

Supplement: Table S4 — KEGG Classification of the unigenes. (ZIP) [file pone.0079516.s004.zip › Kegg/Pathway_Map/ko00311.png]

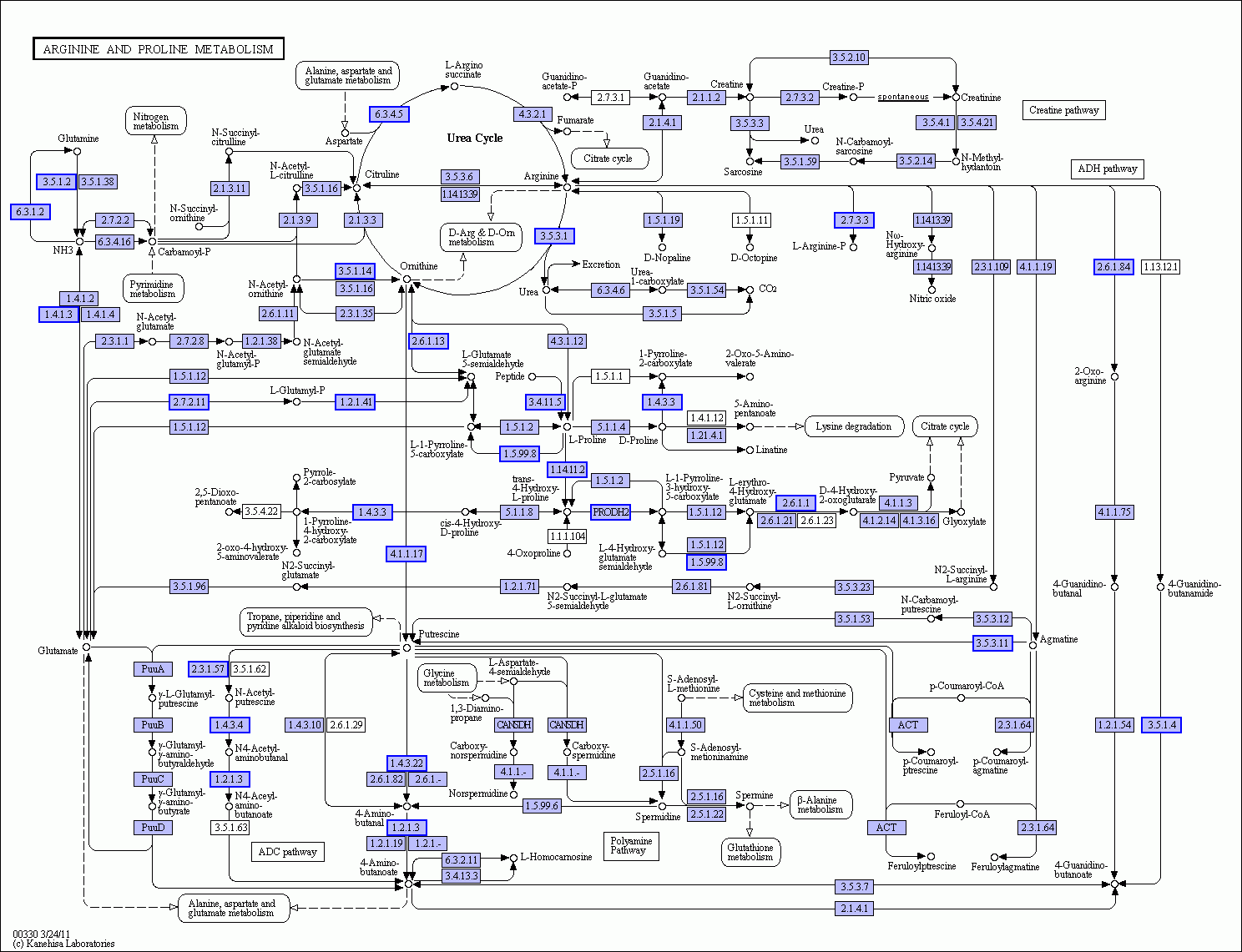

Supplement: Table S4 — KEGG Classification of the unigenes. (ZIP) [file pone.0079516.s004.zip › Kegg/Pathway_Map/ko00330.png]

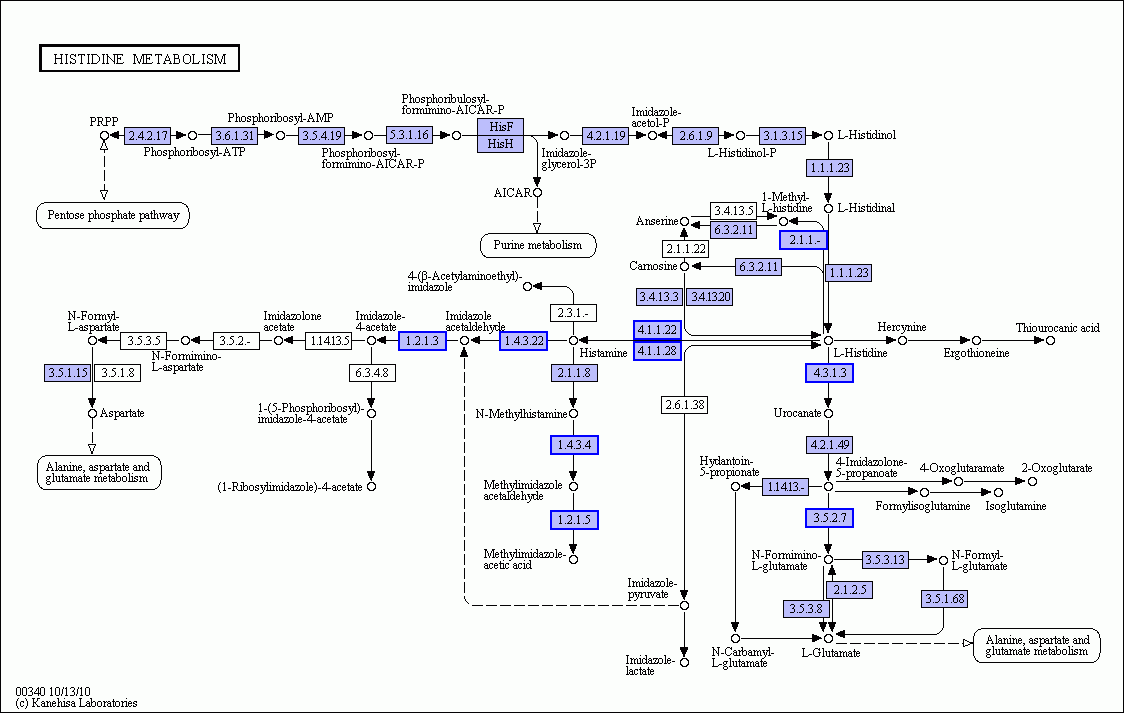

Supplement: Table S4 — KEGG Classification of the unigenes. (ZIP) [file pone.0079516.s004.zip › Kegg/Pathway_Map/ko00340.png]

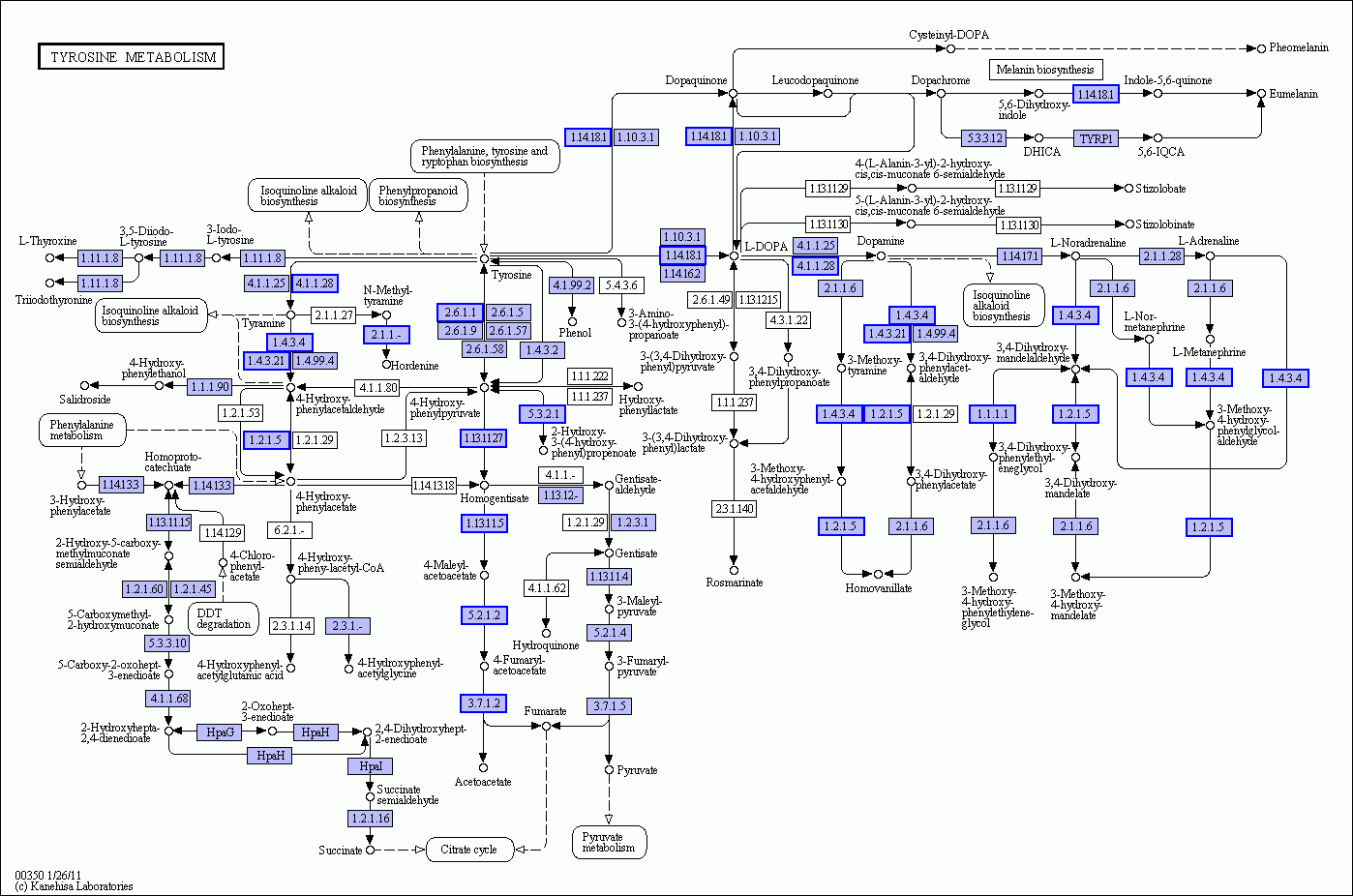

Supplement: Table S4 — KEGG Classification of the unigenes. (ZIP) [file pone.0079516.s004.zip › Kegg/Pathway_Map/ko00350.png]

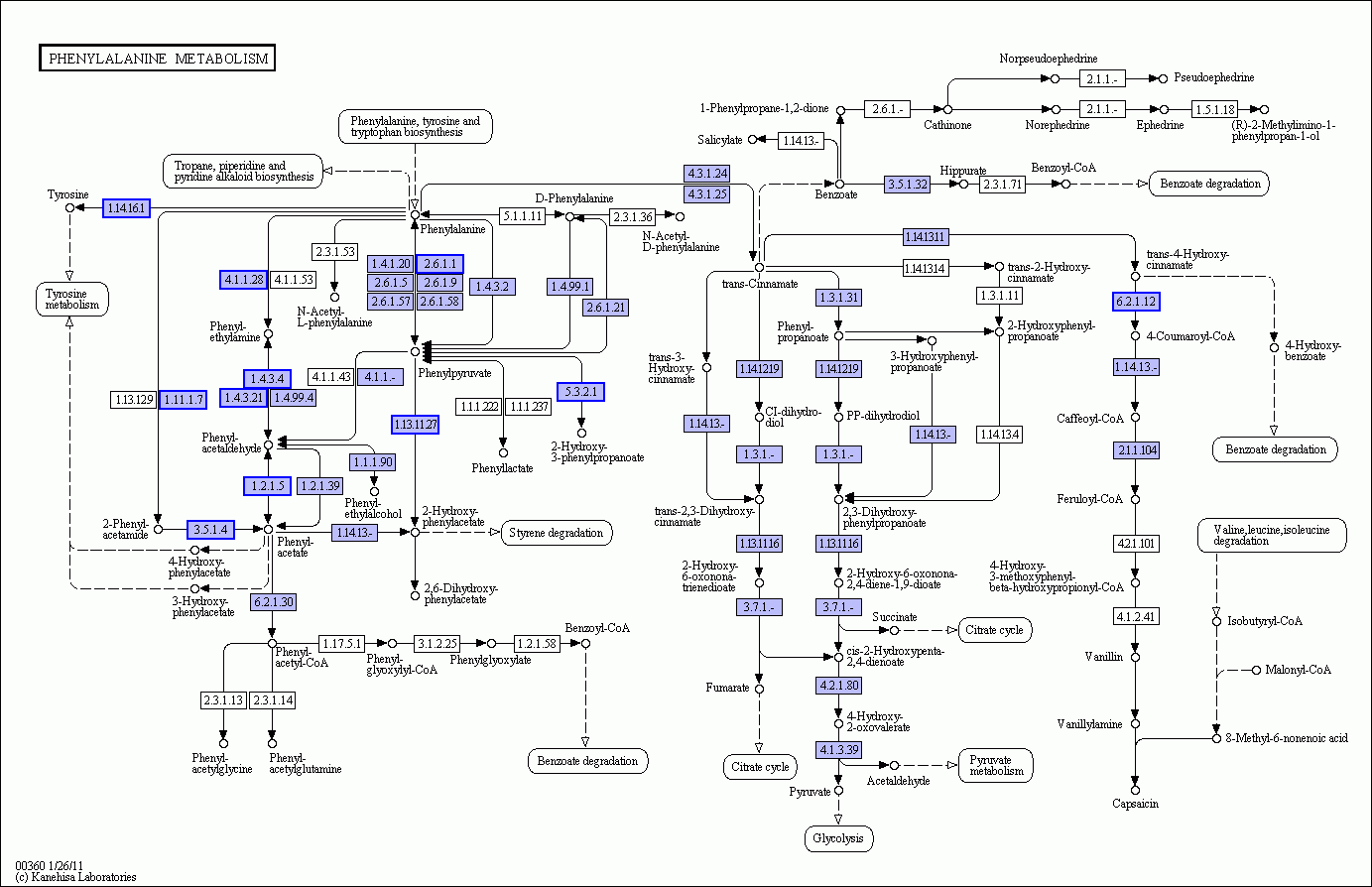

Supplement: Table S4 — KEGG Classification of the unigenes. (ZIP) [file pone.0079516.s004.zip › Kegg/Pathway_Map/ko00360.png]

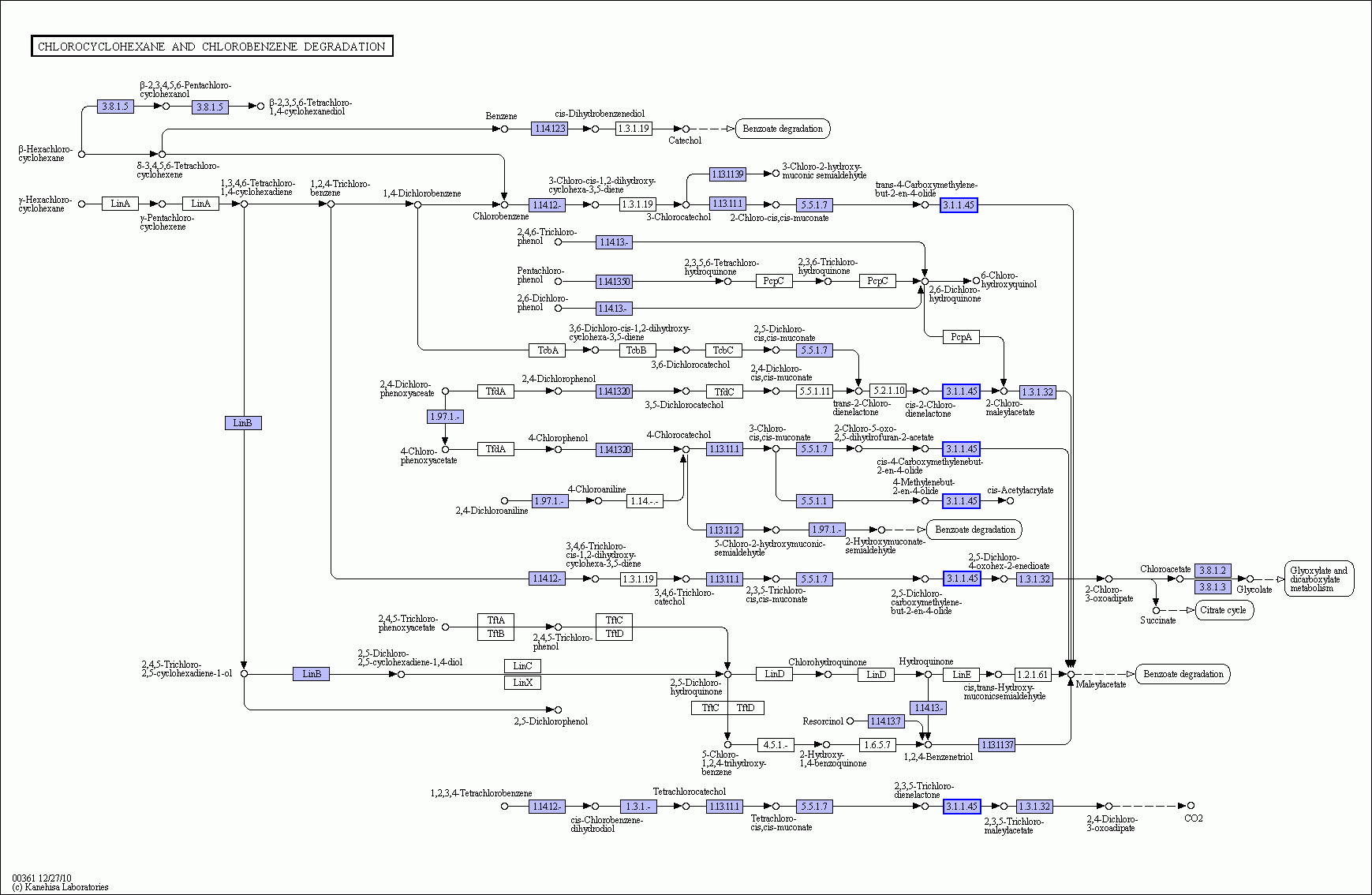

Supplement: Table S4 — KEGG Classification of the unigenes. (ZIP) [file pone.0079516.s004.zip › Kegg/Pathway_Map/ko00361.png]

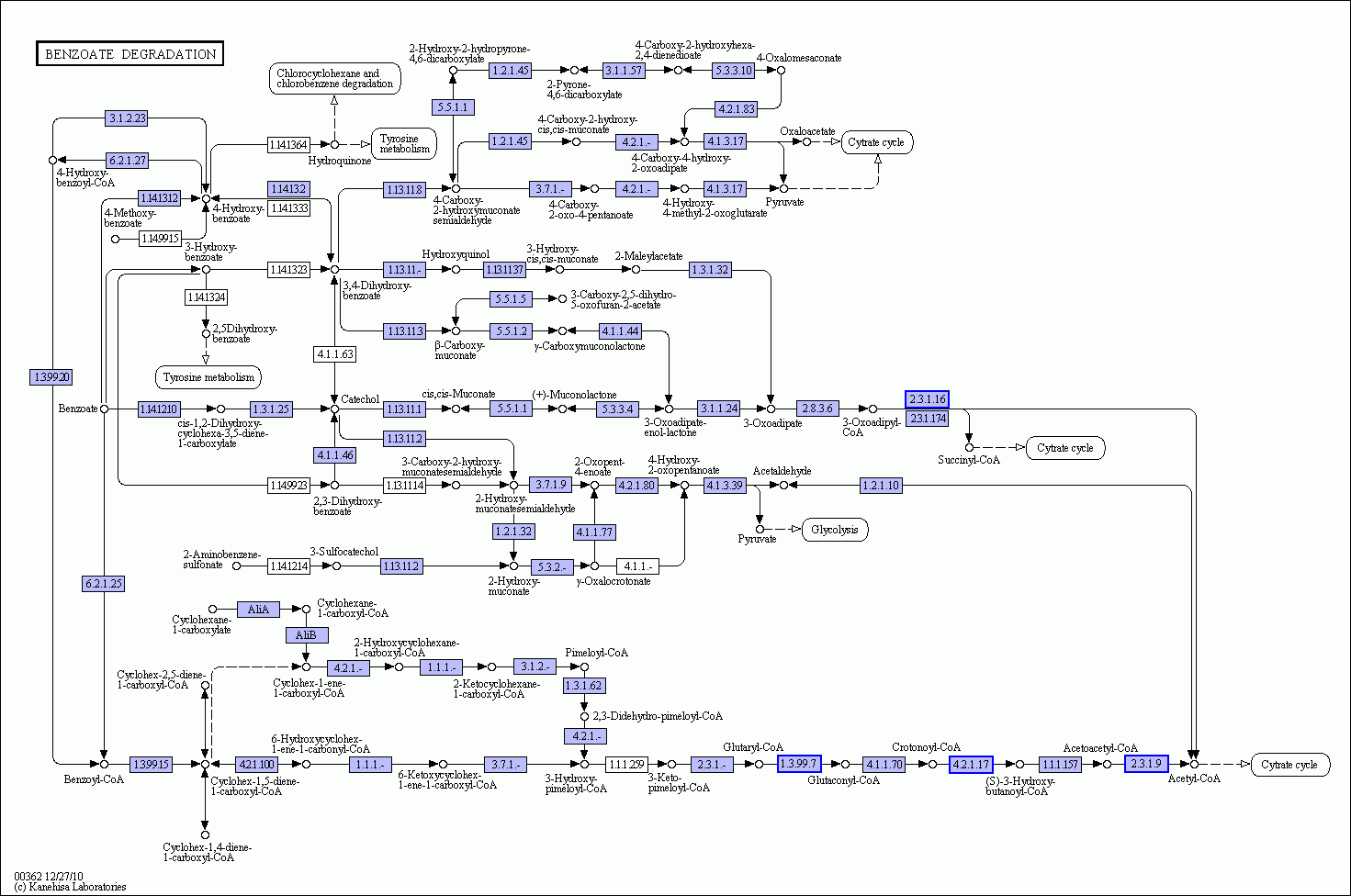

Supplement: Table S4 — KEGG Classification of the unigenes. (ZIP) [file pone.0079516.s004.zip › Kegg/Pathway_Map/ko00362.png]

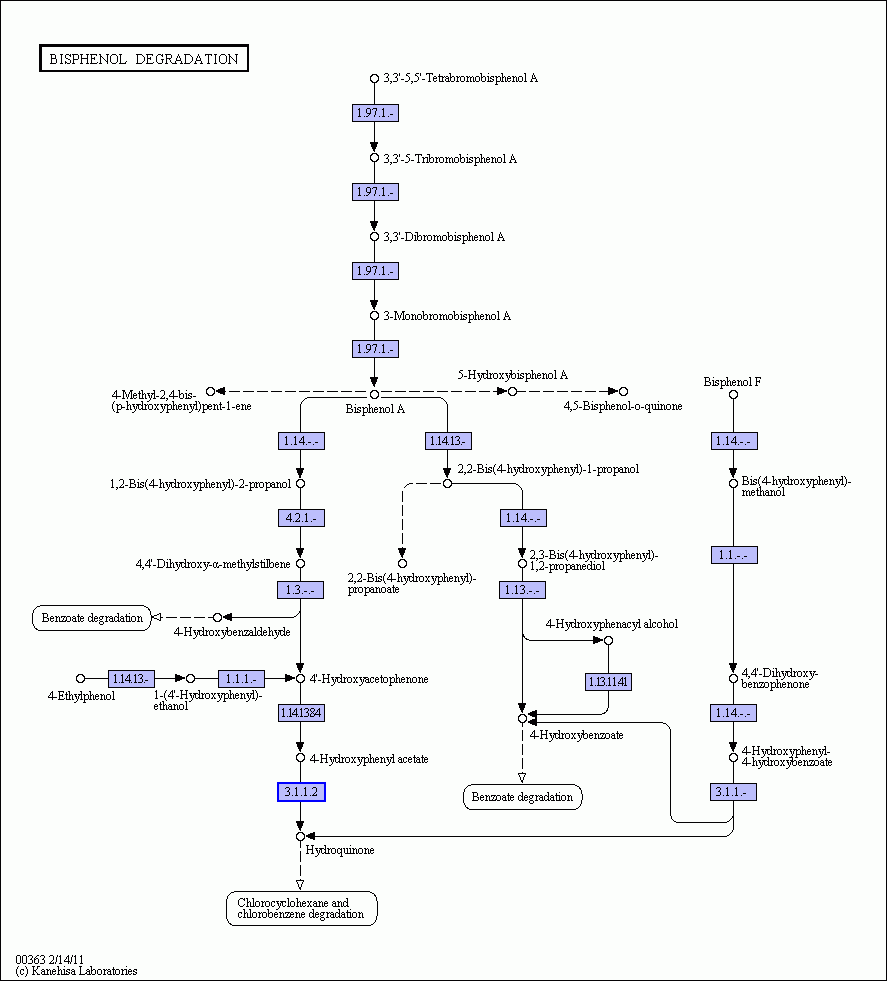

Supplement: Table S4 — KEGG Classification of the unigenes. (ZIP) [file pone.0079516.s004.zip › Kegg/Pathway_Map/ko00363.png]

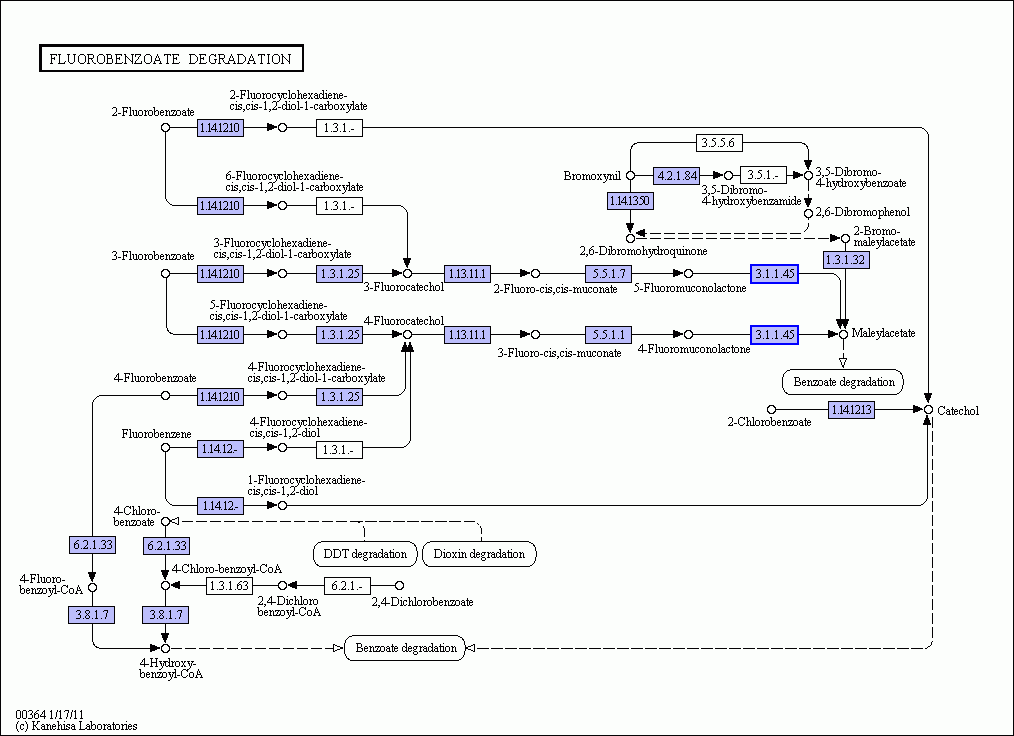

Supplement: Table S4 — KEGG Classification of the unigenes. (ZIP) [file pone.0079516.s004.zip › Kegg/Pathway_Map/ko00364.png]

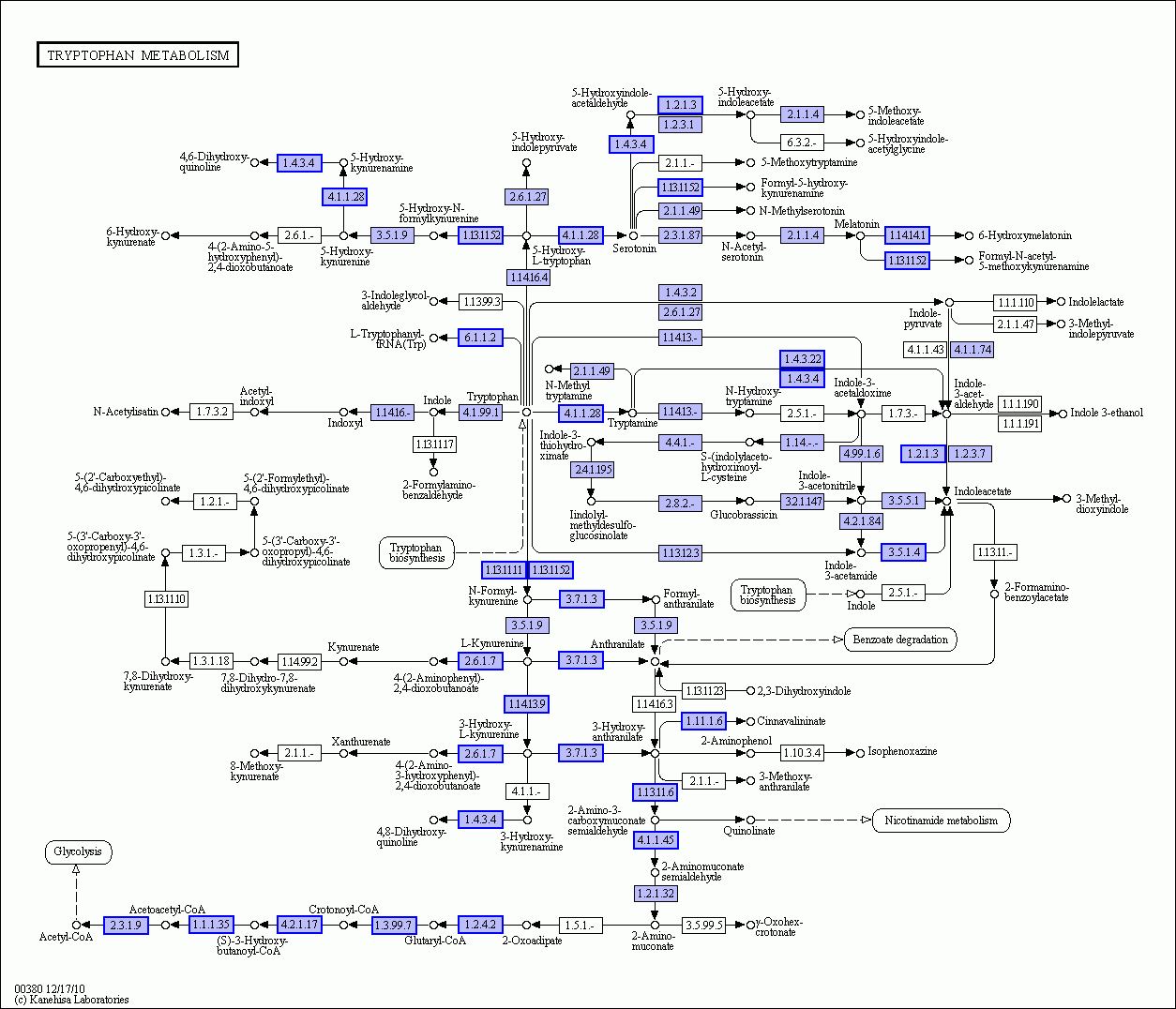

Supplement: Table S4 — KEGG Classification of the unigenes. (ZIP) [file pone.0079516.s004.zip › Kegg/Pathway_Map/ko00380.png]

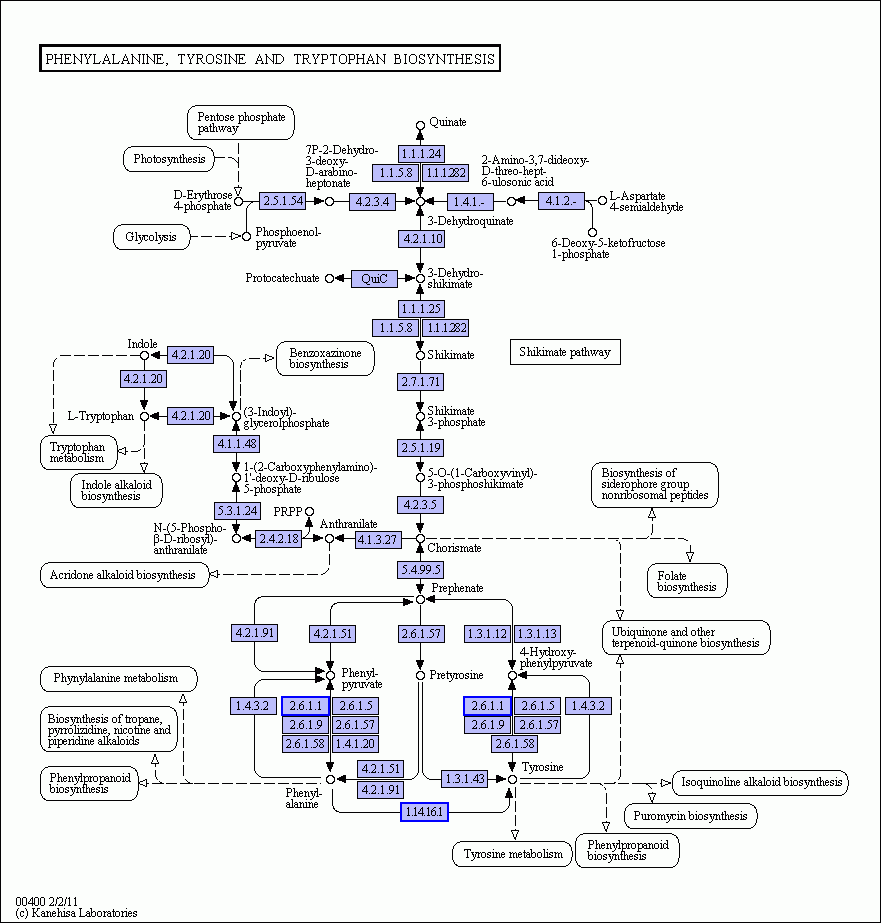

Supplement: Table S4 — KEGG Classification of the unigenes. (ZIP) [file pone.0079516.s004.zip › Kegg/Pathway_Map/ko00400.png]

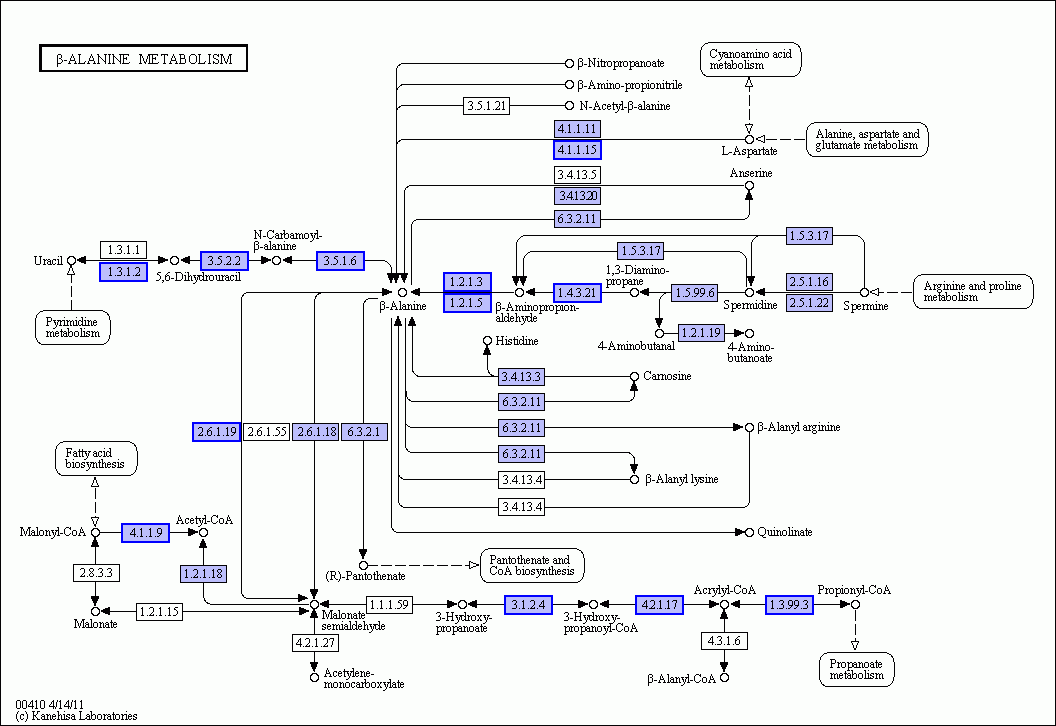

Supplement: Table S4 — KEGG Classification of the unigenes. (ZIP) [file pone.0079516.s004.zip › Kegg/Pathway_Map/ko00410.png]

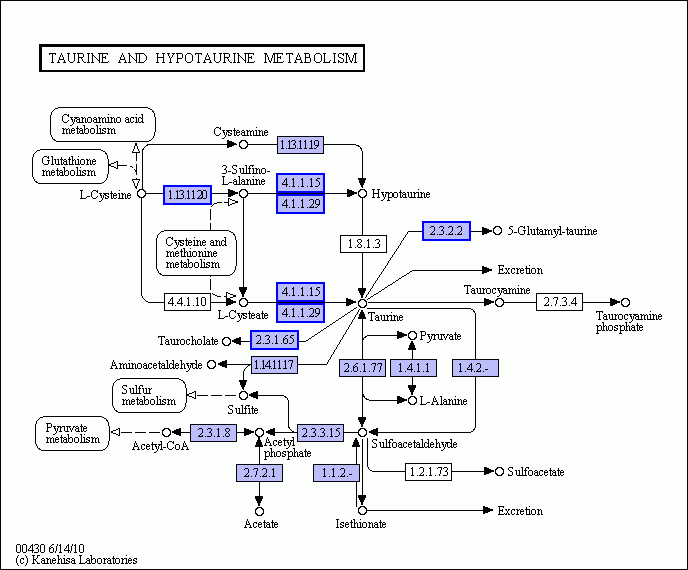

Supplement: Table S4 — KEGG Classification of the unigenes. (ZIP) [file pone.0079516.s004.zip › Kegg/Pathway_Map/ko00430.png]

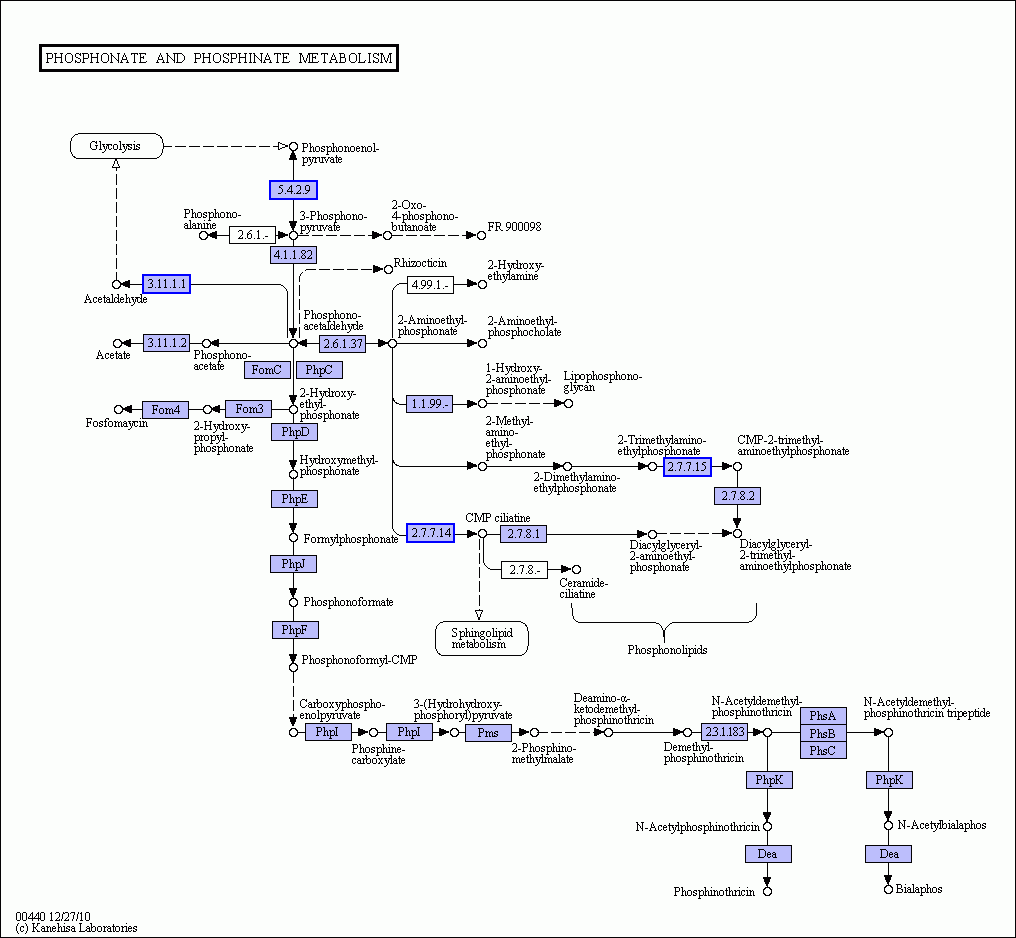

Supplement: Table S4 — KEGG Classification of the unigenes. (ZIP) [file pone.0079516.s004.zip › Kegg/Pathway_Map/ko00440.png]

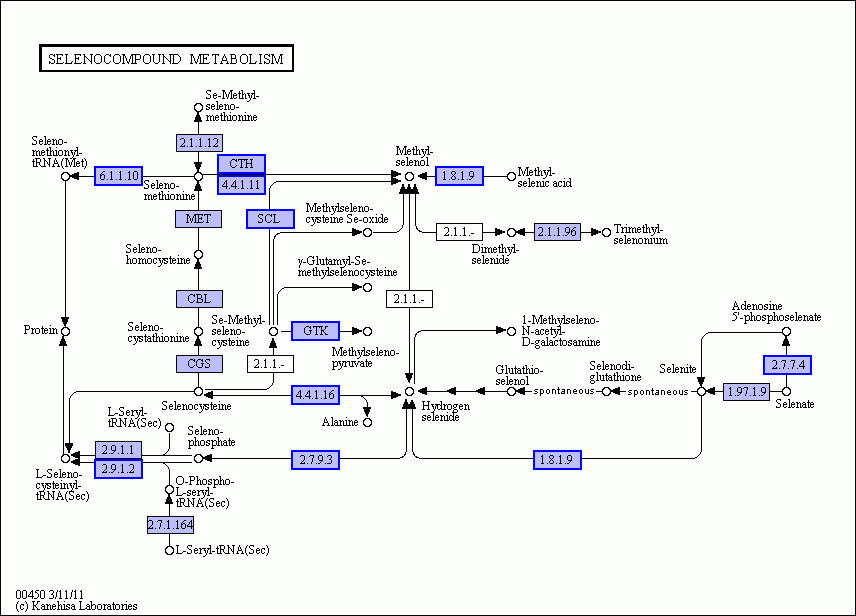

Supplement: Table S4 — KEGG Classification of the unigenes. (ZIP) [file pone.0079516.s004.zip › Kegg/Pathway_Map/ko00450.png]

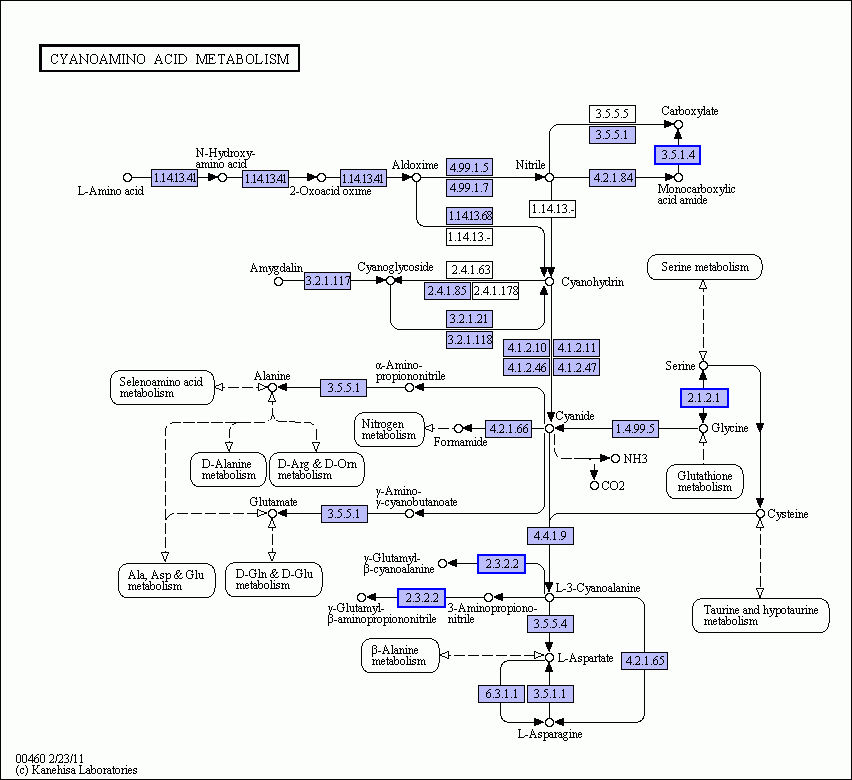

Supplement: Table S4 — KEGG Classification of the unigenes. (ZIP) [file pone.0079516.s004.zip › Kegg/Pathway_Map/ko00460.png]

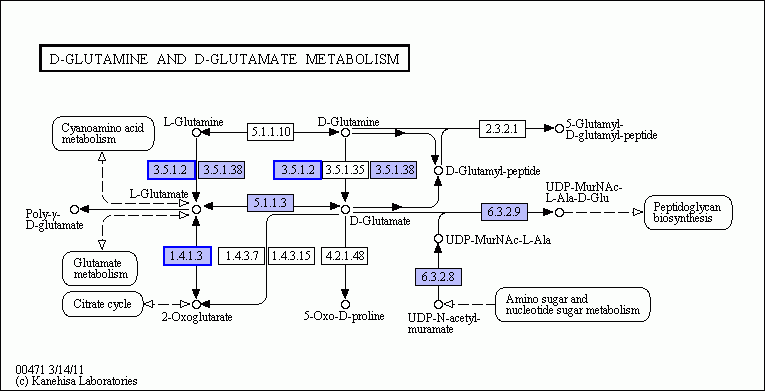

Supplement: Table S4 — KEGG Classification of the unigenes. (ZIP) [file pone.0079516.s004.zip › Kegg/Pathway_Map/ko00471.png]

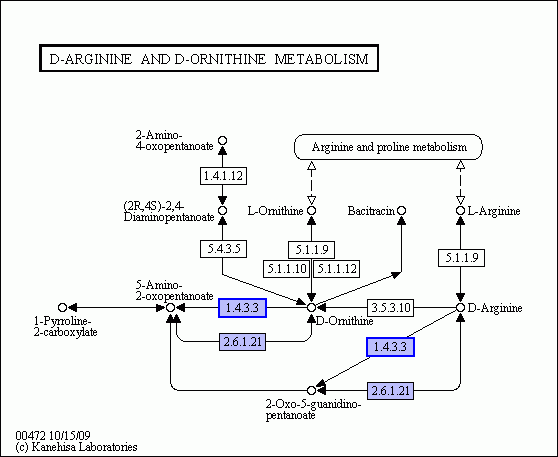

Supplement: Table S4 — KEGG Classification of the unigenes. (ZIP) [file pone.0079516.s004.zip › Kegg/Pathway_Map/ko00472.png]

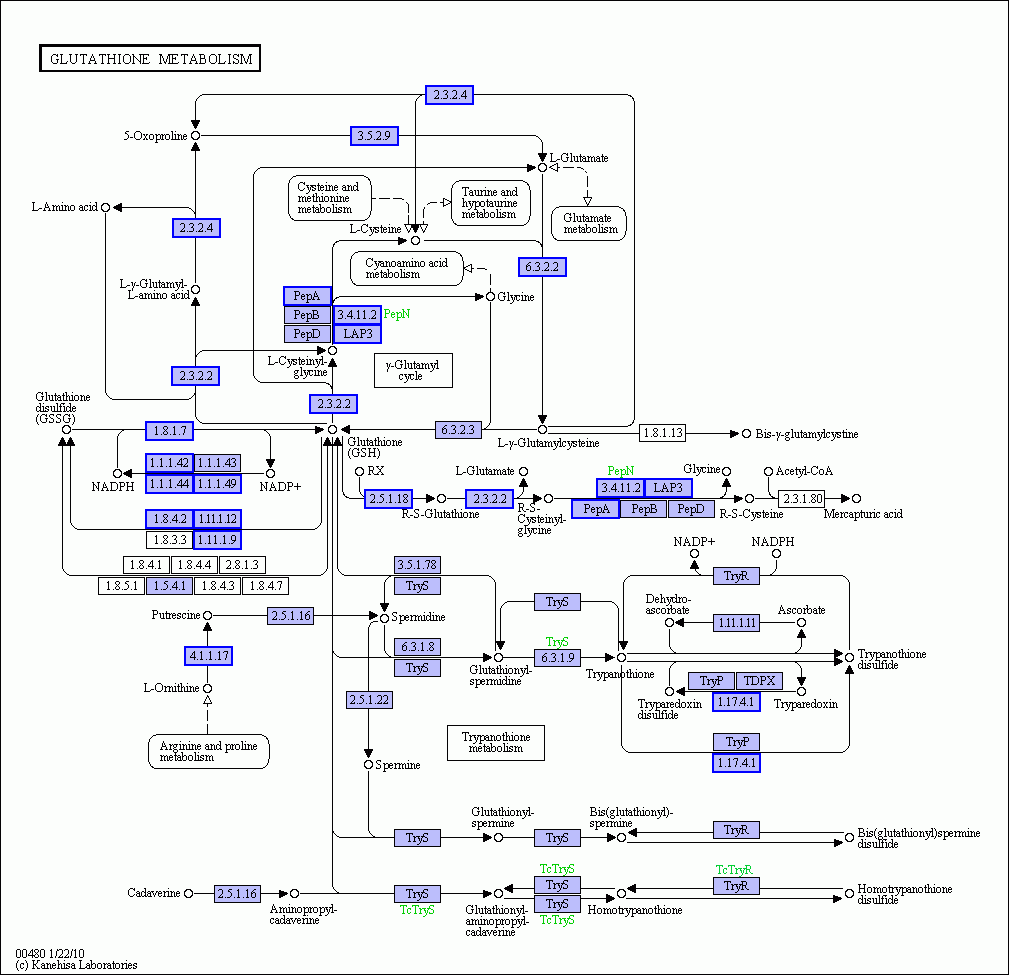

Supplement: Table S4 — KEGG Classification of the unigenes. (ZIP) [file pone.0079516.s004.zip › Kegg/Pathway_Map/ko00480.png]

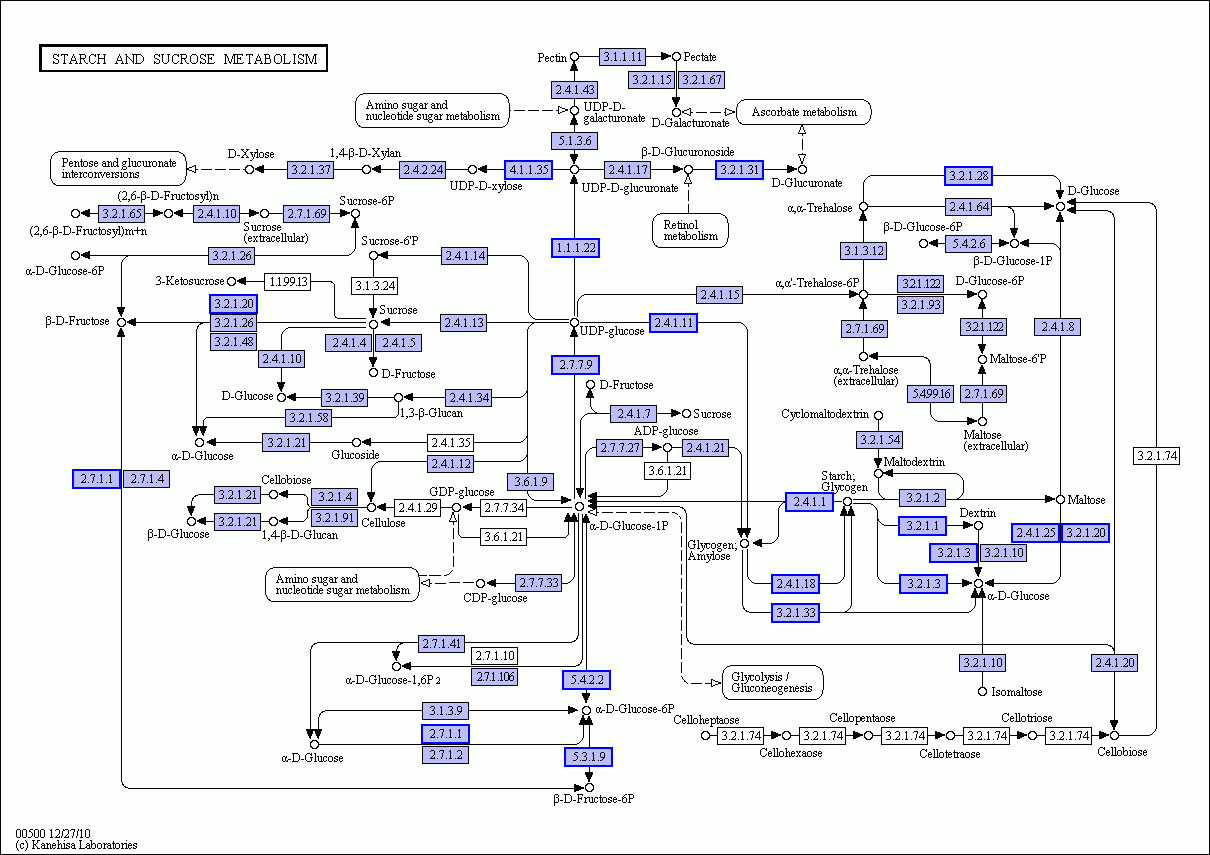

Supplement: Table S4 — KEGG Classification of the unigenes. (ZIP) [file pone.0079516.s004.zip › Kegg/Pathway_Map/ko00500.png]

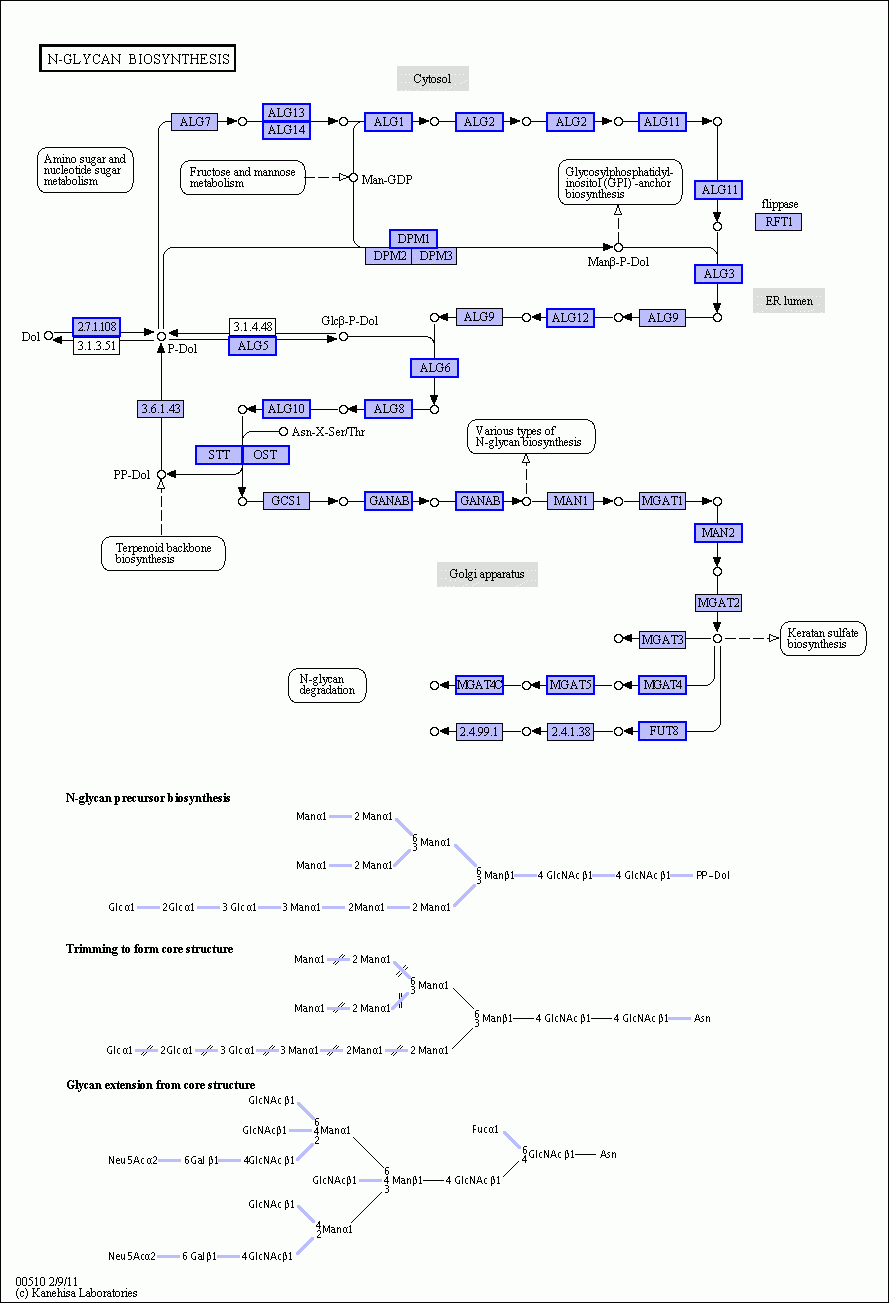

Supplement: Table S4 — KEGG Classification of the unigenes. (ZIP) [file pone.0079516.s004.zip › Kegg/Pathway_Map/ko00510.png]

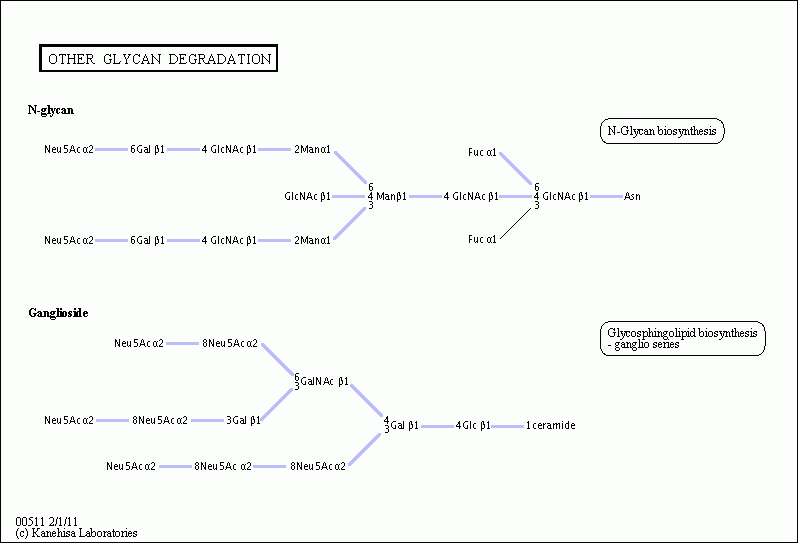

Supplement: Table S4 — KEGG Classification of the unigenes. (ZIP) [file pone.0079516.s004.zip › Kegg/Pathway_Map/ko00511.png]

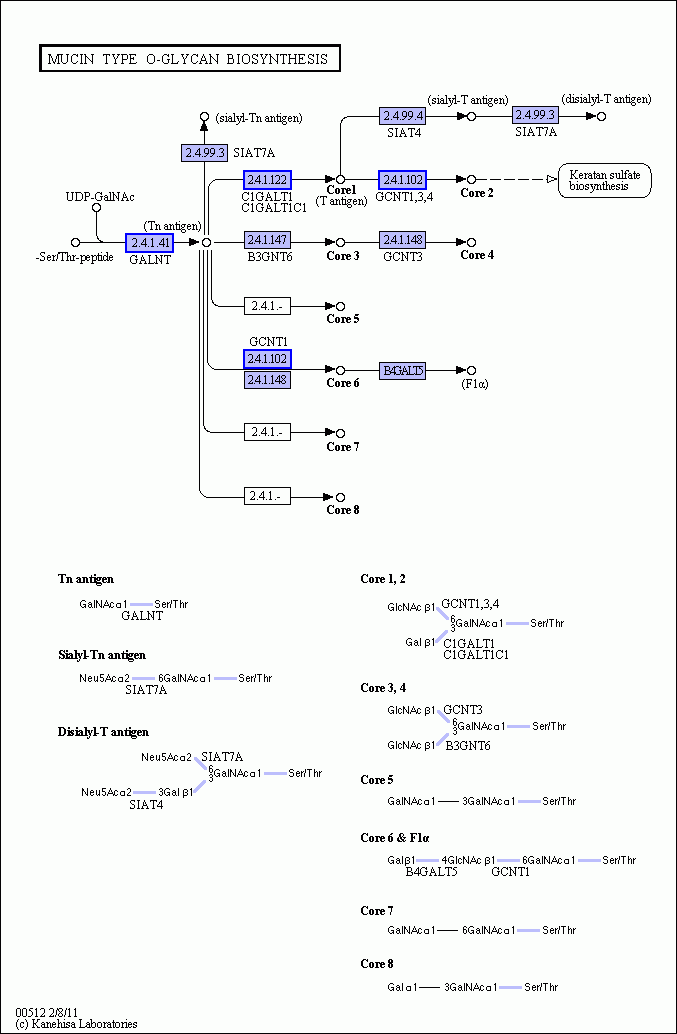

Supplement: Table S4 — KEGG Classification of the unigenes. (ZIP) [file pone.0079516.s004.zip › Kegg/Pathway_Map/ko00512.png]

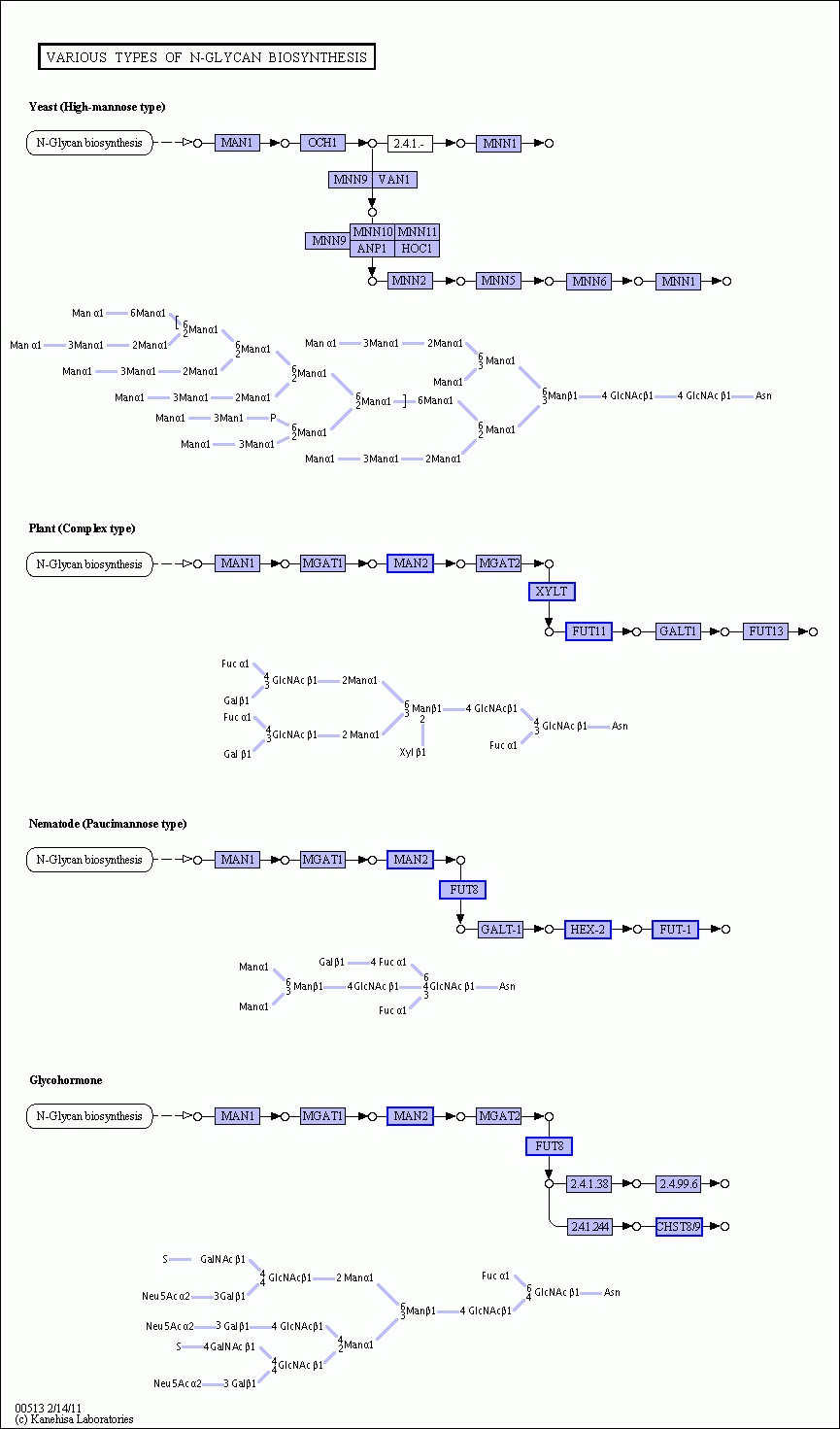

Supplement: Table S4 — KEGG Classification of the unigenes. (ZIP) [file pone.0079516.s004.zip › Kegg/Pathway_Map/ko00513.png]

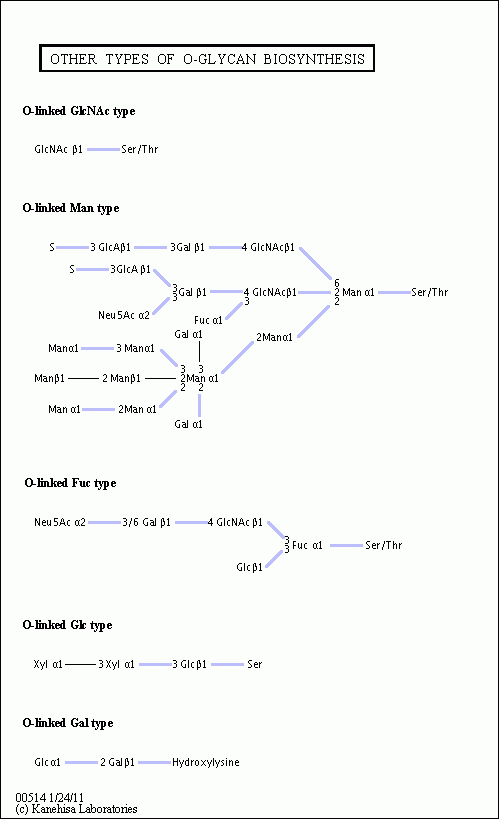

Supplement: Table S4 — KEGG Classification of the unigenes. (ZIP) [file pone.0079516.s004.zip › Kegg/Pathway_Map/ko00514.png]

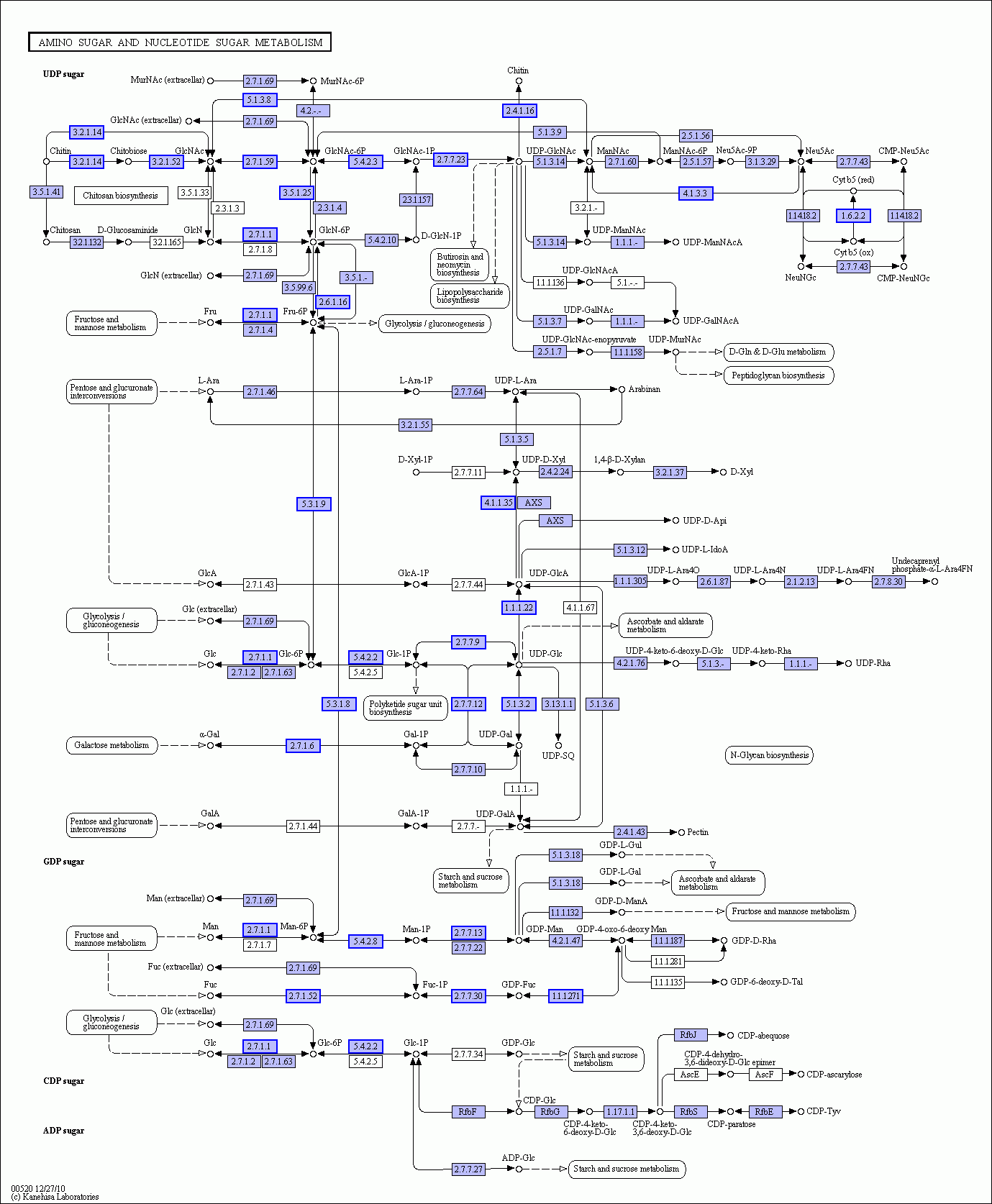

Supplement: Table S4 — KEGG Classification of the unigenes. (ZIP) [file pone.0079516.s004.zip › Kegg/Pathway_Map/ko00520.png]

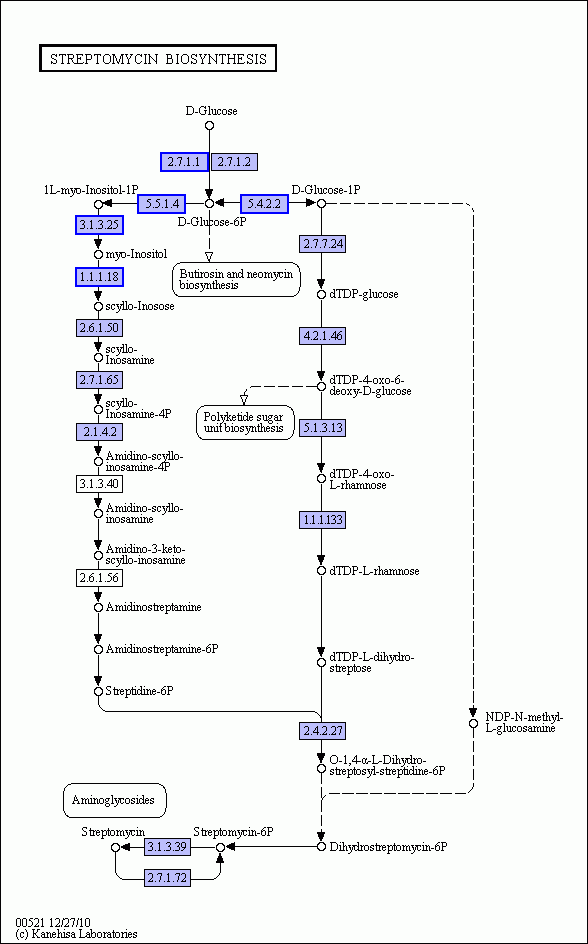

Supplement: Table S4 — KEGG Classification of the unigenes. (ZIP) [file pone.0079516.s004.zip › Kegg/Pathway_Map/ko00521.png]

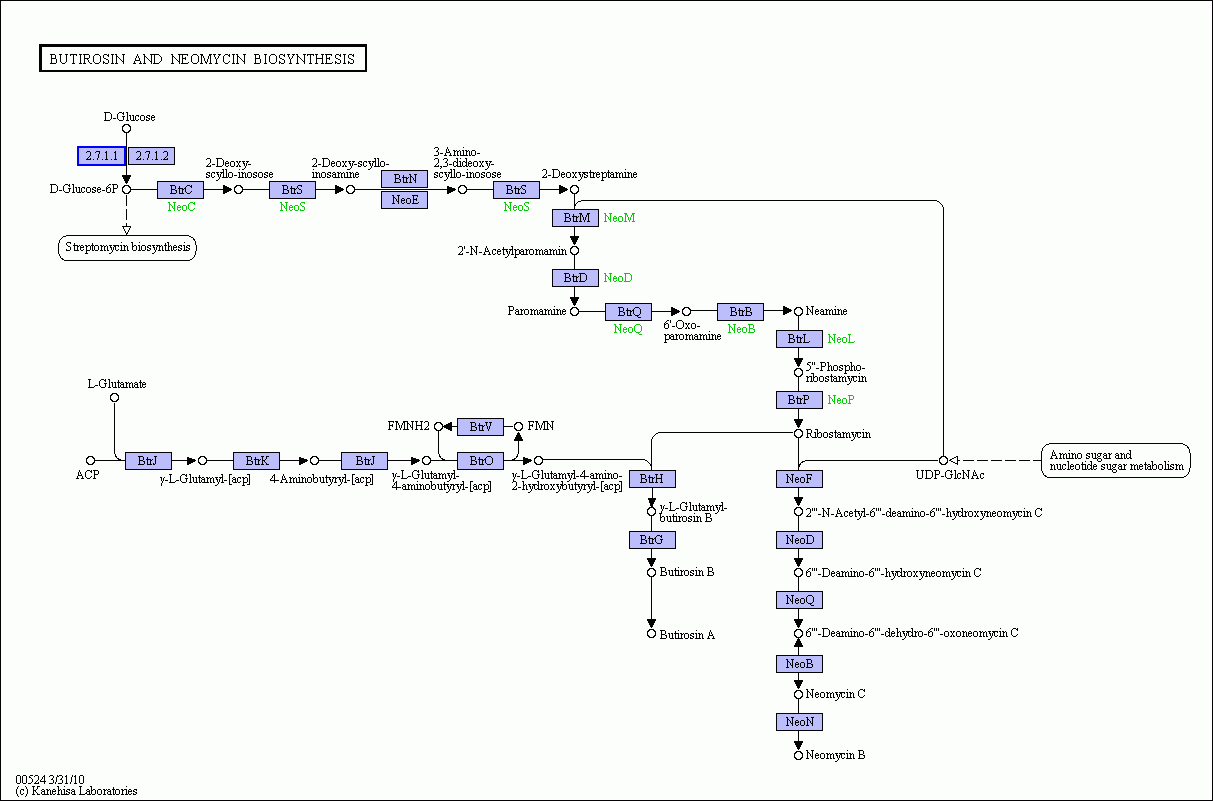

Supplement: Table S4 — KEGG Classification of the unigenes. (ZIP) [file pone.0079516.s004.zip › Kegg/Pathway_Map/ko00524.png]

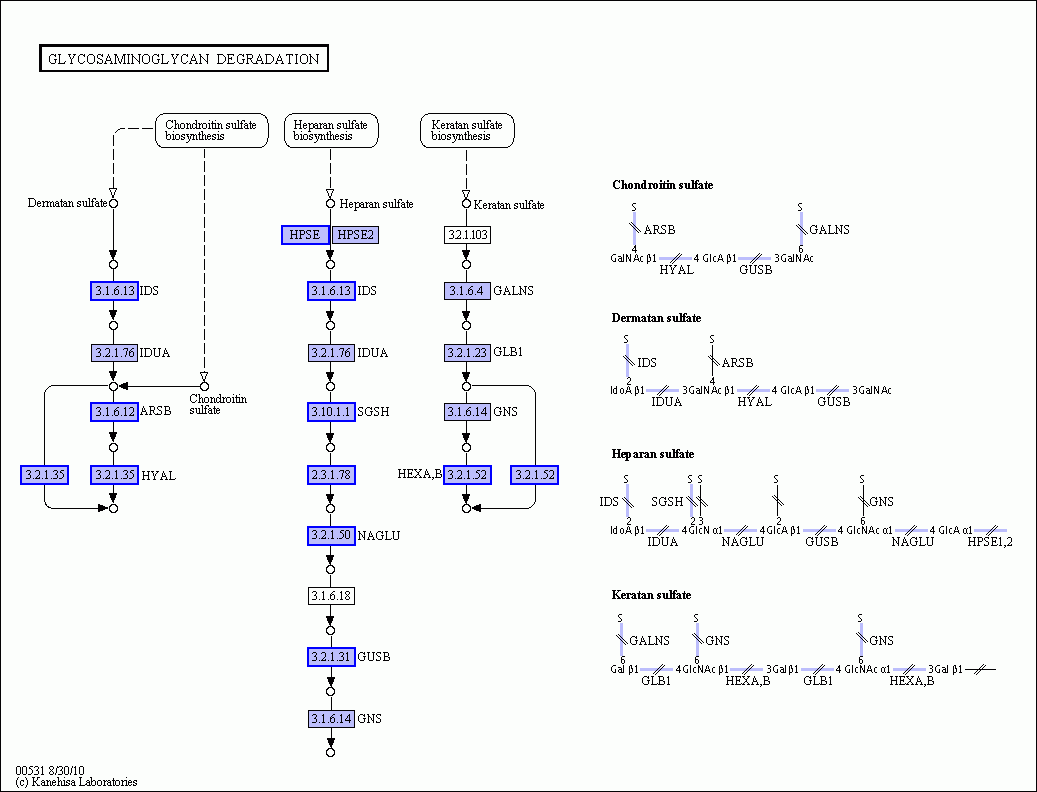

Supplement: Table S4 — KEGG Classification of the unigenes. (ZIP) [file pone.0079516.s004.zip › Kegg/Pathway_Map/ko00531.png]

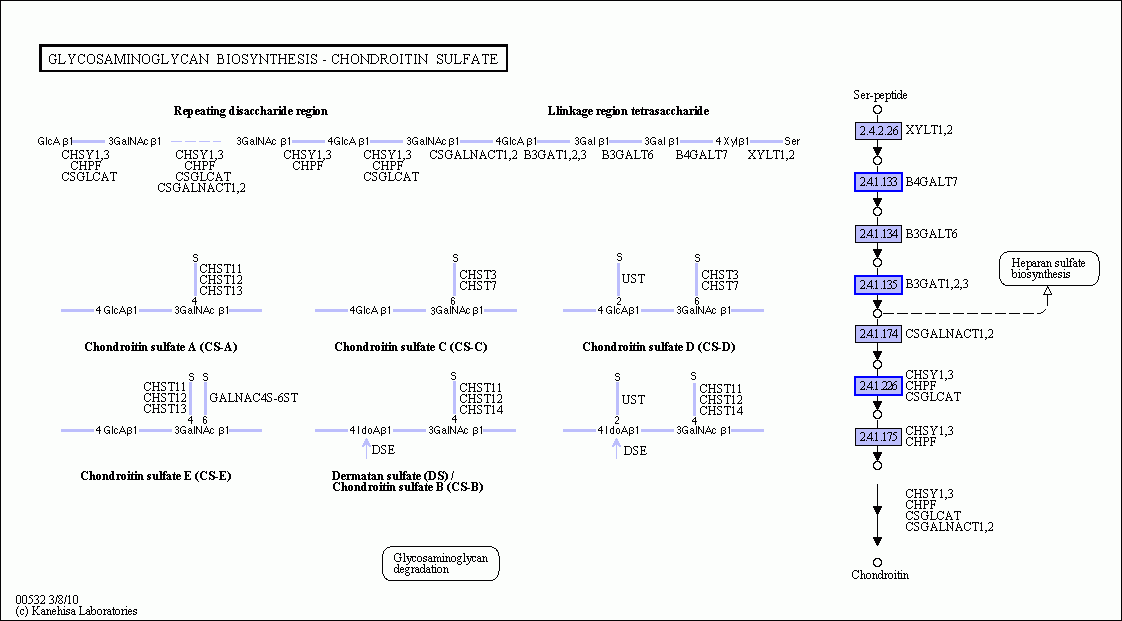

Supplement: Table S4 — KEGG Classification of the unigenes. (ZIP) [file pone.0079516.s004.zip › Kegg/Pathway_Map/ko00532.png]

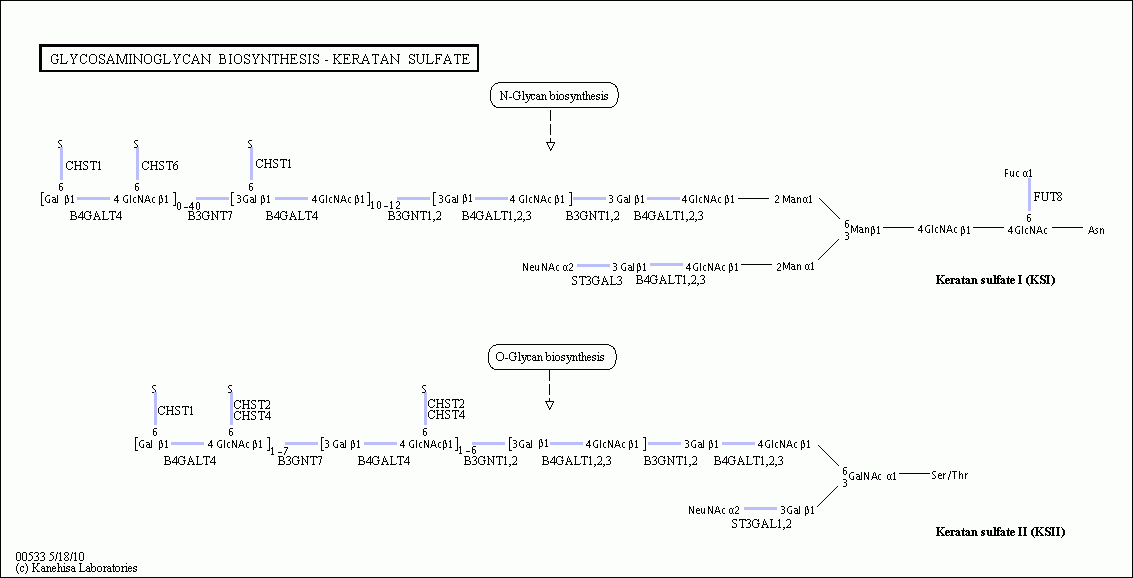

Supplement: Table S4 — KEGG Classification of the unigenes. (ZIP) [file pone.0079516.s004.zip › Kegg/Pathway_Map/ko00533.png]

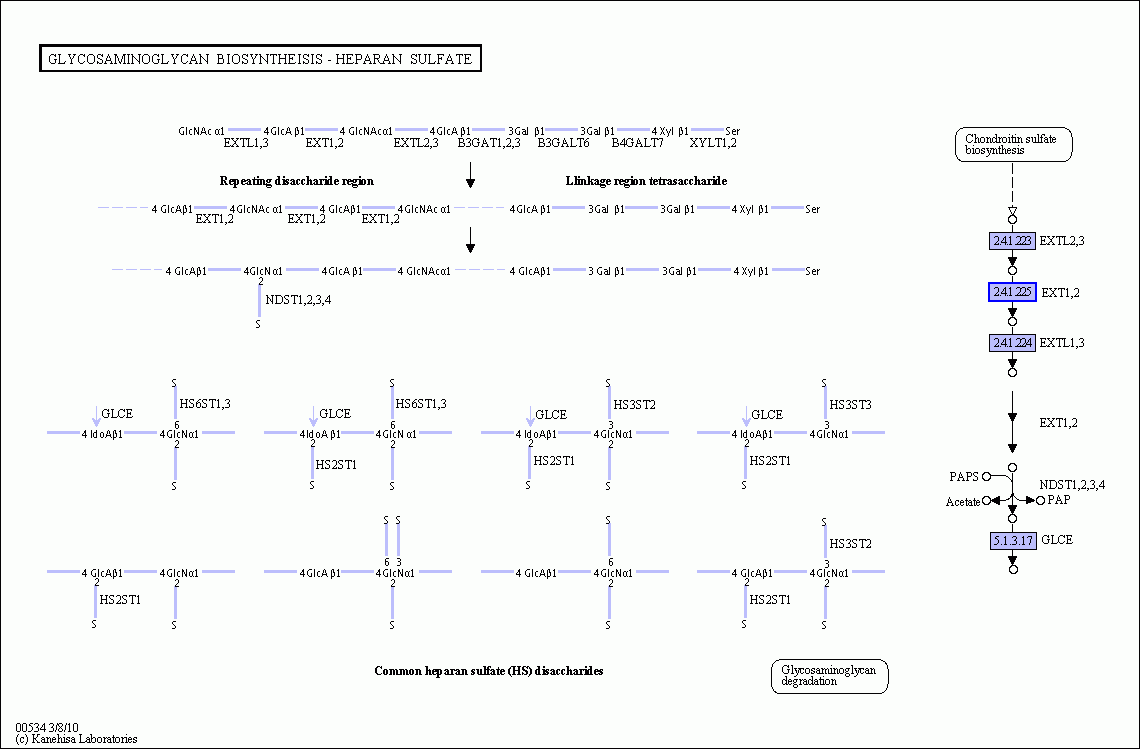

Supplement: Table S4 — KEGG Classification of the unigenes. (ZIP) [file pone.0079516.s004.zip › Kegg/Pathway_Map/ko00534.png]

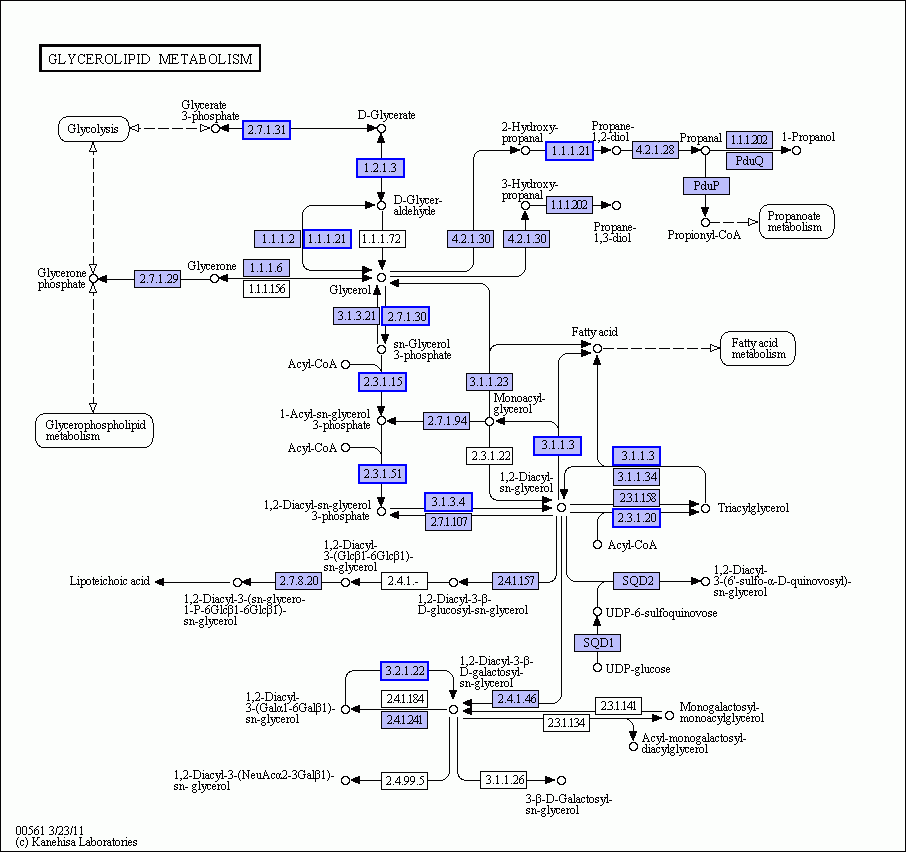

Supplement: Table S4 — KEGG Classification of the unigenes. (ZIP) [file pone.0079516.s004.zip › Kegg/Pathway_Map/ko00561.png]

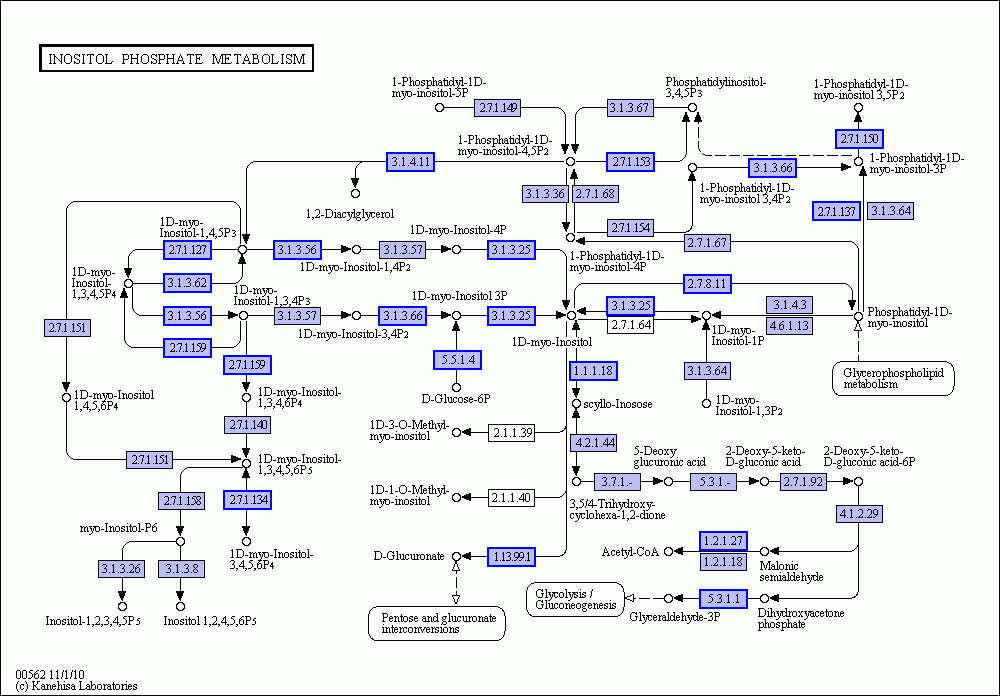

Supplement: Table S4 — KEGG Classification of the unigenes. (ZIP) [file pone.0079516.s004.zip › Kegg/Pathway_Map/ko00562.png]

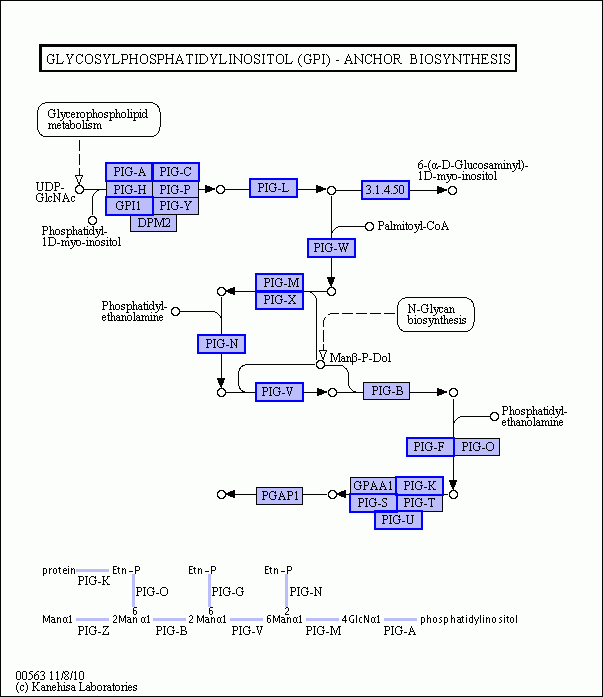

Supplement: Table S4 — KEGG Classification of the unigenes. (ZIP) [file pone.0079516.s004.zip › Kegg/Pathway_Map/ko00563.png]

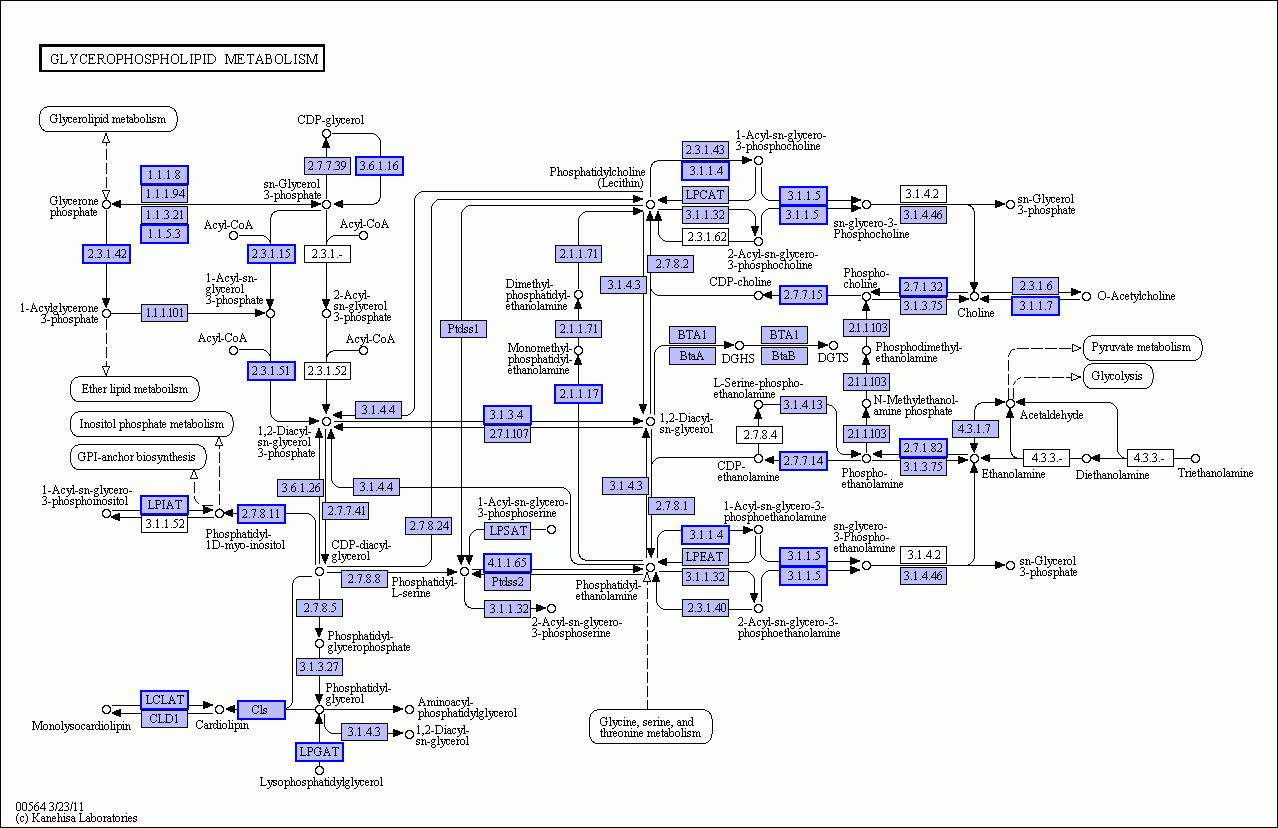

Supplement: Table S4 — KEGG Classification of the unigenes. (ZIP) [file pone.0079516.s004.zip › Kegg/Pathway_Map/ko00564.png]

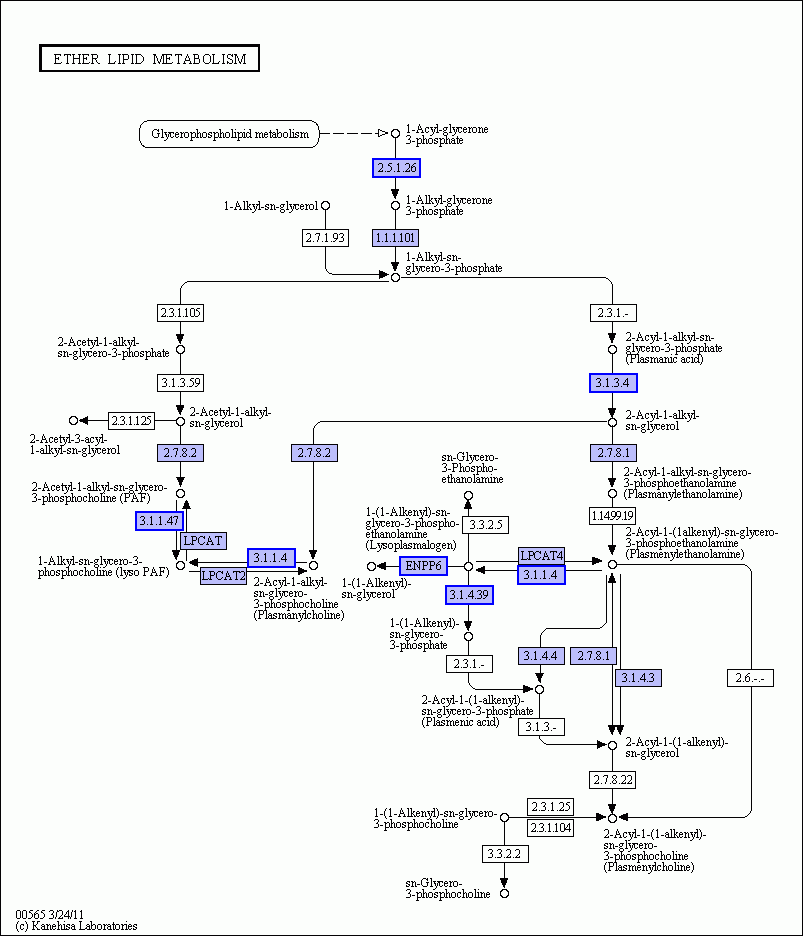

Supplement: Table S4 — KEGG Classification of the unigenes. (ZIP) [file pone.0079516.s004.zip › Kegg/Pathway_Map/ko00565.png]

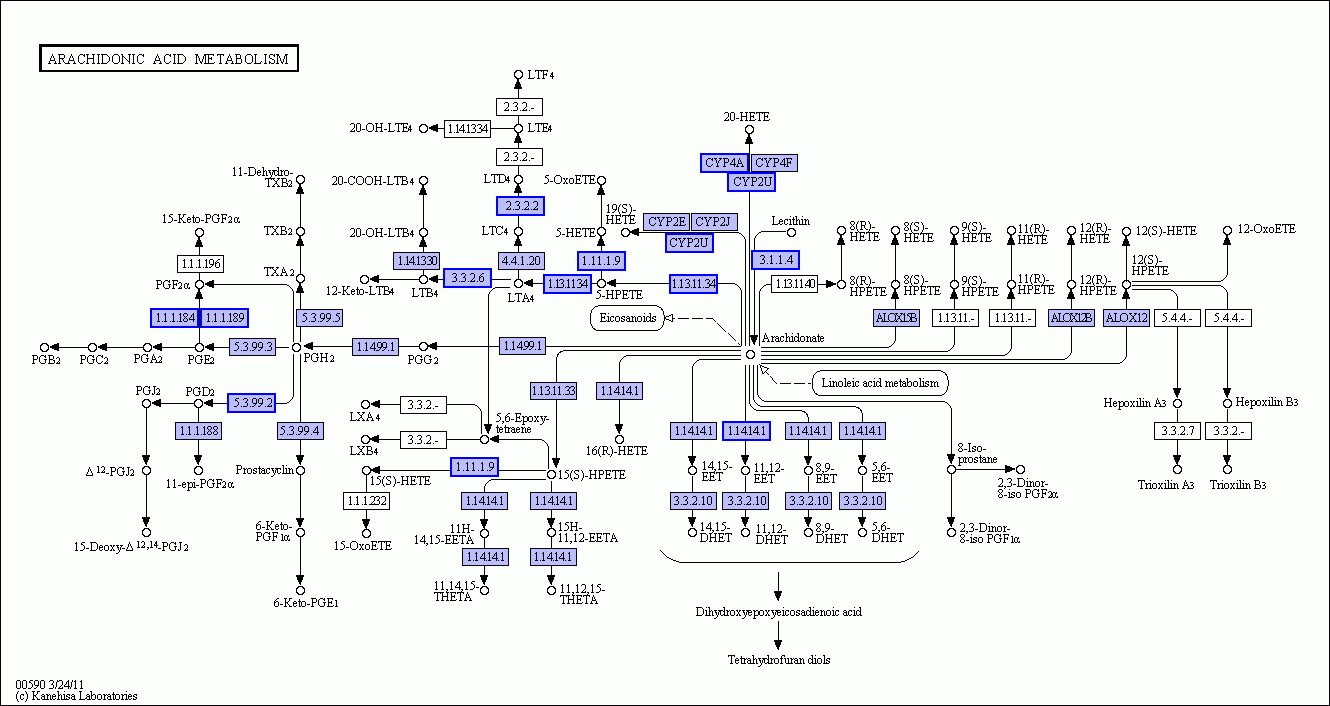

Supplement: Table S4 — KEGG Classification of the unigenes. (ZIP) [file pone.0079516.s004.zip › Kegg/Pathway_Map/ko00590.png]

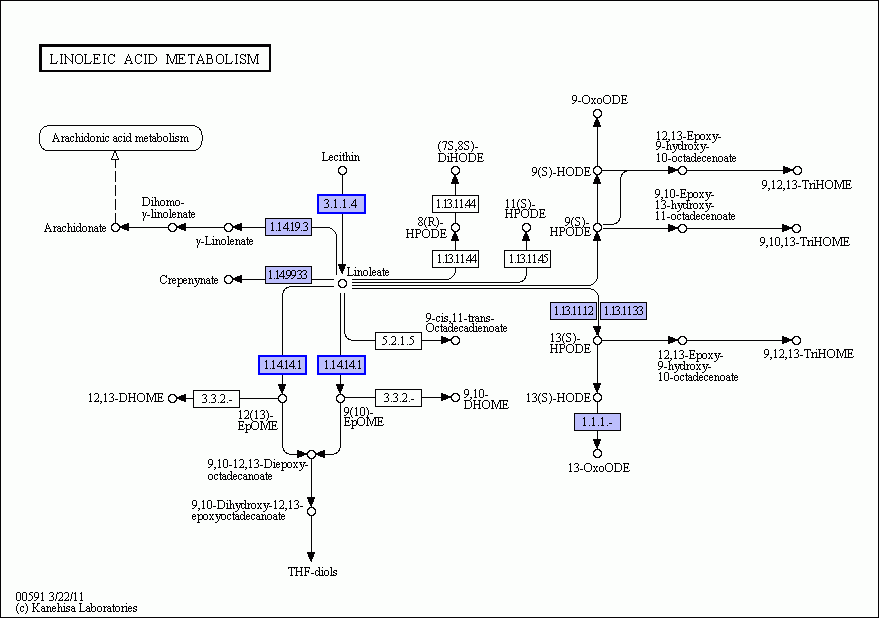

Supplement: Table S4 — KEGG Classification of the unigenes. (ZIP) [file pone.0079516.s004.zip › Kegg/Pathway_Map/ko00591.png]

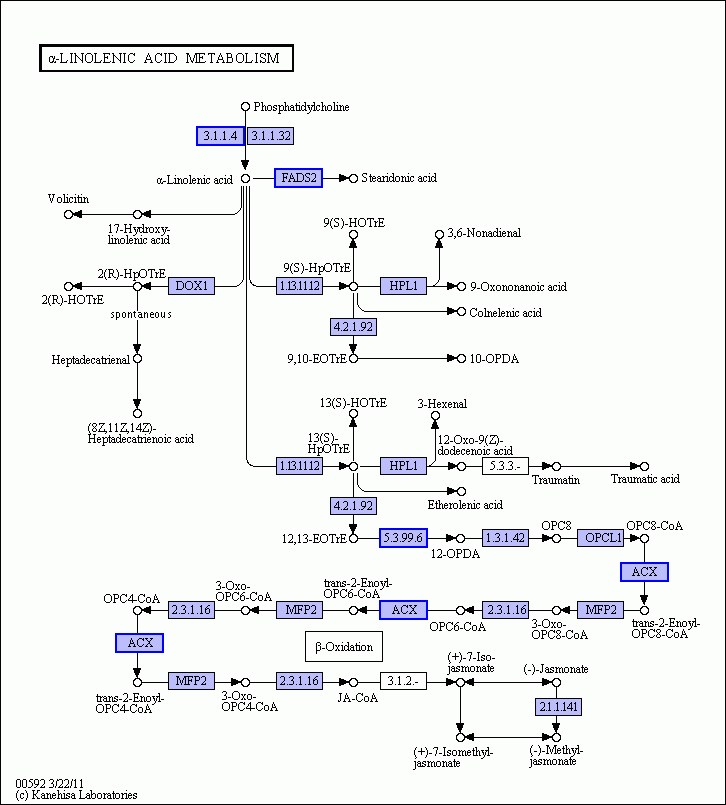

Supplement: Table S4 — KEGG Classification of the unigenes. (ZIP) [file pone.0079516.s004.zip › Kegg/Pathway_Map/ko00592.png]

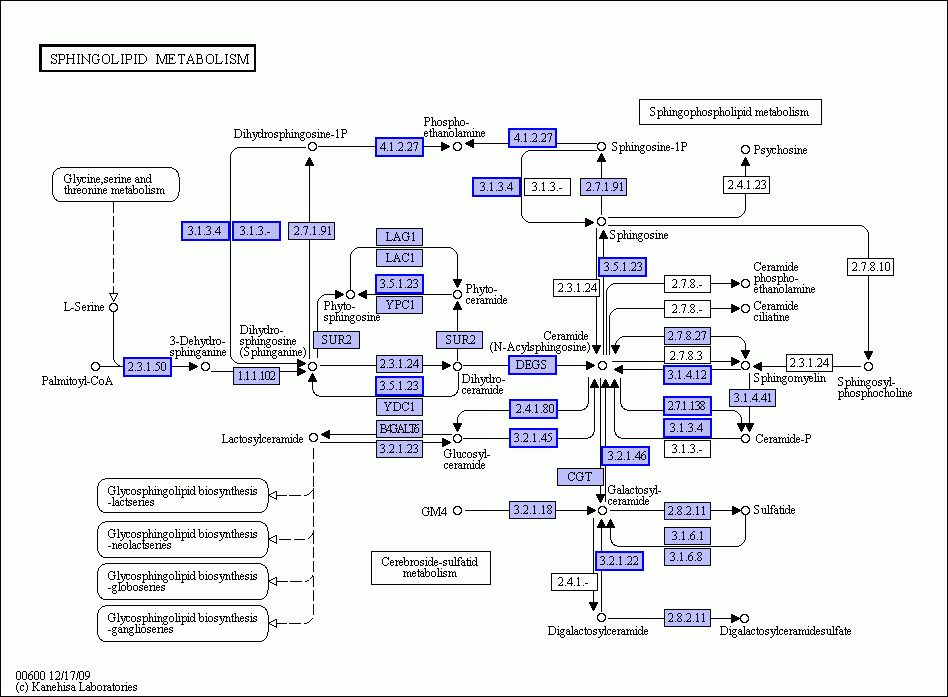

Supplement: Table S4 — KEGG Classification of the unigenes. (ZIP) [file pone.0079516.s004.zip › Kegg/Pathway_Map/ko00600.png]

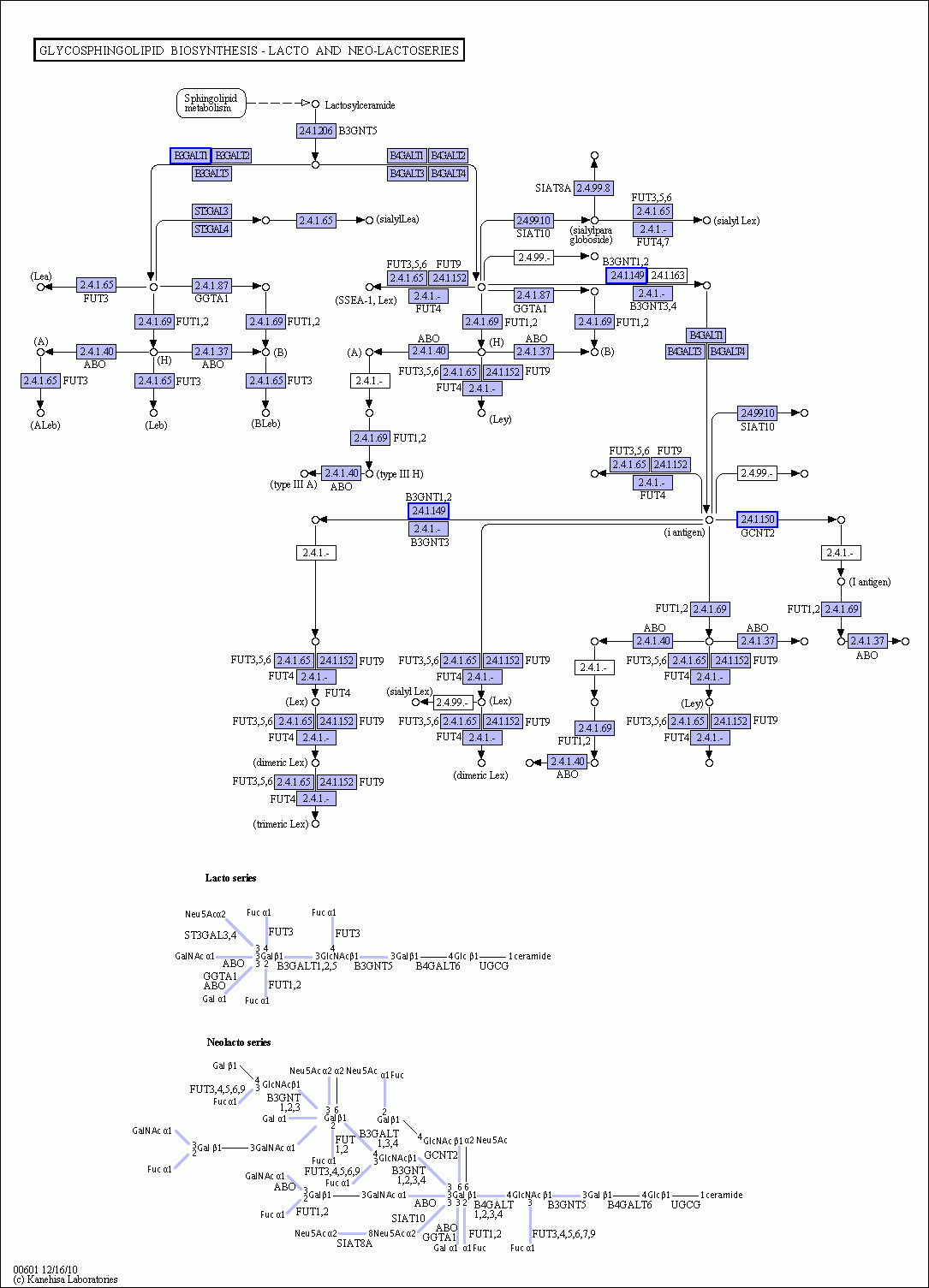

Supplement: Table S4 — KEGG Classification of the unigenes. (ZIP) [file pone.0079516.s004.zip › Kegg/Pathway_Map/ko00601.png]

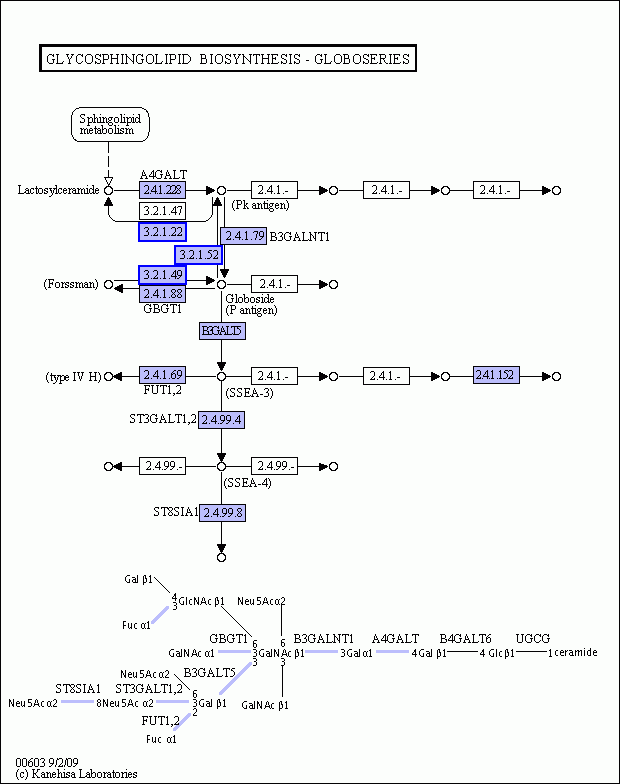

Supplement: Table S4 — KEGG Classification of the unigenes. (ZIP) [file pone.0079516.s004.zip › Kegg/Pathway_Map/ko00603.png]

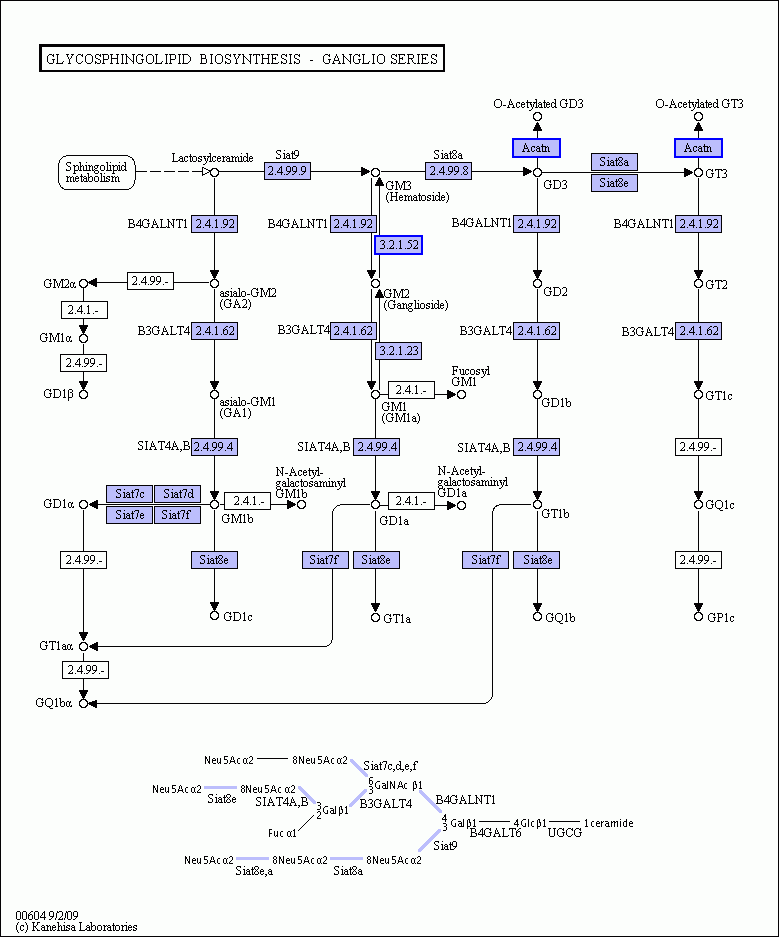

Supplement: Table S4 — KEGG Classification of the unigenes. (ZIP) [file pone.0079516.s004.zip › Kegg/Pathway_Map/ko00604.png]

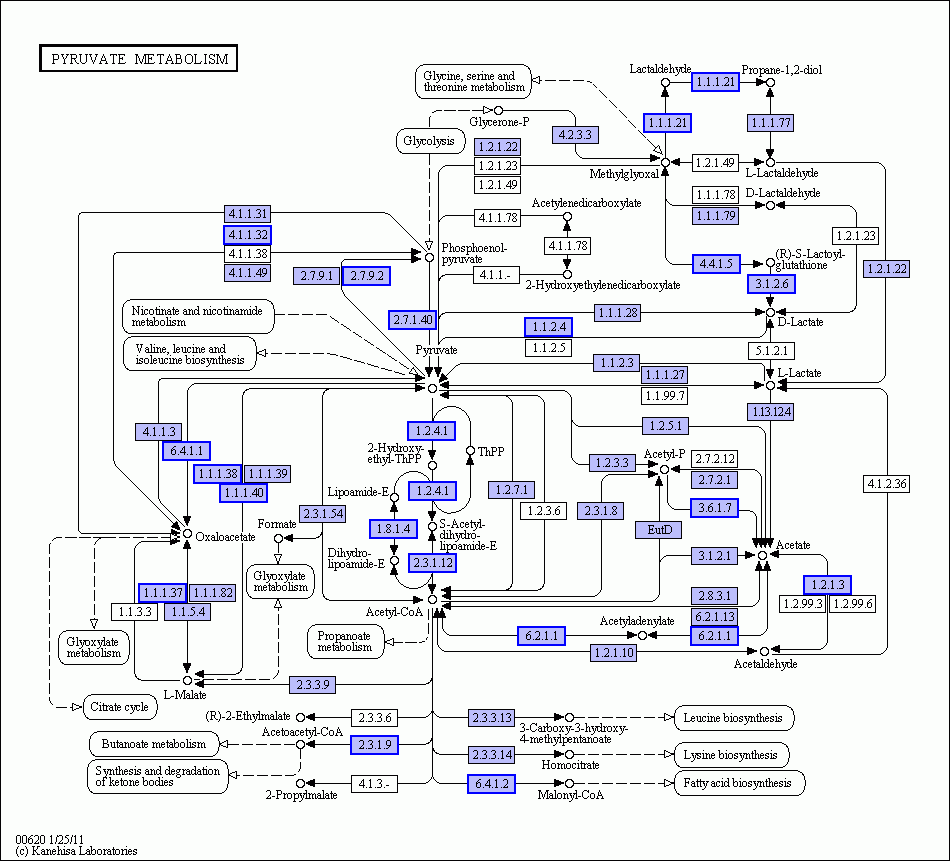

Supplement: Table S4 — KEGG Classification of the unigenes. (ZIP) [file pone.0079516.s004.zip › Kegg/Pathway_Map/ko00620.png]

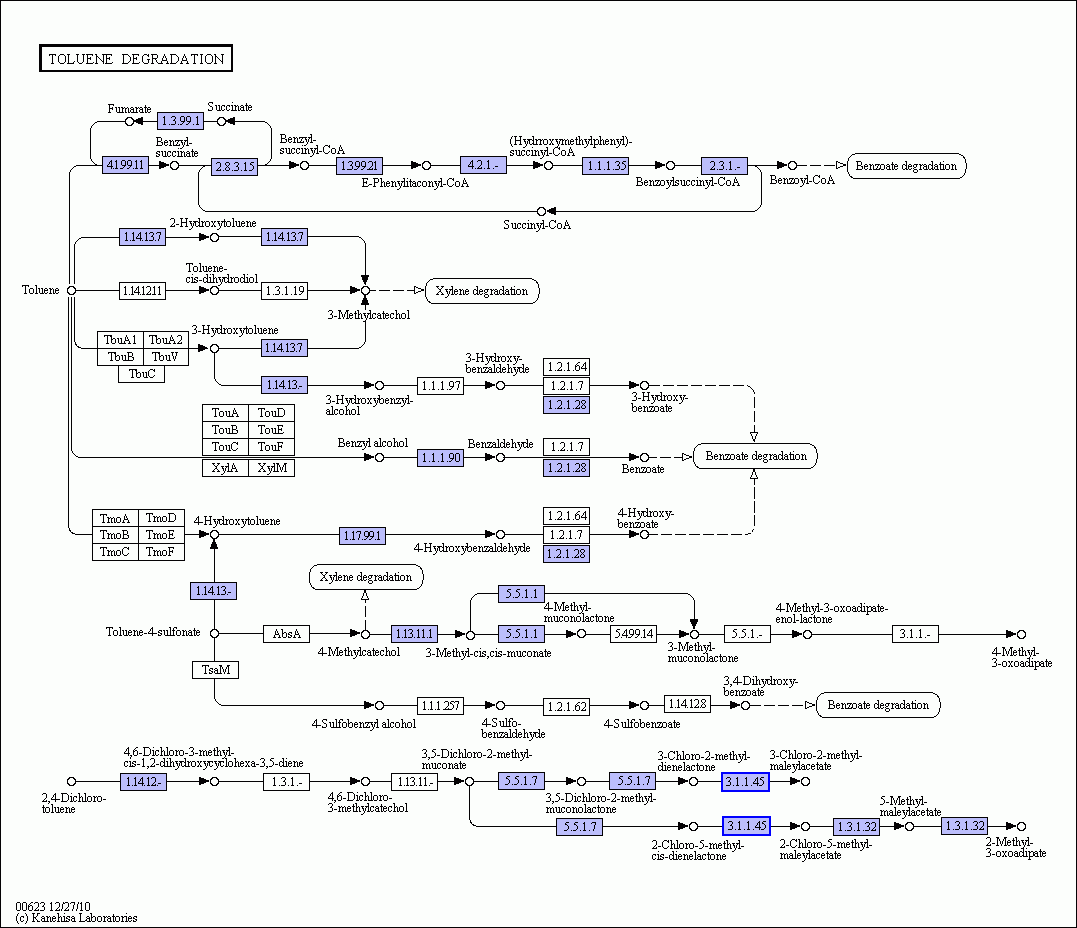

Supplement: Table S4 — KEGG Classification of the unigenes. (ZIP) [file pone.0079516.s004.zip › Kegg/Pathway_Map/ko00623.png]

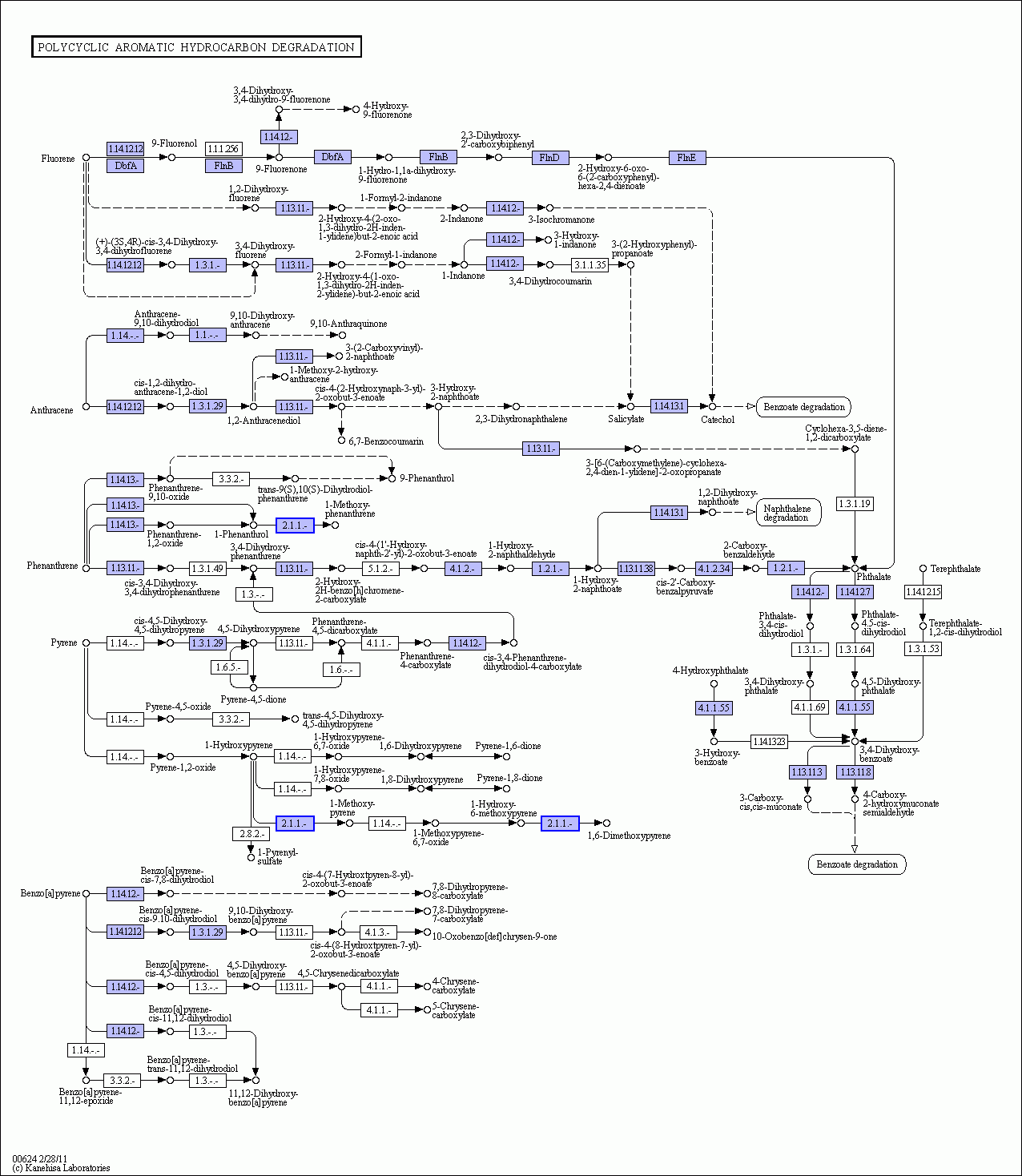

Supplement: Table S4 — KEGG Classification of the unigenes. (ZIP) [file pone.0079516.s004.zip › Kegg/Pathway_Map/ko00624.png]

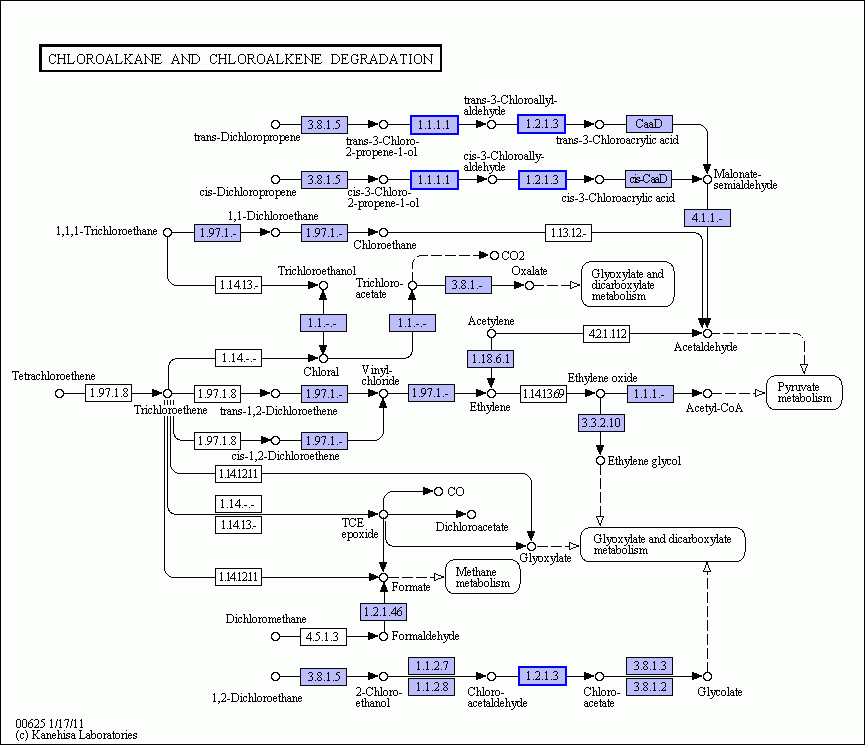

Supplement: Table S4 — KEGG Classification of the unigenes. (ZIP) [file pone.0079516.s004.zip › Kegg/Pathway_Map/ko00625.png]

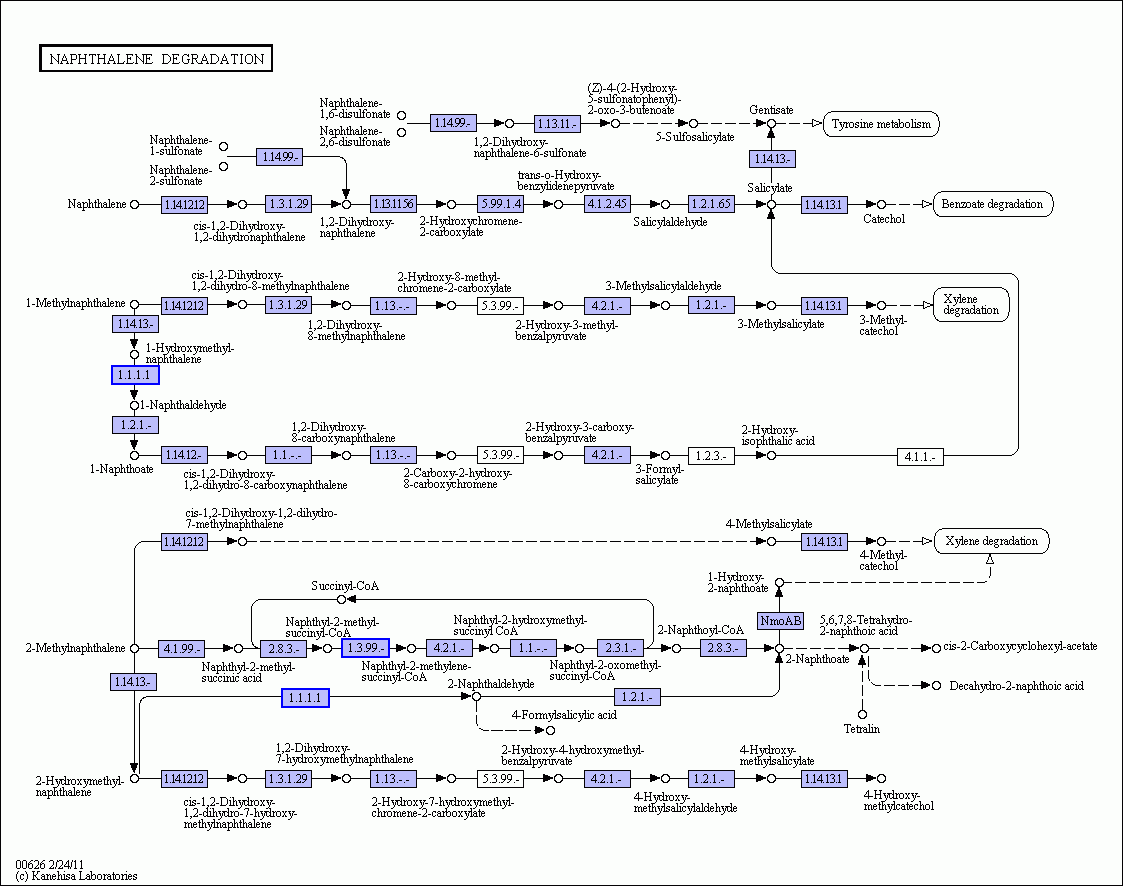

Supplement: Table S4 — KEGG Classification of the unigenes. (ZIP) [file pone.0079516.s004.zip › Kegg/Pathway_Map/ko00626.png]

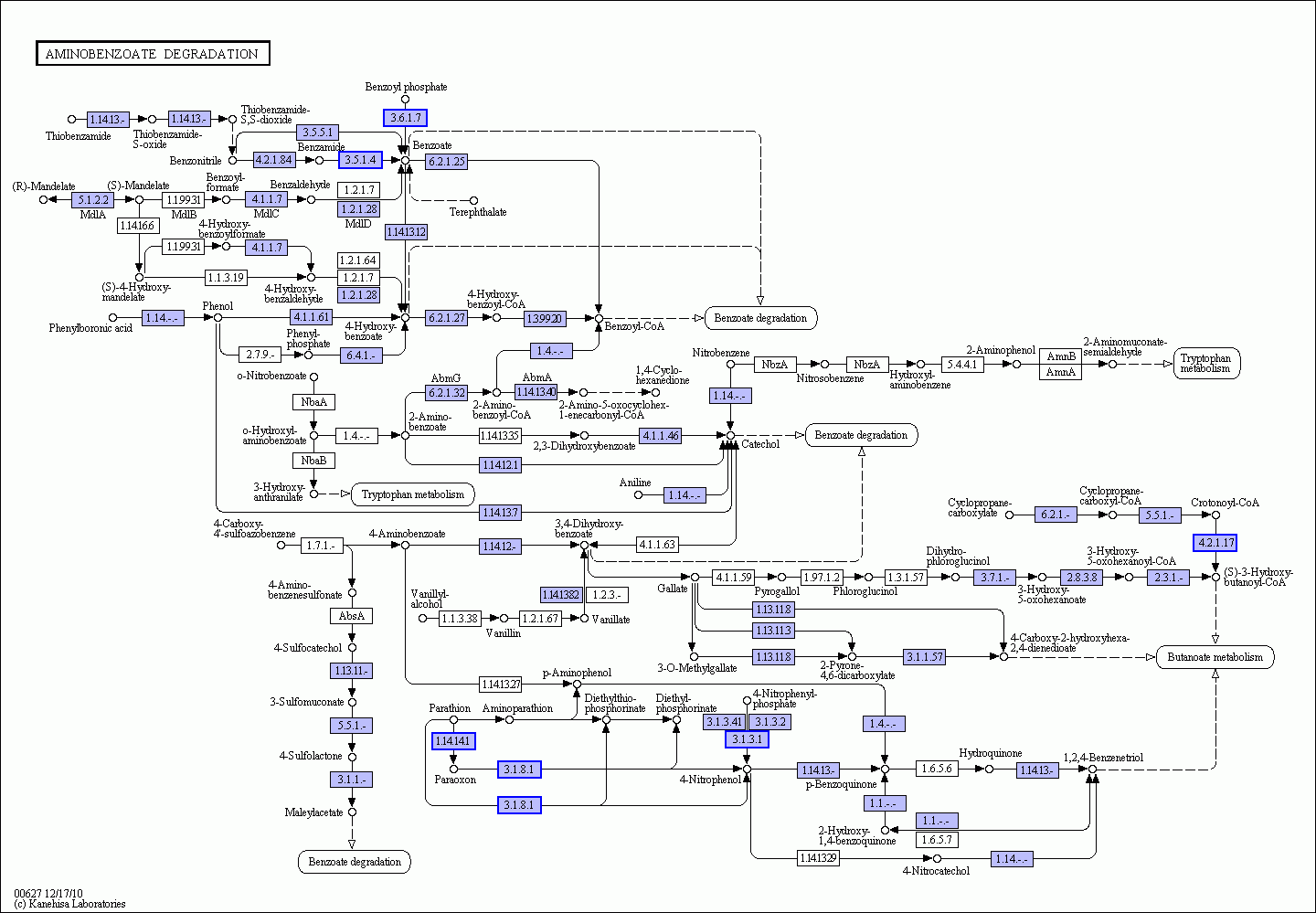

Supplement: Table S4 — KEGG Classification of the unigenes. (ZIP) [file pone.0079516.s004.zip › Kegg/Pathway_Map/ko00627.png]

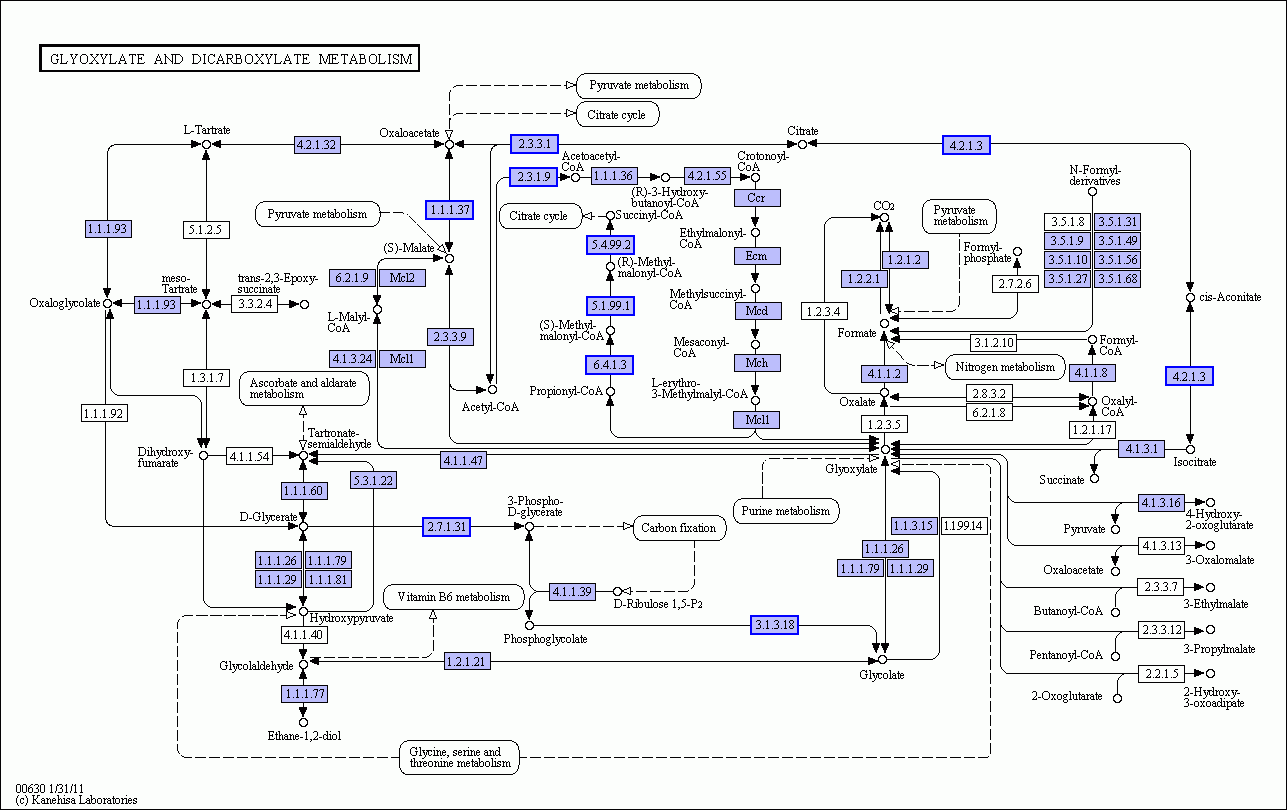

Supplement: Table S4 — KEGG Classification of the unigenes. (ZIP) [file pone.0079516.s004.zip › Kegg/Pathway_Map/ko00630.png]

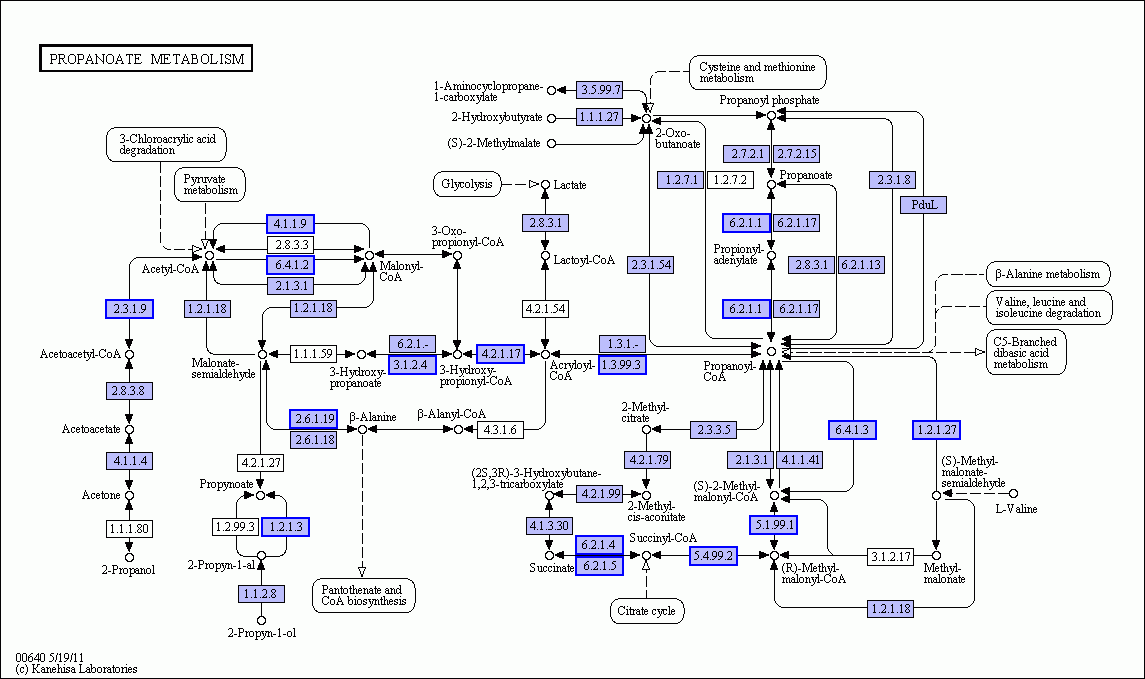

Supplement: Table S4 — KEGG Classification of the unigenes. (ZIP) [file pone.0079516.s004.zip › Kegg/Pathway_Map/ko00640.png]

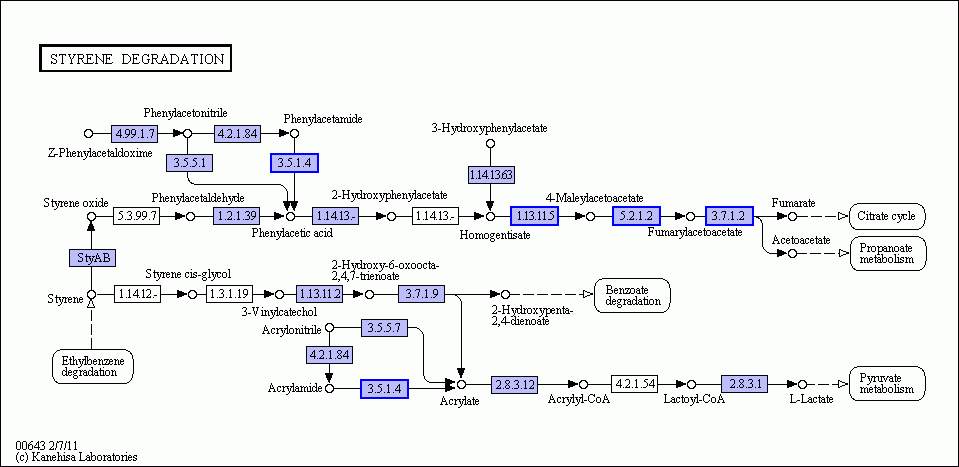

Supplement: Table S4 — KEGG Classification of the unigenes. (ZIP) [file pone.0079516.s004.zip › Kegg/Pathway_Map/ko00643.png]

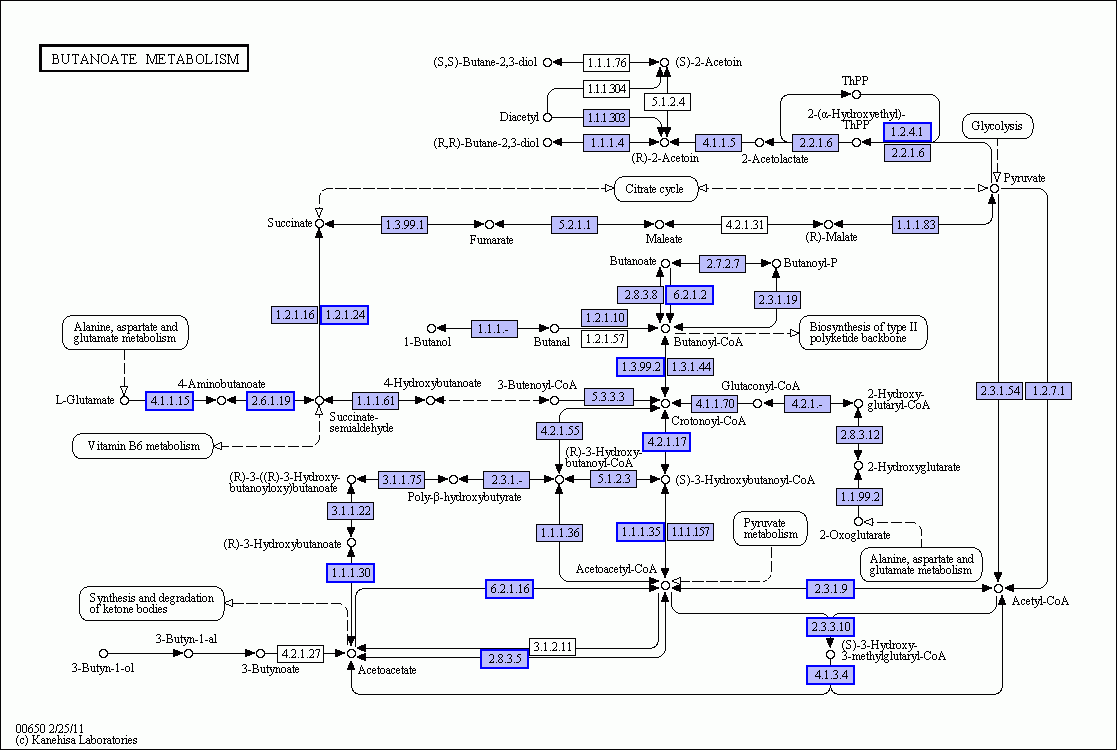

Supplement: Table S4 — KEGG Classification of the unigenes. (ZIP) [file pone.0079516.s004.zip › Kegg/Pathway_Map/ko00650.png]

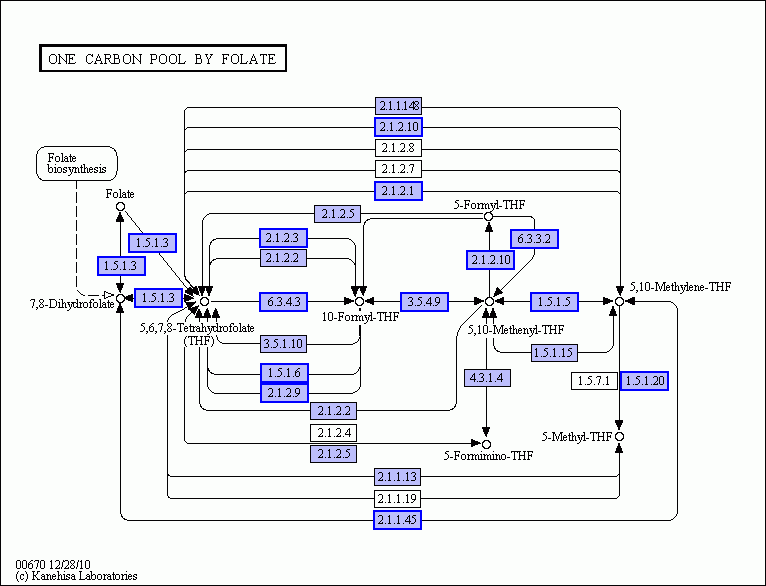

Supplement: Table S4 — KEGG Classification of the unigenes. (ZIP) [file pone.0079516.s004.zip › Kegg/Pathway_Map/ko00670.png]

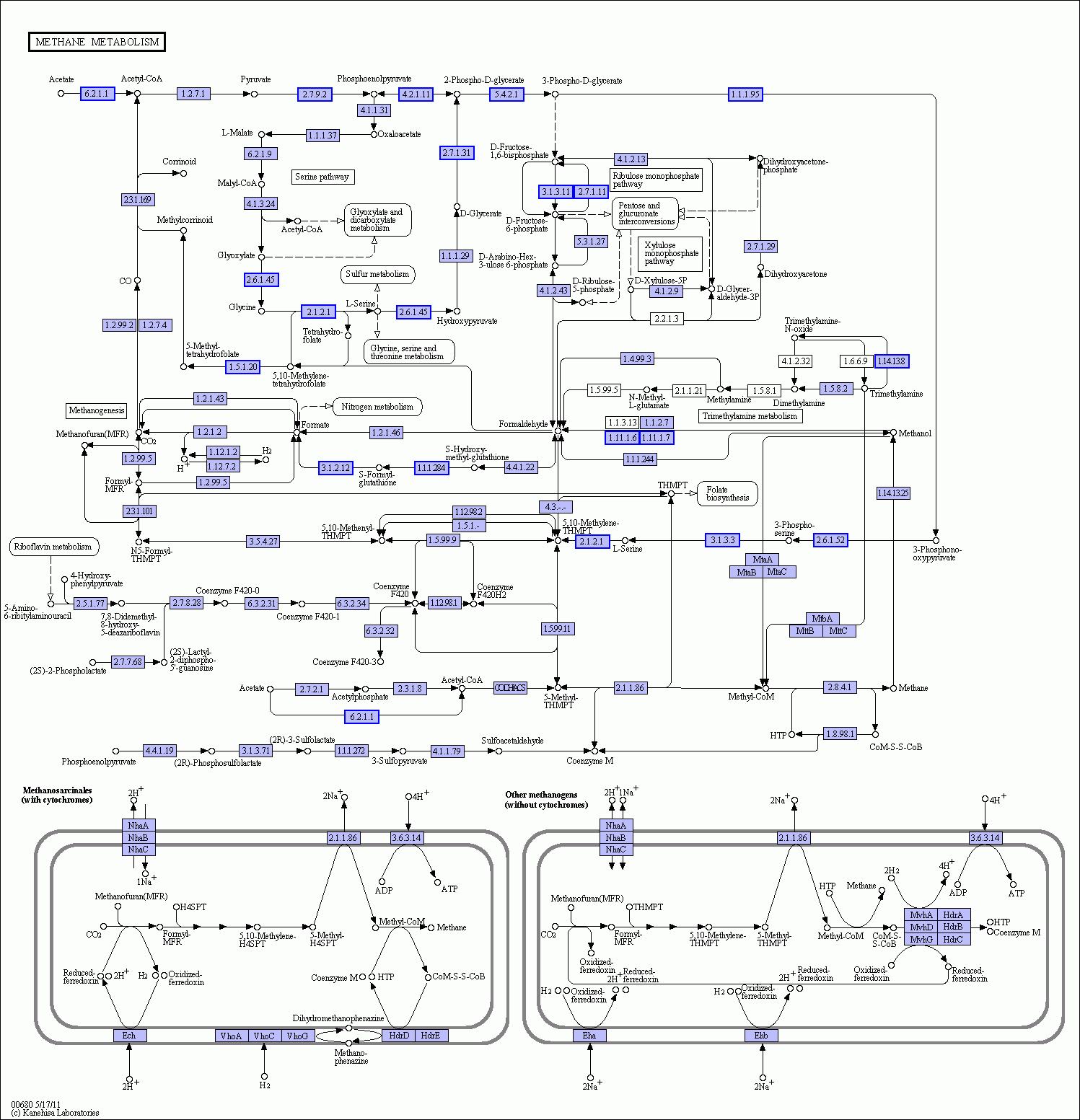

Supplement: Table S4 — KEGG Classification of the unigenes. (ZIP) [file pone.0079516.s004.zip › Kegg/Pathway_Map/ko00680.png]

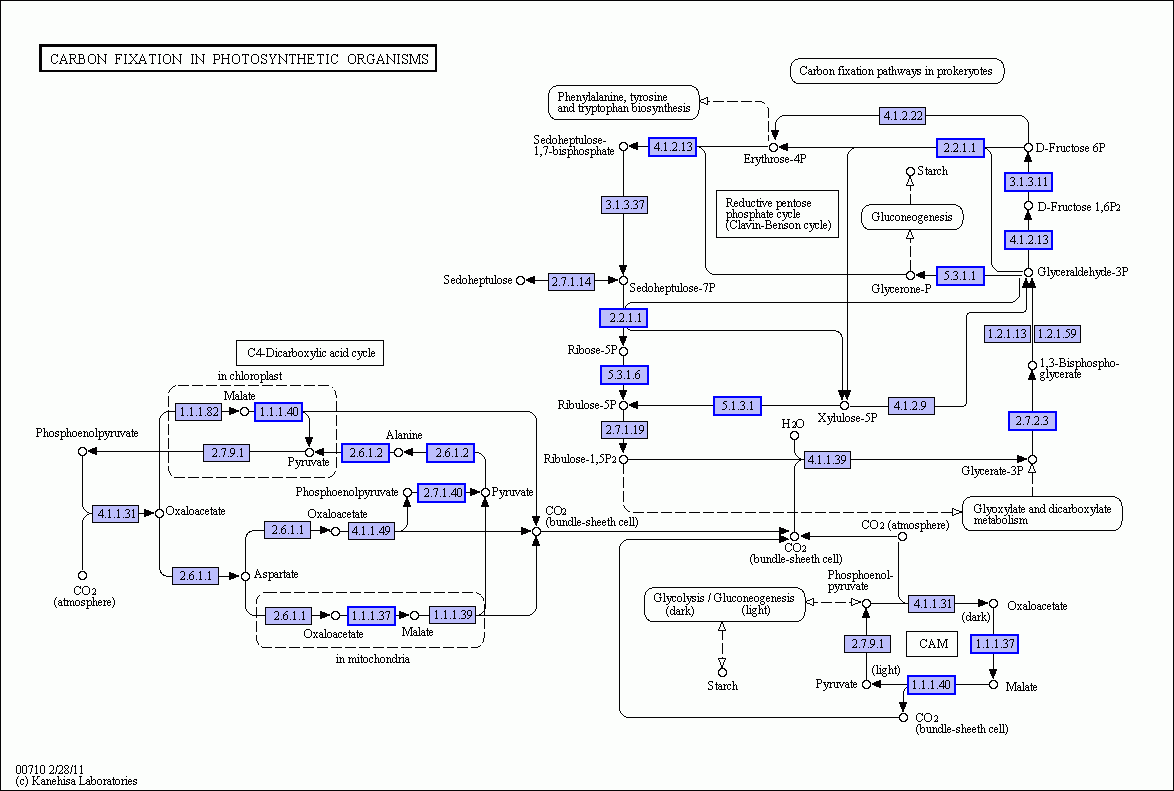

Supplement: Table S4 — KEGG Classification of the unigenes. (ZIP) [file pone.0079516.s004.zip › Kegg/Pathway_Map/ko00710.png]

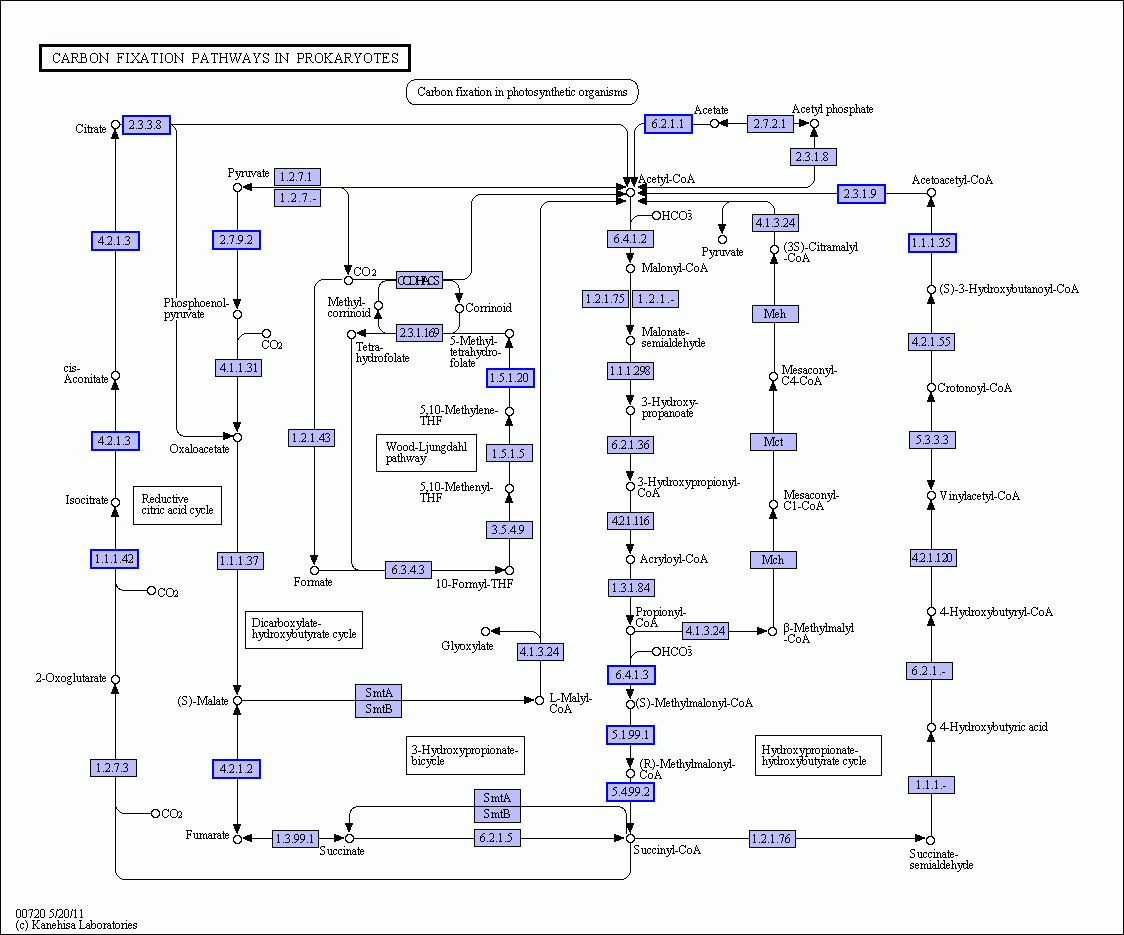

Supplement: Table S4 — KEGG Classification of the unigenes. (ZIP) [file pone.0079516.s004.zip › Kegg/Pathway_Map/ko00720.png]

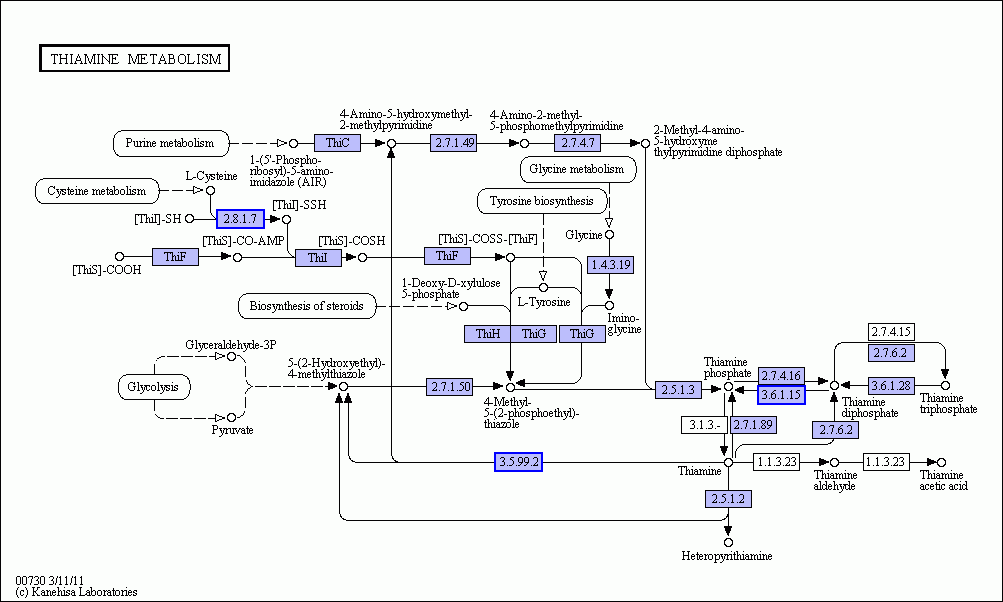

Supplement: Table S4 — KEGG Classification of the unigenes. (ZIP) [file pone.0079516.s004.zip › Kegg/Pathway_Map/ko00730.png]

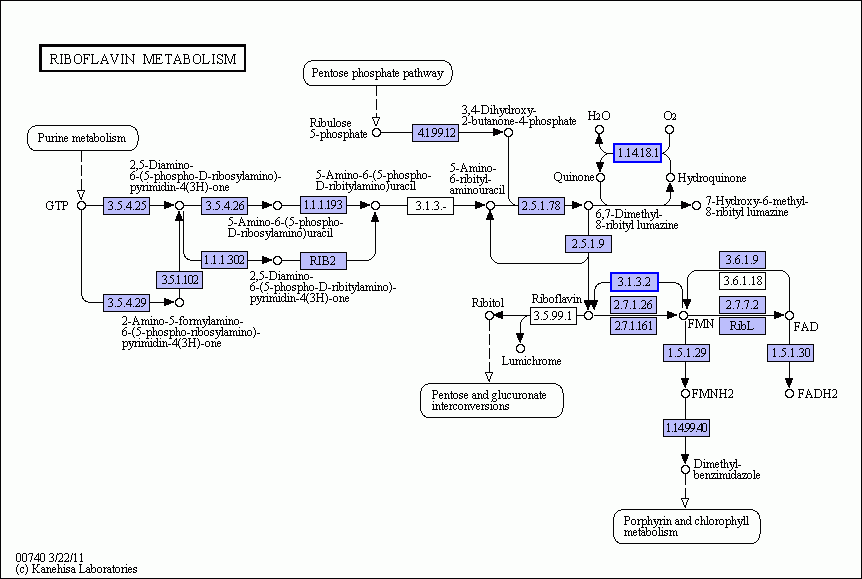

Supplement: Table S4 — KEGG Classification of the unigenes. (ZIP) [file pone.0079516.s004.zip › Kegg/Pathway_Map/ko00740.png]

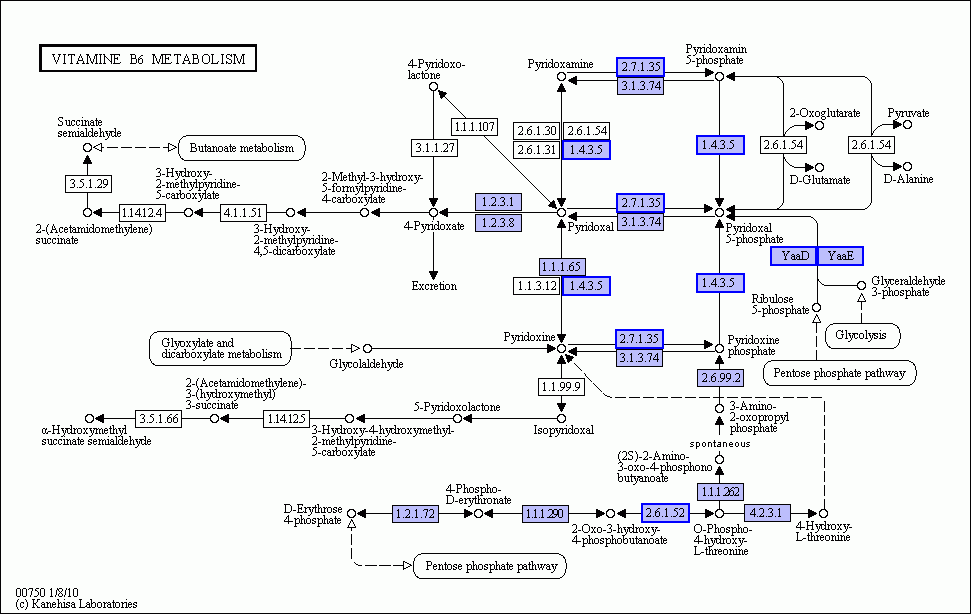

Supplement: Table S4 — KEGG Classification of the unigenes. (ZIP) [file pone.0079516.s004.zip › Kegg/Pathway_Map/ko00750.png]

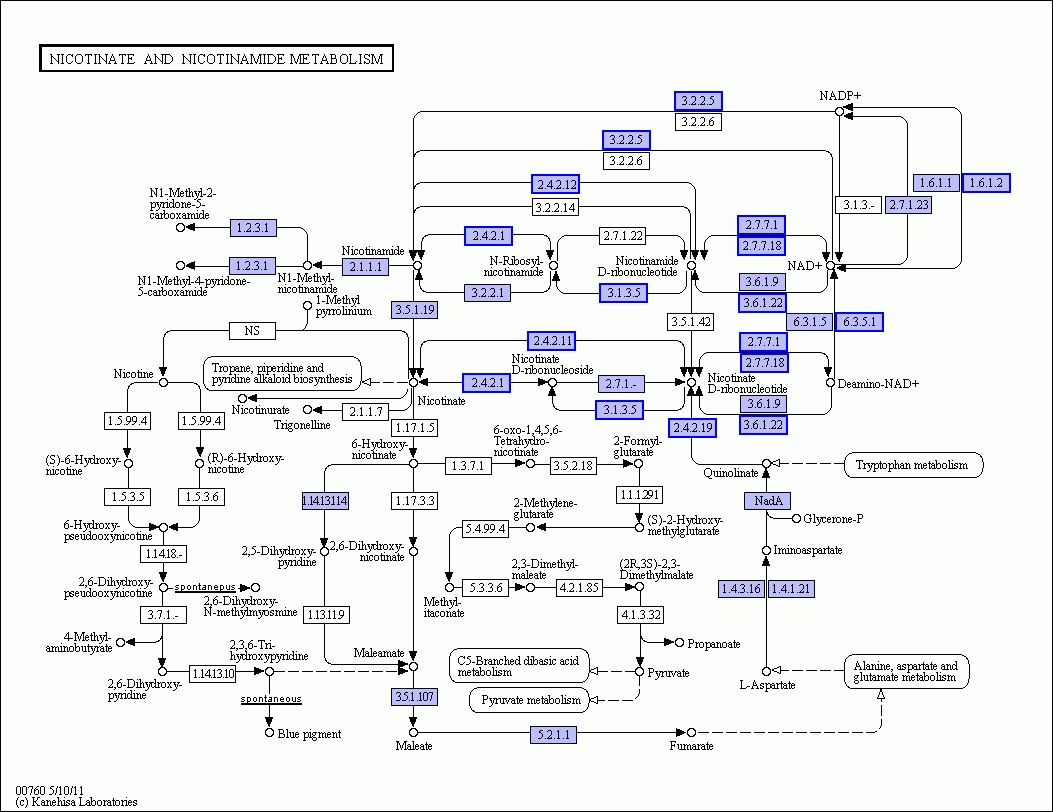

Supplement: Table S4 — KEGG Classification of the unigenes. (ZIP) [file pone.0079516.s004.zip › Kegg/Pathway_Map/ko00760.png]

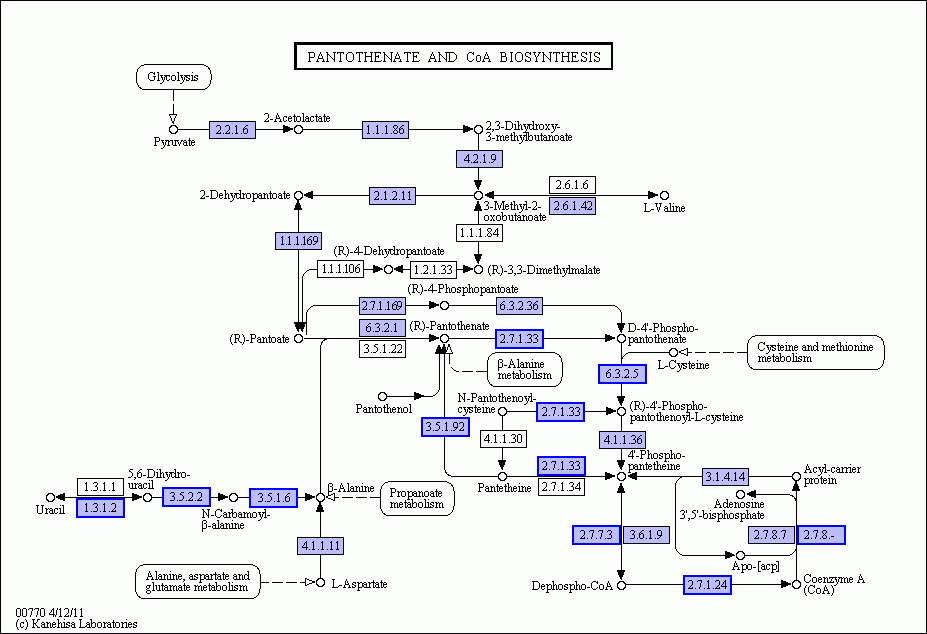

Supplement: Table S4 — KEGG Classification of the unigenes. (ZIP) [file pone.0079516.s004.zip › Kegg/Pathway_Map/ko00770.png]

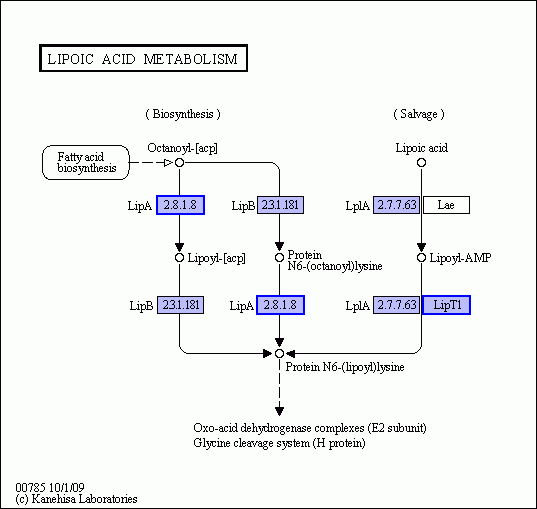

Supplement: Table S4 — KEGG Classification of the unigenes. (ZIP) [file pone.0079516.s004.zip › Kegg/Pathway_Map/ko00785.png]

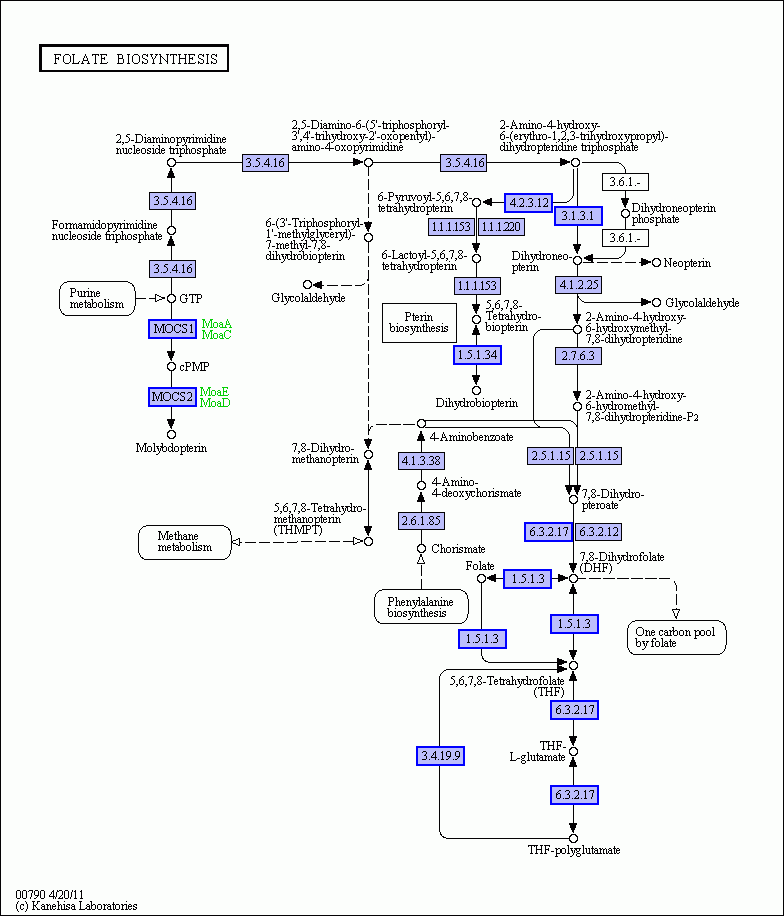

Supplement: Table S4 — KEGG Classification of the unigenes. (ZIP) [file pone.0079516.s004.zip › Kegg/Pathway_Map/ko00790.png]

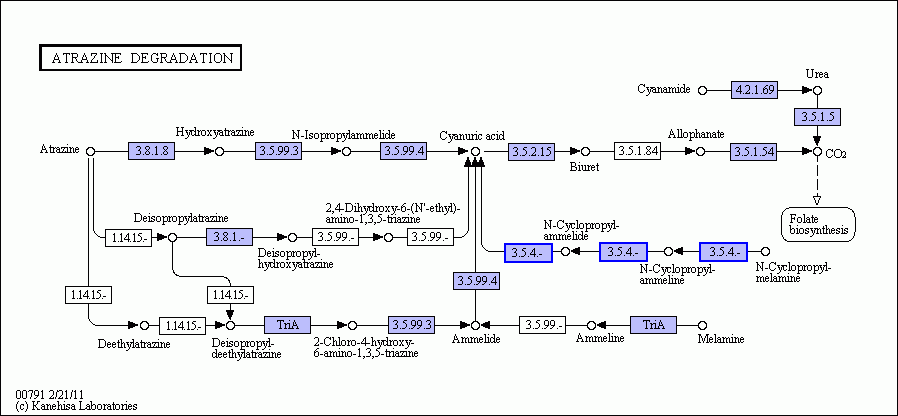

Supplement: Table S4 — KEGG Classification of the unigenes. (ZIP) [file pone.0079516.s004.zip › Kegg/Pathway_Map/ko00791.png]

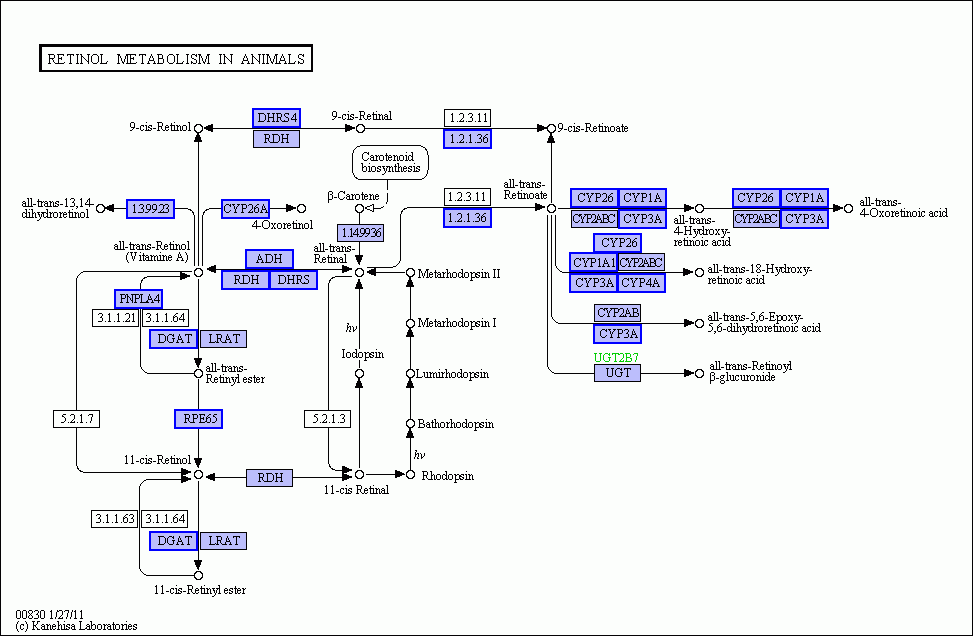

Supplement: Table S4 — KEGG Classification of the unigenes. (ZIP) [file pone.0079516.s004.zip › Kegg/Pathway_Map/ko00830.png]

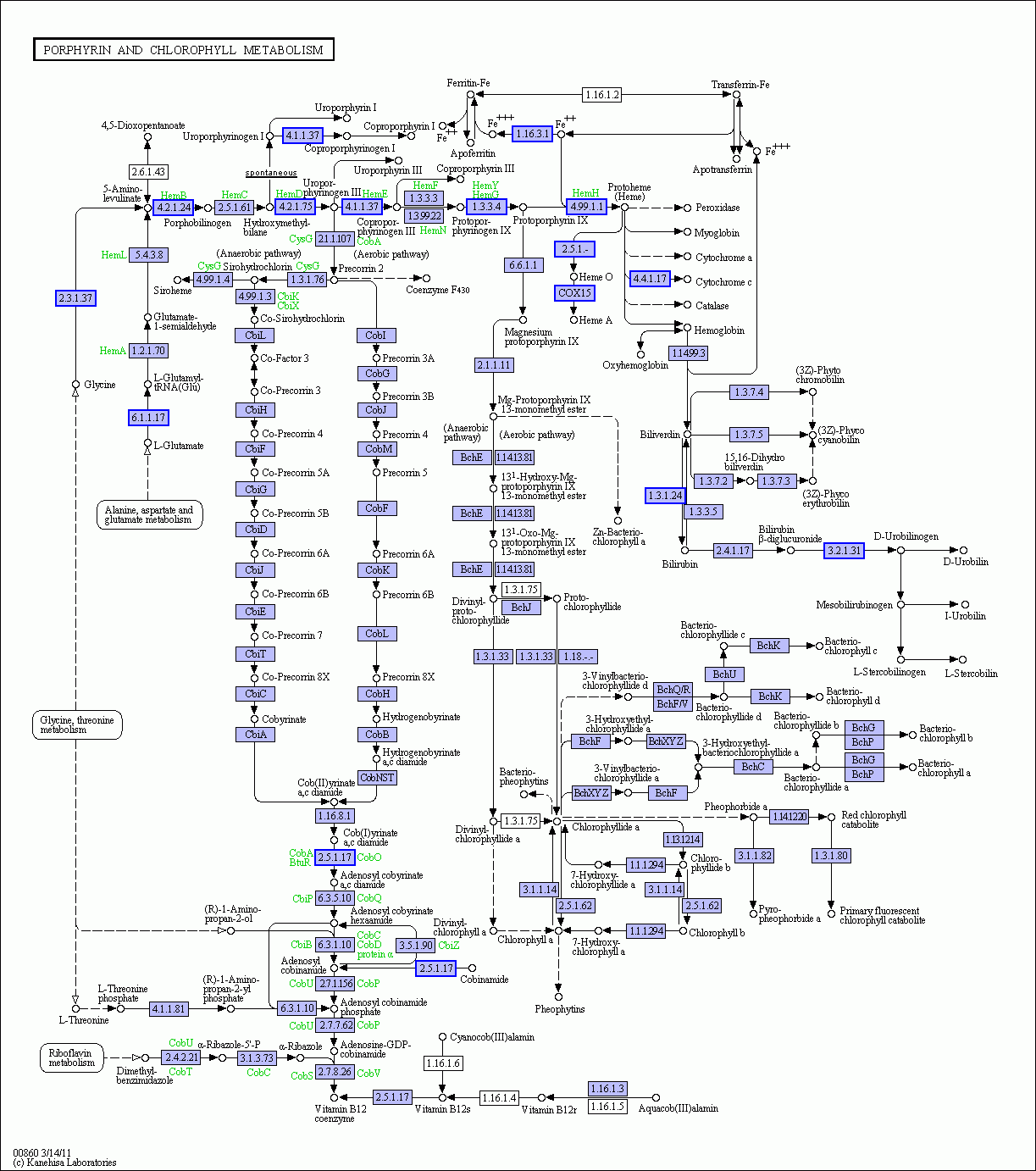

Supplement: Table S4 — KEGG Classification of the unigenes. (ZIP) [file pone.0079516.s004.zip › Kegg/Pathway_Map/ko00860.png]

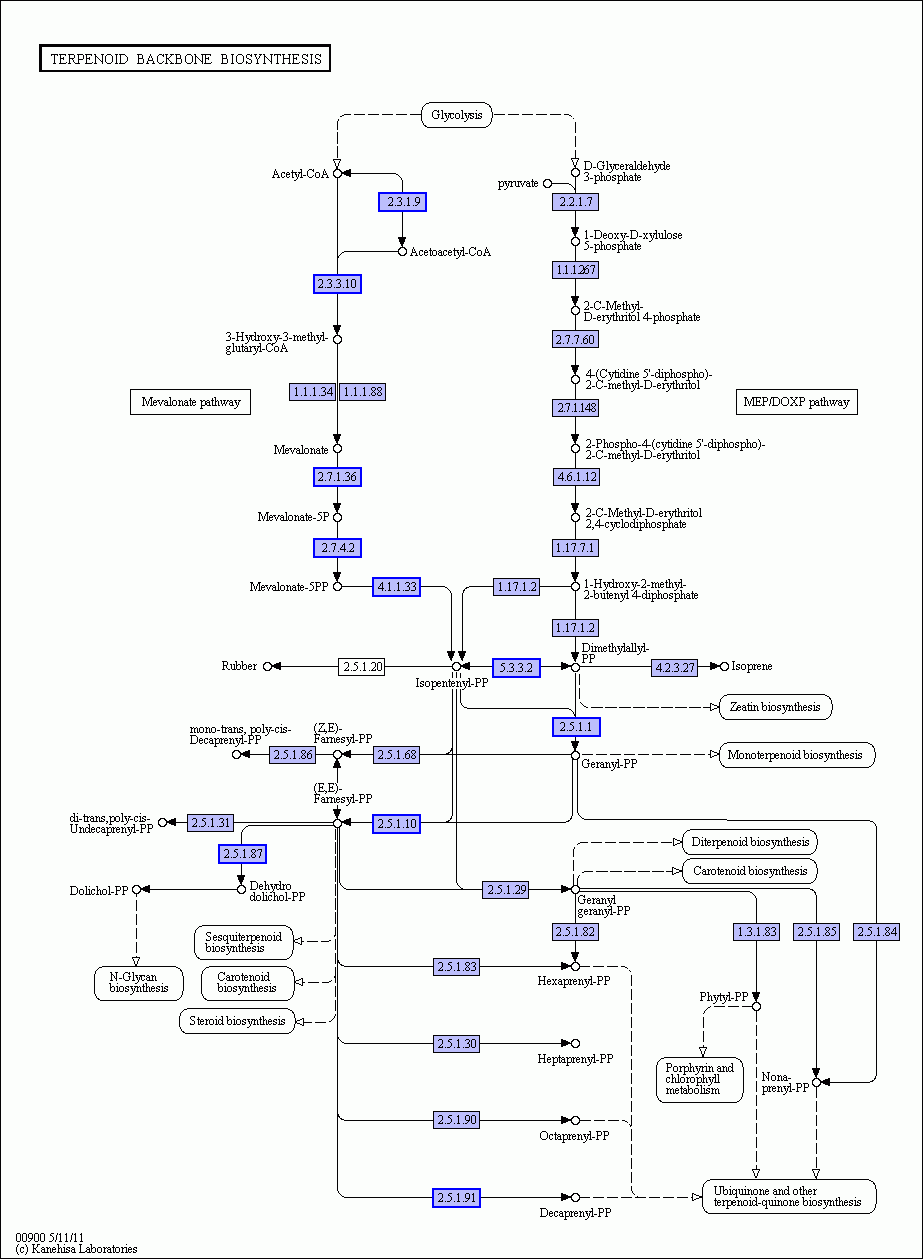

Supplement: Table S4 — KEGG Classification of the unigenes. (ZIP) [file pone.0079516.s004.zip › Kegg/Pathway_Map/ko00900.png]

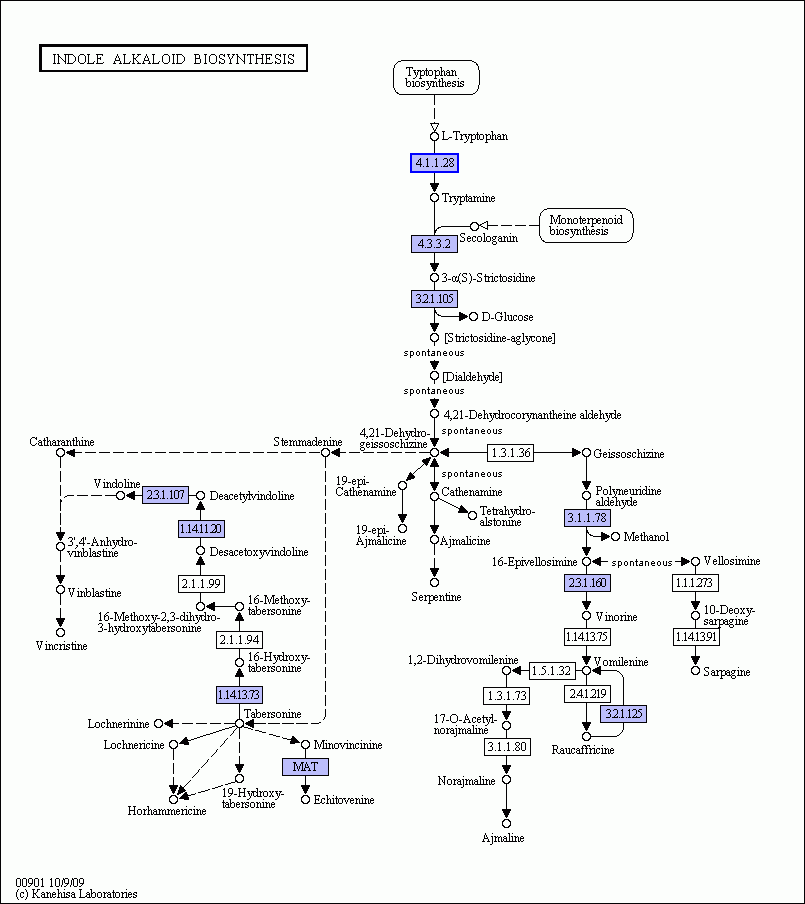

Supplement: Table S4 — KEGG Classification of the unigenes. (ZIP) [file pone.0079516.s004.zip › Kegg/Pathway_Map/ko00901.png]

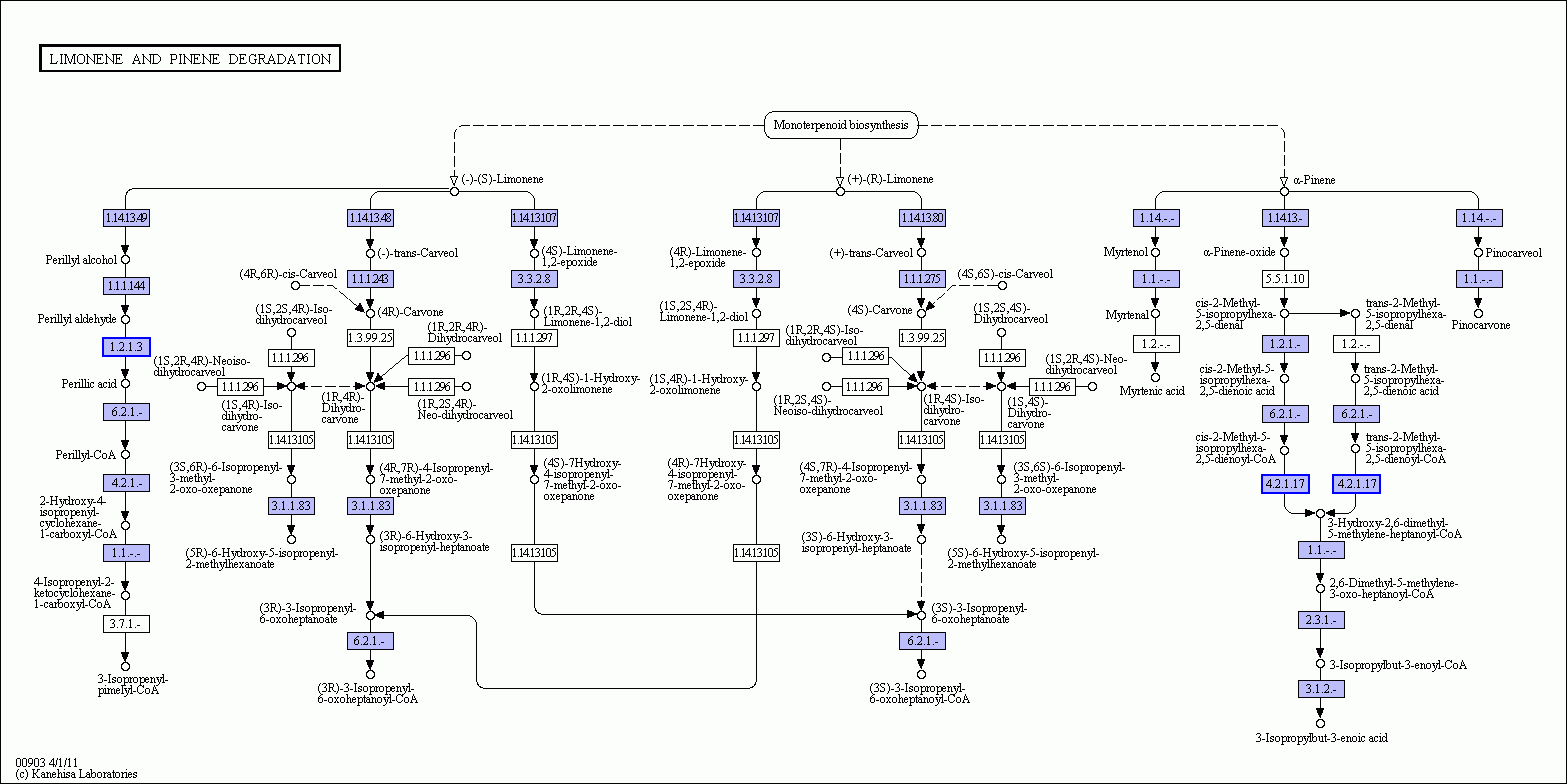

Supplement: Table S4 — KEGG Classification of the unigenes. (ZIP) [file pone.0079516.s004.zip › Kegg/Pathway_Map/ko00903.png]

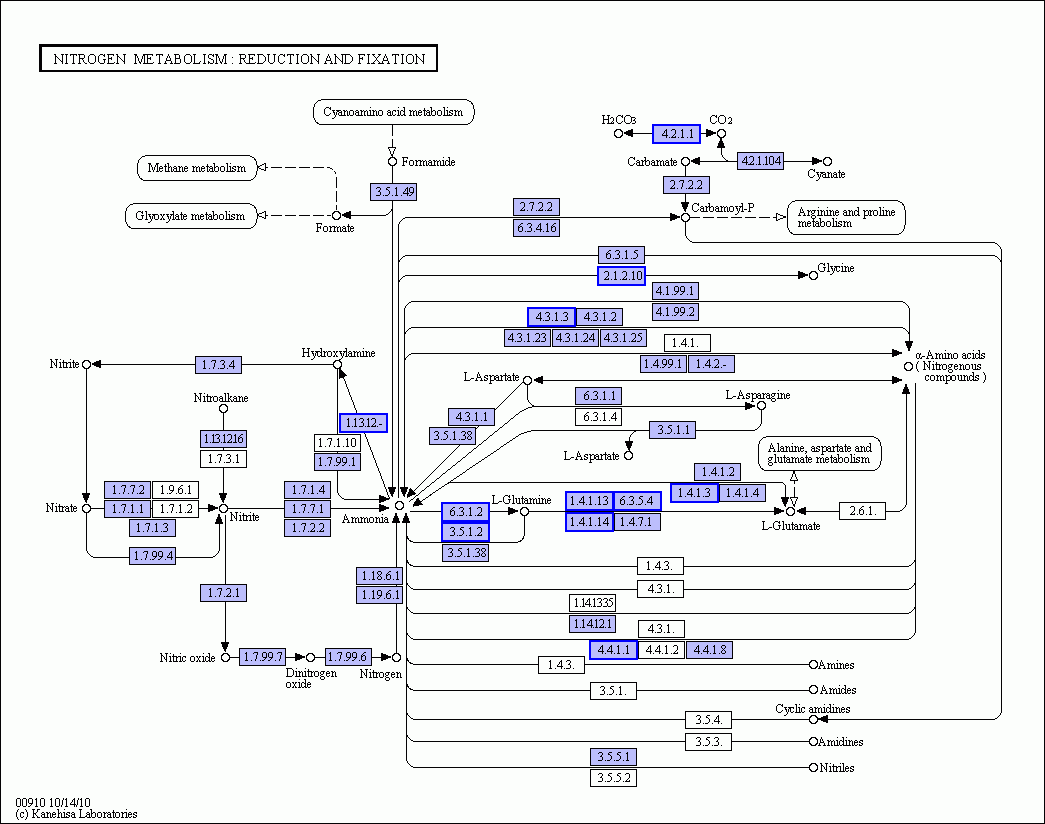

Supplement: Table S4 — KEGG Classification of the unigenes. (ZIP) [file pone.0079516.s004.zip › Kegg/Pathway_Map/ko00910.png]

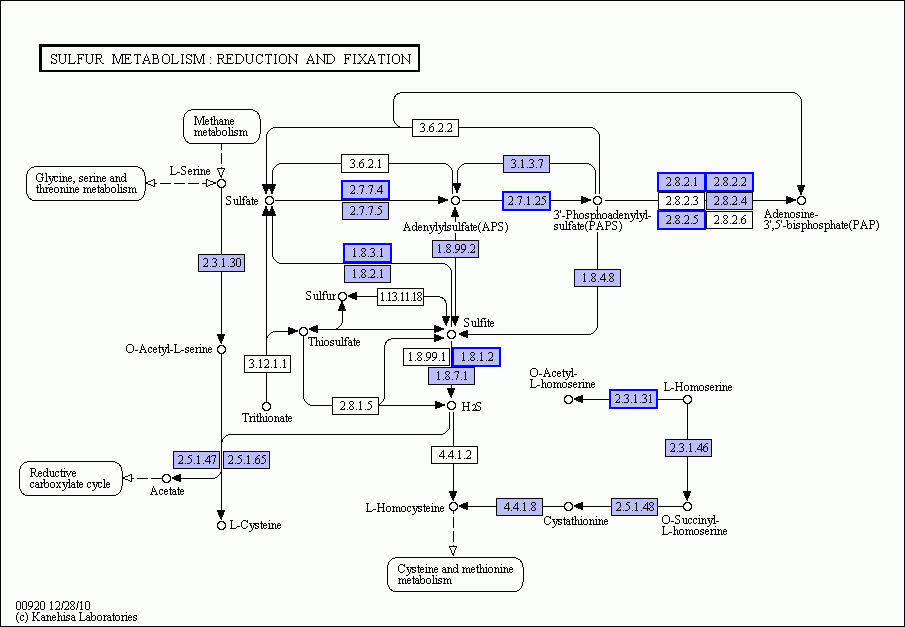

Supplement: Table S4 — KEGG Classification of the unigenes. (ZIP) [file pone.0079516.s004.zip › Kegg/Pathway_Map/ko00920.png]
