# Supplementary material for: Sequencing and De Novo Assembly of the Asian Clam (Corbicula fluminea) Transcriptome Using the Illumina GAIIx Method
Source: PLoS One. 2013 Nov 7;8(11):e79516. doi: 10.1371/journal.pone.0079516 (PMC3820681; doi:10.1371/journal.pone.0079516)
Supplement: Table S6 — The RT-PCR primers and Sanger sequencing of the 15 analyzed genes. (ZIP) [file pone.0079516.s006.zip › Table S6.docx]

Table.S6. RT-PCR Primers and Sanger sequencing of the 15 analyzed genes.

| **1.**  **>Locus_507_T2 Gene:*Cf(Cu/Zn)SOD*, Accession No. KF218347, size 234**  **RT-PCR Primers:** **F:AATGGATCGTTCTCCTGTCAAT**  **R:GAAATAACTGGTCTGGCTGCTG** |
| --- |
| GAAATAACTGGTCTGGCTGCTGGAGAACATGGCTTTCATGTGCATGAATTTGGTGACAATAGCAATGGTTGCACAAGTGCAGGAGCTCACTACAATCCAGACAAGGTGGACCATGCTGGACCAGAAGACGCTGTCAGACATGTTGGAGACCTAGGGAATGTTGTGGCTGATGAGGCTGGATGTAAGGTGGATATCACAGACAAAGTGATCACATTGACAGGAGAACGATCCATA |
| **2.**  **>** **Locus_937_T2 Gene:*CfGPx-A*, Accession No. KF218345, size 372**  **RT-PCR Primers:** **F:GGATTGGGAAAGATGGTTGGG**  **R:CAGTTGTTGCTGGGTAGAGC** |
| GGGATTGGGAAAGATGGTTGGGCCATTTTGTTCTCTCACCCAGCTGATTACACCCCAGTATGTACAACAGAGCTGGGTATGGTTAACAAGATGACAGACAAATTCAAGGCAAAGAATGTAAAACTGATTGCCCTGTCCTGTGATGATGTGGAGAGCCACAAAGGCTGGACGAAGGACATCTTGGACTATGCAGGCACGTCCCAGTTTGACTTCCCAATCATTGCTGATCCAAAGCGGGACCTTGCTGTACAGCTTGGAATGCTCGACCCTGATGAGAAAGATGCCGCAGGCCTGCCACTCACTTGCAGAGCTGTATTTATCATTGGTCCTGAATACAGACTGAAGTTGTCTATGCTCTACCCAGCAACAACT |
| **3.**  **> Locus_2050_T1 Gene:** ***CfGST-mu*, Accession No. KF218346, size 369**  **RT-PCR Primers:** **F:CTGCGTGCCAATGTTCTTGA**  **R:GCCCAGCCAATCCGTTTATT** |
| GCCCAGCCAATCCGTTTATTGCTCACCTACGTAGGCGAAGATTTCACGGACACCCAATACGAACAAGGCGATGCACCTGAATTCAGTCGAGATGCATGGTTTAGCGTCAAACCAAAATACGCAGAGTTCCTTGACTTTCCAAATTTACCTTACTACATAGACGGTGACGTGAAGATCACACAGAGCAACGCCATATTGAAACACATTGCCAGAAAACACAAGCTGGATGGTGAAACAGAGAGGGATAAGGCCATAGTCGACATGCTTCTAGAGCAAGCAATGGACTTAAGGAACGGCGTTGTTAGGCTCTGTTATAATCCAGATTATGAGAATCTGAAGGTAGACTATTTCAAGAACATTGGCACGCAG |
| **4.**  **> Locus_9557_T1 Gene:** ***CfTPX1*, Accession No. KF218348, size 393**  **RT-PCR Primers:** **F:CCAGATAGACACCGTAAGCCT**  **R:GCACAGCAAAGTGGAAGGAG** |
| GCACAGCAAAATGGAAGGAGATCGCTGGGTCACTCTTTACAATGGAGCCAAGCCATCATTTCAAAACCAGCTCCTGATTGGAATGGAACTGCTGTTGTTGATGGAAAGTTCAAGGAAATCAAGCTCCAGGATTACAAAGGAAAATATTTAGTGTTTTTCTTCTATCCTCTGGACTTTACTTTTATCTGTCCGACTGAAGTTGTGGCATTTAATGAAAGACTGGAAGAGTTCAAGAAAATCAACACGGAAGTTGTAGCATGTTCTGTAGATTCTCCACATACTCATCTTGCATGGATCAACACTCCAAGATCCAGTGGTGGGCTCGGATCCATGAAGTACCCTCTGTTGTCAGATATTACACATGCAATTTCAAAGGCTTACGGTGTCTATCTG |
| **5.**  **> Locus_9742_T3 Gene:** ***CfTPX2*, Accession No. KF218349, size 273**  **RT-PCR Primers:** **F:CTGCTCCAGGCTTCCAGTT**  **R:CCGAGAAAACAAGGTGGTCTG** |
| CCGAGAAAACAAGGTGGTCTGGGTAGTATGAACATCCCCTTGATAGCAGATAAGACCCAAGAGATTTCAAGGACATATGGCTGTCTCACGGAGGATGAGGGAGTTCCATTTAGAGCACTTTTCATCATTGACGATAAAGGCAATCTTCGCCAGATCACCATCAATGACCTTCCAGTGGGACGTAACGTCGATGAGGTGCTGAGACTTGTGCAAGGCTTCCAGTTCACTGATAAACACGGAGAAGTGTGCCCTGCAAACTGGAAGCCTGGAGCA |
| **6.**  **> Locus_5127_T1 Gene:** ***CfCYP4*, Accession No. KF218340, size 459**  **RT-PCR Primers: F:GCTGAAACAGGCGAGAGGTT**  **R:AAGAAATGGCACTGGCGGT** |
| TGGCTGAAACCAAGCGAGAGGTTTGAGGTCTTCGAGTACGTATGTTTATGTACCTTGGAGATCATCCTCAAGTGTGCCTTCTCTTACAACAAGGACGTCCAAAGTGCAGGGGACACAAACCCATATGTCAAAGCTGTAAACAGAATCTCAGATAGTGTTCAGTACAGATTTATGAACCCGCAGTTGTTATGGGATTTCTTATGGTACCGGTGTAAAGTTGGAAAACAGTTCAAAGAAGATTGTGATTTCGTGCATTCGGTAGCTGAAGACATTATTGACAGAAGAAAAGAAACATTGGATAAAACTGGTCTTAACAAAGAAGATCGTTATATCGACTTCCTCGACATATTATTGACGGCAAAAGACGAAGAAGGACGTGGTTTATCAAGAATGGAAATCAGAAATGAAGTGGACACTTTCTTGTTTGAAGGCCACGACACAACCGCCAGGCCCATTTCT |
| **7.**  **> Locus_2545****_T1 Gene:** ***CfCYP30*, Accession No. KF218341 size 612**  **RT-PCR Primers: F:CAAGTTTACCGACAGGGTGC**  **R:CAGTGTGTTGGCTGCTTCAG** |
| TCAAGGTTTAACGACAGGGTGCGTGTCCTTCCTTTAACGGAGAGATGGGAATCTACGGAAGAGGGATCTGCTGTTTCTGTAGCTGAGGGGGAACACTGGAGATTTCTCCGAGGCACGCTCAGTCCCACATTCACTTCAGGAAAAATGAGAGAGATGAGTCCTTATATTCACAAGTGTATGGATACGCTTCATGAAATATTGGAAGAAAACATAAAGAATAACCCAGATGGATTTGACGTTTCCCCTTTACTGGCAGGCTACACACTGGATGTGATATGTAGTACTGGATTCGGCATGGATATTGACGCACAAAGAGATGTGAATAATAAATTCATCAAGTATTCAAAGGAGTTCTTGGAATTCAGAGGAACGAGGAATCTTGTCTTCCTTTTGTATATCTTCTTTCCAGACTTCGTGAAATTCTTGAACGTCACGACAAATATTGTTTCTAATGAAGCTTTTGATTTCTTCATGACAAGTTTAAGTAGAGCATTCCAGGAGAGACGACAAGACAGCTCGAGCCATCGCGACTTACTGCAGCTACTGATCAATGCGCACAAAGGAGACCTTGGGGAAAGTAAAGATGAACCTGAAGCAGCAACCCCACCTGAG |
|  |
| **8.**  **> Locus_15328_T1 Gene:** ***CfGABAT1*, Accession No. KF218344,size 510**  **RT-PCR Primers: F:GTGGTGCTGTTCAATGTGGTT**  **R:AAGCCAGACGAAGATTCCAGT** |
| TTAAGCCCAGAACGAGATTCCAGTAAGGCAGTTAAGGCACAGGATGAACATGAGCAACCGGAGCGTGACCAATGGGCCAGTGATCTGGACTTTATTCTGTCTTGTGTAGGCCTAGCCGTGGGTCTTGGAAACATATGGAGATTTCCCTATCTCTGCTACAAAAACGGAGGAGGTGCGTTTTTAATCCCGTACTTGATCTGTCTGTTCACGTGCGGTGTGCCGGTGTTTATGCTGGAGCTTGCTGTCGGACAGTACATGTCCATCGGAAGTCTAGAAGCATGGGCCAAATTCGTCCCCGCGTTCAAAGGTATTGGCATAGCAACGCTAATAGTGGTATTTCTGGCCAACCTATACTATATAGTGATTCTAGCCTGGGCAGTGTACTACCTATTCATGTCCTTCACGCCCACTCTACCCTGGTCACACTGCAACAACGACTGGAATACTGACAGATGTTTCTCAGCAGCTCGAAACGTGTCTGTTGTTTCAACCACATTGACAAGCACCACC |
| **9.** |
| **> Locus_34056_T1 Gene:** ***CfGABARAP*, Accession No. KF218343,size 276**  **RT-PCR Primers: F:GACAGAGTGCCAGTAATAGTTGAGA**  **R:GTGTTGTGCCCCTTGCTTTAT** |
| CCGGACAGAGTGCCAGTAATAGTTGAGAAGGCACCAAAAGCCCGTGTAGGAGACTTGGACAAGAAAAAGTACCTGGTACCATCAGATCTAACTGTGGGACAGTTCTATTTCCTTATTAGGAAAAGAATTCACTTGCGACCAGAGGACGCCCTGTTTTTCTTTGTTAATAATGTGATTCCACCCACCAGTGCAACTATGGGATCTCTTTACCAGGAACATCACGAGGAGGATTTCTTCTTGTACATTGCATACAGTGACGAAAGCGTTTATGGCGCA |
| **10.**  **> Locus_3281_T24 Gene:** ***CfGABARAPL2*, Accession No. KF218342,size 264**  **RT-PCR Primers: F:ATCAAAGAAAAGCCGAGTCAAC**  **R:AGAAATCCATCGTCATCCTTGT** |
| GAGTCAACGAAAATCAGAGACAAATATCCAGAGAGAATTCCTGTGATTGTAGAGAGAGATCCAAAGTCACAGATTCAAGATATTGACAAGAGGAAATTTCTGGTTCCCAATGATATTTCAGTGGCTCAGTTTATGTGGATCATCAGAAAGAGGATCCAGCTACCATCAGAGAAAGCTATCTTCCTGTTTGTAGAAAAAGTGCTGCCTCAGTCAAGTGCGAGTATGGGCCAAGTCTATGAAGAACACAAGGATGAGGTGGATTTC |
| **11.**  **> Locus_6681_T1 Gene:** ***CfHsp22*, Accession No. KF218338,size 282**  **RT-PCR Primers: F:CGGTGTGTTTTGCGTGAAC**  **R:CGTGATTTGTTTTCGGACTG** |
| CGTGATTTGTTTTCGGACTGGATTAAAGAATTCGATGATGATTGGAGATCGATGGAATTCGAGGAGTCGAGAAAACGATTCGATAGAGAGTTAGAAAGAATTAGAAAGGACCTATTTAAACTCGACACCGGTTCCACAATGCTCCAGGTTGAAAGGCCCTTTATTACGGATCCAATCGGAAATAAGAAGCTAGCGCTAAGGTTTGACTGCAGTCAATTTAAACCAGAAGAAATTAGCATAAAAACCCTGGACAAACGTTTATGTGTTCACGCAAAACACACC |
| **12.**  **> Locus_4416_T3 Gene:** ***CfHsp40*, Accession No. KF218339, size 672**  **RT-PCR Primers: F:TGGGCATTCCTTCCTTTGGT**  **R:GTCGCTGAGGCTTATGATGTG** |
| TCGCTGAAGGCTTATGATGTGTTATCAGATCCTCGAAAGCGTGCAGTGTATGATCAGTTTGGAGAAGAGGGTCTAAAGAATGGTGTTCCAAGTGGAACAGTTGAGACAGGAGCATGGACACAGGGCTACACTTTCCATGGAAATGCAGAGAAGGTGTTCAGAGATTTCTTTGGGGGAGACAATCCATTCCAAGAGTTTTATGATAGAGTGGACGGTGATTTGAGCATGTCTTTTGGTGGCTTGGCTGGGCGTGGACGAAAGAAGCAAGACCCGCCCATTGAACGTGACCTGTACCTATCATTGGAAGAAGTCTACCATGGCTGCACAAAGAAAATGAAGATTTCAAGAAGAGTTATGAATGAAGATGGTCACACATCAAGTATCCGTGACAAGATTTTGACGATTACAGTAAAGAAGGGCTGGAAACCCAATACCAGAATCACATTCCCAGAGGAAGGTGACCAAGGACCCAATAACGTACCAGCCGACATTGTTTTTATTGTCAAAGACAAGCAGCATCCCAGATTCCGCCGGGAAGGAACCAACCTAATTCACACTGCCAAAGTCCCGCTGGGAAAGGCTCTGACTGGATGTACAGTTGACATTCACACATTGGATGATAGAATGCTCCATATTCCAATCAACGACATATCAAGCCTGGATTTGTTAAGG |
| **13.**  **> Locus_11190_T1 Gene:** ***CfHsp60*, Accession No. KC979065, size 477**  **RT-PCR Primers: F:ACTTGCCAACCAACAGCGA**  **R:AATACCCTCCTCAACCGCAG** |
| GTTGACGGAGAGGCTCTCAGTACACTTGTACTCAACAGGTTGAAGGTTGGACTCCAGGTGGCAGCAGTGAAAGCCCCAGGGTTTGGGGACAACAGAAAGAACACCCTGAGGGACATGGCTATTGCTACTGGAGGTGTTGTGTTCGGTGATGAGGCAGACATGTACAAACTAGAAGATATACAGTCCAATGACTTTGGTCGTGTCGGAGAGGTTACCATCACCAAGGATGATACCCTGATAATGAAGGGCAAGGGTGATCCTGCAGATATTGAGAAAAGAATTAACCAGATCAAAGACGACATCGAGATGTCAACATCGGAGTATGAAAAGGAGAAGATGAATGAACGATTGGCCAAGTTGTCCAATGGTATTGCTCTGCTCAAGATCGGAGGAACAAGTGAAGTAGAAGTCAATGAAAAGAAAGACAGAGTAACTGATGCACTGAATGCTACGAAAGCTGCGGTTGAGGAGGGTATT |
| **14.**  **> Locus_10053_T1 Gene:** ***CfHsp70*, Accession No. KC979064, size 467**  **RT-PCR Primers: F:CGGTCGGCTCGTTGATGATT**  **R:AGCACGGGAAAGTGGAGATT** |
| CACGGGGAAGTGGAGATTATTGCGAACGACCAGGGCAACAGGACCACACCAAGCTATGTGGCGTTCACGGATTCAGAAAGACTGGTTGGAGAAGCCGCATTGAACCAGGCGGCAATGAATCCAACCAACACGGTATATGATGCCAAACGGTTAATAGGACGCAAGTTCGATGACACAAATGTTCAAAATGATATGAAGCACTGGCCATTTAAGGTAATAAGCGTCGGTGGCAGGCCGAAAATCCAGGCCGAGTTCAAGGGCGAGGTGAAAACCTTCGCTCCTGAAGAAATCAGCTCTATGGTTTTGACAAAGATGAAGGAAACTGCTGAAGCTTACCTTGGTCAAAAGATTAAAAACGCTGTTGTGACGGTGCCGGCGTACTTCAACGACGCGCAGAGACTGGCMACAAAGGATGCCGGCGTCATTGCTGGTTTGAATGTGATGAGAATCATCAACGAGCCGACCGAG |
| **15.**  **> Locus_426_T20 Gene:** ***CfHsp90*, Accession No. KC979063, size 570**  **RT-PCR Primers: F:CATCAATGCCAAGACCGAGTT**  **R:GTGTCATCAAGCGAGGGTTTG** |
| GTCATCAAGCGAGGGTTTGAGGTTATCTACATGACAGACCCCATTGATGAATATTCTGTGCAGCAACTGAAGGAGTTTGAAGGCAAGAATCTTGTGTGCGTGACAAAGGAAGGACTGGAATTGCCAGAAGATGAGGCAGAAAAGAAGAAGCGTGAAGAACAGAAGGCCGAGTTTGAGGGTCTCTGCAAGGTCATGAAGGAAATCCTTGACAAGAAAGTAGAGAAGGTCACTGTATCCAATCGATTGGTGAACTCCCCATGCTGTATTGTAACCAGTCAGTATGGATGGTCAGCCAACATGGAGCGCATCATGAAGGCCCAGGCCCTGAGAGATTCCAGCACCATGGGTTACATGGCAGCGAAGAAACACCTAGAAATCAATCCTGACCATTCAATCATCAAGGCACTCAAAGAGAAAGTCAGTGTTGACAAAAATGACAAGTCAGTGAAGGATTTGGTATTGTTGATGTTTGAGACCGCCCTCCTGGCTTCAGGATTTGCTCTGGAAGACCCAACCACACATGCCAATAGAATCAACAGAATGATCAAACTCGGTCTTGGCATTGATGAG |
